# Supplementary material for: Conversations about FGM in primary care: a realist review on how, why and under what circumstances FGM is discussed in general practice consultations
Source: BMJ Open. 2021 Mar 22;11(3):e039809. doi: 10.1136/bmjopen-2020-039809 (PMC7986780; doi:10.1136/bmjopen-2020-039809)
Supplement: Supplementary data [file bmjopen-2020-039809supp001.pdf]

English general medical practitioners managing female genital mutilation in light of recent policy and legislative changes: A realist synthesis

Sharon Dixon, Claire Duddy, Gabrielle Harrison, Chrysanthi Papoutsi, Sue Ziebland, Frances Griffiths.

Prospero: CRD4201891996

Review Question:

1. From a realist synthesis of published literature, what influences how GPs manage female genital mutilation (FGM) in their clinical practice and why?
2. What influences general medical practitioners (GPs) actions when they consider initiating discussion about female genital mutilation (FGM) with patients in primary care? Where, when and why are these influences active?
3. What influences how GPs respond to a patient-initiated disclosure of FGM during a primary care consultation? Where, when and why are these influences active?

4. **Searches:**

We will use the RAMESES quality standards for realist synthesis to guide this realist review.

We will use an information specialist to support the literature searches.

Initial programme theory will be derived from reviewing governmental policy documents relevant to the management of FGM in primary care and FGM guidance from doctors' professional bodies. The subsequent search strategy will be designed to identify empirical and theoretical literature to test the initial programme theories. The search process in a realist review is iterative and responsive to evolving programme theory. . The preliminary search will be run with keywords identified from an exploratory literature review, and will include FGM, primary care, and general practice/GP. e would search broadly, including using the following databases: Medline, Embase, PsycINFO, Global Health, Cinahl, Web of Science, Sociological Abstracts, Anthropology Plus, Social Science Abstracts and ASSIA, and undertake forward and backward citation searches to identify relevant papers

Subsequent literature searches will be responsive to emergent programme theory including potentially relevant contexts, mechanisms, and outcomes. As new theory emerges, new evidence needs to be considered to test this. We will purposively search and iteratively review evidence. All steps and decisions taken during the review will be reported and justified, including details about actions taken We will report review search strategies (initial and subsequent searches), the number of studies identified and assessed for relevance, the number and type of papers included and excluded and reasons for exclusion.

Types of study to be included:

All study designs and theoretical papers, including conceptual papers and commentaries will be considered if they contribute to the development or testing of programme theory. Papers considering interventions in health care contexts very different to the UK healthcare system will be excluded. Where there is a large body of literature, we will identify systematic reviews in the first instance. We will include papers written in English.

Condition or domain being studied:

How GPs are managing women and families from FGM affected communities

Participants/population:

GPs working in the UK

Intervention/exposure:

Any identified factor or context that may influence GPs' actions and reactions in relation to their management of patients affected by FGM.

Comparator/control:

Contexts where an identified influence is not present

Primary outcome:

This realist review will result in programme theory, tested against existing evidence, on what influences GPs actions and reactions in relation to FGM in primary care consultations and, where, when and why these influences are activated.

Timing and effect measures:

No limitation placed on timing of influences.

Effect is defined as action or reaction of GPs in relation to FGM within primary care consultations

Secondary outcome:

None

Data extraction (selection and coding):

Data extraction and organisation of the data will be undertaken by SD. Documents will be initially screened with title and abstract, and selected for inclusion for full text screening. Following full text screening, relevant articles will be included for coding in Nvivo. A random sample of 10% of finally included documents, screened abstracts, and coded full text documents will be independently checked by another member of the study team as a quality assurance measure. Disagreements will be resolved by discussion within the study team.

The full text of included papers will be uploaded onto qualitative data analysis software tool (NVivo)a. We will code text sections for their relevance (or not) to each programme theory, and within that code context, mechanisms, and outcomes. The coding structure will be initially deductive derived from the initial programme theory, and also inductive, generated to categorise data in included papers. The processes of both coding and analysis are iterative. Through cross comparison of coded data, we expect to generate theory. This may suggest the need for further interrogation of collated evidence and so new coding and searches for new evidence.

Risk of bias (quality) assessment:

The study will be guided by the RAMESES quality standards for realist reviews (http://www.ramesesproject.org/Standards_and_Training_materials.php#qual_stand_rs)

Papers will be appraised for relevance to the theory under test, not necessarily the topic under consideration, and for the rigour of the conclusions the authors drew. All included papers will be assessed independently by two team members.

Strategy for data synthesis:

Data analysis and synthesis will use a realist logic of analysis. In a realist review, the process of data analysis and synthesis co-exist and overlap. In realist terms, synthesis is progress towards explanations, which are framed as relationships between the contexts (C) in which outcomes under observation occur (O), and the mechanisms that potentially link these (M). These relationships are postulated as CMO configurations (CMOC), which are considered within over-arching programme theory. During analysis, we will use interpretive cross comparison, to understand and explain how different interventions have produced outcomes in different contexts and how we understand the potential mechanisms that link these. During CMOC development, we will review how the data supports the developing CMOCs, how new CMOCs relate to other postulated CMOCs and how they relate to programme theory being developed. The synthesis will include retroductive analysis seeking to identify hidden causes that can explain observed patterns in the data.

Analysis of subgroups or subsets:

We will potentially be testing more than one programme theory

.
