# Supplementary material for: Conversations about FGM in primary care: a realist review on how, why and under what circumstances FGM is discussed in general practice consultations
Source: BMJ Open. 2021 Mar 22;11(3):e039809. doi: 10.1136/bmjopen-2020-039809 (PMC7986780; doi:10.1136/bmjopen-2020-039809)
Supplement: Supplementary data [file bmjopen-2020-039809supp002.pdf]

BMJ Publishing Group Limited (BMJ) disclaims all liability and responsibility arising from any reliance placed on this supplemental material which has been supplied by the author(s)

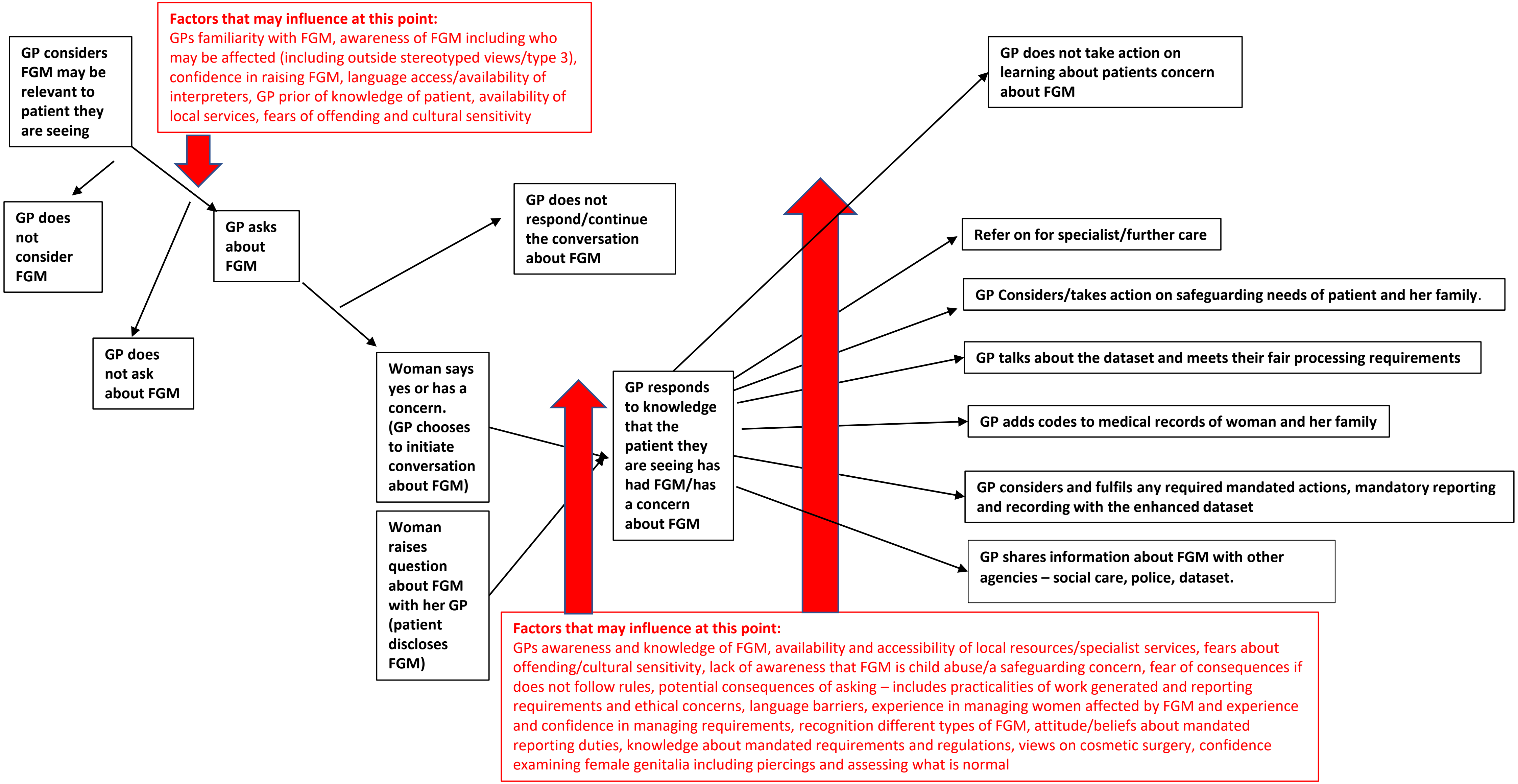

The initial programme theory mapped onto a primary care consultation. The black boxes represent hypothesised possible outcomes and the red text and boxes represent potential contextual or mechanistic factors identified from an exploratory literature review and stakeholder expertise.
