# Supplementary material for: Conversations about FGM in primary care: a realist review on how, why and under what circumstances FGM is discussed in general practice consultations
Source: BMJ Open. 2021 Mar 22;11(3):e039809. doi: 10.1136/bmjopen-2020-039809 (PMC7986780; doi:10.1136/bmjopen-2020-039809)
Supplement: Supplementary data [file bmjopen-2020-039809supp003.pdf]

Appendix C: Search strategies

Initial scoping searches (March 2016)

Ovid MEDLINE Search #1

▲ Searches

1 Circumcision, Female/

2 female genital mutilation.ti,ab.

3 ((female? or women or girl*) adj2 circumcis*).ti,ab.

4 ((genital? adj2 (cut or cuts or cutting)) and (female? or women or girl*)).ti,ab.

5 1 or 2 or 3 or 4

6 Prevalence/

7 *"surveys and questionnaires"/ or health care surveys/ or health surveys/

8 (prevalen* or burden or trend? or estimat* or survey?).ti.

9 6 or 7 or 8

10 5 and 9

11 limit 10 to yr="2006 -Current"

Ovid MEDLINE Search #2

#▲ Searches

1 Circumcision, Female/

2 female genital mutilation.ti,ab.

3 ((female? or women or girl*) adj2 circumcis*).ti,ab.

4 ((genital? adj2 (cut or cuts or cutting)) and (female? or women or girl*)).ti,ab.

5 1 or 2 or 3 or 4

6 (Qualitative systematic review* or (systematic review and qualitative)).ti,ab.

7 (evidence synthesis or realist synthesis).ti,ab.

8 (Qualitative and synthesis).ti,ab.

9 (meta-synthesis* or meta synthesis* or metasynthesis).ti,ab.

10 (meta-ethnograph* or metaethnograph* or meta ethnograph*).ti,ab.

11 (meta-study or metastudy or meta study).ti,ab.

12 (realist review? or realist synthesis).ti,ab.

13 systematic review*.ti,ab. and qualitative research/

14 6 or 7 or 8 or 9 or 10 or 11 or 12 or 13

15 5 and 14

16 qualitative research/

17 *interviews as topic/ or focus groups/ or narration/

18 observation.ti.

19 interview?.ti.

20 (qualitative adj2 (interview* or study)).ti,ab.

21 (qualitative or focus group? or story or stories or narration or narrative* or discourse or discursive or grounded theory or ethnogra* or phenomenolog*).ti,ab.

22 16 or 17 or 18 or 19 or 20 or 21

23 5 and 22

24 15 or 23

Global Health Search #1

▲ Searches

1 female genital mutilation.ti,ab.

2 ((female? or women or girl*) adj2 circumcis*).ti,ab.

3 ((genital? adj2 (cut or cuts or cutting)) and (female? or women or girl*)).ti,ab.

4 1 or 2 or 3

5 Prevalence/

6 surveys/ or household surveys/

7 (prevalen* or burden or trend? or estimat* or survey?).ti.

8 5 or 6 or 7

9 4 and 8

10 limit 9 to yr="2006 -Current"

Global Health Search #2

#

▲

Searches

1 female genital mutilation.ti,ab.

2 ((genital? adj2 (cut or cuts or cutting)) and (female? or women or girl*)).ti,ab.

3 ((female? or women or girl*) adj2 circumcis*).ti,ab.

4 1 or 2 or 3

5 (qualitative or focus group? or story or stories or narration or narrative* or discourse or discursive or grounded theory or ethnogra* or phenomenolog*).ti,ab.

6 interview*.ti,ab.

7 5 or 6

8 4 and 7

Embase

▲ Searches

1 exp female genital mutilation/

2 female genital mutilation.ti,ab.

3 ((female? or women or girl*) adj2 circumcis*).ti,ab.

4 ((genital? adj2 (cut or cuts or cutting)) and (female? or women or girl*)).ti,ab.

5 1 or 2 or 3 or 4

6 (Qualitative systematic review* or (systematic review and qualitative)).ti,ab.

7 (evidence synthesis or realist synthesis).ti,ab.

8 (Qualitative and synthesis).ti,ab.

9 (meta-synthesis* or meta synthesis* or metasynthesis).ti,ab.

10 (meta-ethnograph* or metaethnograph* or meta ethnograph*).ti,ab.

11 (meta-study or metastudy or meta study).ti,ab.

12 (realist review? or realist synthesis).ti,ab.

13 systematic review*.mp. and exp qualitative studies/

14 6 or 7 or 8 or 9 or 10 or 11 or 12 or 13

15 5 and 14

16 grounded theory/ or naturalistic inquiry/ or qualitative research/

17 exp *interview/

18 observation.ti.

19 interview?.ti.

20 (qualitative adj2 (interview* or study)).ti,ab.

21 (qualitative or focus group? or story or stories or narration or narrative* or discourse or discursive or grounded theory or ethnogra* or phenomenolog*).ti,ab.

22 16 or 17 or 18 or 19 or 20 or 21

23 5 and 22

24 15 or 23

PsycINFO

▲ Searches

1 circumcision/

2 female genital mutilation.ti,ab.

3 ((genital? adj2 (cut or cuts or cutting)) and (female? or women or girl*)).ti,ab.

4 ((female? or women or girl*) adj2 circumcis*).ti,ab.

5 1 or 2 or 3 or 4

6 (Qualitative systematic review* or (systematic review and qualitative)).ti,ab.

7 (evidence synthesis or realist synthesis).ti,ab.

8 (Qualitative and synthesis).ti,ab.

9 (meta-synthesis* or meta synthesis* or metasynthesis).ti,ab.

10 (meta-ethnograph* or metaethnograph* or meta ethnograph*).ti,ab.

11 (meta-study or metastudy or meta study).ti,ab.

12 (realist review? or realist synthesis).ti,ab.

13 systematic review*.ti,ab. and qualitative research/

14 6 or 7 or 8 or 9 or 10 or 11 or 12 or 13

15 5 and 14

16 qualitative research/ or grounded theory/ or observation methods/

17 exp *interviews/

18 observation.ti.

19 interview?.ti.

20 (qualitative adj2 (interview* or study)).ti,ab.

21 (qualitative or focus group? or story or stories or narration or narrative* or discourse or discursive

or grounded theory or ethnogra* or phenomenolog*).ti,ab.

22 16 or 17 or 18 or 19 or 20 or 21

23 5 and 22

24 15 or 23

These search strategies were also adapted and run in

• ASSIA,

• Sociological Abstracts

• CINAHL

• Anthropology Plus

• Web of Science (Core Collection databases)

A total of 1554 references were screened by title and abstract, 181 were read in full text and 22 contributed to the synthesis.

Main FGM searches (August 2017)

Summary of searching and results

Database Interface Coverage Date GP

hits

UK

Hits

Ovid MEDLINE(R) Epub

Ahead of Print, In-Process &

Other Non-Indexed

Citations, Ovid MEDLINE(R)

Daily and Ovid MEDLINE(R)

OvidSp 1946-present 22/08/2017 21 384

Embase OvidSp 1974 to 2017

August 21

22/08/2017 31 396

PsycINFO OvidSp 1967 to August

Week 2 2017

22/08/2017 0 57

CINAHL EBSCOHost 1982-present 22/08/2017 16 575

Web of Science Core

Collection

Thomson

Reuters

1945-present 22/08/2017 0 280

Nexis UK Lexis Nexis 22/08/2017 0 260

Proquest Social Science

Databases

Proquest 22/08/2017 5 100

MEDLINE

#

▲

Searches

1 Circumcision, Female/

2 (femal genital mutilation or fgm).ti,ab.

3 ((genital? adj2 (cut or cuts or cutting)) and (female? or women or girls)).ti,ab.

4 ((female? or women or girl*) adj2 circumcis*).ti,ab.

5 1 or 2 or 3 or 4

6 exp General Practice/

7 general practitioners/ or physicians, family/ or physicians, primary care/

8 Primary Health Care/

9 (((general or family) adj2 (practi* or physician? or doctor?)) or gp or gps).ti,ab.

10 (doctor? or physician?).ti.

11 (primary adj2 (care or healthcare)).ti,ab.

12 6 or 7 or 8 or 9 or 10 or 11

13 exp United Kingdom/

14 (united kingdom or uk or britain or gb or england or wales or scotland or northern ireland).ti,ab,in.

15 (nhs or national health service).ti,ab,in.

16 (british or bjgp or bmj or hsj or pulse or gp or general practi* or primary care or nursing standard or nursing times).jw.

17 13 or 14 or 15 or 16

18 5 and 12 and 17

19 5 and 17

Embase

#

▲

Searches

1 female genital mutilation/

2 (femal genital mutilation or fgm).ti,ab.

3 ((genital? adj2 (cut or cuts or cutting)) and (female? or women or girls)).ti,ab.

4 ((female? or women or girl*) adj2 circumcis*).ti,ab.

5 1 or 2 or 3 or 4

6 General Practice/

7 general practitioner/

8 Primary Medical Care/

9 (((general or family) adj2 (practi* or physician? or doctor?)) or gp or gps).ti,ab.

10 (doctor? or physician?).ti.

11 (primary adj2 (care or healthcare)).ti,ab.

12 6 or 7 or 8 or 9 or 10 or 11

13 exp United Kingdom/

14 (united kingdom or uk or britain or gb or england or wales or scotland or northern ireland).ti,ab,in.

15 (nhs or national health service).ti,ab,in.

16 (british or bjgp or bmj or hsj or pulse or gp or general practi* or primary care or nursing standard or nursing times).jw.

17 13 or 14 or 15 or 16

18 5 and 12 and 17

19 5 and 17

PsycINFO

#

▲

Searches

1 (exp Human Females/ or exp Female Genitalia/) and exp Circumcision/

2 (femal genital mutilation or fgm).ti,ab.

3 ((genital? adj2 (cut or cuts or cutting)) and (female? or women or girls)).ti,ab.

4 ((female? or women or girl*) adj2 circumcis*).ti,ab.

5 1 or 2 or 3 or 4

6 general practitioners/

7 Primary Health Care/

8 (((general or family) adj2 (practi* or physician? or doctor?)) or gp or gps).ti,ab.

9 (doctor? or physician?).ti.

10 (primary adj2 (care or healthcare)).ti,ab.

11 6 or 7 or 8 or 9 or 10

12 (united kingdom or uk or britain or gb or england or wales or scotland or northern ireland).ti,ab,in.

13 (nhs or national health service).ti,ab,in.

14 (british or bjgp or bmj or hsj or pulse or gp or general practi* or primary care or nursing standard or nursing times).jw.

15 12 or 13 or 14

16 5 and 11 and 15

17 5 and 15

CINAHL

Query

S19 S5 AND S17

S18 S5 AND S12 AND S17

S17 S13 OR S14 OR S15 OR S16

S16 SO british or bjgp or bmj or hsj or pulse or gp or general practi* or primary care or nursing standard or nursing times)

S15 TX nhs or "national health service"

S14 TX "united kingdom" or uk or britain or gb or england or wales or scotland or "northern ireland"

S13 (MH "United Kingdom+")

S12 S6 OR S7 OR S8 OR S9 OR S10 OR S11

S11 TI ((primary N2 (care or healthcare))) OR AB ((primary N2 (care or healthcare)))

S10 TI doctor? or physician?

S9 TI ((((general or family) N2 (practi* or physician? or doctor?)) or gp or gps)) OR AB ((((general or family) N2 (practi* or physician? or doctor?)) or gp or gps))

S8 (MH "Primary Health Care")

S7 (MH "Physicians, Family")

S6 (MH "Family Practice")

S5 S1 OR S2 OR S3 OR S4

S4 TI (((female? or women or girl*) N2 circumcis*)) OR AB (((female? or women or girl*) N2 circumcis*))

S3 TI (((genital? N2 (cut or cuts or cutting)) and (female? or women or girls))) OR AB (((genital? N2 (cut or cuts or cutting)) and (female? or women or girls)))

S2 TI ("femal genital mutilation" or fgm) OR AB ("femal genital mutilation" or fgm)

S1 (MH "Circumcision, Female")

Web of Science (Core Collection)

Set Results

5 280 #3 AND #1

4 0 #3 AND #2 AND #1

3 5,132,695 TOPIC: ("united kingdom" or uk or britain or gb or england or wales or scotland or "northern ireland") OR ADDRESS: ("united kingdom" or uk or britain or gb or england or wales or scotland or "northern ireland") OR TOPIC: (nhs or "national health service") OR ADDRESS: (nhs or "national health service") OR PUBLICATION NAME: (british or bjgp or bmj or hsj or pulse or gp or general practi* or primary care or nursing standard or nursing times)

2 339,275 TOPIC: ((((general or family) NEAR/2 (practi* or physician? or doctor?)) or gp or gps)) OR TITLE: (doctor? or physician?) OR TOPIC: ((primary NEAR/2 (care or healthcare)))

1 5,251 TOPIC: ("femal genital mutilation" or fgm) OR TOPIC: (((genital? NEAR/2 (cut or cuts or cutting)) and (female? or women or girls))) OR TOPIC: (((female? or women or girl*) NEAR/2 circumcis*))

Nexis UK

fgm OR "female genital mutilation" In the Headline

gp OR gps OR general practitioners OR doctors Anywhere in the text

Limits

Previous 2 year

UK publications

Proquest Social Science Databases

Set Search

S7 S3 AND S5

S6 S3 AND S4 AND S5

S5 all("united kingdom" or uk or britain or gb or england or wales or scotland or "northern ireland") OR all(nhs OR "national health service")

S4 all(((general or family) NEAR/2 (practi* or physician or doctor)) or gp or gps) OR ti(doctor OR physician) OR all("primary care" OR "primary healthcare" OR "primary health care")

S3 all("femal genital mutilation" or fgm) OR all((((genital? NEAR/2 (cut or cuts or cutting)) and (female? or women or girls)))) OR all((((female? or women or girl*) NEAR/2 circumcis*)))

A total of 2052 references were screened by title and abstract, 73 were read in full text.

Website searches

In addition to these searches, a search of relevant advocacy organisation websites was undertaken, including searching:

• Sharon add a list of links to relevant organisation homepages here – just illustrative not everything, say, maybe 5?

Update searches (July 2018, April 2019)

Summary of searching and results (July 2018)

Database Interface Coverage Date GP

hits

UK

Hits

Ovid MEDLINE(R) Epub

Ahead of Print, In-Process &

Other Non-Indexed

Citations, Ovid MEDLINE(R)

Daily and Ovid MEDLINE(R)

OvidSp 1946-present 22/08/2017 21 384

Embase OvidSp 1974 to 2017

August 21

22/08/2017 31 396

PsycINFO OvidSp 1967 to August

Week 2 2017

22/08/2017 0 57

CINAHL EBSCOHost 1982-present 22/08/2017 16 575

Web of Science Core

Collection

Thomson

Reuters

1945-present 22/08/2017 0 280

Nexis UK Lexis Nexis 22/08/2017 0 260

Proquest Social Science

Databases

Proquest 22/08/2017 5 100

Update searches in July 2018 replicated the Main FGM searches shown above, limited to results added to databases from August 2017 onwards.

Summary of searching and results (April 2019)

Database Interface Coverage Date GP

hits

Other

health

profs

Hits

Ovid MEDLINE(R) Epub

Ahead of Print, In-Process

& Other Non-Indexed

Citations, Ovid

MEDLINE(R) Daily and

Ovid MEDLINE(R)

OvidSp 1946-present 23/04/2019 2 7

Embase OvidSp 1974 to 2017

August 21

23/04/2019 1 31

PsycINFO OvidSp 1967 to August

Week 2 2017

23/04/2019 0 0

CINAHL EBSCOHost 1982-present 23/04/2019 3 10

Web of Science Core

Collection

Thomson

Reuters

1945-present 23/04/2019 7 Not

searched

Nexis UK Lexis Nexis 23/04/2019 90 Not

searched

Proquest Social Science

Databases

Proquest 23/04/2019 3 48

Update searches in April 2019 replicated the Main FGM searches shown above, limited to results added to databases from July 2018 onwards. For pragmatic reasons, WoS Core Collection and Nexis UK were only searched for terms relating to GPs/primary care and not other health professional groups.

A total of 429 new references were identified in the update searches and screened by title and abstract, 92 were read in full text.

Supplementary searches (Various dates)

Summary of searching and results: DVLA, IPV/DVA and Prevent searches (August 2017)

Database Interface Coverage Date DVLA IPV Prevent

Ovid MEDLINE(R) Epub

Ahead of Print, InProcess & Other NonIndexed Citations, Ovid

MEDLINE(R) Daily and

Ovid MEDLINE(R)

OvidSp 1946-present 22/08/2017 52 142 7

Embase OvidSp 1974 to 2017

August 21

22/08/2017 137 244 9

PsycINFO OvidSp 1967 to August

Week 2 2017

22/08/2017 8 69 1

CINAHL EBSCOHost 1982-present 22/08/2017 53 399 69

Web of Science Core

Collection

Thomson

Reuters

1945-present 22/08/2017 74 150 14

Proquest Social Science

Databases

Proquest 22/08/2017 10 57 16

Summary of searching and results: Mandatory reporting search (May 2018)

Database Interface Coverage Date Results

PubMed PubMed 1946-present May 2018

Web of Science (Core

Collection)

Thomson

Reuters

1945-present May 2018

DVLA (August 2017)

MEDLINE

#

▲

Searches

1 ("driver and vehicle licensing agency" or dvla).ti,ab.

2 ((driving or driver?) adj5 (notif* or report* or inform*)).ti,ab.

3 ((driving or driver?) adj5 (ability or competen* or incompeten* or status or continu*

or discontinu* or stop* or quit* or cease* or cessation)).ti,ab.

4 ((driving or driver?) adj3 (licens* or law* or legal*)).ti,ab.

5 (driv* adj3 (fit or fitness)).ti,ab.

6 Licensure/ and Automobile Driving/

7 1 or 2 or 3 or 4 or 5 or 6

8 exp General Practice/

9 general practitioners/ or physicians, family/ or physicians, primary care/

10 Primary Health Care/

11 (((general or family) adj2 (practi* or physician? or doctor?)) or gp or gps).ti,ab.

12 (doctor? or physician?).ti.

13 (primary adj2 (care or healthcare)).ti,ab.

14 8 or 9 or 10 or 11 or 12 or 13

15 exp United Kingdom/

16 (united kingdom or uk or britain or gb or england or wales or scotland or northern ireland).ti,ab,in.

17 (nhs or national health service).ti,ab,in.

18 (british or bjgp or bmj or hsj or pulse or gp or general practi* or primary care or nursing standard or nursing times).jw.

19 15 or 16 or 17 or 18

20 7 and 14 and 19

Embase

#

▲

Searches

1 ("driver and vehicle licensing agency" or dvla).ti,ab.

2 ((driving or driver?) adj5 (notif* or report* or inform*)).ti,ab.

3 ((driving or driver?) adj5 (ability or competen* or incompeten* or status or continu* or discontinu* or stop* or quit* or cease* or cessation)).ti,ab.

4 ((driving or driver?) adj3 (licens* or law* or legal*)).ti,ab.

5 (driv* adj3 (fit or fitness)).ti,ab.

6 driving ability/ or driver licence/

7 1 or 2 or 3 or 4 or 5 or 6

8 General Practice/

9 general practitioner/

10 Primary Medical Care/

11 (((general or family) adj2 (practi* or physician? or doctor?)) or gp or gps).ti,ab.

12 (doctor? or physician?).ti.

13 (primary adj2 (care or healthcare)).ti,ab.

14 8 or 9 or 10 or 11 or 12 or 13

15 exp United Kingdom/

16 (united kingdom or uk or britain or gb or england or wales or scotland or northern ireland).ti,ab,in.

17 (nhs or national health service).ti,ab,in.

18 (british or bjgp or bmj or hsj or pulse or gp or general practi* or primary care or nursing standard or nursing times).jw.

19 15 or 16 or 17 or 18

20 7 and 14 and 19

PsycINFO

▲ Searches

1 ("driver and vehicle licensing agency" or dvla).ti,ab.

2 ((driving or driver?) adj5 (notif* or report* or inform*)).ti,ab.

3 ((driving or driver?) adj5 (ability or competen* or incompeten* or status or continu* or discontinu* or stop* or quit* or cease* or cessation)).ti,ab.

4 ((driving or driver?) adj3 (licens* or law* or legal*)).ti,ab.

5 (driv* adj3 (fit or fitness)).ti,ab.

6 1 or 2 or 3 or 4 or 5

7 general practitioners/

8 Primary Health Care/

9 (((general or family) adj2 (practi* or physician? or doctor?)) or gp or gps).ti,ab.

10 (doctor? or physician?).ti.

11 (primary adj2 (care or healthcare)).ti,ab.

12 7 or 8 or 9 or 10 or 11

13 (united kingdom or uk or britain or gb or england or wales or scotland or northern

ireland).ti,ab,in.

14 (nhs or national health service).ti,ab,in.

15 (british or bjgp or bmj or hsj or pulse or gp or general practi* or primary care or nursing standard or nursing times).jw.

16 13 or 14 or 15

17 6 and 12 and 16

CINAHL

S6 S3 AND S4 AND S5

S5 (((driving or driver?) N5 (notif* or report* or inform*))) OR (((driving or driver?) N5 (ability or competen* or incompeten* or status or continu* or discontinu* or stop* or quit* or cease* or cessation))) OR (((driving or driver?) N3 (licens* or law* or legal*))) OR ((driv* N3 (fit or fitness))) OR ("driver and vehicle licensing agency" or dvla)

S4 (MH "United Kingdom+") OR (AB "united kingdom" or uk or britain or gb or england or wales or scotland or "northern ireland") OR (AB nhs or "national health service") OR (SO british or bjgp or bmj or hsj or pulse or gp or general practi* or primary care or nursing standard or nursing times) OR (TI "united kingdom" or uk or britain or gb or england or wales or scotland or "northern ireland") OR (TI nhs or "national health service")

S3 S1 OR S2

S2 TI ((((general or family) N2 (practi* or physician? or doctor?)) or gp or gps)) OR AB ((((general or family) N2 (practi* or physician? or doctor?)) or gp or gps)) OR TI (doctor? or physician?) OR TI ((primary N2 (care or healthcare))) OR AB ((primary N2 (care or healthcare)))

S1 (MH "Family Practice") OR (MH "Physicians, Family") OR (MH "Primary Health Care")

Web of Science (Core Collection)

#

4

74 #3 AND #2 AND #1

#

3

36,160 TS=("driver and vehicle licensing agency" or dvla) OR TS=(((driving or driver?) NEAR/5 (notif* or report* or inform*))) OR TS=(((driving or driver?) NEAR/5

(ability or competen* or incompeten* or status or continu* or discontinu* or

stop* or quit* or cease* or cessation))) OR TS=(((driving or driver?) NEAR/3

(licens* or law* or legal*))) OR TS=((driv* NEAR/3 (fit or fitness)))

#

2

5,132,695 TS=("united kingdom" or uk or britain or gb or england or wales or scotland or

"northern ireland") OR ADDRESS: ("united kingdom" or uk or britain or gb or

england or wales or scotland or "northern ireland") OR TS=(nhs or "national

health service") OR ADDRESS: (nhs or "national health service") OR

PUBLICATION NAME: (british or bjgp or bmj or hsj or pulse or gp or general

practi* or primary care or nursing standard or nursing times)

#

1

339,275 TS=((((general or family) NEAR/2 (practi* or physician? or doctor?)) or gp or

gps)) OR TI=(doctor? or physician?) OR TS=((primary NEAR/2 (care or

healthcare)))

Proquest Social Science Databases

S6 S1 AND S2 AND S5

S5 all("driver and vehicle licensing agency" OR dvla) OR all((driving OR driver) NEAR/5 (notif* OR report* OR inform*)) OR all(((driving OR driver) NEAR/5 (ability OR competen* OR incompeten* OR status OR continu* OR discontinu* OR stop* OR quit* OR cease* OR cessation))) OR all(((driving OR driver) NEAR/3 (licens* OR law* OR legal*))) OR all((driv* NEAR/3 (fit OR fitness)))

S2 all(((general or family) NEAR/2 (practi* or physician or doctor)) or gp or gps) OR ti(doctor OR physician) OR all("primary care" OR "primary healthcare" OR "primary health care")

S1 all("united kingdom" or uk or britain or gb or england or wales or scotland or "northern ireland") OR all(nhs OR "national health service")

A total of 131 citations were screened by title and abstract, 6 were screened in full text and none were included in the final synthesis.

Prevent (August 2017)

MEDLINE

#

▲ Searches

1 (prevent adj (program* or strateg* or initiative or policy)).ti,ab.

2

(prevent* and (terroris* or counterterroris* or antiterroris* or extremis* or counterextremis* or antiextremis* or radicali* or counterradicali* or antiradicali*)).ti.

3

(prevent* adj5 (terroris* or counterterroris* or antiterroris* or extremis* or counterextremis* or antiextremis* or radicali* or counterradicali* or antiradicali*)).ti,ab.

4 1 or 2 or 3

5 exp General Practice/

6 general practitioners/ or physicians, family/ or physicians, primary care/

7 Primary Health Care/

8 (((general or family) adj2 (practi* or physician? or doctor?)) or gp or gps).ti,ab.

9 (doctor? or physician?).ti.

10 (primary adj2 (care or healthcare)).ti,ab.

11 5 or 6 or 7 or 8 or 9 or 10

12 exp United Kingdom/

13

(united kingdom or uk or britain or gb or england or wales or scotland or northern ireland).ti,ab,in.

14 (nhs or national health service).ti,ab,in.

15

(british or bjgp or bmj or hsj or pulse or gp or general practi* or primary care or nursing standard or nursing times).jw.

16 12 or 13 or 14 or 15

17 4 and 11 and 16

Embase

#

▲ Searches

1 (prevent adj (program* or strateg* or initiative or policy)).ti,ab.

2

(prevent* and (terroris* or counterterroris* or antiterroris* or extremis* or counterextremis* or antiextremis* or radicali* or counterradicali* or antiradicali*)).ti.

3

(prevent* adj5 (terroris* or counterterroris* or antiterroris* or extremis* or counterextremis* or antiextremis* or radicali* or counterradicali* or antiradicali*)).ti,ab.

4 1 or 2 or 3

5 General Practice/

6 general practitioner/

7 Primary Medical Care/

8 (((general or family) adj2 (practi* or physician? or doctor?)) or gp or gps).ti,ab.

9 (doctor? or physician?).ti.

10 (primary adj2 (care or healthcare)).ti,ab.

11 5 or 6 or 7 or 8 or 9 or 10

12 exp United Kingdom/

13

(united kingdom or uk or britain or gb or england or wales or scotland or northern ireland).ti,ab,in.

14 (nhs or national health service).ti,ab,in.

15

(british or bjgp or bmj or hsj or pulse or gp or general practi* or primary care or nursing standard or nursing times).jw.

16 12 or 13 or 14 or 15

17 4 and 11 and 16

PsycINFO

▲ Searches

1 (prevent adj (program* or strateg* or initiative or policy)).ti,ab.

2

(prevent* and (terroris* or counterterroris* or antiterroris* or extremis* or counterextremis* or antiextremis* or radicali* or counterradicali* or antiradicali*)).ti.

3

(prevent* adj5 (terroris* or counterterroris* or antiterroris* or extremis* or counterextremis* or antiextremis* or radicali* or counterradicali* or antiradicali*)).ti,ab.

4 1 or 2 or 3

5 general practitioners/

6 Primary Health Care/

7 (((general or family) adj2 (practi* or physician? or doctor?)) or gp or gps).ti,ab.

8 (doctor? or physician?).ti.

9 (primary adj2 (care or healthcare)).ti,ab.

10 5 or 6 or 7 or 8 or 9

11

(united kingdom or uk or britain or gb or england or wales or scotland or northern ireland).ti,ab,in.

12 (nhs or national health service).ti,ab,in.

13

(british or bjgp or bmj or hsj or pulse or gp or general practi* or primary care or nursing standard or nursing times).jw.

14 11 or 12 or 13

15 4 and 10 and 14

CINAHL

S12 S3 AND S4 AND S11

S11 AB ((prevent N1 (program* or strateg* or initiative or policy))) OR TI ((prevent* and (terroris* or counterterroris* or antiterroris* or extremis* or counterextremis* or antiextremis* or radicali* or counterradicali* or antiradicali*))) OR AB ((prevent* N5 (terroris* or counterterroris* or antiterroris* or extremis* or counterextremis* or antiextremis* or radicali* or counterradicali* or antiradicali*)))

S4 (MH "United Kingdom+") OR (AB "united kingdom" or uk or britain or gb or england or wales or scotland or "northern ireland") OR (AB nhs or "national health service") OR (SO british or bjgp or bmj or hsj or pulse or gp or general practi* or primary care or nursing standard or nursing times) OR (TI "united kingdom" or uk or britain or gb or england or wales or scotland or "northern ireland") OR (TI nhs or "national health service")

S3 S1 OR S2

S2 TI ((((general or family) N2 (practi* or physician? or doctor?)) or gp or gps)) OR AB ((((general or family) N2 (practi* or physician? or doctor?)) or gp or gps)) OR TI (doctor? or physician?) OR TI ((primary N2 (care or healthcare))) OR AB ((primary N2 (care or healthcare)))

S1 (MH "Family Practice") OR (MH "Physicians, Family") OR (MH "Primary Health Care")

Web of Science (Core Collection)

8 14 #7 AND #2 AND #1

7 2,755 TS=((prevent NEXT (program* or strateg* or initiative or policy))) OR TI=((prevent* and (terroris* or counterterroris* or antiterroris* or extremis* or counterextremis* or antiextremis* or radicali* or counterradicali* or antiradicali*))) OR TS=((prevent* NEAR/5 (terroris* or counterterroris* or antiterroris* or extremis* or counterextremis* or antiextremis* or radicali* or counterradicali* or antiradicali*)))

2 5,132,695 TS=("united kingdom" or uk or britain or gb or england or wales or scotland or "northern ireland") OR ADDRESS: ("united kingdom" or uk or britain or gb or england or wales or scotland or "northern ireland") OR TS=(nhs or "national health service") OR ADDRESS: (nhs or "national health service") OR PUBLICATION NAME: (british or bjgp or bmj or hsj or pulse or gp or general practi* or primary care or nursing standard or nursing times)

1 339,275 TS=((((general or family) NEAR/2 (practi* or physician? or doctor?)) or gp or gps)) OR TI=(doctor? or physician?) OR TS=((primary NEAR/2 (care or healthcare)))

Proquest Social Science Databases

Set Search

S11 S1 AND S2 AND S10

S10 all((prevent NEXT (program* OR strateg* OR initiative OR policy))) OR ti((prevent* AND (terroris* OR counterterroris* OR antiterroris* OR extremis* OR counterextremis* OR antiextremis* OR radicali* OR counterradicali* OR antiradicali*))) OR all((prevent* NEAR/5 (terroris* OR counterterroris* OR antiterroris* OR extremis* OR counterextremis* OR antiextremis* OR radicali* OR counterradicali* OR antiradicali*)))

S2 all(((general or family) NEAR/2 (practi* or physician or doctor)) or gp or gps) OR ti(doctor OR physician) OR all("primary care" OR "primary healthcare" OR "primary health care")

S1 all("united kingdom" or uk or britain or gb or england or wales or scotland or "northern ireland") OR all(nhs OR "national health service")

A total of 22 citations were screened by title and abstract, 17 were screened in full text and 5 were included in the final synthesis.

IPV/DVA (August 2017)

MEDLINE

▲ Searches

1 domestic violence/ or spouse abuse/

2 ((domestic or spous* or partner? or wife or wives or husband) adj2 (violence or abuse*)).ti,ab.

3 (batter* adj2 (spous* or partner? or wife or wives or husband)).ti,ab.

4 1 or 2 or 3

5 exp General Practice/

6 general practitioners/ or physicians, family/ or physicians, primary care/

7 Primary Health Care/

8 (((general or family) adj2 (practi* or physician? or doctor?)) or gp or gps).ti,ab.

9 (doctor? or physician?).ti.

10 (primary adj2 (care or healthcare)).ti,ab.

11 5 or 6 or 7 or 8 or 9 or 10

12 exp United Kingdom/

13

(united kingdom or uk or britain or gb or england or wales or scotland or northern ireland).ti,ab,in.

14 (nhs or national health service).ti,ab,in.

15

(british or bjgp or bmj or hsj or pulse or gp or general practi* or primary care or nursing standard or nursing times).jw.

16 12 or 13 or 14 or 15

17 4 and 11 and 16

Embase

▲ Searches

1 domestic violence/ or battered woman/ or family violence/ or exp partner violence/

2 ((domestic or spous* or partner? or wife or wives or husband) adj2 (violence or abuse*)).ti,ab.

3 (batter* adj2 (spous* or partner? or wife or wives or husband)).ti,ab.

4 1 or 2 or 3

5 General Practice/

6 general practitioner/

7 Primary Medical Care/

8 (((general or family) adj2 (practi* or physician? or doctor?)) or gp or gps).ti,ab.

9 (doctor? or physician?).ti.

10 (primary adj2 (care or healthcare)).ti,ab.

11 5 or 6 or 7 or 8 or 9 or 10

12 exp United Kingdom/

13

(united kingdom or uk or britain or gb or england or wales or scotland or northern ireland).ti,ab,in.

14 (nhs or national health service).ti,ab,in.

15

(british or bjgp or bmj or hsj or pulse or gp or general practi* or primary care or nursing standard or nursing times).jw.

16 12 or 13 or 14 or 15

17 4 and 11 and 16

PsycINFO

▲ Searches

1 domestic violence/ or battered females/ or intimate partner violence/ or exp partner abuse/

2 ((domestic or spous* or partner? or wife or wives or husband) adj2 (violence or abuse*)).ti,ab.

3 (batter* adj2 (spous* or partner? or wife or wives or husband)).ti,ab.

4 1 or 2 or 3

5 general practitioners/

6 Primary Health Care/

7 (((general or family) adj2 (practi* or physician? or doctor?)) or gp or gps).ti,ab.

8 (doctor? or physician?).ti.

9 (primary adj2 (care or healthcare)).ti,ab.

10 5 or 6 or 7 or 8 or 9

11

(united kingdom or uk or britain or gb or england or wales or scotland or northern ireland).ti,ab,in.

12 (nhs or national health service).ti,ab,in.

13

(british or bjgp or bmj or hsj or pulse or gp or general practi* or primary care or nursing standard or nursing times).jw.

14 11 or 12 or 13

15 4 and 10 and 14

CINAHL

S10 S3 AND S4 AND S9

S9 S7 OR S8

S8 AB (((domestic or spous* or partner? or wife or wives or husband) N2 (violence or abuse*))) OR AB ((batter* N2 (spous* or partner? or wife or wives or husband))) OR TI (((domestic or spous* or partner? or wife or wives or husband) N2 (violence or abuse*))) OR TI ((batter* N2 (spous* or partner? or wife or wives or husband)))

S7 (MH "Domestic Violence") OR (MH "Intimate Partner Violence")

S4 (MH "United Kingdom+") OR (AB "united kingdom" or uk or britain or gb or england or wales or scotland or "northern ireland") OR (AB nhs or "national health service") OR (SO british or bjgp or bmj or hsj or pulse or gp or general practi* or primary care or nursing standard or nursing times) OR (TI "united kingdom" or uk or britain or gb or england or wales or scotland or "northern ireland") OR (TI nhs or "national health service")

S3 S1 OR S2

S2 TI ((((general or family) N2 (practi* or physician? or doctor?)) or gp or gps)) OR AB ((((general or family) N2 (practi* or physician? or doctor?)) or gp or gps)) OR TI (doctor? or physician?) OR TI ((primary N2 (care or healthcare))) OR AB ((primary N2 (care or healthcare)))

S1 (MH "Family Practice") OR (MH "Physicians, Family") OR (MH "Primary Health Care")

Web of Science (Core Collection)

6 150 #5 AND #2 AND #1

5 15,568 TS=(((domestic or spous* or partner? or wife or wives or husband) NEAR/2 (violence or abuse*))) OR TS=((batter* NEAR/2 (spous* or partner? or wife or wives or husband)))

2 5,132,695 TS=("united kingdom" or uk or britain or gb or england or wales or scotland or

"northern ireland") OR ADDRESS: ("united kingdom" or uk or britain or gb or england or wales or scotland or "northern ireland") OR TS=(nhs or "national health service") OR ADDRESS: (nhs or "national health service") OR PUBLICATION NAME: (british or bjgp or bmj or hsj or pulse or gp or general practi* or primary care or nursing standard or nursing times)

1 339,275 TS=((((general or family) NEAR/2 (practi* or physician? or doctor?)) or gp or gps)) OR TI=(doctor? or physician?) OR TS=((primary NEAR/2 (care or healthcare)))

Proquest Social Science Databases

S8 S1 AND S2 AND S7

S7 all(((domestic or spous* or partner? or wife or wives or husband) NEAR/2 (violence or abuse*))) OR all((batter* NEAR/2 (spous* or partner? or wife or wives or husband)))

S2 all(((general or family) NEAR/2 (practi* or physician or doctor)) or gp or gps) OR ti(doctor OR physician) OR all("primary care" OR "primary healthcare" OR "primary health care")

S1 all("united kingdom" or uk or britain or gb or england or wales or scotland or "northern ireland") OR all(nhs OR "national health service")

A total of 352 citations were screened by title and abstract, 62 were screened in full text and 15 were included in the final synthesis.

Mandatory reporting

PubMed

Search Query

#29 Select 6 document(s) Filters: published in the last 10 years; English

#28 Search (((((relig* OR muslim* OR islam* or "far right") AND (extremis* OR terror* or radicalisation OR radicalization)) OR (((relig* OR muslim* OR islam* or "far right" OR extremis* OR terror* or radicalisation OR radicalization) AND ((prevent strateg*[Title/Abstract]) OR prevent program*[Title/Abstract])))) AND "last 10 years"[PDat] AND English[lang])) AND ((uk OR "united kingdom" OR gb OR britian OR england OR wales OR scotland OR "northern ireland" OR nhs OR british) AND "last 10 years"[PDat] AND English[lang]) Filters: published in the last 10 years; English

#27 Search uk OR "united kingdom" OR gb OR britian OR england OR wales OR scotland OR "northern ireland" OR nhs OR british Filters: published in the last 10 years;

English

#26 Search ((relig* OR muslim* OR islam* or "far right") AND (extremis* OR terror* or radicalisation OR radicalization)) OR (((relig* OR muslim* OR islam* or "far right" OR extremis* OR terror* or radicalisation OR radicalization) AND ((prevent strateg*[Title/Abstract]) OR prevent program*[Title/Abstract]))) Filters: published in the last 10 years; English

#24 Search (((((relig* OR muslim* OR islam* or "far right") AND (extremis* OR terror* or radicalisation OR radicalization))) AND ("last 10 years"[PDat] AND English[lang] AND (infant[MeSH] OR child[MeSH] OR adolescent[MeSH])))) OR (((((relig* OR muslim* OR islam* or "far right" OR extremis* OR terror* or radicalisation OR radicalization) AND ("last 10 years"[PDat] AND English[lang] AND (infant[MeSH] OR child[MeSH] OR adolescent[MeSH])))) AND (((prevent strateg*[Title/Abstract]) OR prevent program*[Title/Abstract]) AND ("last 10 years"[PDat] AND English[lang] AND (infant[MeSH] OR child[MeSH] OR adolescent[MeSH])))) AND ("last 10 years"[PDat] AND English[lang] AND (infant[MeSH] OR child[MeSH] OR adolescent[MeSH]))) Filters: published in the last 10 years; English

#23 Search (((((relig* OR muslim* OR islam* or "far right") AND (extremis* OR terror* or radicalisation OR radicalization))) AND ("last 10 years"[PDat] AND English[lang] AND (infant[MeSH] OR child[MeSH] OR adolescent[MeSH])))) OR (((((relig* OR muslim* OR islam* or "far right" OR extremis* OR terror* or radicalisation OR radicalization) AND ("last 10 years"[PDat] AND English[lang] AND (infant[MeSH] OR child[MeSH] OR adolescent[MeSH])))) AND (((prevent strateg*[Title/Abstract]) OR prevent program*[Title/Abstract]) AND ("last 10 years"[PDat] AND English[lang] AND (infant[MeSH] OR child[MeSH] OR adolescent[MeSH])))) AND ("last 10 years"[PDat] AND English[lang] AND (infant[MeSH] OR child[MeSH] OR adolescent[MeSH]))) Filters: published in the last 10 years; English; Child: birth-18 years

#22 Search (((relig* OR muslim* OR islam* or "far right" OR extremis* OR terror* or radicalisation OR radicalization) AND ("last 10 years"[PDat] AND English[lang] AND (infant[MeSH] OR child[MeSH] OR adolescent[MeSH])))) AND (((prevent strateg*[Title/Abstract]) OR prevent program*[Title/Abstract]) AND ("last 10

years"[PDat] AND English[lang] AND (infant[MeSH] OR child[MeSH] OR adolescent[MeSH]))) Filters: published in the last 10 years; English; Child: birth-18 years

#21 Search relig* OR muslim* OR islam* or "far right" OR extremis* OR terror* or radicalisation OR radicalization Filters: published in the last 10 years; English; Child: birth-18 years

#20 Search (prevent strateg*[Title/Abstract]) OR prevent program*[Title/Abstract] Filters: published in the last 10 years; English; Child: birth-18 years

#19 Search ((relig* OR muslim* OR islam* or "far right") AND (extremis* OR terror* or radicalisation OR radicalization)) Filters: published in the last 10 years; English; Child: birth-18 years

#15 Search "moral imperative" OR "ethical imperative" OR "emotional imperative" Filters: published in the last 10 years; English; Child: birth-18 years

#14 Search "moral imperative" OR "ethical imperative" OR "emotional imperative" Filters: published in the last 10 years; Child: birth-18 years

#13 Search "moral imperative" OR "ethical imperative" OR "emotional imperative" Filters: Child: birth-18 years

#12 Search ((mandatory report*[tiab] OR mandated report*[tiab] OR mandatory notif*[tiab] OR mandated notif*[tiab])) OR (mandat*[ti] AND (report*[ti] OR notif*[ti])) Filters: Child: birth-18 years

#10 Search ((mandatory report*[tiab] OR mandated report*[tiab] OR mandatory notif*[tiab] OR mandated notif*[tiab])) OR (mandat*[ti] AND (report*[ti] OR notif*[ti]))

#11 Search ((mandatory report*[tiab] OR mandated report*[tiab] OR mandatory notif*[tiab] OR mandated notif*[tiab])) OR (mandat*[ti] AND (report*[ti] OR notif*[ti])) Filters: Systematic Reviews

#9 Search mandat*[ti] AND (report*[ti] OR notif*[ti])

#8 Search mandatory report*[tiab] OR mandated report*[tiab] OR mandatory notif*[tiab] OR mandated notif*[tiab]

#7 Search (((physician*[Title] OR doctor*[Title] OR general practi*[Title] OR family physician*[Title] OR "primary care"[Title]))) AND ((educat*[Title] OR learn*[Title]

OR teach*[Title] OR train*[Title])) Filters: Systematic Reviews; published in the last 10 years; English

#6 Search (((physician*[Title] OR doctor*[Title] OR general practi*[Title] OR family physician*[Title] OR "primary care"[Title]))) AND ((educat*[Title] OR learn*[Title] OR teach*[Title] OR train*[Title])) Filters: Systematic Reviews; English

#5 Search (((physician*[Title] OR doctor*[Title] OR general practi*[Title] OR family physician*[Title] OR "primary care"[Title]))) AND ((educat*[Title] OR learn*[Title] OR teach*[Title] OR train*[Title])) Filters: Systematic Reviews

#4 Search (((physician*[Title] OR doctor*[Title] OR general practi*[Title] OR family physician*[Title] OR "primary care"[Title]))) AND ((educat*[Title] OR learn*[Title] OR teach*[Title] OR train*[Title]))

#3 Search (educat*[Title] OR learn*[Title] OR teach*[Title] OR train*[Title])

#2 Search (physician*[Title] OR doctor*[Title] OR general practi*[Title] OR family physician*[Title] OR "primary care"[Title])

Web of Science (Core Collection)

29 8 #25 AND #23

28 88 #27 AND #10

27 521 #23 AND #21

26 63 TS=("prevent strateg*" OR "prevent program*")

25 165 TS=("prevent strateg*" OR "prevent program*")

24 521 #23 AND #21

23 1,576,775 TS=(physician* OR doctor* OR "general practi" OR "family practi*" OR "primary care" OR healthcare OR "health care") OR TI=(health* OR medicine* OR nurs*)

22 2 #21 AND #12

21 5,487 #20 OR #19

20 1,960 TS=((relig* OR muslim* OR islam* or "far right") NEAR/5 (extremis* OR terror* or radicalisation OR radicalization))

19 3,531 TS=("home office" OR "illegal migrant*" OR "illegal immigra*" OR "undocumented migran*" OR "undocumented immigran*" OR illegals)

18 363 #15 OR #13 Refined by: PUBLICATION YEARS: (2018 OR 2008 OR 2017 OR

2016 OR 2015 OR 2014 OR 2013 OR 2012 OR 2011 OR 2010 OR 2009) AND

[excluding] DOCUMENT TYPES: (EDITORIAL MATERIAL OR MEETING

ABSTRACT OR BOOK REVIEW OR LETTER OR NEWS ITEM OR PROCEEDINGS

PAPER)

17 405 #15 OR #13 Refined by: PUBLICATION YEARS: (2018 OR 2008 OR 2017 OR

2016 OR 2015 OR 2014 OR 2013 OR 2012 OR 2011 OR 2010 OR 2009)

16 597 #15 OR #13

15 321 #14 AND #12

14 64,467 TS=((child* OR infant* OR teen* OR adolescen*) NEAR/3 (abuse* OR

violence OR maltreat*)) OR TI=((child* OR infant* OR teen* OR adolescen*)

AND (abuse* OR violence OR maltreat*)) OR TS=(safeguarding OR "safe

guarding")

13 290 #12 AND #10

12 2,582 TOPIC: ((mandat* OR compulsory) NEAR/3 (report* OR notif*)) OR TITLE:

((mandat* OR compulsory) AND (report* OR notif*))

11 64 #10 AND #9

10 5,653,563 TOPIC: ("united kingdom" or uk or britain or british or gb or england or

"northern ireland" or scotland or wales or nhs) OR ADDRESS: ("united

kingdom" or uk or britain or british or gb or england or "northern ireland"

or scotland or wales or nhs)

9 787 TOPIC: ("cultural competenc*") AND TOPIC: (physician* OR doctor* OR

"general practi*" OR "family practi" OR "primary care")

8 128 TITLE: ("cultural competenc*") AND TOPIC: (educat* OR learn* OR teach*

OR train*) AND TOPIC: (physician* OR doctor* OR "general practi*" OR

"family practi" OR "primary care")

7 179 TOPIC: ("moral imperative" OR "ethical imperative" OR "emotional

imperative") AND TOPIC: (educat* OR learn* OR teach* OR train*)

6 4 TOPIC: ("moral imperative" OR "ethical imperative" OR "emotional

imperative") AND TOPIC: ((child* OR adolecen* OR teen* OR infan*)

NEAR/5 (abuse* OR violence OR maltreat*))

5 0 TITLE: ("moral imperative" OR "ethical imperative" OR "emotional

imperative") AND TOPIC: ((child* OR adolecen* OR teen* OR infan*) NEAR/5 (abuse* OR violence OR maltreat*))

4 287 TITLE: ("moral imperative" OR "ethical imperative" OR "emotional imperative")

3 1 TOPIC: ("moral imperative" OR "ethical imperative" OR "emotional imperative") AND TOPIC: (((mandat* OR compulsory) NEAR/2 (report* OR notif*)))

2 55 TOPIC: ("moral imperative" OR "ethical imperative" OR "emotional imperative") AND TOPIC: (doctor* OR physician* OR "general practi*" OR "family practi*")

1 891 TOPIC: ("moral imperative" OR "ethical imperative" OR "emotional imperative")

A total of 88 citations were screened by title and abstract, 36 were screened in full text and 10 were included in the final synthesis.
