# Supplementary material for: Conversations about FGM in primary care: a realist review on how, why and under what circumstances FGM is discussed in general practice consultations
Source: BMJ Open. 2021 Mar 22;11(3):e039809. doi: 10.1136/bmjopen-2020-039809 (PMC7986780; doi:10.1136/bmjopen-2020-039809)
Supplement: Supplementary data [file bmjopen-2020-039809supp004.pdf]

Appendix D. Full set of derived CMOC/full programme theory.

The need for FGM knowledge and awareness:

1) Lacking knowledge or skills (including about the cultural contexts of FGM, safeguarding requirements, who might be affected, and the different types of FGM and their clinical consequences) impacts on GPs' ability to provide optimal care for women affected by FGM Practitioners may not be aware that they lack knowledge, including which patients may be affected and their care needs (C). This lack of knowledge (M) results in their inability to meet their care needs. (O) (1-23).
2) Lacking the necessary knowledge and skills (C), is associated with a lack of confidence (M) which impacts on clinical care for women with FGM (O) (10, 24, 25). This includes having the knowledge and confidence to consider who may be at risk (26).
3) Feeling that they have adequate knowledge (including how to respond to a disclosure) (C) helped clinicians feel confident (M) to ask to ask (O) (27, 28).
4) Women who perceive healthcare professionals lack knowledge and skills to manage FGM (C), or who have experiences stigma (C) may lack confidence that health services (M) will meet their care needs (O) (5, 10,24, 29-33).
5) (C)Health professionals experience emotional reactions to encountering FGM such as anger, shock, and pity; they find encountering FGM without adequate knowledge is "frightening"(35)(M). Professionals try to hide their reactions but are aware their reaction may be apparent to the women (21, 23, 34-37) (O).
6) Community members see health care providers reacting with shock or horror to their FGM (C). This provokes feelings of shame (M) which reduces their likelihood of accessing services (O) (22, 38-42) .
7) However, clinicians confident in managing FGM (C) are able to reassure women (M) and meet their care needs (O) (28, 43, 44).
8) Healthcare professionals may experience a strong emotional responses to FGM (C). This may make them feel panicked or frightened (M) and abandon their usual routines and practices (O) (28, 93).
9) FGM is usually a relatively small part of the GP workload (C). GPs may not identify learning about FGM as a priority (M) for themselves. The GPs then lack knowledge and skills (O)(45,46).
10) Clinicians who encounter FGM more frequently in the line of their work (C), may become sensitised to FGM (M) and motivated (M) to learn more or develop their knowledge and skills (O). The converse may also be true (8, 12, 14, 47).
11) FGM can be difficult to correctly identify (48), especially types 1 and 4 and associated with less symptoms (C) and GPs may not have the expertise or confidence (M) to correctly identify or manage FGM (O) (20, 49-53).

Talking about FGM and communication:

12) A key skill GPs need is being able to talk about FGM sensitively). Fears (M) of offending women by not knowing how to raise the issue(C) can lead GPs to avoid talking about FGM (O) (4,, 10, 16, 21, 22, 23, 29, 35, 48, 54-57) .
13) Professionals who understand that FGM can be sensitive or taboo subject (C) may be fearful of offending women (M) and avoid discussing FGM with women (O) (23,29,54, 55, 58,59). This contextual factor may be evolving as community attitudes towards the practice of FGM change including meaning that talking about FGM is less taboo in some communities (37, 60-64, 92).
14) Not being aware of or not recognising evolving community practices (C) risks (M) offending community members thereby reducing effective communication/consultations (O) (37). Or that professionals are not able to accurately appraise risk (11) (61).
15) Raising FGM in a way that is normalised within the consultation, for example as a standard question on an assessment form (C) may reduce the embarrassment (M) and facilitate asking (O) (23). Prompts in the records may help clinicians to do this (23, 65).

16) Challenges around the use of terminology can complicate communication between GPs and their patients affected by FGM. Some women may find the terms FGM offensive or frightening , or if the term FGM is not familiar to the woman (C)), or she does not align her cultural practice (for example labial elongation) with FGM (C), then she may not relate her experience to FGM (M) or know how to reply if a GP asks her about FGM(O) (O) (66-70).
17) Women's experiences of poor communication and difficulties in engagement with health professionals (e.g. language, cultural differences, perceived judgement), and including non-verbal communication, led to them feeling not understood or respected (M), causing a lack of confidence and trust in health services (O) (21, 29, 30, 39, 71, 72).
18) Women who feel pitied or judged(C) may be reluctant (M) to make a disclosure to a health care professional (O) (73, 74).
19) Members of communities affected by FGM acting as health advocates (C) may help promote trust and educate communities and professionals (M) to facilitate access to services (O) (75-77).
20) Language barriers and a lack of understanding of how the health setting works, including communicating with primary care receptionists (C) can make accessing services difficult or stressful (M) and lead to avoidance (O) (78).
21) Whether FGM is relevant to the health concern which the woman brings to her GP appointment (C), could impact on whether the GP or woman are willing to raise or discuss FGM (M), and how such a conversation may be received or experienced (O) (36, 37, ,60, 67, 79). This may apply when GPs consider asking women about FGM to consider safeguarding needs within their families, rather than because of their own health needs (27, 37, 80,81)) .
22) Coding FGM into medical records introduces potential tensions around balancing the needs of the woman (and her confidentiality) with the potential need of her family (C) which may cause confusion or uncertainty for GPs (M), and lead to improvised strategies or inconsistent coding (O) (81- 83).
23) Women who perceive that the HCP is preoccupied with their FGM (C), can feel disrespected (M) and disengage with health care settings (O) (37,60, 84, 85) .
24) GPs gender (C) may influence whether the woman or GP feel it is culturally appropriate (M) to talk about FGM (O) (23,38, 55, 86).
25) Time pressures in the consultation (C) mean GPs may be reluctant (M) to discuss FGM (O). (23, 46, 55, 87)
26) Language barriers are a significant context which influence the conversations between GPs and women about FGM (C) which reduce communication (M) between women and GPs with impacts on communication effectiveness and their care (O) (4, 17, 21,23, 29, 55, 57, 58, 78, 86, 88).
27) Strategies to address language barriers add their own complications. Official interpreters are recommended, but may not be available or trusted by women, for example if they both perceive FGM as taboo, or she fears they will not respect her confidentiality. This can lead to fear (M) and reduced engagement with health professionals (O) (4, 6, 17, 23,29, 35, 36, 38, 55,56, 89,90).
28) The presence of family members (as interpreters, or witnesses) in the consultation (C) may inhibit GPs feeling able to raise FGM (O) with the women, because of concerns about privacy and confidentiality (M) (4, , 55, 58, 91).

The need for guidelines and access to specialist services:

29) Researchers and commentators suggest that having access to clear and supportive guidelines about what clinicians should do (C) will enable professionals (M) to ask women about FGM and optimise their care (O)(19, 20,35,92,93). Even when guidelines exist, awareness of them may be incomplete, or they may not be followed, as demonstrated by four UK hospital studies (47, 56, 57, 94). The reasons for this in the case of FGM warrant exploration. Having prompts to normalise asking about FGM may help clinicians broach the subject (95), especially if linked to training or referral pathways(96).

30) FGM is a complex area for health care professionals to manage, and this management may include needing to report women and their families to other authorities. If professionals are developing awareness of FGM, without accompanying guidance (C), they may experience uncertainty and face what they experience as ethical tensions (M) and risk making incorrect or uncertain decisions regarding reporting (O) (92,93)
31) Lacking guidance, including guidelines and certainty about what good care comprises (C) can lead to practitioners feeling uncertain (M) and improvising how they offer care (O) (22, 23).
32) Knowing how to react or having access to specialist services (C) may help GPs and community members feel confident (M) to talk about FGM (O) (23, 29, 52, 53, 72, 97).
33) When Health care professionals speak about FGM within a framework of offering support and services (C), it is more likely to be experienced as acceptable by the woman (M, O) (64).
34) Training is more likely to be effective (C) in changing behaviour and promoting asking (O) when it is supported by resources and referral pathways or protocol for intervention (M) (28, 73, 96, 98). Specialist access may be especially important to support practitioners in low prevalence areas (23).

Mandated actions including mandatory reporting and the FGM Enhanced dataset requirements:

35) The mandatory reporting duty (C) may cause distress and reduce trust (M) in professionals which may deter women from seeking help or disclosing their FGM (O) (52, 55, 60, 62, 99-101).
36) Concerns that medical encounters or records are not confidential may cause fear/apprehension (M) and deter women from a disclosure of her needs or concerns (O) (46, 91, 102).
37) The requirement to submit personally identifiable data to the FGM enhanced dataset (C) may reduce women's trust in the confidentiality of the GP consultation (M) and make her reluctant to disclose FGM (O) or make GPs reluctant to raise FGM (O) because of concerns about confidentiality (M) (55, 60, 75, 103-106).
38) The ways in which mandatory reporting or the enhanced dataset are raised in the consultation, including when this happens repeatedly (C), may lead women to feel that the professionals' interest in more in data collection than them, or make them feel judged or fearful (M), and avoid attending healthcare altogether (O) (60)
39) The concern (M) that making a mandated report (C) would have a potentially negative impact on trust (O) in on-going professional relationships (O) was an important potential consideration for professionals (107-110) and identified as a potential deterrent for help seeking (108,111).
40) Another concern about managing the legislative requirements includes that FGM can be difficult to identify on examination. GPs may feel that they do not have the skills needed to identify or manage FGM and so not feel confident in being able to identify FGM to confidently code it (O)(48-50) (46), which may impact on data accuracy (O) (112). Education (C) is needed to help practitioners feel able (M) to approach mandatory reporting (O) (122).
41) Practitioners making mandated reports need to feel confident that their report will be adequately responded to, without causing harm (113-114). They may be helped by training (113).
42) In addition to lack of knowledge or training (C), practitioners may have concerns about confidentiality (C/M) or fear of causing stigma (C/M) which leads to incomplete or inaccurate coding of FGM (115). Practitioners may perceive a need to feel certain (C) before making a mandated report (O) so that they do not risk making a mistake (M)(116-117).
43) When young people know that the professional whom they are speaking to is mandated to share the information with other authorities (C), they may feel more reluctant to trust the professional (M), and less likely to make a disclosure (O) (118).
44) A Perceptions of how trustworthy the authority being referred onto may contribute to decisions as to whether or not to disclose. Those who are potentially fearful of authorities or perceive themselves to be vulnerable (C), for example those with uncertain migrant status (C), or if they fear that their disclosure risks placing others at risk of trouble (c), may be more fearful of mandatory reporting or data sharing (M) and avoid accessing services (O) (107)(119-124)

References:

1. Khaja K, Lay K, Boys S. Female Circumcision: Toward an Inclusive Practice of Care. Health Care for Women International. 2010;31(8):686-99.
2. Leval A, Widmark C, Tishelman C, Ahlberg BM. THE ENCOUNTERS THAT RUPTURE THE MYTH: CONTRADICTIONS IN MIDWIVES' DESCRIPTIONS AND EXPLANATIONS OF CIRCUMCISED WOMEN IMMIGRANTS' SEXUALITY. Health Care for Women International. 2010;25(8):743-60.
3. Odemerho BI, Baier M. Female Genital Cutting and the Need for Culturally Competent Communication. The Journal for Nurse Practitioners. 2012;8(6):452-7.
4. Lazar JN, Johnson-Agbakwu CE, Davis OI, Shipp MPL. Providers' Perceptions of Challenges in Obstetrical Care for Somali Women. Obstetrics and Gynecology International. 2013;2013:149640.
5. Johnson-Agbakwu CE, Helm T, Killawi A, Padela AI. Perceptions of obstetrical interventions and female genital cutting: insights of men in a Somali refugee community. Ethnicity and Health. 2014;19(4):440-57.
6. Byrskog U, Olsson P, Essén B, Allvin M-K. Being a bridge: Swedish antenatal care midwives' encounters with Somali-born women and questions of violence; a qualitative study. BMC Pregnancy and Childbirth. 2015;15(1).
7. Turkmani S, Homer C, Varol N, Dawson A. A survey of Australian midwives' knowledge, experience, and training needs in relation to female genital mutilation. Women and Birth. 2018;31(1):25-30
8. Tantet C, Aupiais C, Bourdon M, Sorge F, Pagès A, Levy D, et al. Female genital mutilation: an evaluation of the knowledge of French general and specialized travel medicine practitioners. Journal of Travel Medicine. 2018;25(1):tax090.
9. González-Timoneda A, Ros VR, González-Timoneda M, Sánchez AC. Knowledge, attitudes and practices of primary healthcare professionals to female genital mutilation in Valencia, Spain: are we ready for this challenge? BMC health services research. 2018;18(1):579.
10. Vangen S, Johansen REB, Sundby J, Træen B, Stray-Pedersen B. Qualitative study of perinatal care experiences among Somali women and local health care professionals in Norway. European Journal of Obstetrics & Gynecology and Reproductive Biology. 2004;112(1):29-35.
11. Tamaddon L, Johnsdotter S, Liljestrand J, Essén B. Swedish Health Care Providers' Experience and Knowledge of Female Genital Cutting. Health Care for Women International. 2006;27(8):709-22.
12. Kaplan-Marcusan A, Torán-Monserrat P, Moreno-Navarro J, Fàbregas MJC, Muñoz-Ortiz L. Perception of primary health professionals about Female Genital Mutilation: from healthcare to intercultural competence. BMC Health Services Research. 2009;9(11).
13. Leye E, Ysebaert I, Deblonde J, Claeys P, Vermeulen G, Jacquemyn Y, et al. Female genital mutilation: Knowledge, attitudes and practices of Flemish gynaecologists. The European Journal of Contraception and Reproductive Health Care. 2009;13(2):182-90.
14. Zaidi N, Khalil A, Roberts C, Browne M. Knowledge of female genital mutilation among healthcare professionals. Journal of Obstetrics and Gynecology. 2009;27(2):161-4.
15. Relph S, Inamdar R, Singh H, Yoong W. Female genital mutilation/cutting: knowledge, attitude and training of health professionals in inner city London. European Journal of Obstetrics & Gynecology and Reproductive Biology. 2013;168(2):195-8.
16. Cappon S, L'Ecluse C, Clays E, Tency I, Leye E. Female genital mutilation: Knowledge, attitude and practices of Flemish midwives. Midwifery. 2015;31(3):e29-e35.
17. Dawson A, Homer CS, Turkmani S, Black K, Varol N. A systematic review of doctors' experiences and needs to support the care of women with female genital mutilation. International Journal of Gynecology & Obstetrics. 2015;131(1):35-40.
18. Dawson A, Turkmani S, Fray S, Nanayakkara S, Varol N, Homer C. Evidence to inform education, training and supportive work environments for midwives involved in the care of women with female genital mutilation: A review of global experience. Midwifery. 2015;31(1):229-38.
19. Zurynski Y, Sureshkumar P, Phu A, Elliott E. Female genital mutilation and cutting: a systematic literature review of health professionals' knowledge, attitudes and clinical practice. BMC international health and human rights. 2015;15:32.
20. Reig-Alcaraz M, Siles-González J, Solano-Ruiz C. A mixed-method synthesis of knowledge, experiences and attitudes of health professionals to Female Genital Mutilation. Journal of Advanced Nursing. 2015;72(2):245-60.
21. Smith H, Stein K. Health information interventions for female genital mutilation. Int ernational Journal of Gynecology and Obstetrics. 2017;136:(136:):79-82.

22. Jordal M, Wahlberg A. Challenges in providing quality care for women with female genital cutting in Sweden–A literature review. Sexual & Reproductive Healthcare. 2018;17:91-6.
23. Evans C, Tweheyo R, McGarry J, Eldridge J, Albert J, Nkoyo V, et al. Crossing cultural divides: A qualitative systematic review of factors influencing the provision of healthcare related to female genital mutilation from the perspective of health professionals. PloS one. 2019;14(3):e0211829.
24. Dawson AJ, Turkmani S, Varol N, Nanayakkara S, Sullivan E, Homer CS. Midwives' experiences of caring for women with female genital mutilation: Insights and ways forward for practice in Australia. Women and Birth. 2015;28(3):207-14.
25. Nash E, Ranka P, editors. FEMALE GENITAL MUTILATION: KNOWLEDGE, CONFIDENCE, AND APPROACH TO CARE IN CLINICAL PRACTICE OF MIDWIVES AND NURSES IN THE UK. 21st FIGO World Congress of Gynecology and Obstetrics; 2015; Vancouver, BC Canada: International Journal of Gynecology and Obstetrics.
26. Ramsay J, Rutterford C, Gregory A, Dunne D, Eldridge S, Sharp D, et al. Domestic violence: knowledge, attitudes, and clinical practice of selected UK primary healthcare clinicians. British Journal of General Practice. 2012;62(602):e647-e55.
27. Taft, A., Broom, D.H. and Legge, D., 2004. General practitioner management of intimate partner abuse and the whole family: qualitative study. *Bmj*, *328*(7440), p.618.
28. Sundborg E, Törnkvist L, Saleh-Stattin N, Wändell P, Hylander I. To ask, or not to ask: the hesitation process described by district nurses encountering women exposed to intimate partner violence. Journal of Clinical Nursing. 2017;26(15-16):2256-65.
29. Norman K, Hemmings J, Hussein E, Otoo-Oyortey N. FGM is always with us: Experiences, Perceptions and Beliefs of Women Affected by Female Genital Mutilation in London. Results from a PEER study London: Options Consultancy Services Ltd

FORWARD; 2009.
30. Hussein E, FORWARD. Women's Experiences, perceptions, and attitudes of female Genital Mutilation

The Bristol PEER study FORWARD; 2010.
31. Glover J, Liebling H, Barrett… H. The psychological and social impact of female genital mutilation: A holistic conceptual framework. 2017.
32. Norman K, Belay Gegzabher S, Otoo-Oyortey N. “Between Two Cultures”: A Rapid PEER Study Exploring Migrant Communities’ Views on Female Genital Mutilation in Essex and Norfolk, UK. London: National FGM Centre

FORWARD; 2016.
33. Mbanya VN, Gele AA, Diaz E, Kumar B. Health care-seeking patterns for female genital mutilation/cutting among young Somalis in Norway. BMC Public Health. 2018;18(1):517.
34. Ogunsiji O. Female Genital Mutilation (FGM): Australian Midwives’ Knowledge and Attitudes. Health Care for Women International. 2015;36(11):1179-93.
35. Widmark C, Tishelman C, Ahlberg BM. A study of Swedish midwives' encounters with infibulated African women in Sweden. Midwifery. 2002;18(2):113-25.
36. Horowitz CR, Jackson JC. Female “circumcision” African women confront American medicine. Journal of General Internal Medicine. 1997;12(8):491-9.
37. Johnsdotter S, Essén B. Cultural change after migration: Circumcision of girls in Western migrant communities. Best Practice & Research Clinical Obstetrics & Gynaecology. 2016;32:15-25.
38. Abdullahi A, Copping J, Kessel A, Luck M, Bonell C. Cervical screening: Perceptions and barriers to uptake among Somali women in Camden. Public Health. 2009;123(10):680-5.
39. Oguntoye S, Otoo-Oyortey N, Hemmings J, Norman K, Hussein E. “FGM is with us Everyday”: Women and Girls Speak out about Female Genital Mutilation in the UK. World Academy of Science, Engineering and Technology. 2009;54:1020-5.
40. Hamid A, Grace KT, Warren N. A Meta-Synthesis of the Birth Experiences of African Immigrant Women Affected by Female Genital Cutting. Journal of Midwifery & Women's Health. 2018;63(2):185-95.
41. Recchia N, McGarry J. “Don’t judge me”: narratives of living with FGM. International Journal of Human Rights in Healthcare. 2017;10(1):4-13.
42. del Mar Pastor-Bravo M, Almansa-Martínez P, Jiménez-Ruiz I. Living with mutilation: A qualitative study on the consequences of female genital mutilation in women's health and the healthcare system in Spain. Midwifery. 2018;66:119-26.

43. Vloeberghs E, van der Kwaak A, Knipscheer J, van den Muijsenbergh M. Coping and chronic psychosocial consequences of female genital mutilation in the Netherlands. Ethnicity and Health. 2013;17(6):677-95.
44. Moxey JM, Jones LL. A qualitative study exploring how Somali women exposed to female genital mutilation experience and perceive antenatal and intrapartum care in England. BMJ Open. 2016;6(1):e0009846.
45. Baillot H, Murray N, Connelly E, Howard N. Addressing female genital mutilation in Europe: a scoping review of approaches to participation, prevention, protection, and provision of services. International Journal for Equity in Health. 2018;17(1):21.
46. Baillot H, Murray N, Connelly E, Howard N. Tackling Female Genital Mutilation in Scotland: A Scottish Model of Intervention. Scotland: Scottish Refugee Council

London School of Hygiene and Tropical Medicine 2014.
47. Purchase T, Lamoudi M, Colman S, Allen S, Latthe P, Jolly K. A survey on knowledge of female genital mutilation guidelines - Purchase - 2013 - Acta Obstetricia et Gynecologica Scandinavica - Wiley Online Library. ACTA Obstetricia et Gynecologica Scandinavia. 2013(92:):858–86.
48. Abdulcadir J, Dugerdil A, Boulvain M, Yaron M, Margairaz C, Irion O, et al. Missed opportunities for diagnosis of female genital mutilation. International Journal of Gynecology & Obstetrics. 2014;125(3):256-60.
49. Hodes D, Armitage A, Robinson K, Creighton SM. Female genital mutilation in children presenting to a London safeguarding clinic: a case series. Archives of Disease in Childhood. 2016;101(3):212-6.
50. Hodes D, Armitage A, Dykes A, editors. Female genital mutilation in London and the UNICEF report; a local perspective on worldwide statistics. Annual Conference of the Royal College of Paediatrics and Child Health, RCPCH 2014; 2014 2014-04-01; Birmingham, UK: Archives of Disease in Childhood.
51. Ayadi O'Donnell N, Pall K, Leoni M, Debelle G, Lynn R, Armitage A, et al., editors. Female genital mutilation (FGM) surveillance in under 16 years olds in the UK and Ireland. Royal College of Paediatrics and Child Health Annual Conference, RCPCH 2018; 2018 2018-03-01; United Kingdom: Archives of Disease in Childhood.
52. Mathers N, Rymer J. Mandatory reporting of female genital mutilation by healthcare professionals. British Journal of General Practice. 2015;65(635):282-3.
53. RCGP. Female Genital Mutilation : a clinical approach for GPs 2017 [updated under review 8.3.2017. Available from: http://www.rcgp.org.uk/policy/rcgp-policy-areas/female-genital-mutilation.aspx.
54. Johansen REB. Care for Infibulated Women Giving Birth in Norway: An Anthropological Analysis of Health Workers' Management of a Medically and Culturally Unfamiliar Issue. Medical Anthropology Quarterly. 2006;20(4):516-44.
55. Clayton-Hathaway K. A pilot evaluation of health services for communities affected by FGM/C in Oxfordshire. Oxfordshire: Oxford Against Cutting

Healthwatch Oxfordshire; 2016.
56. Zenner N, Liao LM, Richens Y, Creighton SM. Quality of obstetric and midwifery care for pregnant women who have undergone female genital mutilation. Journal of Obstetrics and Gynecology. 2013;33(5):459-62.
57. Gabrasadig R, Asamoah F, Wilson N, editors. Female genital mutilation: knowledge, training and experience of healthcare professionals at a London hospital. Annual Conference of the Royal College of Paediatrics and Child Health, RCPCH 2015; 2015 2015-04-01; Birmingham, UK: Archives of Disease in Childhood.
58. Upvall MJ, Mohammed K, Dodge PD. Perspectives of Somali Bantu refugee women living with circumcision in the United States: A focus group approach. International Journal of Nursing Studies. 2009;46(3):360-8.
59. Safari F. A qualitative study of women's lived experience after deinfibulation in the UK. Midwifery. 2013;29(2):154-8.
60. Karlsen S, Carver N, Mogilnicka M, Pantazis C. When safeguarding becomes stigmatising: A report on the impact of FGM-safeguarding procedures on people with a Somali heritage living in Bristol. Bristol: University of Bristol; 2019.
61. Creighton SM, Samuel Z, Otoo-Oyortey N, Hodes D. Tackling female genital mutilation in the UK. BMJ. 2019;364:l15.
62. FORWARD. "A Big Wake-Up Call": Participatory Study on Shifts in Attitudes Towards FGM Amongst Community Women in Bristol, Summary Report. FORWARD

Refugee Women of Bristol; 2017.
63. Brown E, Porter C. The Tackling FGM Initiative: Evaluation of the Second Phase

(2013-2016). London: Options Consultancy Services Ltd; 2016.
64. Brown E, Porter C, Unit OP. Evaluation of FGM Prevention among Communities Affected by FGM: A Participatory Ethnographic Evaluation Research (PEER) Study. Endline Phase 2. Options Consultancy Services Ltd; 2016.
65. Horwood J, Morden A, Bailey JE, Pathak N, Feder G. Assessing for domestic violence in sexual health environments: a qualitative study. Sex Transm Infect. 2018;94(2):88-92.
66.
66. Mohamed N, Schickler P, Warsame Z, Glew C. 'Hear Our Voices': A Report on Participatory Workshops on FGM/C (Female Genital Mutilation/Cutting) with the Somali Community in Tower Hamlets. London: Women's Health and Family Services; 2014.
67. Hearst, A.A. and Molnar, A.M., 2013, June. Female genital cutting: an evidence-based approach to clinical management for the primary care physician. In *Mayo Clinic Proceedings* (Vol. 88, No. 6, pp. 618-629). Elsevie.
68. Ajibona A, editor The understanding of the term female genital mutilation or FGM amongst patients with FGM in a United Kingdom inner city ante-natal clinic. 21st FIGO World Congress of Gynecology and Obstetrics; 2015; Vancouver, BC Canada: International Journal of Obstetrics and Gynaecology.
69. Ariyo D, Ssali R, King-Webb L, Ikpaahindi S. Voices of the Community:

Exploring Female Genital Mutilation in the African Community

across Greater Manchester. Afruca; 2015.
70. Brown K, Beecham D, Barrett H. The Applicability of Behaviour Change in Intervention Programmes Targeted at Ending Female Genital Mutilation in the EU: Integrating Social Cognitive and Community Level Approaches. Obstetrics and Gynecology International. 2013;2013(2013):324362.
71. Straus L, McEwen A, Hussein FM. Somali women's experience of childbirth in the UK: Perspectives from Somali health workers. Midwifery. 2009;25(2):181-6.
72. Glover J, Liebling H, Barrett H, Goodman S. The psychological and social impact of female genital mutilation: A holistic conceptual framework. Journal of International Studies. 2017;10(2):219-38.
73. Feder G, Wathen CN, MacMillan HL. An evidence-based response to intimate partner violence: WHO guidelines. JAMA. 2013;310(5):479-80.
74. Spangaro J, Koziol-McLain J, Zwi A, Rutherford A, Frail M-A, Ruane J. Deciding to tell: qualitative configurational analysis of decisions to disclose experience of intimate partner violence in antenatal care. Social Science & Medicine. 2016;154:45-53.
75. Dixon S, Agha K, Ali F, El Hindi L, Kelly B, Locock L, et al. Female genital mutilation in the UK- where are we, where do we go next? Involving communities in setting the research agenda. Research Involvement and Engagement. 2018;4(29).
76. Connelly E, Murray N, Baillot H, Howard N. Missing from the debate? A qualitative study exploring the role of communities within interventions to address female genital mutilation in Europe. BMJ Open. 2018;8:e021430.
77. Gordon MMAJH. Management of female genital mutilation : the Northwick Park Hospital experience - McCafrey - 1995 - BJOG: An International Journal of Obstetrics & Gynaecology - Wiley Online Library. 1995.
78. Foundations OS. Somalis in Leicester. New York, NY, USA: Open Society Foundations; 2014.
79. Fawcett RJ, Kernohan G. A retrospective analysis of 34 potentially missed cases of female genital mutilation in the emergency department. Emergenct Medicine Journal. 2017;35(10):587-9.
80. Ambuel B. Healthcare Can Change from Within: Sustained Improvement in the Healthcare Response to Intimate Partner Violence | SpringerLink. 2013.
81. Szilassy E, Drinkwater J, Hester M, Larkins C, Stanley N, Turner W, et al. Making the links between domestic violence and child safeguarding: an evidence-based pilot training for general practice. Health & Social Care in the Community. 2017;25(6):1722-32.
82. Drinkwater J, Stanley N, Szilassy E, Larkins C, Hester M, Feder G. Juggling confidentiality and safety: a qualitative study of how general practice clinicians document domestic violence in families with children. British Journal of General Practice. 2017;67(659):e437-e44.
83. Woodman J, Allister J, Rafi I, de Lusignan S, Belsey J, Petersen I, Gilbert R. A simple approach to improve recording of concerns about childmaltreatment in primary care records: developing a quality improvement intervention. British journal of general practice. 2012 Jul 1;62(600):e478-86.

84. Middleton J. Preventing violent extremism: the role of doctors. The Lancet. 2016;388(10057):2219-21.
85. Scamell M, Ghumman A. The experience of maternity care for migrant women living with female genital mutilation: A qualitative synthesis. Birth. 2019;46(1):15-23.
86. Salad J, Verdonk P, de Boer F, Abma TA. “A Somali girl is Muslim and does not have premarital sex. Is vaccination really necessary?” A qualitative study into the perceptions of Somali women in the Netherlands about the prevention of cervical cancer. International Journal for Equity in Health. 2015;14(1):68.
87. Widmark C, Levál A, Tishelman C, Ahlberg BM. Obstetric care at the intersection of science and culture: Swedish doctors' perspectives on obstetric care of women who have undergone female genital cutting. Journal of Obstetrics and Gynecology. 2010;30(6):553-8.
88. Foundations OS. Somalis in London. New York, NY, USA: Open Society Foundations; 2014. Contract No.: ISBN: 978-1-940983-07-3.
89. Harper Bulman K, McCourt C. Somali refugee women's experiences of maternity care in west London: A case study. Critical Public Health. 2010;12(4):365-80.
90. Wellock VK. Domestic abuse: Black and minority-ethnic women's perspectives. Midwifery. 2010;26(2):181-8.
91. Feder GS, Hutson M, Ramsay J, Taket AR. Women exposed to intimate partner violence: expectations and experiences when they encounter health care professionals: a meta-analysis of qualitative studies. Archives of Internal Medicine. 2006;166(1):22-37.
92. Johnsdotter S. Discrimination of Certain Ethnic Groups? Ethical Aspects of Implementing FGM Legislation in Sweden. Report. Malmo, Sweden: Faculty of Health and Society, University of Malmo; 2009 2009. Contract No.: FoU Rapport 2009:3.
93. Leye E, Powell RA, Nienhuis G, Claeys P, Temmerman M. Health care in Europe for women with genital mutilation. Health Care for Women International. 2006;27(4):362-78.
94. Paliwal P, Ali S, Bradshaw S, Hughes A, Jolly K. Management of type III female genital mutilation in Birmingham, UK: a retrospective audit. Midwifery. 2014;30(3):282-8.
95. Horwood J, Morden A, Bailey JE, Pathak N, Feder G. Assessing for domestic violence in sexual health environments: a qualitative study. Sexually Transmitted Infections. 2017;94(2):88-92.
96. Feder G, Davies RA, Baird K, Dunne D, Eldridge S, Griffiths C, et al. Identification and Referral to Improve Safety (IRIS) of women experiencing domestic violence with a primary care training and support programme: a cluster randomised controlled trial. The Lancet. 2011;378(9805):1788-95.
97. Creighton SM, Liao LM. Tackling female genital mutilation in the UK. BMJ : British Medical Journal. 2013;347:f7150.

98. Turner W, Hester M, Broad J, Szilassy E, Feder G, Drinkwater J, et al. Interventions to improve the response of professionals to children exposed to domestic violence and abuse: a systematic review. Child Abuse Review. 2017;26(1):19-39.99. Ashby J, Richardson A, Brawley D, E H, editors. National survey of practice and experience of mandatory reporting of female genital mutilation (FGM) amongst sexual health care professionals. 4th Joint Conference of the British HIV Association, BHIVA with the British Association for Sexual Health and HIV, BASHH 2018; 2018; United Kingdom: HIV Medicine.
100. Plugge E, Adam S, El Hindi L, Gitau J, Shodunke N, Mohamed-Ahmed O. The prevention of female genital mutilation in England: what can be done? Journal of public health (Oxford, England). 2018;fdy128.
101. Rymer J, editor Female genital mutilation. RCOG World Congress 2015; 2015; Brisbane, Australia: BJOG: An International Journal of Obstetrics and Gynaecology.
102. Taket A, Nurse J, Smith K, Watson J, Shakespeare J, Lavis V, et al. Routinely asking women about domestic violence in health settings. BMJ: British Medical Journal. 2003;327(7416):673-6.
103. Bewley S, Kelly B, Darke K, Erskine K, Gerada C, Lohr P, et al. Mandatory submission of patient identifiable information to third parties: FGM now, what next? BMJ : British Medical Journal. 2015;351:h5146.
104. Murray L, Windsor C, Parker E, Tewfik O. The Experiences of African Women Giving Birth in Brisbane, Australia. Health Care for Women International. 2010;31(5):458-72.
105. Naftalin J, Bewley S. Mandatory reporting of FGM. British Journal of General Practice. 2015;65(638):450-1.
106. Dixon S. The FGM enhanced dataset: how are we going to discuss this with our patients? 2015.
107. Emam KE, Mercer J, Moreau K, Grava-Gubins I, Buckeridge D, Jonker E. Physician privacy concerns when disclosing patient data for public health purposes during a pandemic influenza outbreak. BMC Public Health. 2011;11(1):454.

108. Bismark MM, Mathews B, Morris JM, Thomas LA, Studdert DM. Views on mandatory reporting of impaired health practitioners by their treating practitioners: a qualitative study from Australia. BMJ Open. 2016;6:e011988.
109. Feng J-Y, Chen S-J, Wilk NC, Yang W-P, Fetzer S. Kindergarten teachers' experience of reporting child abuse in Taiwan: Dancing on the edge. Children and Youth Services Review. 2009;31(3):405-9.
110. Gallagher A, Wainwright P, Tompsett H, Atkins C. Findings from a Delphi exercise regarding conflicts of interests, general practitioners and safeguarding children: 'Listen carefully, judge slowly'. Journal of Medical Ethics. 2012;38(2):87-92.
111. Beran R. Mandatory notification of impaired doctors. Internal Medicine Journal. 2014;44(12a):1161-5.
112. Erskine K. Collecting data on female genital mutilation. BMJ : British Medical Journal. 2014;348:g3222.
113. Foster R, Olson-Dorff D, Reiland H, Budzak-Garza A. Commitment, confidence and concerns: Assessing health care professionals' child maltreatment reporting attitudes. Child Abuse & Neglect. 2017;67:54-63.
114. McTavish JR, Kimber M, Devries K, Colombini M, MacGregor JCD, Wathen CN, et al. Mandated reporters' experiences with reporting child maltreatment: a meta-synthesis of qualitative studies. BMJ Open. 2017;7(10):e013942.
115. Johansen REB, Ziyada MM, Shell-Duncan B, Kaplan AM, Leye E. Health sector involvement in the management of female genital mutilation/cutting in 30 countries. BMC Health Services Research. 2018;18(1):240.
116. Falkiner M, Thomson D, Day A. Teachers' Understanding and Practice of Mandatory Reporting of Child Maltreatment. Children Australia. 2017;42(1):38-48.
117. Talsma M, Bengtsson Boström K, Östberg A-L. Facing suspected child abuse – what keeps Swedish general practitioners from reporting to child protective services? Scandinavian Journal of Primary Health Care. 2013;33(1):21-6.
118. Lawson D, Niven B. The Impact of Mandatory Reporting Legislation on New Zealand Secondary School Students' Attitudes towards Disclosure of Child Abuse. International Journal of Children's Rights. 2015;23:491-528.
119. Casla K, Roderick P, Pollock AM. Disclosure of patients' data to the UK Home Office must stop. BMJ : British Medical Journal. 2017;358:j3613.
120. Hiam L. Grenfell survivors shouldn't be afraid to go to hospital. BMJ : British Medical Journal. 2017;358:j3292.
121. Hiam L, Steele S, McKee M. Creating a 'hostile environment for migrants': the British government's use of health service data to restrict immigration is a very bad idea. Health Economics, Policy and Law. 2018;13(2):107-17.

122. Creighton SM, Hodes D. Female genital mutilation: what every paediatrician should know. Archives of disease in childhood. 2016 Mar 1;101(3):267-71.

123. Potter JL, Milner A. Tuberculosis: looking beyond 'migrant'as a category to understand experience. Race Equality Foundation Briefing Paper: A Better Health Briefing. 2018;44.

124. Simpson J, Robinson K, Creighton SM, Hodes D. Female genital mutilation: the role of health professionals in prevention, assessment, and management. Bmj. 2012 Mar 14;344:e1361.
