# Supplementary material for: Conversations about FGM in primary care: a realist review on how, why and under what circumstances FGM is discussed in general practice consultations
Source: BMJ Open. 2021 Mar 22;11(3):e039809. doi: 10.1136/bmjopen-2020-039809 (PMC7986780; doi:10.1136/bmjopen-2020-039809)
Supplement: Author's manuscript [file bmjopen-2020-039809.draft_revisions.pdf]

BMJ Open is committed to open peer review. As part of this commitment we make the peer review history of every article we publish publicly available.

When an article is published we post the peer reviewers' comments and the authors' responses online. We also post the versions of the paper that were used during peer review. These are the versions that the peer review comments apply to.

The versions of the paper that follow are the versions that were submitted during the peer review process. They are not the versions of record or the final published versions. They should not be cited or distributed as the published version of this manuscript.

BMJ Open is an open access journal and the full, final, typeset and author-corrected version of record of the manuscript is available on our site with no access controls, subscription charges or pay-per-view fees (http://bmjopen.bmj.com).

If you have any questions on BMJ Open's open peer review process please email info.bmjopen@bmj.com

BMJ Open

# When do GPs talk about FGM with their patients? A realist synthesis

| | |
|---|---|
| Journal: | *BMJ Open* |
| Manuscript ID | bmjopen-2020-039809 |
| Article Type: | Original research |
| Date Submitted by the Author: | 01-May-2020 |
| Complete List of Authors: | Dixon, Sharon; University of Oxford, Nuffield Department of Primary Care Health Sciences<br>Duddy, Claire; University of Oxford, Nuffield Department of Primary Care Health Sciences<br>Harrison, Gabrielle; University Hospitals Coventry and Warwickshire NHS Trust<br>Papoutsi, Chrysanthi; University of Oxford, Nuffield Department of Primary Care Health Sciences<br>Ziebland, Sue; University of Oxford, NDPCHS<br>Griffiths, Frances; University of Warwick, Warwick Medical School; University of the Witwatersrand, Centre for Health Policy |
| Keywords: | PRIMARY CARE, Health policy < HEALTH SERVICES ADMINISTRATION & MANAGEMENT, Organisation of health services < HEALTH SERVICES ADMINISTRATION & MANAGEMENT, Quality in health care < HEALTH SERVICES ADMINISTRATION & MANAGEMENT |
| | |

SCHOLARONE™
Manuscripts

*I, the Submitting Author has the right to grant and does grant on behalf of all authors of the Work (as defined in the below author licence), an exclusive licence and/or a non-exclusive licence for contributions from authors who are: i) UK Crown employees; ii) where BMJ has agreed a CC-BY licence shall apply, and/or iii) in accordance with the terms applicable for US Federal Government officers or employees acting as part of their official duties; on a worldwide, perpetual, irrevocable, royalty-free basis to BMJ Publishing Group Ltd ("BMJ") its licensees and where the relevant Journal is co-owned by BMJ to the co-owners of the Journal, to publish the Work in this journal and any other BMJ products and to exploit all rights, as set out in our licence.*

*The Submitting Author accepts and understands that any supply made under these terms is made by BMJ to the Submitting Author unless you are acting as an employee on behalf of your employer or a postgraduate student of an affiliated institution which is paying any applicable article publishing charge ("APC") for Open Access articles. Where the Submitting Author wishes to make the Work available on an Open Access basis (and intends to pay the relevant APC), the terms of reuse of such Open Access shall be governed by a Creative Commons licence – details of these licences and which Creative Commons licence will apply to this Work are set out in our licence referred to above.*

*Other than as permitted in any relevant BMJ Author's Self Archiving Policies, I confirm this Work has not been accepted for publication elsewhere, is not being considered for publication elsewhere and does not duplicate material already published. I confirm all authors consent to publication of this Work and authorise the granting of this licence.*

For peer review only

Title Page

# When do GPs talk about FGM with their patients?

A realist synthesis

Authors:

Sharon Dixon[1], Claire Duddy[1], Gabrielle Harrison[2], Chrysanthi Papoutsi[1], Sue Ziebland[1], Frances Griffiths[3,4]

1=Nuffield Department of Primary Care Health Sciences, University of Oxford

2=University Hospitals Coventry and Warwickshire, NHS trust

3=Warwick Medical School, University of Warwick.

4=Centre for Health Policy, University of the Witwatersrand, South Africa

Corresponding author:

Sharon Dixon

Nuffield Department of Primary Care Health Sciences, Radcliffe Primary Care Building, Radcliffe Observatory Quarter, Woodstock Rd, Oxford OX2 6GG

Telephone: 01865 617865

Email: Sharon.dixon@phc.ox.ac.uk

MESH terms:

FGM

Primary care

Realist synthesis

Word count:

4726

For peer review only

For peer review only

# When do GPs talk about FGM with their patients?

A realist synthesis


## Abstract

**Objectives:** Little is known about the management of FGM in primary care. There have been significant recent statutory changes relevant to GPs in England, including a mandatory reporting duty. We undertook a realist synthesis to explore what influences how and when GPs discuss FGM with their patients.

**Setting:** Primary care in England

**Data Sources**: Realist literature synthesis searching ten databases with terms: GPs, primary care, obstetrics, gynaecology, midwifery and FGM (UK and worldwide). Citation chasing was utilised, and relevant grey literature was included, including searching FGM advocacy organisation websites for relevant data. Other potentially relevant literature fields were searched for evidence to inform programme theory development. We included all study designs and papers that presented evidence about factors potentially relevant to considering how, why and in what circumstances GPs feel able to discuss FGM with their patients

**Primary outcome measure:** This realist review developed programme theory, tested against existing evidence, on what influences GPs actions and reactions to FGM in primary care consultations and, where, when and why these influences are activated.

**Results**: 122 documents were included in the synthesis. Our analysis found that GPs need knowledge and training to help them support their patients with FGM, including who may be affected, what needs they may have and , and how to talk sensitively about FGM. Access to specialist services and guidance may help them with this role. Reporting requirements may complicate these conversations.

**Conclusions:** There is a pressing need to develop (and evaluate) training to help GPs meet FGM affected communities' health needs and to promote the accessibility of primary care. Education and resources should be developed in partnership with community members. The impact of the mandatory reporting requirement and the Enhanced dataset on healthcare interactions in primary care warrants evaluation.


**Trial registration:** Prospero 2018 CRD42018091996

**Keywords:** FGM, female genital mutilation, female circumcision, GP, primary care, realist synthesis

## Strengths and limitations of this study:

- A realist approach to synthesis facilitated inclusion of wide range of data sources, and consideration of this research question despite little direct primary care research about FGM, with a comprehensive and iterative approach to data searching for relevant evidence.
- This method facilitated the inclusion of community, charity, and advocacy organisation data contributing evidence that might not have been accessible using other methods.

- We searched widely for data to inform the question in comparative fields.
- The tabulation and characterisation of the published research is itself valuable and highlights potential research gaps.

## Introduction

Female genital mutilation (FGM) is defined as all procedures that intentionally alter or cause injury to the female genitalia for non-medical reasons. There are no known health benefits, and many documented harms, including immediate and long term physical and psychological consequences. FGM is recognised internationally as an act of violence against women and girls. FGM is categorised into 4 types: type 1 (clitoridectomy), type 2 (partial or total removal of clitoris and labia minora/majora), Type 3 (infibulation) and type 4 (all other harmful procedures, including pricking and piercing)(1).

UNICEF estimate that around the world 200 million girls and women in 30 countries have been subjected to FGM. Global migration from areas where FGM is traditionally practiced means that FGM is now a worldwide health concern (2).

In 2011, it was estimated that 137,000 women and girls with FGM, from countries where FGM is traditionally practiced, were permanently resident in England and Wales. Prevalence of FGM was highest in urban areas, but women and girls affected by FGM were likely to live in every local authority area in England and Wales (3).

In 2014, the UK government hosted the first Girl Summit, in partnership with UNICEF, at which they pledged to mobilise domestic and international efforts to stamp out FGM within a generation, launching a raft of initiatives including a £ 1.4 million FGM prevention programme with NHS England, and legislative changes (4). The FGM prevention programme sought to improve how the NHS responds to FGM, including making training about FGM and safeguarding statutory for NHS organisations (5), and sets out expectations for NHS staff, including about data recording (6).

FGM has been a specific offence in the UK since 1985 (7). Before 2019, there had been no UK convictions, a fact described as a "national scandal" in 2014 by a Home Affairs Select Committee reporting on the case for an FGM National Action Plan (with aims included achieving a successful prosecution and improving safeguarding and services)(8). In 2015, a mandatory reporting duty was introduced in England and Wales, requiring all registered professionals to report all cases in under 18 year olds where FGM was identified on examination or through a first-hand disclosure directly to the police (9). Additionally, an FGM Enhanced Dataset was introduced in 2015 in England, mandating the submission of quarterly data returns including personally identifiable data from all GP practices (10). Data return rates from primary care to the Enhanced Dataset have been low, with only 64 GP practices in England submitting data returns in 2018-2019(11) . The reasons for this are not known. Concerns have been raised by clinicians and community members about the potential impacts of mandatory reporting and the Enhanced Dataset on trust and patient-doctor relationships (12-15).

In this realist synthesis we seek to understand factors that can potentially influence how GPs and women and girls from FGM affected communities interact in English primary care, in the current UK context. This was identified as an important area for exploration in a research user consultation where community members and professionals were asked what they identified as FGM research and service priorities (16). Prior systematic reviews have shown that around the world, health professionals do not have adequate knowledge about FGM, although these reviews primarily focused on obstetrics and gynaecology (17, 18).

Our exploratory review identified the relevant literature as disparate and heterogeneous. Therefore, we identified a need for bringing together and making sense of different types of evidence that would help develop our understanding of how, why, and under what circumstances FGM is discussed (or not) in GP consultations in England, in the context of recent policy changes.

To explore this over-arching review aim, we identified the following review questions:

For peer review only

1. What influences how general practitioners (GPs) manage female genital mutilation (FGM) in their clinical practice and why?
2. What influences GPs actions when they consider initiating discussion about female genital mutilation (FGM) with patients in primary care? Where, when and why are these influences active?
3. What influences how GPs respond to a patient-initiated disclosure of FGM during a primary care consultation? Where, when and why are these influences active?

## Methods

For GPs, supporting women with FGM and managing the attendant reporting, safeguarding and clinical needs associated with this can be viewed as a complex intervention (defined by the Medical Research Council (MRC) as an intervention with several interacting components, and where the behaviours required by the intervention are numerous or complex (19). The FGM Prevention Programme included the provision of new educational materials, safeguarding resources and new obligations to document and report FGM. This requires GPs to participate in educational opportunities, consider when and how to discuss FGM with their patients, and consider when and how they need to comply with reporting requirements.

Realist synthesis is a theory driven and interpretive systematic review methodology with an explanatory rather than judgemental focus which can be used to evaluate the impact of complex policy. Adopting a realist synthesis methodological approach, the research question does not only explore whether an intervention works – or not – but explicitly considers under what circumstances (when, why and how) an intervention might generate outcomes. A realist synthesis seeks to explore the contexts under which outcomes occur, and the mechanisms (processes which connect the context and the outcome) which link them (20).

There is little primary research about how English GPs are managing their patients with FGM. The realist review approach as defined by RAMESES will allow development of programme theory based on evidence about managing FGM in other health care settings (for example obstetrics and midwifery), from grey literature, including opinion pieces and charity publications, and for testing of evolving programme theory in potentially comparable healthcare challenges in English primary care.

We used the RAMESES publication standards to develop (21) and report this realist synthesis (22), and followed methodology described in other realist syntheses (23). The study protocol is available as Appendix A.

**Patient and Public involvement:** This synthesis was developed following a patient and public involvement research priority setting project, which identified this question as a research priority (16). The findings of this synthesis were reviewed with stakeholders and PPI collaborators who advised on resonance and relevance.

**Initial programme theory development**: An initial programme theory (a theory describing how and why the interventions being considered are hypothesised to operate to generate outcomes) (22) considering how and under what circumstances GPs in England might talk about FGM with their patients was derived by SD based on an exploratory literature review and relevant policy documentation (Appendix B). An expert advisory group consisting of 6 primary healthcare professionals, including local and national FGM experts, acted as project stakeholders and advised on how the programme theory fitted within primary care processes, a recognised contribution to realist synthesis (24).

**Searching:** Searching for data for inclusion in this review was conducted in multiple stages. Initial exploratory scoping searches identified an initial set of relevant documents which informed the development of the initial programme theory and contributed to theory refinement. The main searches to identify evidence to test our programme theory were developed by an information specialist and conducted in the following databases: MEDLINE, Embase, PsycINFO, Global Health, CINAHL, Web of Science, Sociological Abstracts, Anthropology Plus, Social Science Abstracts and ASSIA. We searched for literature on health professionals and FGM in the UK and worldwide, in primary care and obstetrics and gynaecology, and we searched for international qualitative studies and reviews on FGM. There was no time limit set for the search. We included other health settings where the reporting and communication requirements might be similar because of the lack of primary care data. We built our dataset iteratively by searching reference lists and conducting forward citation searches for

For peer review only

key papers. We included grey literature identified in this way and utilised recommendations from experts in FGM, including systematically searching for reports from English FGM advocacy organisations. We conducted update searches, to ensure that the most recent evidence was included. We also conducted secondary searches in the following bodies of literature, identified as areas where comparable contexts and mechanisms may occur to inform our developing programme theory: intimate partner violence (IPV) in primary care, Driving and vehicle Licensing Agency (DVLA) reporting, the Prevent programme, and mandatory reporting (in health and education). The searches are available in the online supplementary file (Appendix C).

**Selection of documents:** We included all study designs and papers that presented evidence about factors potentially relevant to considering how, why and in what circumstances GPs feel able to discuss (or not) FGM with their patients in English primary care settings, following the recent regulatory changes. Where documents from non-UK settings were identified, we excluded those written in languages other than English and from contexts likely to be significantly different from UK primary care. We included opinion pieces in influential UK medical journals as these could provide explanations relevant to UK primary care. All titles and abstracts were screened by SD. Ten percent of selected abstracts were independently reviewed by GH/CD and discrepancies discussed to inform the remaining screening process. All included full text documents were reviewed and inclusion agreed with GH/CD/study team.

**Data extraction and analysis:** Papers selected for full text review were loaded into NVivo and coded. We based initial codes on the initial programme theory, adding further emergent codes during analysis. We examined the coded data for patterns (demi-regularities (25)) from which we identified potential contexts, mechanisms and outcomes of interest. These were iteratively examined within the evidence to develop configurations of contexts, mechanisms, and outcomes (CMOC). The expert advisory group commented on the resonance of the developing and finally presented CMOCs. We prioritised high quality empirical data, however we included evidence from other sources if it was relevant to the developing primary care CMOCs (20, 26). The resulting programme theory was developed in realist format depicting the relationship between context, outcome, and the mechanism linking them. Developing theory was tested for resonance by considering it against evidence from comparative literature fields (24)

## Results:

In the searching directly related to FGM (including the scoping, main and update searches), 4035 abstracts were identified and screened, leading to full text review of 346 papers, with an additional 49 papers added for full text review from recommendation, advocacy organisation searching and citation tracking leading to the final inclusion of 92 papers from the FGM literature. Comparative literature was identified from the secondary searches from UK primary care in IPV, the Prevent strategy, DVLA reporting, and mandated reporting; these searches identified 593 citations, of which 121 were included for full text review, and 30 informed the synthesis.

Figure 1 summarises our searching and screening processes, while Tables 1 and 2 summarise the characteristics of the papers included in the synthesis

Figure 1:

For peer review only

Table 1. Characteristics/components of the 92 papers making up the FGM literature evidence which contributed to CMOC derivation are detailed below:

| **Type of study** | **Reference number** |
|---|---|
| Systematic/literature review | 17,18,29,33,43,44,45,46,47,61,63,101 |
| Qualitative | 13,27,28,30,31,32,36,48,55,57,58,59,60,62,64,65,66,67,68,70,78,81,82,83,87,93,97,106,108,109,112,113 |
| Quantitative (including survey/questionnaires) | 34,35,37,38,39,40,41,42,49,69,77,80,88,90,136 |
| Audit / case series | 72,73,74,75,79,99,115,120,134 |
| Charity reports | 14,53,54,56,85,89,91,96,107 |
| Other (e.g. editorial opinion piece) | 12,15,16,71,76,84,86,92,98,114,118,121,122,123,135 |
| **Country of research origin** | **Reference number** |
| UK | 13,14,40,41,49,53,54,55,56,62,64,66,68,69,71,73,74,75,79,80,82,83,85,86,87,89,91,93,99, 107,109,115,120,134 |
| Other European | 27,32,34,35,36,37,38,39,42,57,59,65,67,70,72,78,106,112,113,136 |
| America / Canada | 28,30,31,60,81 |
| Australia | 48,58,90,108 |
| Africa | 77 |
| **Whose perspective** | **Reference number** |
| Provider | 27,30,32,34,35,37,38,39,40,41,42,48,49,58,59,67,69,72,73,74,75,78,79,80,90,99,112,115,120,134 |
| Community | 13,28,31,53,54,55,56,57,60,62,64,65,66,68,81,82,83,85,87,88,89,91,96,106,107,108 |
| Both | 14,36,70,71,93,97,109,113 |
| **Details of providers** | **Reference number** |
| Obstetrics /gynaecology / midwifery | 27,30,32,36,37,39,40,42,48,49,58,59,69,72,79,90,109,112,115 |
| Other secondary care | 37,41,73,74,75,77,80,99,113,120,134 |
| Mixed including primary care | 14,34,35,38,71,78 |
| **Country of origin of women research based upon/conducted with/included** | **Reference numbers** |
| Somalia / infibulated women/typ3 FGM | 28,30,31,32,36,54,57,59,66,68,81,82,83,89,93,96,106,107,109,113 |
| Mixed African | 13,14,53,56,60,62,64,65,67,77,87,91,108 |
| Mixed | 73,74 (1 case in a clinic case series from Malaysia, both papers from same author/clinic) |

For peer review only

Table 2: Characteristics of the 30 papers included from literature identifies in secondary searches:

| **Intimate Partner Violence** | **Reference numbers** |
|---|---|
| Primary qualitative | 52,95,103,104,105,110,116 |
| Primary quantitative | 50,117 |
| Other/opinion/guidance/review/service evaluation | 51,94,102,111,119,124 |
| **Mandatory Reporting** | **Reference numbers** |
| Primary qualitative | 125,126,127,132 |
| Primary quantitative | 129,131,133,137 |
| Other/opinion/guidance/review | 128,130 |
| **Prevent Strategy** | **Reference numbers** |
| Primary qualitative | 140,141 |
| Primary quantitative | |
| Other/opinion/guidance/review | 100,138,139 |

## Findings:

This section is a narrative account of the final programme theory (explanatory configurations of contexts, mechanisms and outcomes) developed by this synthesis considering how, why and under what circumstances GPs might initiate (or not) or respond to FGM (or not) in the setting of English primary care. A full set of CMOCs with supporting data is available as a supplementary file (Appendix D). The programme theory is presented within four themes: FGM knowledge and awareness, communication about FGM, the role of guidelines and service provision, and mandatory reporting requirements. The over-arching realist programme theory is illustrated graphically in figures 3 and 4.

## FGM knowledge and awareness:

Health professionals need to have adequate knowledge about FGM to meet the care needs of patients who may be affected by FGM (including having had FGM or potentially being at risk of FGM) (17, 18, 27-47). Awareness of who might be affected by FGM, and the different types of FGM and their associated clinical consequences, will influences whether GPs identify a need to consider or ask about FGM with their patients. Health professionals also need to know about relevant legislation, and their statutory and safeguarding requirements in relation to FGM(17,18,34, 38,41,47,61,79)

Whether GPs are aware (or not) of what they may not know about FGM, lacking knowledge about FGM affects practitioners' ability and confidence when caring for women with FGM (36, 48, 49), including confidence to consider who may be at risk (50). Knowing how to respond to a disclosure or when identifying that a woman has FGM may help GPs feel confident to ask (51, 52). In turn, women who perceive that GPs do not have the

For peer review only

knowledge or skills to recognise their FGM related care needs, or who feel potentially stigmatised because of their FGM, may lack confidence in accessing healthcare (31, 36, 48, 53-57).

Health professionals report experiencing strong emotional reactions to encountering FGM, including anger, shock and pity (58-61), and that seeing FGM without having adequate knowledge can be "frightening" (59). Experiencing these strong emotional responses may contribute to clinicians feeling panicked, and abandoning usual practices and routines (52, 61). Although professionals describe trying to hide their reactions, they were aware that they may be apparent to the women (47). This observation is mirrored by evidence from community members who describe feeling ashamed or judged when health care professionals react with shock or horror to their FGM, notably during physical examinations (53,62,63,64,67). This can impact on their willingness to access services (46, 62-65), including attending for cervical smears (66). This could be mitigated against when professionals were able to act with confidence and sensitivity (52, 67, 68).

Health professionals with experience of supporting patients with FGM were likely to have more knowledge about FGM (34, 38, 40, 69). A potential challenge for GPs is that FGM may form only a small part of their workload (70, 71), meaning that learning about FGM may not be identified as a priority. Another potential difficulty is that FGM can be hard to identify (72). Recent data from a specialist paediatric clinic in England noted that the examination signs in type 4 can be subtle, and potentially difficult to identify (73-75) and others have noted that not all GPs will have the necessary expertise to identify all types of FGM (12, 76). Added to this complexity is that self-reporting by women about their FGM type in health settings has been shown not to be reliable (77), which could impact on how women feel able to respond to some questions about FGM.

### Talking about FGM and communication:

A key skill GPs need is being able to talk about FGM sensitively (36, 45, 78). GPs who do not feel confident in raising the subject of FGM, for example because they are worried that they will upset or offend women, may avoid talking about FGM (14, 30, 31, 42, 46, 47, 53, 59, 72, 79, 80). Health professionals who perceived that discussing FGM can be culturally taboo or a sensitive subject could also be fearful of offending women reported sometimes avoiding talking about FGM (14, 47, 53, 78, 81, 82), described in one study as an "expression of respect"(78). This contextual factor may be evolving as community attitudes towards talking about FGM are shifting, meaning that talking about FGM is becoming less taboo in some communities (61, 83-87). Lacking awareness of shifting community attitudes can risk offending or alienating members of those communities (61, 84) which risks reducing effective communication.

The words or terminology that GPs use when they talk about FGM can complicate communication with their patients. For example, if the term FGM is not familiar to the woman (88), offends or alarms her (89, 90), or she does not align her cultural practice (e.g. labial elongation) with FGM (91), then she may not relate her experience to FGM. This can complicate conversations about the potential health consequences of FGM or whether women perceive the conversation is relevant to their experience of FGM (92).

Women who were aware of previous difficult experiences with communication and engagement with health professionals (e.g. language, cultural differences, perceived judgement) where they had not felt understood or respected describe a lack of confidence and trust in health services (45, 53-55, 62, 90, 93). Women who feel pitied or judged may be less likely to feel able to make a disclosure to healthcare professionals (94, 95). Language barriers or a lack of understanding about how health services work, including negotiating with primary care reception staff can complicate access to healthcare (96).

A potential strategy that could help facilitate both the acceptability and accessibility of services is the involvement of community health advocates, such as members of FGM affected communities, who can act as a bridge between communities and services, for example by promoting trust and providing education for both community members and health professionals (16, 97, 98).

For GPs, whether FGM is raised by the patient or the GP, an important contextual factor is whether FGM is perceived to be relevant to the health concern which the woman brings to her GP appointment. This can influence both whether the subject of FGM is broached, and then if broached, how it is received and experienced (14, 60, 99). Women who feel that the healthcare professional is pre-occupied with their FGM,

For peer review only

rather than their health concerns may disengage from the healthcare setting (83, 100, 101). This is also potentially relevant when GPs consider asking women about their experience of FGM with the aim of considering the safeguarding needs within their families, rather than the woman's own health needs (61, 102, 103). Balancing the needs of the woman who is presenting with the potential needs of her wider family may introduce complex considerations for GPs when they are considering how and into whose medical notes they code FGM into primary care medical records (103-105).

An important context which influences how able (and enabled) GPs and their patients are to effectively communicate about FGM is whether there is a language barrier between them, or not (14, 18, 30, 45, 47, 53, 80, 81, 96, 106-108) . Strategies to address language barriers add their own complications. Official interpreters are recommended, but may not be available or trusted by women, for example if they both perceive FGM as taboo, or she fears they will not respect her confidentiality. This can lead to fear and reduced engagement with health professionals (18, 30, 32, 47, 60, 66, 79, 109, 110). The presence of family members (as interpreters, or witnesses) in the consultation may inhibit GPs from feeling able to raise FGM with the women, because of concerns about privacy and confidentiality (14, 30, 111).

Finally factors such as the GPs gender may influence whether the woman or GP feel it is culturally appropriate to talk about FGM (14, 47, 66, 106). Time constraints in the consultation may act as a potential barrier which deters GPs when they are contemplating discussing FGM (14, 51, 71, 112).

**The need for guidelines and access to specialist services:**

Researchers and commentators suggest that having access to clear guidelines will enable professionals to ask women about FGM and optimise their care (17, 44, 59, 113, 114). However even when guidelines exist, awareness of them may be incomplete, or they may not be followed, as demonstrated by four UK hospital studies (69, 79, 80, 115). Access to guidance may be especially important for clinicians who see FGM less often (47). Lacking guidance, including a lack of certainty of what "good care" comprises can lead clinicians to feel unsure, and improvise how they offer care (46, 47), and risks incorrect decision making (113, 114).

Normalising asking about FGM, for example by using prompts in the medical record may overcome some clinician barriers and help them begin these discussions (116), especially if these are then linked to resources or care pathways (117).

Having access to services which could offer specialist assessments, treatment or advice may help GPs feel enabled to raise the subject of FGM (12, 53, 55, 76, 118). When health professionals speak about FGM within a framework of offering support and services, it is more likely to be experienced as acceptable by their patients (87). When training and education are supported by referral pathways or protocols for intervention, they are more likely to be effective in changing behaviour and promoting clinicians asking (52, 94, 117, 119).

**Mandated actions including The Mandatory Reporting Duty and The FGM Enhanced Dataset requirements:**

It is unknown what impact the FGM reporting duties have on healthcare interactions, but concerns have been raised that the Mandatory Reporting Duty in FGM may cause women distress (120), or reduce their trust in healthcare professionals, which may deter women from seeking healthcare or disclosing their FGM to healthcare professionals (12-14, 83, 121). Furthermore, if women perceive that the healthcare professional is more interested in data management about their FGM than their needs, they may feel disrespected and avoid healthcare settings (83).

The requirement to send personally identifiable data to the FGM Enhanced Dataset was also identified as a potential barrier to talking about FGM, by both women and GPs (14-16, 122, 123). When women are not confident that their medical encounters or records are confidential, they may feel fearful and avoid making disclosures (111, 124).

For peer review only - http://bmjopen.bmj.com/site/about/guidelines.xhtml

The concern that making a mandated report would have a negative impact on on-going effective doctor-patient relationships may be an important consideration for professionals (125-129), including whether this might deter patients from  accessing services (130). Lacking confidence that making a mandated report will be met with an acceptable or adequate response may pose challenges for healthcare professionals (128, 131).

Practitioners may need to feel certain before making a mandated report (132, 133), and challenges in identifying less apparent forms of FGM may add tensions to the requirements for mandated reporting (128). In the case of FGM, this could be complicated if GPs do not feel confident that they have the knowledge or skills to correctly identify FGM (71, 90), especially type 1 and 4 which may be harder to visualise on examination and more commonly encountered (73, 134). Reporting without training may lead to inaccurate data collection (135). Clinician concerns about confidentiality and stigma could also contribute to incomplete or inaccurate data coding (136).

When young people know that the professional whom they are speaking to is mandated to share the information with other authorities, they may feel more reluctant to trust the professional, and less likely to make a disclosure (137). Those who are potentially fearful of authorities or perceive themselves to be vulnerable, for example those with uncertain migrant status, may be more fearful of mandatory reporting or data sharing and avoid accessing services (138-141).

Figure 2: Conceptual depiction of factors that may influence when GPs may or may not talk about FGM with their patients

Figure 3: Conceptual diagram illustrating the over-arching programme theory for this synthesis:

**Discussion:**

**Summary:**

GPs need adequate knowledge to support their patients with FGM including the different FGM types and their different clinical presentations, needs, and cultural contexts. This includes needing to be aware of local legislative, statutory, and safeguarding requirements. GPs need skills to discuss FGM sensitively and with appropriate terminology. Language barriers can complicate conversations about FGM. Access to official interpreters is recommended but they may not always be available. Even when available, there are potential pitfalls which GPs should be aware of, including consideration of who else is present in the consultation. The requirements of mandatory reporting and the FGM Enhanced Dataset may bring additional complications into the primary care consultation. Community health advocates could have a role in facilitating access to services.

**Strengths and Limitations:**

As illustrated by table 1, only limited evidence was directly relevant to primary care. GPs have a vital role in managing FGM, yet there is little evidence about their attitudes, knowledge or behaviour towards managing FGM in primary care, and none in the context of the 2015 policy changes. This synthesis therefore utilises evidence from provider experiences in other healthcare settings, predominantly specialist clinics, and obstetrics and gynaecology services. Some challenges are likely to be comparable between these settings and primary care, namely needing adequate knowledge and managing challenges with language and communication. However, there are differences between primary and secondary care that may limit this extrapolation, for example that in obstetrics, FGM will almost always be relevant to the woman's reason for attendance, which is not the case in general practice. To address this lack of direct evidence we have also undertaken a primary qualitative study with GPs (Dixon et al. BJGP, in press). The lack of primary data about GPs necessitated complex searches and we may have inadvertently not identified important evidence.

We identified limited evidence on the experiences and needs of women from outside of Africa and with forms of FGM other than type 3. Given the evidence that these types may not be those that GPs most commonly

For peer review only

encounter, this is a potentially significant limitation. Whether these findings can be extrapolated to inform healthcare for women with different FGM types or from other FGM practicing countries (for example Malaysia or Indonesia) is unclear.

The GP consultation represents a coming together of GP and patient, and a strength of this synthesis is that it explores whether GPs talk about FGM using experiential evidence from both perspectives. Realist methodology allowed us to generate explanatory programme theory relevant to GPs from evidence on managing FGM in other healthcare settings and within other healthcare contexts, despite the lack of primary care data. Realist methodology supports the inclusion of grey literature. This project has benefitted from information on English community and GP perspectives reported by FGM advocacy organisations, which may not have been included in a traditional systematic review. Using iterative searching and citation tracking maximised data inclusion.

## Comparison with existing literature:

The FGM literature is predominantly descriptive. This helps define potential challenges but offers less evidence about effective interventions with which to address them.

We have identified deficiencies in professional knowledge as an important contextual factor that can influence whether GPs talk about FGM. One study with eleven midwives evaluated the effectiveness of an educational intervention and found that it was successful in promoting knowledge and confidence in managing FGM (142). This intervention used case studies, which have been suggested by community advocates for FGM education (14, 53). A 2016 survey of medical students from five medical schools in London reported that the majority of respondents had not received formal teaching on FGM and were not aware of potential associated health issues. Having had formal education increased awareness, but despite this, only 50% of respondents who had been formally educated felt confident about identifying FGM on examination (143). UK medical students have voted to ask for education about FGM. Medical students reported that after attending a workshop which included education on FGM, the UK law, and how to talk about FGM, that 75% of them felt more confident about communicating with a patient who had had FGM (144). There is a call for FGM education to promote professionals' cultural competency (18, 29, 33, 145). Cultural competency education for healthcare professionals has benefits (146), including for patients (147), although formal cultural competency training is often lacking in general practice (148). We have not identified any literature evaluating the impact of FGM education for primary care practitioners on their clinical confidence or cultural competencies.

That it can be important for health professionals to manage their own emotional reactions when they are supporting patients affected by FGM is resonant with research into intimate partner violence, which tells us that clinician responses including blaming, judging or pitying should be avoided (94).

In tabulating the available evidence relevant to our synthesis question, we note that the available FGM evidence is predominantly from obstetric settings, with a lack of evidence from other settings, notably primary care. The holistic life-course health needs for women with FGM, including their FGM related needs outside of safeguarding, paediatric, or obstetric settings, are important service and research needs (16, 149). We also note that much of the identified research considers the needs and experience of women with type 3 FGM, yet an English specialist paediatric clinic most commonly identified type 4 FGM and no cases of infibulation and identified a girl from Malaysia with FGM (73). It is important that clinicians are aware of the practice of FGM in some Asian countries (including Indonesia and Malaysia) although there is little evidence about prevalence to guide them (73).

## Implications for research and practice:

This research will help GPs (and GP educators) consider what knowledge or skills are needed to support GPs to feel confident to talk about FGM with their patients. It may help them consider the challenges when using interpreters to talk about FGM, and potential challenges of managing the FGM reporting requirements.

Research is needed to explore what FGM-affected communities need from GPs. This needs to include all types of FGM, and all communities that practice FGM. The potential impacts of the mandatory reporting and FGM

Enhanced dataset requirements on healthcare interactions need to be evaluated, including GP and community perspectives. Educational interventions, research, and services should be developed in partnership with community members, utilising their expertise and experience, to ensure resources meet their needs.

Funding Declaration: Sharon Dixon was funded by a National Institute for Health Research (NIHR) In-Practice fellowship for this research project. The views expressed are those of the author(s) and not necessarily those of the NIHR or the Department of Health and Social Care.

Competing Interests Statement: Sharon Dixon is a trustee of Oxford Against Cutting. Following this project, she is the FGM lead for the RCGP. Sharon Dixon has held small grants to develop and report on the patient and public involvement project that under-pinned this research.

There are no other author competing interests to declare.

Author Contributions:

Sharon Dixon and Frances Griffiths wrote and developed the study protocol, the initial programme theory,  and conducted the data analysis and synthesis.

Chrysanthi Papoutsi provided methodological guidance and advice.

Claire Duddy and Gabrielle Harrison read and agreed on abstracts and data for final inclusion.

Claire Duddy assisted with study searching and data management.

All authors contributed to the development and writing of this manuscript

For peer review only

References

1. WHO. Female genital mutilation Fact Sheet. World Health Organization; 2017 2017-02-07 11:25:18.
2. UNICEF. Female genital mutilation/cutting: A global concern - UNICEF DATA. New York: United Nations Children's Fund; 2016 2016-02-03.
3. Macfarlane A, Dorkenoo E. Prevalence of Female Genital Mutilation

in England and Wales:

National and local estimates. 2015.
4. Girl Summit 2014 - GOV.UK [press release]. 2019.
5. England N. NHS England » Female genital mutilation – care and prevention 2019 [Available from: https://www.england.nhs.uk/ourwork/safeguarding/our-work/fgm/.
6. England N. NHS England » Female Genital Mutilation Prevention Programme: Requirements for NHS staff 2017 [Available from: https://www.england.nhs.uk/2014/12/fgm-prevention/.
7. Female Genital Mutilation Prosecution Guidance | The Crown Prosecution Service: s of information; 2019 [Available from: https://www.cps.gov.uk/legal-guidance/female-genital-mutilation-prosecution-guidance.
8. Home Affairs Committee HoC. Female genital mutilation: the case for a national action plan Second Report of Session 2014–15. London: the House of Commons; 2014.
9. Government HM. Mandatory reporting of female genital mutilation: procedural information. In: Office TH, editor. London: HMSO; 2015.
10. Department of Health H. FGM Prevention Programme. Understanding the FGM Enhanced dataset – updated guidance and clarification to support implementation. In: Health Do, editor. London: The Stationary Office; 2015.
11. Female Genital Mutilation (FGM) Enhanced Dataset

April 2018 to March 2019, England, experimental statistics, Annual Report NHS Digital: HSCIC; 2019 [27.9.2019]. Available from: https://digital.nhs.uk/data-and-information/publications/statistical/female-genital-mutilation/april-2018---march-2019.
12. Mathers N, Rymer J. Mandatory reporting of female genital mutilation by healthcare professionals. British Journal of General Practice. 2015;65(635):282-3.
13. Plugge E, Adam S, El Hindi L, Gitau J, Shodunke N, Mohamed-Ahmed O. The prevention of female genital mutilation in England: what can be done? Journal of public health (Oxford, England). 2018;fdy128.
14. Clayton-Hathaway K. A pilot evaluation of health services for communities affected by FGM/C in Oxfordshire. Oxfordshire: Oxford Against Cutting

Healthwatch Oxfordshire; 2016.
15. Bewley S, Kelly B, Darke K, Erskine K, Gerada C, Lohr P, et al. Mandatory submission of patient identifiable information to third parties: FGM now, what next? BMJ : British Medical Journal. 2015;351:h5146.
16. Dixon S, Agha K, Ali F, El Hindi L, Kelly B, Locock L, et al. Female genital mutilation in the UK- where are we, where do we go next? Involving communities in setting the research agenda. Research Involvement and Engagement. 2018;4(29).
17. Zurynski Y, Sureshkumar P, Phu A, Elliott E. Female genital mutilation and cutting: a systematic literature review of health professionals' knowledge, attitudes and clinical practice. BMC international health and human rights. 2015;15:32.
18. Dawson A, Homer CS, Turkmani S, Black K, Varol N. A systematic review of doctors' experiences and needs to support the care of women with female genital mutilation. International Journal of Gynecology & Obstetrics. 2015;131(1):35-40.

19. Craig P, Dieppe P, Macintyre S, Michie S, Nazareth I, Petticrew M. Developing and evaluating complex interventions: the new Medical Research Council guidance. 2008.
20. Pawson R, Greenhalgh T, Harvey G, Walshe K. Realist review--a new method of systematic review designed for complex policy interventions. Journal of health services research & policy. 2005;10 Suppl 1:21-34.
21. Wong G, Greenhalgh T, Westhorp G, Pawson R. Development of methodological guidance, publication standards and training materials for realist and meta-narrative reviews: the RAMESES (Realist And Meta-narrative Evidence Syntheses – Evolving Standards) project. 2014.
22. Wong G, Greenhalgh T, Westhorp G, Buckingham J, Pawson R. RAMESES publication standards: realist syntheses. BMC medicine. 2013;11:21.
23. Papoutsi C, Nuffield Department of Primary Care Health Sciences UoO, Radcliffe Observatory Quarter, Woodstock Road, Oxford OX2 6GG, UK, Mattick K, Centre for Research in Professional Learning UoE, St Luke's Campus, Exeter EX1 2LU, UK, Pearson M, National Institute for Health Research (NIHR) Collaboration for Leadership in Applied Health Research and Care South West Peninsula IoHR, University of Exeter Medical School, South Cloisters, St Luke's Campus, Exeter EX1 2LU, UK, et al. Social and professional influences on antimicrobial prescribing for doctors-in-training: a realist review. Journal of Antimicrobial Chemotherapy. 2017;72(9):2418-30.
24. Pawson R, Greenhalgh T, Harvey G, Walshe K. Realist synthesis: an introduction. University of Manchester: ESRC Research Methods Programme; 2004.
25. Jagosh J, Pluye P, Wong G, argo M, Salsberg J, Bush PL, et al. Critical reflections on realist review: insights from customizing the methodology to the needs of participatory research assessment. *Research synthesis methods*. 2014;*5* (2):pp.131-41.
26. Pawson R. Digging for Nuggets: How 'Bad' Research Can Yield 'Good' Evidence. http://dxdoiorg/101080/13645570600595314. 2007.
27. Leval A, Widmark C, Tishelman C, Ahlberg BM. THE ENCOUNTERS THAT RUPTURE THE MYTH: CONTRADICTIONS IN MIDWIVES' DESCRIPTIONS AND EXPLANATIONS OF CIRCUMCISED WOMEN IMMIGRANTS' SEXUALITY. Health Care for Women International. 2010;25(8):743-60.
28. Khaja K, Lay K, Boys S. Female Circumcision: Toward an Inclusive Practice of Care. Health Care for Women International. 2010;31(8):686-99.
29. Odemerho BI, Baier M. Female Genital Cutting and the Need for Culturally Competent Communication. The Journal for Nurse Practitioners. 2012;8(6):452-7.
30. Lazar JN, Johnson-Agbakwu CE, Davis OI, Shipp MPL. Providers' Perceptions of Challenges in Obstetrical Care for Somali Women. Obstetrics and Gynecology International. 2013;2013:149640.
31. Johnson-Agbakwu CE, Helm T, Killawi A, Padela AI. Perceptions of obstetrical interventions and female genital cutting: insights of men in a Somali refugee community. Ethnicity and Health. 2014;19(4):440-57.
32. Byrskog U, Olsson P, Essén B, Allvin M-K. Being a bridge: Swedish antenatal care midwives' encounters with Somali-born women and questions of violence; a qualitative study. BMC Pregnancy and Childbirth. 2015;15(1).
33. Varol N, Hall JJ, Black K, Turkmani S, Dawson A. Evidence-based policy responses to strengthen health, community and legislative systems that care for women in Australia with female genital mutilation / cutting. Reproductive Health. 2017;14(1):63.
34. Tantet C, Aupiais C, Bourdon M, Sorge F, Pagès A, Levy D, et al. Female genital mutilation: an evaluation of the knowledge of French general and specialized travel medicine practitioners. Journal of Travel Medicine. 2018;25(1):tax090.
35. González-Timoneda A, Ros VR, González-Timoneda M, Sánchez AC. Knowledge, attitudes and practices of primary healthcare professionals to female genital mutilation in Valencia, Spain: are we ready for this challenge? BMC health services research. 2018;18(1):579.
36. Vangen S, Johansen REB, Sundby J, Træen B, Stray-Pedersen B. Qualitative study of perinatal care experiences among Somali women and local health care professionals in Norway. European Journal of Obstetrics & Gynecology and Reproductive Biology. 2004;112(1):29-35.

For peer review only

37. Tamaddon L, Johnsdotter S, Liljestrand J, Essén B. Swedish Health Care Providers' Experience and Knowledge of Female Genital Cutting. Health Care for Women International. 2006;27(8):709-22.
38. Kaplan-Marcusan A, Torán-Monserrat P, Moreno-Navarro J, Fàbregas MJC, Muñoz-Ortiz L. Perception of primary health professionals about Female Genital Mutilation: from healthcare to intercultural competence. BMC Health Services Research. 2009;9(11).
39. Leye E, Ysebaert I, Deblonde J, Claeys P, Vermeulen G, Jacquemyn Y, et al. Female genital mutilation: Knowledge, attitudes and practices of Flemish gynaecologists. The European Journal of Contraception and Reproductive Health Care. 2009;13(2):182-90.
40. Zaidi N, Khalil A, Roberts C, Browne M. Knowledge of female genital mutilation among healthcare professionals. Journal of Obstetrics and Gynecology. 2009;27(2):161-4.
41. Relph S, Inamdar R, Singh H, Yoong W. Female genital mutilation/cutting: knowledge, attitude and training of health professionals in inner city London. European Journal of Obstetrics & Gynecology and Reproductive Biology. 2013;168(2):195-8.
42. Cappon S, L'Ecluse C, Clays E, Tency I, Leye E. Female genital mutilation: Knowledge, attitude and practices of Flemish midwives. Midwifery. 2015;31(3):e29-e35.
43. Dawson A, Turkmani S, Fray S, Nanayakkara S, Varol N, Homer C. Evidence to inform education, training and supportive work environments for midwives involved in the care of women with female genital mutilation: A review of global experience. Midwifery. 2015;31(1):229-38.
44. Reig-Alcaraz M, Siles-González J, Solano-Ruiz C. A mixed-method synthesis of knowledge, experiences and attitudes of health professionals to Female Genital Mutilation. Journal of Advanced Nursing. 2015;72(2):245-60.
45. Smith H, Stein K. Health information interventions for female genital mutilation. International Journal of Gynecology & Obstetrics. 2017;136(S1):79-82.
46. Jordal M, Wahlberg A. Challenges in providing quality care for women with female genital cutting in Sweden–A literature review. Sexual & Reproductive Healthcare. 2018;17:91-6.
47. Evans C, Tweheyo R, McGarry J, Eldridge J, Albert J, Nkoyo V, et al. Crossing cultural divides: A qualitative systematic review of factors influencing the provision of healthcare related to female genital mutilation from the perspective of health professionals. PloS one. 2019;14(3):e0211829.
48. Dawson AJ, Turkmani S, Varol N, Nanayakkara S, Sullivan E, Homer CS. Midwives' experiences of caring for women with female genital mutilation: Insights and ways forward for practice in Australia. Women and Birth. 2015;28(3):207-14.
49. Nash E, Ranka P, editors. FEMALE GENITAL MUTILATION: KNOWLEDGE, CONFIDENCE, AND APPROACH TO CARE IN CLINICAL PRACTICE OF MIDWIVES AND NURSES IN THE UK. 21st FIGO World Congress of Gynecology and Obstetrics; 2015; Vancouver, BC Canada: International Journal of Gynecology and Obstetrics.
50. Ramsay J, Rutterford C, Gregory A, Dunne D, Eldridge S, Sharp D, et al. Domestic violence: knowledge, attitudes, and clinical practice of selected UK primary healthcare clinicians. British Journal of General Practice. 2012;62(602):e647-e55.
51. Hegarty K, Taft A, Feder G. Violence between intimate partners: working with the whole family. BMJ : British Medical Journal. 2008;337:a839.
52. Sundborg E, Törnkvist L, Saleh-Stattin N, Wändell P, Hylander I. To ask, or not to ask: the hesitation process described by district nurses encountering women exposed to intimate partner violence. Journal of Clinical Nursing. 2017;26(15-16):2256-65.
53. Norman K, Hemmings J, Hussein E, Otoo-Oyortey N. FGM is always with us: Experiences, Perceptions and Beliefs of Women Affected by Female Genital Mutilation in London. Results from a PEER study London: Options Consultancy Services Ltd FORWARD; 2009.

54. Hussein E. Women's Experiences, Perceptions and Attitudes of Female Genital Mutilation: The Bristol PEER study FORWARD; 2010.
55. Glover J, Liebling H, Barrett H, Goodman S. The psychological and social impact of female genital mutilation: A holistic conceptual framework. Journal of International Studies. 2017;10(2):219-38.

For peer review only

56. Norman K, Belay Gegzabher S, Otoo-Oyortey N. “Between Two Cultures”: A Rapid PEER Study Exploring Migrant Communities’ Views on Female Genital Mutilation in Essex and Norfolk, UK. London: National FGM Centre

FORWARD; 2016.
57. Mbanya VN, Gele AA, Diaz E, Kumar B. Health care-seeking patterns for female genital mutilation/cutting among young Somalis in Norway. BMC Public Health. 2018;18(1):517.
58. Ogunsiji O. Female Genital Mutilation (FGM): Australian Midwives’ Knowledge and Attitudes. Health Care for Women International. 2015;36(11):1179-93.
59. Widmark C, Tishelman C, Ahlberg BM. A study of Swedish midwives' encounters with infibulated African women in Sweden. Midwifery. 2002;18(2):113-25.
60. Horowitz CR, Jackson JC. Female “circumcision” African women confront American medicine. Journal of General Internal Medicine. 1997;12(8):491-9.
61. Johnsdotter S, Essén B. Cultural change after migration: Circumcision of girls in Western migrant communities. Best Practice & Research Clinical Obstetrics & Gynaecology. 2016;32:15-25.
62. Oguntoye S, Otoo-Oyortey N, Hemmings J, Norman K, Hussein E. “FGM is with us Everyday”: Women and Girls Speak out about Female Genital Mutilation in the UK. World Academy of Science, Engineering and Technology. 2009;54:1020-5.
63. Hamid A, Grace KT, Warren N. A Meta-Synthesis of the Birth Experiences of African Immigrant Women Affected by Female Genital Cutting. Journal of Midwifery & Women's Health. 2018;63(2):185-95.
64. Recchia N, McGarry J. “Don’t judge me”: narratives of living with FGM. International Journal of Human Rights in Healthcare. 2017;10(1):4-13.
65. del Mar Pastor-Bravo M, Almansa-Martínez P, Jiménez-Ruiz I. Living with mutilation: A qualitative study on the consequences of female genital mutilation in women's health and the healthcare system in Spain. Midwifery. 2018;66:119-26.
66. Abdullahi A, Copping J, Kessel A, Luck M, Bonell C. Cervical screening: Perceptions and barriers to uptake among Somali women in Camden. Public Health. 2009;123(10):680-5.
67. Vloeberghs E, van der Kwaak A, Knipscheer J, van den Muijsenbergh M. Coping and chronic psychosocial consequences of female genital mutilation in the Netherlands. Ethnicity and Health. 2013;17(6):677-95.
68. Moxey JM, Jones LL. A qualitative study exploring how Somali women exposed to female genital mutilation experience and perceive antenatal and intrapartum care in England. BMJ Open. 2016;6(1):e0009846.
69. Purchase TC, Lamoudi M, Colman S, Allen S, Latthe P, Jolly K. A survey on knowledge of female genital mutilation guidelines. Acta Obstetricia et Gynecologica Scandinavica. 2013;92(7):858-61.
70. Baillot H, Murray N, Connelly E, Howard N. Addressing female genital mutilation in Europe: a scoping review of approaches to participation, prevention, protection, and provision of services. International Journal for Equity in Health. 2018;17(1):21.
71. Baillot H, Murray N, Connelly E, Howard N. Tackling Female Genital Mutilation in Scotland: A Scottish Model of Intervention. Scotland: Scottish Refugee Council

London School of Hygiene and Tropical Medicine 2014.
72. Abdulcadir J, Dugerdil A, Boulvain M, Yaron M, Margairaz C, Irion O, et al. Missed opportunities for diagnosis of female genital mutilation. International Journal of Gynecology & Obstetrics. 2014;125(3):256-60.
73. Hodes D, Armitage A, Robinson K, Creighton SM. Female genital mutilation in children presenting to a London safeguarding clinic: a case series. Archives of Disease in Childhood. 2016;101(3):212-6.
74. Hodes D, Armitage A, Dykes A, editors. Female genital mutilation in London and the UNICEF report; a local perspective on worldwide statistics. Annual Conference of the Royal College of

Paediatrics and Child Health, RCPCH 2014; 2014 2014-04-01; Birmingham, UK: Archives of Disease in Childhood.
75. Ayadi O'Donnell N, Pall K, Leoni M, Debelle G, Lynn R, Armitage A, et al., editors. Female genital mutilation (FGM) surveillance in under 16 years olds in the UK and Ireland. Royal College of Paediatrics and Child Health Annual Conference, RCPCH 2018; 2018 2018-03-01; United Kingdom: Archives of Disease in Childhood.
76. Female Genital Mutilation: A clinical approach for GPs. Royal College of General Practitioners; 2017. Contract No.: 5.3.2017.
77. Elmusharaf S, Elhadi N, Almroth L. Reliability of self reported form of female genital mutilation and WHO classification: cross sectional study. BMJ. 2006;333(7559):124.
78. Johansen REB. Care for Infibulated Women Giving Birth in Norway: An Anthropological Analysis of Health Workers' Management of a Medically and Culturally Unfamiliar Issue. Medical Anthropology Quarterly. 2006;20(4):516-44.
79. Zenner N, Liao LM, Richens Y, Creighton SM. Quality of obstetric and midwifery care for pregnant women who have undergone female genital mutilation. Journal of Obstetrics and Gynecology. 2013;33(5):459-62.
80. Gabrasadig R, Asamoah F, Wilson N, editors. Female genital mutilation: knowledge, training and experience of healthcare professionals at a London hospital. Annual Conference of the Royal College of Paediatrics and Child Health, RCPCH 2015; 2015 2015-04-01; Birmingham, UK: Archives of Disease in Childhood.
81. Upvall MJ, Mohammed K, Dodge PD. Perspectives of Somali Bantu refugee women living with circumcision in the United States: A focus group approach. International Journal of Nursing Studies. 2009;46(3):360-8.
82. Safari F. A qualitative study of women's lived experience after deinfibulation in the UK. Midwifery. 2013;29(2):154-8.
83. Karlsen S, Carver N, Mogilnicka M, Pantazis C. When safeguarding becomes stigmatising: A report on the impact of FGM-safeguarding procedures on people with a Somali heritage living in Bristol. Bristol: University of Bristol; 2019.
84. Creighton SM, Samuel Z, Otoo-Oyortey N, Hodes D. Tackling female genital mutilation in the UK. BMJ. 2019;364:l15.
85. FORWARD. "A Big Wake-Up Call": Participatory Study on Shifts in Attitudes Towards FGM Amongst Community Women in Bristol, Summary Report. FORWARD

Refugee Women of Bristol; 2017.
86. Brown E, Porter C. The Tackling FGM Initiative: Evaluation of the Second Phase

(2013-2016). London: Options Consultancy Services Ltd; 2016.
87. Brown E, Porter C, Unit OP. Evaluation of FGM Prevention among Communities Affected by FGM: A Participatory Ethnographic Evaluation Research (PEER) Study. Endline Phase 2. Options Consultancy Services Ltd; 2016.
88. Ajibona A, editor The understanding of the term female genital mutilation or FGM amongst patients with FGM in a United Kingdom inner city ante-natal clinic. 21st FIGO World Congress of Gynecology and Obstetrics; 2015; Vancouver, BC Canada: International Journal of Obstetrics and Gynaecology.
89. Mohamed N, Schickler P, Warsame Z, Glew C. 'Hear Our Voices': A Report on Participatory Workshops on FGM/C (Female Genital Mutilation/Cutting) with the Somali Community in Tower Hamlets. London: Women's Health and Family Services; 2014.
90. Turkmani S, Homer C, Varol N, Dawson A. A survey of Australian midwives' knowledge, experience, and training needs in relation to female genital mutilation. Women and Birth. 2018;31(1):25-30.
91. Ariyo D, Ssali R, King-Webb L, Ikpaahindi S. Voices of the Community: Exploring Female Genital Mutilation in the African Community across Greater Manchester. Afruca; 2015.

For peer review only

92. Brown K, Beecham D, Barrett H. The Applicability of Behaviour Change in Intervention Programmes Targeted at Ending Female Genital Mutilation in the EU: Integrating Social Cognitive and Community Level Approaches. Obstetrics and Gynecology International. 2013;2013(2013):324362.
93. Straus L, McEwen A, Hussein FM. Somali women's experience of childbirth in the UK: Perspectives from Somali health workers. Midwifery. 2009;25(2):181-6.
94. Feder G, Wathen CN, MacMillan HL. An evidence-based response to intimate partner violence: WHO guidelines. JAMA. 2013;310(5):479-80.
95. Spangaro J, Koziol-McLain J, Zwi A, Rutherford A, Frail M-A, Ruane J. Deciding to tell: qualitative configurational analysis of decisions to disclose experience of intimate partner violence in antenatal care. Social Science & Medicine. 2016;154:45-53.
96. Foundations OS. Somalis in Leicester. New York, NY, USA: Open Society Foundations; 2014.
97. Connelly E, Murray N, Baillot H, Howard N. Missing from the debate? A qualitative study exploring the role of communities within interventions to address female genital mutilation in Europe. BMJ Open. 2018;8:e021430.
98. McCafrey M, Jankowska A, Gordon H. Management of female genital mutilation : the Northwick Park Hospital experience. BJOG: An International Journal of Obstetrics & Gynaecology. 1995;102(10):787-90.
99. Fawcett RJ, Kernohan G. A retrospective analysis of 34 potentially missed cases of female genital mutilation in the emergency department. Emergenct Medicine Journal. 2017;35(10):587-9.
100. Middleton J. Preventing violent extremism: the role of doctors. The Lancet. 2016;388(10057):2219-21.
101. Scamell M, Ghumman A. The experience of maternity care for migrant women living with female genital mutilation: A qualitative synthesis. Birth. 2019;46(1):15-23.
102. Ambuel B, Hamberger LK, Guse CE, Melzer-Lange M, Phelan MB, Kistner A. Healthcare can change from within: sustained improvement in the healthcare response to intimate partner violence. Journal of Family Violence. 2013;28(8):833-47.
103. Szilassy E, Drinkwater J, Hester M, Larkins C, Stanley N, Turner W, et al. Making the links between domestic violence and child safeguarding: an evidence-based pilot training for general practice. Health & Social Care in the Community. 2017;25(6):1722-32.
104. Drinkwater J, Stanley N, Szilassy E, Larkins C, Hester M, Feder G. Juggling confidentiality and safety: a qualitative study of how general practice clinicians document domestic violence in families with children. British Journal of General Practice. 2017;67(659):e437-e44.
105. Woodman J, Allister J, Rafi I, de Lusignan S, Belsey J, Petersen I, et al. A simple approach to improve recording of concerns about childmaltreatment in primary care records: developing a quality improvement intervention. British Journal of General Practice. 2012;62(600):e478-e86.
106. Salad J, Verdonk P, de Boer F, Abma TA. "A Somali girl is Muslim and does not have premarital sex. Is vaccination really necessary?" A qualitative study into the perceptions of Somali women in the Netherlands about the prevention of cervical cancer. International Journal for Equity in Health. 2015;14(1):68.
107. Foundations OS. Somalis in London. New York, NY, USA: Open Society Foundations; 2014. Contract No.: ISBN: 978-1-940983-07-3.
108. Murray L, Windsor C, Parker E, Tewfik O. The Experiences of African Women Giving Birth in Brisbane, Australia. Health Care for Women International. 2010;31(5):458-72.
109. Harper Bulman K, McCourt C. Somali refugee women's experiences of maternity care in west London: A case study. Critical Public Health. 2010;12(4):365-80.
110. Wellock VK. Domestic abuse: Black and minority-ethnic women's perspectives. Midwifery. 2010;26(2):181-8.
111. Feder GS, Hutson M, Ramsay J, Taket AR. Women exposed to intimate partner violence: expectations and experiences when they encounter health care professionals: a meta-analysis of qualitative studies. Archives of Internal Medicine. 2006;166(1):22-37.

For peer review only

112. Widmark C, Levál A, Tishelman C, Ahlberg BM. Obstetric care at the intersection of science and culture: Swedish doctors' perspectives on obstetric care of women who have undergone female genital cutting. Journal of Obstetrics and Gynecology. 2010;30(6):553-8.
113. Johnsdotter S. Discrimination of Certain Ethnic Groups? Ethical Aspects of Implementing FGM Legislation in Sweden. Report. Malmo, Sweden: Faculty of Health and Society, University of Malmo; 2009 2009. Contract No.: FoU Rapport 2009:3.
114. Leye E, Powell RA, Nienhuis G, Claeys P, Temmerman M. Health care in Europe for women with genital mutilation. Health Care for Women International. 2006;27(4):362-78.
115. Paliwal P, Ali S, Bradshaw S, Hughes A, Jolly K. Management of type III female genital mutilation in Birmingham, UK: a retrospective audit. Midwifery. 2014;30(3):282-8.
116. Horwood J, Morden A, Bailey JE, Pathak N, Feder G. Assessing for domestic violence in sexual health environments: a qualitative study. Sexually Transmitted Infections. 2017;94(2):88-92.
117. Feder G, Davies RA, Baird K, Dunne D, Eldridge S, Griffiths C, et al. Identification and Referral to Improve Safety (IRIS) of women experiencing domestic violence with a primary care training and support programme: a cluster randomised controlled trial. The Lancet. 2011;378(9805):1788-95.
118. Creighton SM, Liao LM. Tackling female genital mutilation in the UK. BMJ : British Medical Journal. 2013;347:f7150.
119. Turner W, Hester M, Broad J, Szilassy E, Feder G, Drinkwater J, et al. Interventions to improve the response of professionals to children exposed to domestic violence and abuse: a systematic review. Child Abuse Review. 2017;26(1):19-39.
120. Ashby J, Richardson A, Brawley D, E H, editors. National survey of practice and experience of mandatory reporting of female genital mutilation (FGM) amongst sexual health care professionals. 4th Joint Conference of the British HIV Association, BHIVA with the British Association for Sexual Health and HIV, BASHH 2018; 2018; United Kingdom: HIV Medicine.
121. Rymer J, editor Female genital mutilation. RCOG World Congress 2015; 2015; Brisbane, Australia: BJOG: An International Journal of Obstetrics and Gynaecology.
122. Naftalin J, Bewley S. Mandatory reporting of FGM. British Journal of General Practice. 2015;65(638):450-1.
123. Dixon S. The FGM enhanced dataset: how are we going to discuss this with our patients? British Journal of General Practice. 2015;65(641):629.
124. Taket A, Nurse J, Smith K, Watson J, Shakespeare J, Lavis V, et al. Routinely asking women about domestic violence in health settings. BMJ: British Medical Journal. 2003;327(7416):673-6.
125. Emam KE, Mercer J, Moreau K, Grava-Gubins I, Buckeridge D, Jonker E. Physician privacy concerns when disclosing patient data for public health purposes during a pandemic influenza outbreak. BMC Public Health. 2011;11(1):454.
126. Bismark MM, Mathews B, Morris JM, Thomas LA, Studdert DM. Views on mandatory reporting of impaired health practitioners by their treating practitioners: a qualitative study from Australia. BMJ Open. 2016;6:e011988.
127. Feng J-Y, Chen S-J, Wilk NC, Yang W-P, Fetzer S. Kindergarten teachers' experience of reporting child abuse in Taiwan: Dancing on the edge. Children and Youth Services Review. 2009;31(3):405-9.
128. McTavish JR, Kimber M, Devries K, Colombini M, MacGregor JCD, Wathen CN, et al. Mandated reporters' experiences with reporting child maltreatment: a meta-synthesis of qualitative studies. BMJ Open. 2017;7(10):e013942.
129. Gallagher A, Wainwright P, Tompsett H, Atkins C. Findings from a Delphi exercise regarding conflicts of interests, general practitioners and safeguarding children: 'Listen carefully, judge slowly'. Journal of Medical Ethics. 2012;38(2):87-92.
130. Beran R. Mandatory notification of impaired doctors. Internal Medicine Journal. 2014;44(12a):1161-5.

For peer review only

131. Foster R, Olson-Dorff D, Reiland H, Budzak-Garza A. Commitment, confidence and concerns: Assessing health care professionals' child maltreatment reporting attitudes. Child Abuse & Neglect. 2017;67:54-63.
132. Falkiner M, Thomson D, Day A. Teachers' Understanding and Practice of Mandatory Reporting of Child Maltreatment. Children Australia. 2017;42(1):38-48.
133. Talsma M, Bengtsson Boström K, Östberg A-L. Facing suspected child abuse – what keeps Swedish general practitioners from reporting to child protective services? Scandinavian Journal of Primary Health Care. 2013;33(1):21-6.
134. Hodes D. Female Genital Mutilation (FGM): 10 Year Case Series from the First UK Dedicated Clinic - Journal of Adolescent Health. 2018.
135. Erskine K. Collecting data on female genital mutilation. BMJ : British Medical Journal. 2014;348:g3222.
136. Johansen REB, Ziyada MM, Shell-Duncan B, Kaplan AM, Leye E. Health sector involvement in the management of female genital mutilation/cutting in 30 countries. BMC Health Services Research. 2018;18(1):240.
137. Lawson D, Niven B. The Impact of Mandatory Reporting Legislation on New Zealand Secondary School Students' Attitudes towards Disclosure of Child Abuse. International Journal of Children's Rights. 2015;23:491-528.
138. Casla K, Roderick P, Pollock AM. Disclosure of patients' data to the UK Home Office must stop. BMJ : British Medical Journal. 2017;358:j3613.
139. Hiam L. Grenfell survivors shouldn't be afraid to go to hospital. BMJ : British Medical Journal. 2017;358:j3292.
140. Hiam L, Steele S, McKee M. Creating a 'hostile environment for migrants': the British government's use of health service data to restrict immigration is a very bad idea. Health Economics, Policy and Law. 2018;13(2):107-17.
141. Potter JL, Milner A. Tuberculosis: looking beyond 'migrant'as a category to understand experience. Race Equality Foundation Briefing Paper: A Better Health Briefing. 2018;44.
142. Jacoby SD, Smith A. Increasing Certified Nurse-Midwives' Confidence in Managing the Obstetric Care of Women with Female Genital Mutilation/Cutting. *Journal of midwifery & women's health*. 2013;*58* (4),:pp.451-6.
143. Al-Saadi N, Khan H, Auckburally S, Malik B, Al-Saadi A, Saleem A. Reforming medical education: A formal analysis of London medical school's teaching on female genital cutting: EP12. 140. Bjog: An International Journal of Obstetrics and Gynaecology. 2016;123.
144. John N, Raval H, Handa N, editors. Is female genital mutilation adequately taught to medical students? BJOG-AN INTERNATIONAL JOURNAL OF OBSTETRICS AND GYNAECOLOGY; 2018: WILEY 111 RIVER ST, HOBOKEN 07030-5774, NJ USA.
145. Holmes V, Farrington R, Mulongo P. Educating about female genital mutilation. http://dxdoiorg/101080/1473987920161245589. 2016.
146. Horvat L, Horey D, Romios P K-RJ. Cultural competence education for health professionals.: The Cochrane Collaboration; 2014.
147. Truong M, Paradies Y, Priest N. Interventions to improve cultural competency in healthcare: a systematic review of reviews. BMC Health Services Research. 2014;14(1):99.
148. Watt K, Abbott P, Reath J. Developing cultural competence in general practitioners: an integrative review of the literature. BMC Family Practice. 2016;17(1):158.
150. Farage MA, Miller KW, Tzeghai GE, Azuka CE, Sobel JD, Ledger WJ. Female Genital Cutting: Confronting Cultural Challenges and Health Complications Across the Lifespan:. http://dxdoiorg/102217/WHE1463. 2015.

For peer review only

For peer review only

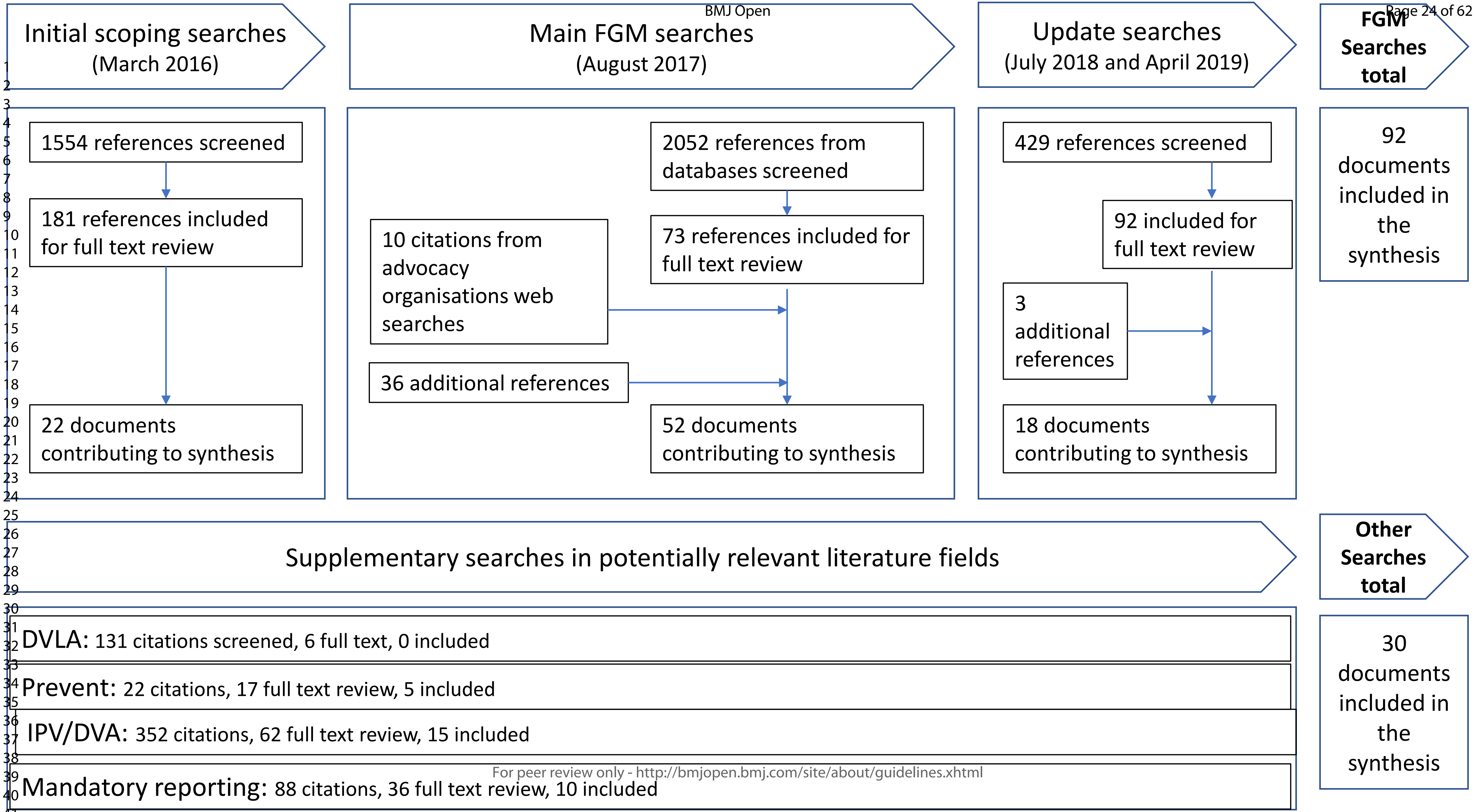
Initial scoping searches (March 2016)
1554 references screened
181 references included for full text review
22 documents contributing to synthesis
Main FGM searches (August 2017)
2052 references from databases screened
73 references included for full text review
10 citations from advocacy organisations web searches
36 additional references
52 documents contributing to synthesis
Update searches (July 2018 and April 2019)
429 references screened
92 included for full text review
3 additional references
18 documents contributing to synthesis
FGM Searches total
92 documents included in the synthesis
Supplementary searches in potentially relevant literature fields
DVLA: 131 citations screened, 6 full text, 0 included
Prevent: 22 citations, 17 full text review, 5 included
IPV/DVA: 352 citations, 62 full text review, 15 included
Mandatory reporting: 88 citations, 36 full text review, 10 included
Other Searches total
30 documents included in the synthesis

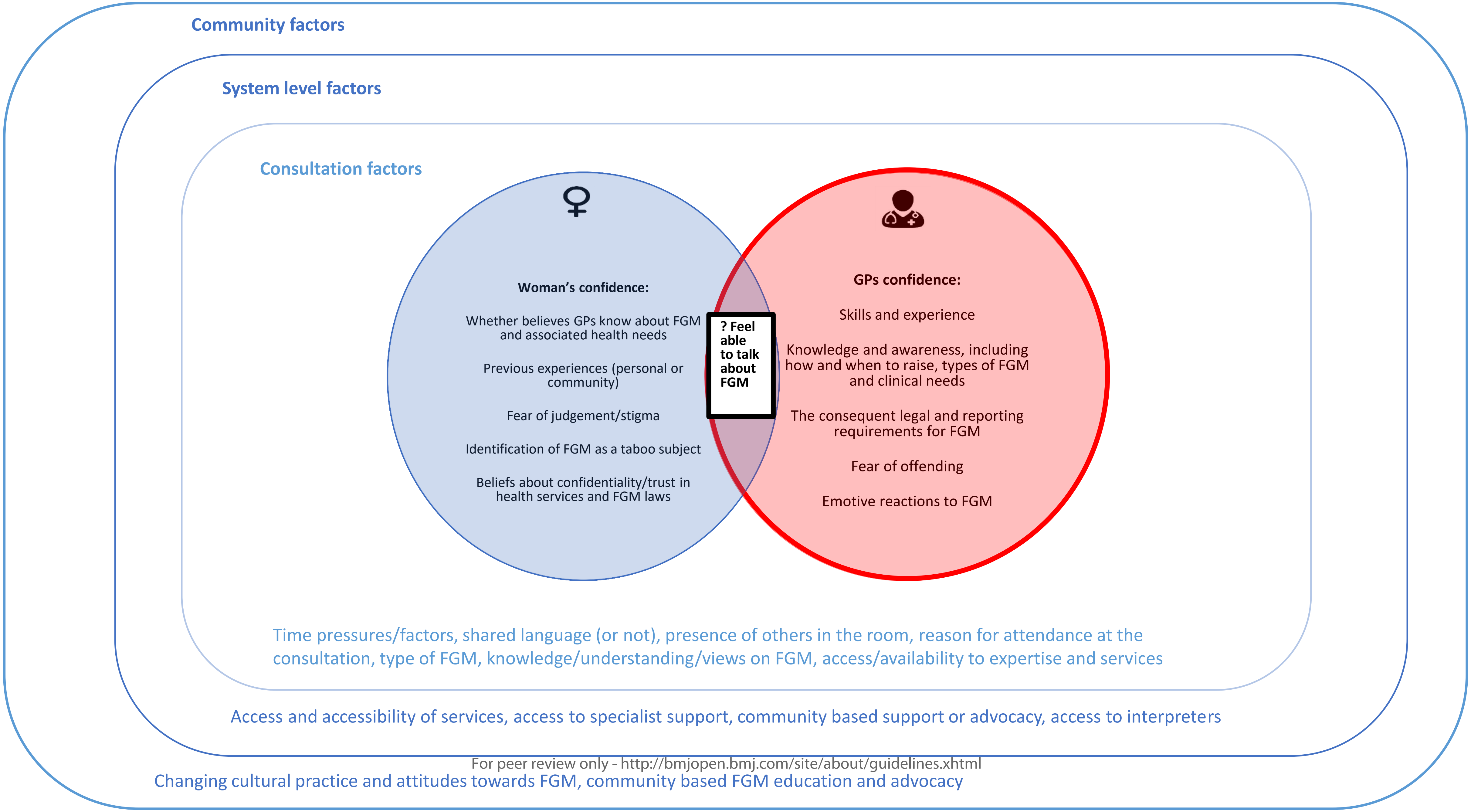
Community factors
System level factors
Consultation factors
Woman's confidence:
Whether believes GPs know about FGM and associated health needs
Previous experiences (personal or community)
Fear of judgement/stigma
Identification of FGM as a taboo subject
Beliefs about confidentiality/trust in health services and FGM laws
? Feel able to talk about FGM
GPs confidence:
Skills and experience
Knowledge and awareness, including how and when to raise, types of FGM and clinical needs
The consequent legal and reporting requirements for FGM
Fear of offending
Emotive reactions to FGM
Time pressures/factors, shared language (or not), presence of others in the room, reason for attendance at the consultation, type of FGM, knowledge/understanding/views on FGM, access/availability to expertise and services
Access and accessibility of services, access to specialist support, community based support or advocacy, access to interpreters
Changing cultural practice and attitudes towards FGM, community based FGM education and advocacy

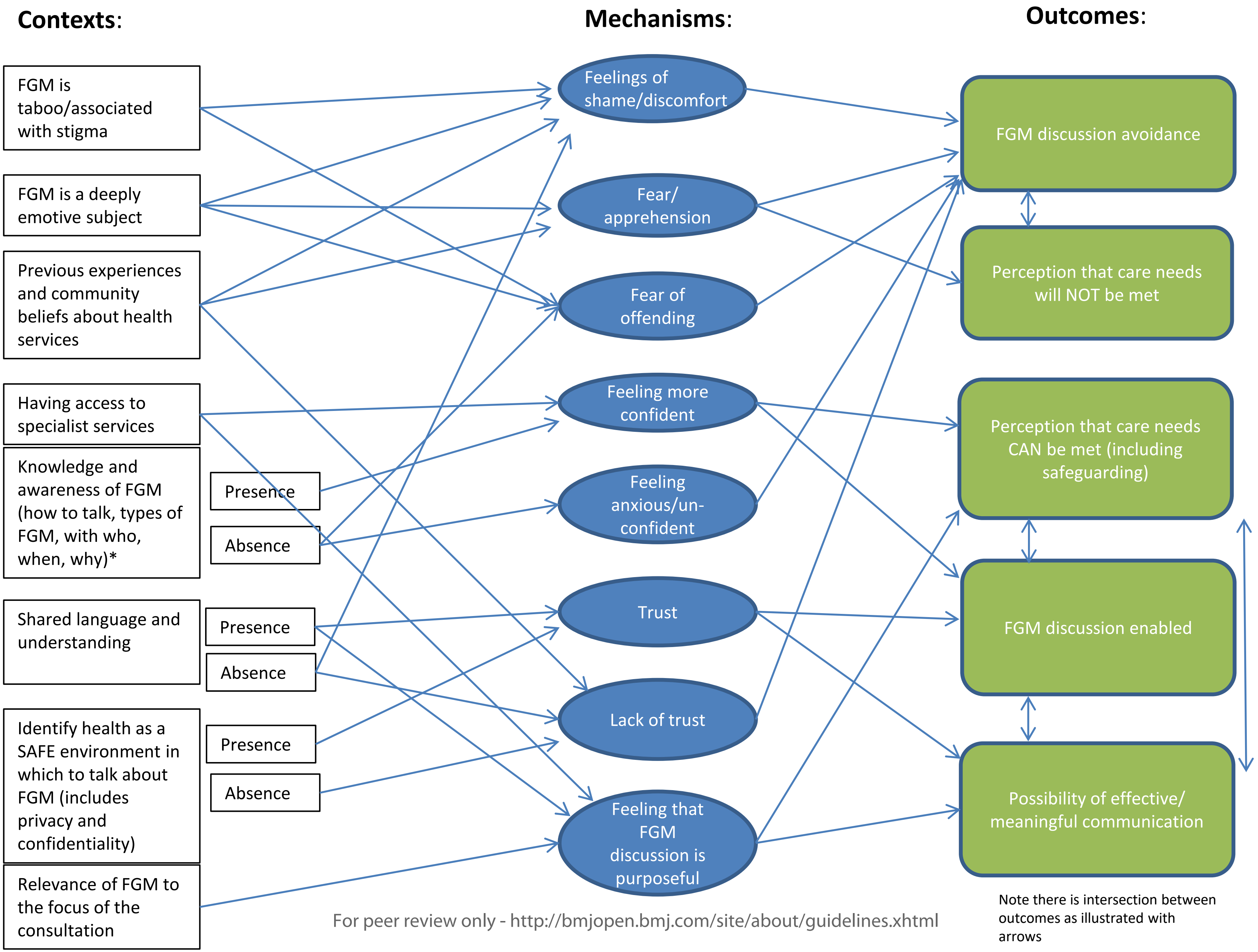


For peer review only - http://bmjopen.bmj.com/site/about/guidelines.xhtml

* This is a Continuum including awareness of lack of knowledge, and lack of this awareness

# English general medical practitioners managing female genital mutilation in light of recent policy and legislative changes: A realist synthesis

Sharon Dixon, Claire Duddy, Gabrielle Harrison, Chrysanthi Papoutsi, Sue Ziebland, Frances Griffiths.

Prospero: CRD4201891996

**Review Question:**

1. From a realist synthesis of published literature, what influences how GPs manage female genital mutilation (FGM) in their clinical practice and why?
2. What influences general medical practitioners (GPs) actions when they consider initiating discussion about female genital mutilation (FGM) with patients in primary care? Where, when and why are these influences active?
3. What influences how GPs respond to a patient-initiated disclosure of FGM during a primary care consultation? Where, when and why are these influences active?

4. **Searches:**

We will use the RAMESES quality standards for realist synthesis to guide this realist review.

We will use an information specialist to support the literature searches.

Initial programme theory will be derived from reviewing governmental policy documents relevant to the management of FGM in primary care and FGM guidance from doctors' professional bodies. The subsequent search strategy will be designed to identify empirical and theoretical literature to test the initial programme theories. The search process in a realist review is iterative and responsive to evolving programme theory. . The preliminary search will be run with keywords identified from an exploratory literature review, and will include FGM, primary care, and general practice/GP. e would search broadly, including using the following databases: Medline, Embase, PsycINFO, Global Health, Cinahl, Web of Science, Sociological Abstracts, Anthropology Plus, Social Science Abstracts and ASSIA, and undertake forward and backward citation searches to identify relevant papers

Subsequent literature searches will be responsive to emergent programme theory including potentially relevant contexts, mechanisms, and outcomes. As new theory emerges, new evidence needs to be considered to test this. We will purposively search and iteratively review evidence. All steps and decisions taken during the review will be reported and justified, including details about actions taken We will report review search strategies (initial and subsequent searches), the number of studies identified and assessed for relevance, the number and type of papers included and excluded and reasons for exclusion.

For peer review only

**Types of study to be included:**

All study designs and theoretical papers, including conceptual papers and commentaries will be considered if they contribute to the development or testing of programme theory. Papers considering interventions in health care contexts very different to the UK healthcare system will be excluded. Where there is a large body of literature, we will identify systematic reviews in the first instance. We will include papers written in English.

**Condition or domain being studied:**

How GPs are managing women and families from FGM affected communities

**Participants/population:**

GPs working in the UK

**Intervention/exposure:**

Any identified factor or context that may influence GPs' actions and reactions in relation to their management of patients affected by FGM.

**Comparator/control:**

Contexts where an identified influence is not present

**Primary outcome:**

This realist review will result in programme theory, tested against existing evidence, on what influences GPs actions and reactions in relation to FGM in primary care consultations and, where, when and why these influences are activated.

**Timing and effect measures:**

No limitation placed on timing of influences.

Effect is defined as action or reaction of GPs in relation to FGM within primary care consultations

**Secondary outcome:**

None

**Data extraction (selection and coding):**

For peer review only

Data extraction and organisation of the data will be undertaken by SD. Documents will be initially screened with title and abstract, and selected for inclusion for full text screening. Following full text screening, relevant articles will be included for coding in Nvivo. A random sample of 10% of finally included documents, screened abstracts, and coded full text documents will be independently checked by another member of the study team as a quality assurance measure. Disagreements will be resolved by discussion within the study team.

The full text of included papers will be uploaded onto qualitative data analysis software tool (NVivo)a. We will code text sections for their relevance (or not) to each programme theory, and within that code context, mechanisms, and outcomes. The coding structure will be initially deductive derived from the initial programme theory, and also inductive, generated to categorise data in included papers. The processes of both coding and analysis are iterative. Through cross comparison of coded data, we expect to generate theory. This may suggest the need for further interrogation of collated evidence and so new coding and searches for new evidence.

**Risk of bias (quality) assessment:**

The study will be guided by the RAMESES quality standards for realist reviews (http://www.ramesesproject.org/Standards_and_Training_materials.php#qual_stand_rs)

Papers will be appraised for relevance to the theory under test, not necessarily the topic under consideration, and for the rigour of the conclusions the authors drew. All included papers will be assessed independently by two team members.

**Strategy for data synthesis:**

Data analysis and synthesis will use a realist logic of analysis. In a realist review, the process of data analysis and synthesis co-exist and overlap. In realist terms, synthesis is progress towards explanations, which are framed as relationships between the contexts (C) in which outcomes under observation occur (O), and the mechanisms that potentially link these (M). These relationships are postulated as CMO configurations (CMOC), which are considered within over-arching programme theory. During analysis, we will use interpretive cross comparison, to understand and explain how different interventions have produced outcomes in different contexts and how we understand the potential mechanisms that link these. During CMOC development, we will review how the data supports the developing CMOCs, how new CMOCs relate to other postulated CMOCs and how they relate to programme theory being developed. The synthesis will include retroductive analysis seeking to identify hidden causes that can explain observed patterns in the data.

**Analysis of subgroups or subsets:**

We will potentially be testing more than one programme theory

For peer review only

.

For peer review only

For peer review only - http://bmjopen.bmj.com/site/about/guidelines.xhtml

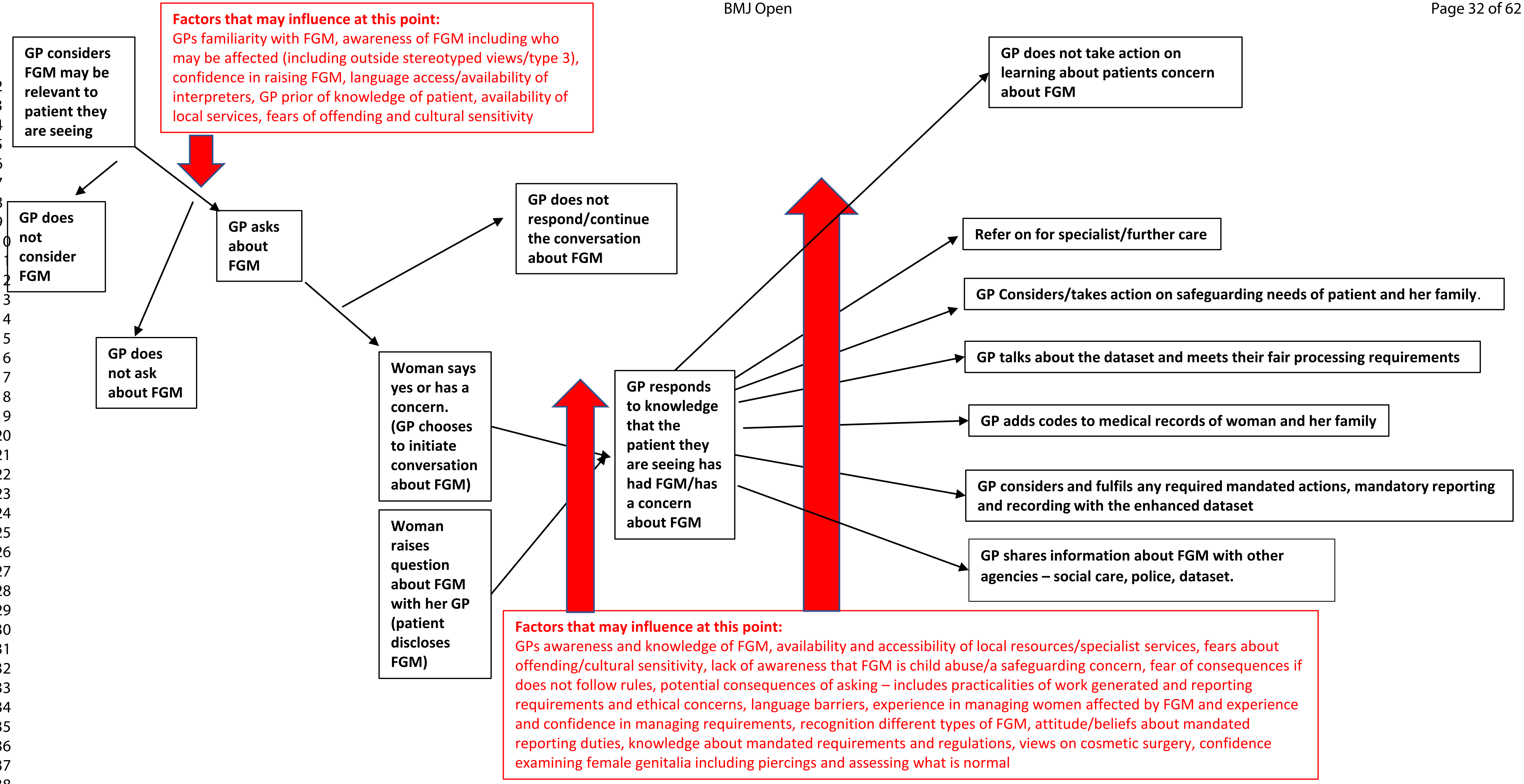


For peer review only - http://bmjopen.bmj.com/site/about/guidelines.xhtml

*The initial programme theory mapped onto a primary care consultation. The black boxes represent hypothesised possible outcomes and the red text and boxes represent potential contextual or mechanistic factors identified from an exploratory literature review and stakeholder expertise.*

# Appendix B: Search strategies

## Initial scoping searches (March 2016)

### Ovid MEDLINE Search #1

| # ▲ | Searches |
|---|---|
| 1 | Circumcision, Female/ |
| 2 | female genital mutilation.ti,ab. |
| 3 | ((female? or women or girl*) adj2 circumcis*).ti,ab. |
| 4 | ((genital? adj2 (cut or cuts or cutting)) and (female? or women or girl*)).ti,ab. |
| 5 | 1 or 2 or 3 or 4 |
| 6 | Prevalence/ |
| 7 | *"surveys and questionnaires"/ or health care surveys/ or health surveys/ |
| 8 | (prevalen* or burden or trend? or estimat* or survey?).ti. |
| 9 | 6 or 7 or 8 |
| 10 | 5 and 9 |
| 11 | limit 10 to yr="2006 -Current" |

### Ovid MEDLINE Search #2

| # ▲ | Searches |
|---|---|
| 1 | Circumcision, Female/ |
| 2 | female genital mutilation.ti,ab. |
| 3 | ((female? or women or girl*) adj2 circumcis*).ti,ab. |
| 4 | ((genital? adj2 (cut or cuts or cutting)) and (female? or women or girl*)).ti,ab. |
| 5 | 1 or 2 or 3 or 4 |
| 6 | (Qualitative systematic review* or (systematic review and qualitative)).ti,ab. |
| 7 | (evidence synthesis or realist synthesis).ti,ab. |
| 8 | (Qualitative and synthesis).ti,ab. |
| 9 | (meta-synthesis* or meta synthesis* or metasynthesis).ti,ab. |
| 10 | (meta-ethnograph* or metaethnograph* or meta ethnograph*).ti,ab. |
| 11 | (meta-study or metastudy or meta study).ti,ab. |
| 12 | (realist review? or realist synthesis).ti,ab. |
| 13 | systematic review*.ti,ab. and qualitative research/ |
| 14 | 6 or 7 or 8 or 9 or 10 or 11 or 12 or 13 |
| 15 | 5 and 14 |
| 16 | qualitative research/ |
| 17 | *interviews as topic/ or focus groups/ or narration/ |
| 18 | observation.ti. |
| 19 | interview?.ti. |
| 20 | (qualitative adj2 (interview* or study)).ti,ab. |
| 21 | (qualitative or focus group? or story or stories or narration or narrative* or discourse or discursive or grounded theory or ethnogra* or phenomenolog*).ti,ab. |
| 22 | 16 or 17 or 18 or 19 or 20 or 21 |
| 23 | 5 and 22 |
| 24 | 15 or 23 |

### Global Health Search #1

| # ▲ | Searches |
|---|---|

For peer review only

1 female genital mutilation.ti,ab.
2 ((female? or women or girl*) adj2 circumcis*).ti,ab.
3 ((genital? adj2 (cut or cuts or cutting)) and (female? or women or girl*)).ti,ab.
4 1 or 2 or 3
5 Prevalence/
6 surveys/ or household surveys/
7 (prevalen* or burden or trend? or estimat* or survey?).ti.
8 5 or 6 or 7
9 4 and 8
10 limit 9 to yr="2006 -Current"

## Global Health Search #2

| # ▲ | Searches |
|---|---|
| 1 | female genital mutilation.ti,ab. |
| 2 | ((genital? adj2 (cut or cuts or cutting)) and (female? or women or girl*)).ti,ab. |
| 3 | ((female? or women or girl*) adj2 circumcis*).ti,ab. |
| 4 | 1 or 2 or 3 |
| 5 | (qualitative or focus group? or story or stories or narration or narrative* or discourse or discursive or grounded theory or ethnogra* or phenomenolog*).ti,ab. |
| 6 | interview*.ti,ab. |
| 7 | 5 or 6 |
| 8 | 4 and 7 |

## Embase

| # ▲ | Searches |
|---|---|
| 1 | exp female genital mutilation/ |
| 2 | female genital mutilation.ti,ab. |
| 3 | ((female? or women or girl*) adj2 circumcis*).ti,ab. |
| 4 | ((genital? adj2 (cut or cuts or cutting)) and (female? or women or girl*)).ti,ab. |
| 5 | 1 or 2 or 3 or 4 |
| 6 | (Qualitative systematic review* or (systematic review and qualitative)).ti,ab. |
| 7 | (evidence synthesis or realist synthesis).ti,ab. |
| 8 | (Qualitative and synthesis).ti,ab. |
| 9 | (meta-synthesis* or meta synthesis* or metasynthesis).ti,ab. |
| 10 | (meta-ethnograph* or metaethnograph* or meta ethnograph*).ti,ab. |
| 11 | (meta-study or metastudy or meta study).ti,ab. |
| 12 | (realist review? or realist synthesis).ti,ab. |
| 13 | systematic review*.mp. and exp qualitative studies/ |
| 14 | 6 or 7 or 8 or 9 or 10 or 11 or 12 or 13 |
| 15 | 5 and 14 |
| 16 | grounded theory/ or naturalistic inquiry/ or qualitative research/ |
| 17 | exp *interview/ |
| 18 | observation.ti. |
| 19 | interview?.ti. |
| 20 | (qualitative adj2 (interview* or study)).ti,ab. |
| 21 | (qualitative or focus group? or story or stories or narration or narrative* or discourse or discursive or grounded theory or ethnogra* or phenomenolog*).ti,ab. |

For peer review only

| | |
|---|---|
| 22 | 16 or 17 or 18 or 19 or 20 or 21 |
| 23 | 5 and 22 |
| 24 | 15 or 23 |

## PsycINFO

| # ▲ | Searches |
|---|---|
| 1 | circumcision/ |
| 2 | female genital mutilation.ti,ab. |
| 3 | ((genital? adj2 (cut or cuts or cutting)) and (female? or women or girl*)).ti,ab. |
| 4 | ((female? or women or girl*) adj2 circumcis*).ti,ab. |
| 5 | 1 or 2 or 3 or 4 |
| 6 | (Qualitative systematic review* or (systematic review and qualitative)).ti,ab. |
| 7 | (evidence synthesis or realist synthesis).ti,ab. |
| 8 | (Qualitative and synthesis).ti,ab. |
| 9 | (meta-synthesis* or meta synthesis* or metasynthesis).ti,ab. |
| 10 | (meta-ethnograph* or metaethnograph* or meta ethnograph*).ti,ab. |
| 11 | (meta-study or metastudy or meta study).ti,ab. |
| 12 | (realist review? or realist synthesis).ti,ab. |
| 13 | systematic review*.ti,ab. and qualitative research/ |
| 14 | 6 or 7 or 8 or 9 or 10 or 11 or 12 or 13 |
| 15 | 5 and 14 |
| 16 | qualitative research/ or grounded theory/ or observation methods/ |
| 17 | exp *interviews/ |
| 18 | observation.ti. |
| 19 | interview?.ti. |
| 20 | (qualitative adj2 (interview* or study)).ti,ab. |
| 21 | (qualitative or focus group? or story or stories or narration or narrative* or discourse or discursive or grounded theory or ethnogra* or phenomenolog*).ti,ab. |
| 22 | 16 or 17 or 18 or 19 or 20 or 21 |
| 23 | 5 and 22 |
| 24 | 15 or 23 |

These search strategies were also adapted and run in

- ASSIA,
- Sociological Abstracts
- CINAHL
- Anthropology Plus
- Web of Science (Core Collection databases)

A total of 1554 references were screened by title and abstract, 181 were read in full text and 22 contributed to the synthesis.

For peer review only

# Main FGM searches (August 2017)

## Summary of searching and results

| Database | Interface | Coverage | Date | GP hits | UK Hits |
|---|---|---|---|---|---|
| Ovid MEDLINE(R) Epub Ahead of Print, In-Process & Other Non-Indexed Citations, Ovid MEDLINE(R) Daily and Ovid MEDLINE(R) | OvidSp | 1946-present | 22/08/2017 | 21 | 384 |
| Embase | OvidSp | 1974 to 2017 August 21 | 22/08/2017 | 31 | 396 |
| PsycINFO | OvidSp | 1967 to August Week 2 2017 | 22/08/2017 | 0 | 57 |
| CINAHL | EBSCOHost | 1982-present | 22/08/2017 | 16 | 575 |
| Web of Science Core Collection | Thomson Reuters | 1945-present | 22/08/2017 | 0 | 280 |
| Nexis UK | Lexis Nexis | | 22/08/2017 | 0 | 260 |
| Proquest Social Science Databases | Proquest | | 22/08/2017 | 5 | 100 |

## MEDLINE

| # ▲ | Searches |
|---|---|
| 1 | Circumcision, Female/ |
| 2 | (femal genital mutilation or fgm).ti,ab. |
| 3 | ((genital? adj2 (cut or cuts or cutting)) and (female? or women or girls)).ti,ab. |
| 4 | ((female? or women or girl*) adj2 circumcis*).ti,ab. |
| 5 | 1 or 2 or 3 or 4 |
| 6 | exp General Practice/ |
| 7 | general practitioners/ or physicians, family/ or physicians, primary care/ |
| 8 | Primary Health Care/ |
| 9 | (((general or family) adj2 (practi* or physician? or doctor?)) or gp or gps).ti,ab. |
| 10 | (doctor? or physician?).ti. |
| 11 | (primary adj2 (care or healthcare)).ti,ab. |
| 12 | 6 or 7 or 8 or 9 or 10 or 11 |
| 13 | exp United Kingdom/ |
| 14 | (united kingdom or uk or britain or gb or england or wales or scotland or northern ireland).ti,ab,in. |
| 15 | (nhs or national health service).ti,ab,in. |
| 16 | (british or bjgp or bmj or hsj or pulse or gp or general practi* or primary care or nursing standard or nursing times).jw. |
| 17 | 13 or 14 or 15 or 16 |
| 18 | 5 and 12 and 17 |
| 19 | 5 and 17 |

## Embase

| # ▲ | Searches |
|---|---|
| 1 | female genital mutilation/ |
| 2 | (femal genital mutilation or fgm).ti,ab. |

For peer review only

3 ((genital? adj2 (cut or cuts or cutting)) and (female? or women or girls)).ti,ab.
4 ((female? or women or girl*) adj2 circumcis*).ti,ab.
5 1 or 2 or 3 or 4
6 General Practice/
7 general practitioner/
8 Primary Medical Care/
9 (((general or family) adj2 (practi* or physician? or doctor?)) or gp or gps).ti,ab.
10 (doctor? or physician?).ti.
11 (primary adj2 (care or healthcare)).ti,ab.
12 6 or 7 or 8 or 9 or 10 or 11
13 exp United Kingdom/
14 (united kingdom or uk or britain or gb or england or wales or scotland or northern ireland).ti,ab,in.
15 (nhs or national health service).ti,ab,in.
16 (british or bjgp or bmj or hsj or pulse or gp or general practi* or primary care or nursing standard or nursing times).jw.
17 13 or 14 or 15 or 16
18 5 and 12 and 17
19 5 and 17

## PsycINFO

| # ▲ | Searches |
|---|---|
| 1 | (exp Human Females/ or exp Female Genitalia/) and exp Circumcision/ |
| 2 | (femal genital mutilation or fgm).ti,ab. |
| 3 | ((genital? adj2 (cut or cuts or cutting)) and (female? or women or girls)).ti,ab. |
| 4 | ((female? or women or girl*) adj2 circumcis*).ti,ab. |
| 5 | 1 or 2 or 3 or 4 |
| 6 | general practitioners/ |
| 7 | Primary Health Care/ |
| 8 | (((general or family) adj2 (practi* or physician? or doctor?)) or gp or gps).ti,ab. |
| 9 | (doctor? or physician?).ti. |
| 10 | (primary adj2 (care or healthcare)).ti,ab. |
| 11 | 6 or 7 or 8 or 9 or 10 |
| 12 | (united kingdom or uk or britain or gb or england or wales or scotland or northern ireland).ti,ab,in. |
| 13 | (nhs or national health service).ti,ab,in. |
| 14 | (british or bjgp or bmj or hsj or pulse or gp or general practi* or primary care or nursing standard or nursing times).jw. |
| 15 | 12 or 13 or 14 |
| 16 | 5 and 11 and 15 |
| 17 | 5 and 15 |

## CINAHL

| # | Query |
|---|---|
| S19 | S5 AND S17 |
| S18 | S5 AND S12 AND S17 |
| S17 | S13 OR S14 OR S15 OR S16 |
| S16 | SO british or bjgp or bmj or hsj or pulse or gp or general practi* or primary care or nursing standard or nursing times) |

For peer review only

| | |
|---|---|
| S15 | TX nhs or "national health service" |
| S14 | TX "united kingdom" or uk or britain or gb or england or wales or scotland or "northern ireland" |
| S13 | (MH "United Kingdom+") |
| S12 | S6 OR S7 OR S8 OR S9 OR S10 OR S11 |
| S11 | TI ( (primary N2 (care or healthcare)) ) OR AB ( (primary N2 (care or healthcare)) ) |
| S10 | TI doctor? or physician? |
| S9 | TI ( (((general or family) N2 (practi* or physician? or doctor?)) or gp or gps) ) OR AB ( (((general or family) N2 (practi* or physician? or doctor?)) or gp or gps) ) |
| S8 | (MH "Primary Health Care") |
| S7 | (MH "Physicians, Family") |
| S6 | (MH "Family Practice") |
| S5 | S1 OR S2 OR S3 OR S4 |
| S4 | TI ( ((female? or women or girl*) N2 circumcis*) ) OR AB ( ((female? or women or girl*) N2 circumcis*) ) |
| S3 | TI ( ((genital? N2 (cut or cuts or cutting)) and (female? or women or girls)) ) OR AB ( ((genital? N2 (cut or cuts or cutting)) and (female? or women or girls)) ) |
| S2 | TI ( "femal genital mutilation" or fgm ) OR AB ( "femal genital mutilation" or fgm ) |
| S1 | (MH "Circumcision, Female") |

## Web of Science (Core Collection)

| Set | Results | |
|---|---|---|
| # 5 | 280 | #3 AND #1 |
| # 4 | 0 | #3 AND #2 AND #1 |
| # 3 | 5,132,695 | TOPIC: ("united kingdom" or uk or britain or gb or england or wales or scotland or "northern ireland") OR ADDRESS: ("united kingdom" or uk or britain or gb or england or wales or scotland or "northern ireland") OR TOPIC: (nhs or "national health service") OR ADDRESS: (nhs or "national health service") OR PUBLICATION NAME: (british or bjgp or bmj or hsj or pulse or gp or general practi* or primary care or nursing standard or nursing times) |
| # 2 | 339,275 | TOPIC: ((((general or family) NEAR/2 (practi* or physician? or doctor?)) or gp or gps)) OR TITLE: (doctor? or physician?) OR TOPIC: ((primary NEAR/2 (care or healthcare))) |
| # 1 | 5,251 | TOPIC: ("femal genital mutilation" or fgm) OR TOPIC: (((genital? NEAR/2 (cut or cuts or cutting)) and (female? or women or girls))) OR TOPIC: (((female? or women or girl*) NEAR/2 circumcis*)) |

## Nexis UK

| | |
|---|---|
| fgm OR "female genital mutilation" | In the Headline |
| gp OR gps OR general practitioners OR doctors | Anywhere in the text |

**Limits**

Previous 2 year

UK publications

## Proquest Social Science Databases

| Set | Search |
|---|---|
| S7 | S3 AND S5 |
| S6 | S3 AND S4 AND S5 |
| S5 | all("united kingdom" or uk or britain or gb or england or wales or scotland or "northern ireland") OR all(nhs OR "national health service") |

For peer review only

S4 all(((general or family) NEAR/2 (practi* or physician or doctor)) or gp or gps) OR ti(doctor OR physician) OR all("primary care" OR "primary healthcare" OR "primary health care")

S3 all("femal genital mutilation" or fgm) OR all((((genital? NEAR/2 (cut or cuts or cutting)) and (female? or women or girls)))) OR all((((female? or women or girl*) NEAR/2 circumcis*)))

A total of 2052 references were screened by title and abstract, 73 were read in full text.

## Website searches

In addition to these searches, a search of relevant advocacy organisation websites was undertaken, including searching:

- Sharon add a list of links to relevant organisation homepages here – just illustrative not everything, say, maybe 5?

## Update searches (July 2018, April 2019)

### Summary of searching and results (July 2018)

| Database | Interface | Coverage | Date | GP hits | UK Hits |
|---|---|---|---|---|---|
| Ovid MEDLINE(R) Epub Ahead of Print, In-Process & Other Non-Indexed Citations, Ovid MEDLINE(R) Daily and Ovid MEDLINE(R) | OvidSp | 1946-present | 22/08/2017 | 21 | 384 |
| Embase | OvidSp | 1974 to 2017 August 21 | 22/08/2017 | 31 | 396 |
| PsycINFO | OvidSp | 1967 to August Week 2 2017 | 22/08/2017 | 0 | 57 |
| CINAHL | EBSCOHost | 1982-present | 22/08/2017 | 16 | 575 |
| Web of Science Core Collection | Thomson Reuters | 1945-present | 22/08/2017 | 0 | 280 |
| Nexis UK | Lexis Nexis | | 22/08/2017 | 0 | 260 |
| Proquest Social Science Databases | Proquest | | 22/08/2017 | 5 | 100 |

Update searches in July 2018 replicated the Main FGM searches shown above, limited to results added to databases from August 2017 onwards.

### Summary of searching and results (April 2019)

| Database | Interface | Coverage | Date | GP hits | Other health profs Hits |
|---|---|---|---|---|---|
| Ovid MEDLINE(R) Epub Ahead of Print, In-Process & Other Non-Indexed Citations, Ovid MEDLINE(R) Daily and Ovid MEDLINE(R) | OvidSp | 1946-present | 23/04/2019 | 2 | 7 |

For peer review only

| | | | | | |
|---|---|---|---|---|---|
| Embase | OvidSp | 1974 to 2017 August 21 | 23/04/2019 | 1 | 31 |
| PsycINFO | OvidSp | 1967 to August Week 2 2017 | 23/04/2019 | 0 | 0 |
| CINAHL | EBSCOHost | 1982-present | 23/04/2019 | 3 | 10 |
| Web of Science Core Collection | Thomson Reuters | 1945-present | 23/04/2019 | 7 | Not searched |
| Nexis UK | Lexis Nexis | | 23/04/2019 | 90 | Not searched |
| Proquest Social Science Databases | Proquest | | 23/04/2019 | 3 | 48 |

Update searches in April 2019 replicated the Main FGM searches shown above, limited to results added to databases from July 2018 onwards. For pragmatic reasons, WoS Core Collection and Nexis UK were only searched for terms relating to GPs/primary care and not other health professional groups.

A total of 429 new references were identified in the update searches and screened by title and abstract, 92 were read in full text.

## Supplementary searches (Various dates)

### Summary of searching and results: DVLA, IPV/DVA and Prevent searches (August 2017)

| Database | Interface | Coverage | Date | DVLA | IPV | Prevent |
|---|---|---|---|---|---|---|
| Ovid MEDLINE(R) Epub Ahead of Print, In-Process & Other Non-Indexed Citations, Ovid MEDLINE(R) Daily and Ovid MEDLINE(R) | OvidSp | 1946-present | 22/08/2017 | 52 | 142 | 7 |
| Embase | OvidSp | 1974 to 2017 August 21 | 22/08/2017 | 137 | 244 | 9 |
| PsycINFO | OvidSp | 1967 to August Week 2 2017 | 22/08/2017 | 8 | 69 | 1 |
| CINAHL | EBSCOHost | 1982-present | 22/08/2017 | 53 | 399 | 69 |
| Web of Science Core Collection | Thomson Reuters | 1945-present | 22/08/2017 | 74 | 150 | 14 |
| Proquest Social Science Databases | Proquest | | 22/08/2017 | 10 | 57 | 16 |

### Summary of searching and results: Mandatory reporting search (May 2018)

| Database | Interface | Coverage | Date | Results |
|---|---|---|---|---|
| PubMed | PubMed | 1946-present | May 2018 | |
| Web of Science (Core Collection) | Thomson Reuters | 1945-present | May 2018 | |

For peer review only

## DVLA (August 2017)

### *MEDLINE*

| # ▲ | Searches |
|---|---|
| 1 | ("driver and vehicle licensing agency" or dvla).ti,ab. |
| 2 | ((driving or driver?) adj5 (notif* or report* or inform*)).ti,ab. |
| 3 | ((driving or driver?) adj5 (ability or competen* or incompeten* or status or continu* or discontinu* or stop* or quit* or cease* or cessation)).ti,ab. |
| 4 | ((driving or driver?) adj3 (licens* or law* or legal*)).ti,ab. |
| 5 | (driv* adj3 (fit or fitness)).ti,ab. |
| 6 | Licensure/ and Automobile Driving/ |
| 7 | 1 or 2 or 3 or 4 or 5 or 6 |
| 8 | exp General Practice/ |
| 9 | general practitioners/ or physicians, family/ or physicians, primary care/ |
| 10 | Primary Health Care/ |
| 11 | (((general or family) adj2 (practi* or physician? or doctor?)) or gp or gps).ti,ab. |
| 12 | (doctor? or physician?).ti. |
| 13 | (primary adj2 (care or healthcare)).ti,ab. |
| 14 | 8 or 9 or 10 or 11 or 12 or 13 |
| 15 | exp United Kingdom/ |
| 16 | (united kingdom or uk or britain or gb or england or wales or scotland or northern ireland).ti,ab,in. |
| 17 | (nhs or national health service).ti,ab,in. |
| 18 | (british or bjgp or bmj or hsj or pulse or gp or general practi* or primary care or nursing standard or nursing times).jw. |
| 19 | 15 or 16 or 17 or 18 |
| 20 | 7 and 14 and 19 |

### *Embase*

| # ▲ | Searches |
|---|---|
| 1 | ("driver and vehicle licensing agency" or dvla).ti,ab. |
| 2 | ((driving or driver?) adj5 (notif* or report* or inform*)).ti,ab. |
| 3 | ((driving or driver?) adj5 (ability or competen* or incompeten* or status or continu* or discontinu* or stop* or quit* or cease* or cessation)).ti,ab. |
| 4 | ((driving or driver?) adj3 (licens* or law* or legal*)).ti,ab. |
| 5 | (driv* adj3 (fit or fitness)).ti,ab. |
| 6 | driving ability/ or driver licence/ |
| 7 | 1 or 2 or 3 or 4 or 5 or 6 |
| 8 | General Practice/ |
| 9 | general practitioner/ |
| 10 | Primary Medical Care/ |
| 11 | (((general or family) adj2 (practi* or physician? or doctor?)) or gp or gps).ti,ab. |
| 12 | (doctor? or physician?).ti. |
| 13 | (primary adj2 (care or healthcare)).ti,ab. |
| 14 | 8 or 9 or 10 or 11 or 12 or 13 |
| 15 | exp United Kingdom/ |
| 16 | (united kingdom or uk or britain or gb or england or wales or scotland or northern ireland).ti,ab,in. |
| 17 | (nhs or national health service).ti,ab,in. |

For peer review only

18 (british or bjgp or bmj or hsj or pulse or gp or general practi* or primary care or nursing standard or nursing times).jw.
19 15 or 16 or 17 or 18
20 7 and 14 and 19

*PsycINFO*

| # ▲ | Searches |
|---|---|
| 1 | ("driver and vehicle licensing agency" or dvla).ti,ab. |
| 2 | ((driving or driver?) adj5 (notif* or report* or inform*)).ti,ab. |
| 3 | ((driving or driver?) adj5 (ability or competen* or incompeten* or status or continu* or discontinu* or stop* or quit* or cease* or cessation)).ti,ab. |
| 4 | ((driving or driver?) adj3 (licens* or law* or legal*)).ti,ab. |
| 5 | (driv* adj3 (fit or fitness)).ti,ab. |
| 6 | 1 or 2 or 3 or 4 or 5 |
| 7 | general practitioners/ |
| 8 | Primary Health Care/ |
| 9 | (((general or family) adj2 (practi* or physician? or doctor?)) or gp or gps).ti,ab. |
| 10 | (doctor? or physician?).ti. |
| 11 | (primary adj2 (care or healthcare)).ti,ab. |
| 12 | 7 or 8 or 9 or 10 or 11 |
| 13 | (united kingdom or uk or britain or gb or england or wales or scotland or northern ireland).ti,ab,in. |
| 14 | (nhs or national health service).ti,ab,in. |
| 15 | (british or bjgp or bmj or hsj or pulse or gp or general practi* or primary care or nursing standard or nursing times).jw. |
| 16 | 13 or 14 or 15 |
| 17 | 6 and 12 and 16 |

*CINAHL*

| | |
|---|---|
| S6 | S3 AND S4 AND S5 |
| S5 | ( ((driving or driver?) N5 (notif* or report* or inform*)) ) OR ( ((driving or driver?) N5 (ability or competen* or incompeten* or status or continu* or discontinu* or stop* or quit* or cease* or cessation)) ) OR ( ((driving or driver?) N3 (licens* or law* or legal*)) ) OR ( (driv* N3 (fit or fitness)) ) OR ( "driver and vehicle licensing agency" or dvla ) |
| S4 | (MH "United Kingdom+") OR ( AB "united kingdom" or uk or britain or gb or england or wales or scotland or "northern ireland" ) OR ( AB nhs or "national health service" ) OR ( SO british or bjgp or bmj or hsj or pulse or gp or general practi* or primary care or nursing standard or nursing times ) OR ( TI "united kingdom" or uk or britain or gb or england or wales or scotland or "northern ireland" ) OR ( TI nhs or "national health service" ) |
| S3 | S1 OR S2 |
| S2 | TI ( (((general or family) N2 (practi* or physician? or doctor?)) or gp or gps) ) OR AB ( (((general or family) N2 (practi* or physician? or doctor?)) or gp or gps) ) OR TI ( doctor? or physician? ) OR TI ( (primary N2 (care or healthcare)) ) OR AB ( (primary N2 (care or healthcare)) ) |
| S1 | (MH "Family Practice") OR (MH "Physicians, Family") OR (MH "Primary Health Care") |

*Web of Science (Core Collection)*

| | | |
|---|---|---|
| # 4 | 74 | #3 AND #2 AND #1 |

For peer review only

| | | |
|---|---|---|
| #3 | 36,160 | TS=("driver and vehicle licensing agency" or dvla) OR TS=(((driving or driver?) NEAR/5 (notif* or report* or inform*))) OR TS=(((driving or driver?) NEAR/5 (ability or competen* or incompeten* or status or continu* or discontinu* or stop* or quit* or cease* or cessation))) OR TS=(((driving or driver?) NEAR/3 (licens* or law* or legal*))) OR TS=((driv* NEAR/3 (fit or fitness))) |
| #2 | 5,132,695 | TS=("united kingdom" or uk or britain or gb or england or wales or scotland or "northern ireland") OR ADDRESS: ("united kingdom" or uk or britain or gb or england or wales or scotland or "northern ireland") OR TS=(nhs or "national health service") OR ADDRESS: (nhs or "national health service") OR PUBLICATION NAME: (british or bjgp or bmj or hsj or pulse or gp or general practi* or primary care or nursing standard or nursing times) |
| #1 | 339,275 | TS=((((general or family) NEAR/2 (practi* or physician? or doctor?)) or gp or gps)) OR TI=(doctor? or physician?) OR TS=((primary NEAR/2 (care or healthcare))) |

*Proquest Social Science Databases*

| | |
|---|---|
| S6 | S1 AND S2 AND S5 |
| S5 | all("driver and vehicle licensing agency" OR dvla) OR all((driving OR driver) NEAR/5 (notif* OR report* OR inform*)) OR all(((driving OR driver) NEAR/5 (ability OR competen* OR incompeten* OR status OR continu* OR discontinu* OR stop* OR quit* OR cease* OR cessation))) OR all(((driving OR driver) NEAR/3 (licens* OR law* OR legal*))) OR all((driv* NEAR/3 (fit OR fitness))) |
| S2 | all(((general or family) NEAR/2 (practi* or physician or doctor)) or gp or gps) OR ti(doctor OR physician) OR all("primary care" OR "primary healthcare" OR "primary health care") |
| S1 | all("united kingdom" or uk or britain or gb or england or wales or scotland or "northern ireland") OR all(nhs OR "national health service") |

A total of 131 citations were screened by title and abstract, 6 were screened in full text and none were included in the final synthesis.

## Prevent (August 2017)

*MEDLINE*

| # ▲ | Searches |
|---|---|
| 1 | (prevent adj (program* or strateg* or initiative or policy)).ti,ab. |
| 2 | (prevent* and (terroris* or counterterroris* or antiterroris* or extremis* or counterextremis* or antiextremis* or radicali* or counterradicali* or antiradicali*)).ti. |
| 3 | (prevent* adj5 (terroris* or counterterroris* or antiterroris* or extremis* or counterextremis* or antiextremis* or radicali* or counterradicali* or antiradicali*)).ti,ab. |
| 4 | 1 or 2 or 3 |
| 5 | exp General Practice/ |
| 6 | general practitioners/ or physicians, family/ or physicians, primary care/ |
| 7 | Primary Health Care/ |
| 8 | (((general or family) adj2 (practi* or physician? or doctor?)) or gp or gps).ti,ab. |
| 9 | (doctor? or physician?).ti. |
| 10 | (primary adj2 (care or healthcare)).ti,ab. |
| 11 | 5 or 6 or 7 or 8 or 9 or 10 |
| 12 | exp United Kingdom/ |
| 13 | (united kingdom or uk or britain or gb or england or wales or scotland or northern ireland).ti,ab,in. |

| | |
|---|---|
| 14 | (nhs or national health service).ti,ab,in. |
| 15 | (british or bjgp or bmj or hsj or pulse or gp or general practi* or primary care or nursing standard or nursing times).jw. |
| 16 | 12 or 13 or 14 or 15 |
| 17 | 4 and 11 and 16 |

*Embase*

| # ▲ | Searches |
|---|---|
| 1 | (prevent adj (program* or strateg* or initiative or policy)).ti,ab. |
| 2 | (prevent* and (terroris* or counterterroris* or antiterroris* or extremis* or counterextremis* or antiextremis* or radicali* or counterradicali* or antiradicali*)).ti. |
| 3 | (prevent* adj5 (terroris* or counterterroris* or antiterroris* or extremis* or counterextremis* or antiextremis* or radicali* or counterradicali* or antiradicali*)).ti,ab. |
| 4 | 1 or 2 or 3 |
| 5 | General Practice/ |
| 6 | general practitioner/ |
| 7 | Primary Medical Care/ |
| 8 | (((general or family) adj2 (practi* or physician? or doctor?)) or gp or gps).ti,ab. |
| 9 | (doctor? or physician?).ti. |
| 10 | (primary adj2 (care or healthcare)).ti,ab. |
| 11 | 5 or 6 or 7 or 8 or 9 or 10 |
| 12 | exp United Kingdom/ |
| 13 | (united kingdom or uk or britain or gb or england or wales or scotland or northern ireland).ti,ab,in. |
| 14 | (nhs or national health service).ti,ab,in. |
| 15 | (british or bjgp or bmj or hsj or pulse or gp or general practi* or primary care or nursing standard or nursing times).jw. |
| 16 | 12 or 13 or 14 or 15 |
| 17 | 4 and 11 and 16 |

*PsycINFO*

| ▲ | Searches |
|---|---|
| 1 | (prevent adj (program* or strateg* or initiative or policy)).ti,ab. |
| 2 | (prevent* and (terroris* or counterterroris* or antiterroris* or extremis* or counterextremis* or antiextremis* or radicali* or counterradicali* or antiradicali*)).ti. |
| 3 | (prevent* adj5 (terroris* or counterterroris* or antiterroris* or extremis* or counterextremis* or antiextremis* or radicali* or counterradicali* or antiradicali*)).ti,ab. |
| 4 | 1 or 2 or 3 |
| 5 | general practitioners/ |
| 6 | Primary Health Care/ |
| 7 | (((general or family) adj2 (practi* or physician? or doctor?)) or gp or gps).ti,ab. |
| 8 | (doctor? or physician?).ti. |
| 9 | (primary adj2 (care or healthcare)).ti,ab. |
| 10 | 5 or 6 or 7 or 8 or 9 |
| 11 | (united kingdom or uk or britain or gb or england or wales or scotland or northern ireland).ti,ab,in. |
| 12 | (nhs or national health service).ti,ab,in. |

For peer review only

| | |
|---|---|
| 13 | (british or bjgp or bmj or hsj or pulse or gp or general practi* or primary care or nursing standard or nursing times).jw. |
| 14 | 11 or 12 or 13 |
| 15 | 4 and 10 and 14 |

*CINAHL*

| | |
|---|---|
| S12 | S3 AND S4 AND S11 |
| S11 | AB ( (prevent N1 (program* or strateg* or initiative or policy)) ) OR TI ( (prevent* and (terroris* or counterterroris* or antiterroris* or extremis* or counterextremis* or antiextremis* or radicali* or counterradicali* or antiradicali*)) ) OR AB ( (prevent* N5 (terroris* or counterterroris* or antiterroris* or extremis* or counterextremis* or antiextremis* or radicali* or counterradicali* or antiradicali*)) ) |
| S4 | (MH "United Kingdom+") OR ( AB "united kingdom" or uk or britain or gb or england or wales or scotland or "northern ireland" ) OR ( AB nhs or "national health service" ) OR ( SO british or bjgp or bmj or hsj or pulse or gp or general practi* or primary care or nursing standard or nursing times ) OR ( TI "united kingdom" or uk or britain or gb or england or wales or scotland or "northern ireland" ) OR ( TI nhs or "national health service" ) |
| S3 | S1 OR S2 |
| S2 | TI ( (((general or family) N2 (practi* or physician? or doctor?)) or gp or gps) ) OR AB ( (((general or family) N2 (practi* or physician? or doctor?)) or gp or gps) ) OR TI ( doctor? or physician? ) OR TI ( (primary N2 (care or healthcare)) ) OR AB ( (primary N2 (care or healthcare)) ) |
| S1 | (MH "Family Practice") OR (MH "Physicians, Family") OR (MH "Primary Health Care") |

*Web of Science (Core Collection)*

| | | |
|---|---|---|
| # 8 | 14 | #7 AND #2 AND #1 |
| # 7 | 2,755 | TS=((prevent NEXT (program* or strateg* or initiative or policy))) OR TI=((prevent* and (terroris* or counterterroris* or antiterroris* or extremis* or counterextremis* or antiextremis* or radicali* or counterradicali* or antiradicali*))) OR TS=((prevent* NEAR/5 (terroris* or counterterroris* or antiterroris* or extremis* or counterextremis* or antiextremis* or radicali* or counterradicali* or antiradicali*))) |
| # 2 | 5,132,695 | TS=("united kingdom" or uk or britain or gb or england or wales or scotland or "northern ireland") OR ADDRESS: ("united kingdom" or uk or britain or gb or england or wales or scotland or "northern ireland") OR TS=(nhs or "national health service") OR ADDRESS: (nhs or "national health service") OR PUBLICATION NAME: (british or bjgp or bmj or hsj or pulse or gp or general practi* or primary care or nursing standard or nursing times) |
| # 1 | 339,275 | TS=((((general or family) NEAR/2 (practi* or physician? or doctor?)) or gp or gps)) OR TI=(doctor? or physician?) OR TS=((primary NEAR/2 (care or healthcare))) |

*Proquest Social Science Databases*

| Set | Search |
|---|---|
| S11 | S1 AND S2 AND S10 |
| S10 | all((prevent NEXT (program* OR strateg* OR initiative OR policy))) OR ti((prevent* AND (terroris* OR counterterroris* OR antiterroris* OR extremis* OR counterextremis* OR antiextremis* OR radicali* OR counterradicali* OR antiradicali*))) OR all((prevent* NEAR/5 (terroris* OR counterterroris* OR antiterroris* OR extremis* OR counterextremis* OR antiextremis* OR radicali* OR counterradicali* OR antiradicali*))) |
| S2 | all(((general or family) NEAR/2 (practi* or physician or doctor)) or gp or gps) OR ti(doctor OR physician) OR all("primary care" OR "primary healthcare" OR "primary health care") |

For peer review only

| | |
|---|---|
| S1 | all("united kingdom" or uk or britain or gb or england or wales or scotland or "northern ireland") OR all(nhs OR "national health service") |

A total of 22 citations were screened by title and abstract, 17 were screened in full text and 5 were included in the final synthesis.

## IPV/DVA (August 2017)

### *MEDLINE*

| # ▲ | Searches |
|---|---|
| 1 | domestic violence/ or spouse abuse/ |
| 2 | ((domestic or spous* or partner? or wife or wives or husband) adj2 (violence or abuse*)).ti,ab. |
| 3 | (batter* adj2 (spous* or partner? or wife or wives or husband)).ti,ab. |
| 4 | 1 or 2 or 3 |
| 5 | exp General Practice/ |
| 6 | general practitioners/ or physicians, family/ or physicians, primary care/ |
| 7 | Primary Health Care/ |
| 8 | (((general or family) adj2 (practi* or physician? or doctor?)) or gp or gps).ti,ab. |
| 9 | (doctor? or physician?).ti. |
| 10 | (primary adj2 (care or healthcare)).ti,ab. |
| 11 | 5 or 6 or 7 or 8 or 9 or 10 |
| 12 | exp United Kingdom/ |
| 13 | (united kingdom or uk or britain or gb or england or wales or scotland or northern ireland).ti,ab,in. |
| 14 | (nhs or national health service).ti,ab,in. |
| 15 | (british or bjgp or bmj or hsj or pulse or gp or general practi* or primary care or nursing standard or nursing times).jw. |
| 16 | 12 or 13 or 14 or 15 |
| 17 | 4 and 11 and 16 |

### *Embase*

| ▲ | Searches |
|---|---|
| 1 | domestic violence/ or battered woman/ or family violence/ or exp partner violence/ |
| 2 | ((domestic or spous* or partner? or wife or wives or husband) adj2 (violence or abuse*)).ti,ab. |
| 3 | (batter* adj2 (spous* or partner? or wife or wives or husband)).ti,ab. |
| 4 | 1 or 2 or 3 |
| 5 | General Practice/ |
| 6 | general practitioner/ |
| 7 | Primary Medical Care/ |
| 8 | (((general or family) adj2 (practi* or physician? or doctor?)) or gp or gps).ti,ab. |
| 9 | (doctor? or physician?).ti. |
| 10 | (primary adj2 (care or healthcare)).ti,ab. |
| 11 | 5 or 6 or 7 or 8 or 9 or 10 |
| 12 | exp United Kingdom/ |
| 13 | (united kingdom or uk or britain or gb or england or wales or scotland or northern ireland).ti,ab,in. |
| 14 | (nhs or national health service).ti,ab,in. |

For peer review only

| | |
|---|---|
| 15 | (british or bjgp or bmj or hsj or pulse or gp or general practi* or primary care or nursing standard or nursing times).jw. |
| 16 | 12 or 13 or 14 or 15 |
| 17 | 4 and 11 and 16 |

*PsycINFO*

| ▲ | Searches |
|---|---|
| 1 | domestic violence/ or battered females/ or intimate partner violence/ or exp partner abuse/ |
| 2 | ((domestic or spous* or partner? or wife or wives or husband) adj2 (violence or abuse*)).ti,ab. |
| 3 | (batter* adj2 (spous* or partner? or wife or wives or husband)).ti,ab. |
| 4 | 1 or 2 or 3 |
| 5 | general practitioners/ |
| 6 | Primary Health Care/ |
| 7 | (((general or family) adj2 (practi* or physician? or doctor?)) or gp or gps).ti,ab. |
| 8 | (doctor? or physician?).ti. |
| 9 | (primary adj2 (care or healthcare)).ti,ab. |
| 10 | 5 or 6 or 7 or 8 or 9 |
| 11 | (united kingdom or uk or britain or gb or england or wales or scotland or northern ireland).ti,ab,in. |
| 12 | (nhs or national health service).ti,ab,in. |
| 13 | (british or bjgp or bmj or hsj or pulse or gp or general practi* or primary care or nursing standard or nursing times).jw. |
| 14 | 11 or 12 or 13 |
| 15 | 4 and 10 and 14 |

*CINAHL*

| | |
|---|---|
| S10 | S3 AND S4 AND S9 |
| S9 | S7 OR S8 |
| S8 | AB ( ((domestic or spous* or partner? or wife or wives or husband) N2 (violence or abuse*)) ) OR AB ( (batter* N2 (spous* or partner? or wife or wives or husband)) ) OR TI ( ((domestic or spous* or partner? or wife or wives or husband) N2 (violence or abuse*)) ) OR TI ( (batter* N2 (spous* or partner? or wife or wives or husband)) ) |
| S7 | (MH "Domestic Violence") OR (MH "Intimate Partner Violence") |
| S4 | (MH "United Kingdom+") OR ( AB "united kingdom" or uk or britain or gb or england or wales or scotland or "northern ireland" ) OR ( AB nhs or "national health service" ) OR ( SO british or bjgp or bmj or hsj or pulse or gp or general practi* or primary care or nursing standard or nursing times ) OR ( TI "united kingdom" or uk or britain or gb or england or wales or scotland or "northern ireland" ) OR ( TI nhs or "national health service" ) |
| S3 | S1 OR S2 |
| S2 | TI ( (((general or family) N2 (practi* or physician? or doctor?)) or gp or gps) ) OR AB ( (((general or family) N2 (practi* or physician? or doctor?)) or gp or gps) ) OR TI ( doctor? or physician? ) OR TI ( (primary N2 (care or healthcare)) ) OR AB ( (primary N2 (care or healthcare)) ) |
| S1 | (MH "Family Practice") OR (MH "Physicians, Family") OR (MH "Primary Health Care") |

*Web of Science (Core Collection)*

| | | |
|---|---|---|
| # 6 | 150 | #5 AND #2 AND #1 |
| # 5 | 15,568 | TS=(((domestic or spous* or partner? or wife or wives or husband) NEAR/2 (violence or abuse*))) OR TS=((batter* NEAR/2 (spous* or partner? or wife or wives or husband))) |

| | | |
|---|---|---|
| # 2 | 5,132,695 | TS=("united kingdom" or uk or britain or gb or england or wales or scotland or "northern ireland") OR ADDRESS: ("united kingdom" or uk or britain or gb or england or wales or scotland or "northern ireland") OR TS=(nhs or "national health service") OR ADDRESS: (nhs or "national health service") OR PUBLICATION NAME: (british or bjgp or bmj or hsj or pulse or gp or general practi* or primary care or nursing standard or nursing times) |
| # 1 | 339,275 | TS=((((general or family) NEAR/2 (practi* or physician? or doctor?)) or gp or gps)) OR TI=(doctor? or physician?) OR TS=((primary NEAR/2 (care or healthcare))) |

*Proquest Social Science Databases*

| | |
|---|---|
| S8 | S1 AND S2 AND S7 |
| S7 | all(((domestic or spous* or partner? or wife or wives or husband) NEAR/2 (violence or abuse*))) OR all((batter* NEAR/2 (spous* or partner? or wife or wives or husband))) |
| S2 | all(((general or family) NEAR/2 (practi* or physician or doctor)) or gp or gps) OR ti(doctor OR physician) OR all("primary care" OR "primary healthcare" OR "primary health care") |
| S1 | all("united kingdom" or uk or britain or gb or england or wales or scotland or "northern ireland") OR all(nhs OR "national health service") |

A total of 352 citations were screened by title and abstract, 62 were screened in full text and 15 were included in the final synthesis.

## Mandatory reporting

*PubMed*

| Search | Query |
|---|---|
| #29 | Select 6 document(s) Filters: published in the last 10 years; English |
| #28 | Search (((((relig* OR muslim* OR islam* or "far right") AND (extremis* OR terror* or radicalisation OR radicalization)) OR (((relig* OR muslim* OR islam* or "far right" OR extremis* OR terror* or radicalisation OR radicalization) AND ((prevent strateg*[Title/Abstract]) OR prevent program*[Title/Abstract])))) AND "last 10 years"[PDat] AND English[lang])) AND ((uk OR "united kingdom" OR gb OR britian OR england OR wales OR scotland OR "northern ireland" OR nhs OR british) AND "last 10 years"[PDat] AND English[lang]) Filters: published in the last 10 years; English |
| #27 | Search uk OR "united kingdom" OR gb OR britian OR england OR wales OR scotland OR "northern ireland" OR nhs OR british Filters: published in the last 10 years; English |
| #26 | Search ((relig* OR muslim* OR islam* or "far right") AND (extremis* OR terror* or radicalisation OR radicalization)) OR (((relig* OR muslim* OR islam* or "far right" OR extremis* OR terror* or radicalisation OR radicalization) AND ((prevent strateg*[Title/Abstract]) OR prevent program*[Title/Abstract]))) Filters: published in the last 10 years; English |

For peer review only

| | |
|---|---|
| #24 | Search (((((relig* OR muslim* OR islam* or "far right") AND (extremis* OR terror* or radicalisation OR radicalization))) AND ("last 10 years"[PDat] AND English[lang] AND (infant[MeSH] OR child[MeSH] OR adolescent[MeSH])))) OR (((((relig* OR muslim* OR islam* or "far right" OR extremis* OR terror* or radicalisation OR radicalization) AND ("last 10 years"[PDat] AND English[lang] AND (infant[MeSH] OR child[MeSH] OR adolescent[MeSH])))) AND (((prevent strateg*[Title/Abstract]) OR prevent program*[Title/Abstract]) AND ("last 10 years"[PDat] AND English[lang] AND (infant[MeSH] OR child[MeSH] OR adolescent[MeSH])))) AND ("last 10 years"[PDat] AND English[lang] AND (infant[MeSH] OR child[MeSH] OR adolescent[MeSH]))) Filters: published in the last 10 years; English |
| #23 | Search (((((relig* OR muslim* OR islam* or "far right") AND (extremis* OR terror* or radicalisation OR radicalization))) AND ("last 10 years"[PDat] AND English[lang] AND (infant[MeSH] OR child[MeSH] OR adolescent[MeSH])))) OR (((((relig* OR muslim* OR islam* or "far right" OR extremis* OR terror* or radicalisation OR radicalization) AND ("last 10 years"[PDat] AND English[lang] AND (infant[MeSH] OR child[MeSH] OR adolescent[MeSH])))) AND (((prevent strateg*[Title/Abstract]) OR prevent program*[Title/Abstract]) AND ("last 10 years"[PDat] AND English[lang] AND (infant[MeSH] OR child[MeSH] OR adolescent[MeSH])))) AND ("last 10 years"[PDat] AND English[lang] AND (infant[MeSH] OR child[MeSH] OR adolescent[MeSH]))) Filters: published in the last 10 years; English; Child: birth-18 years |
| #22 | Search (((relig* OR muslim* OR islam* or "far right" OR extremis* OR terror* or radicalisation OR radicalization) AND ("last 10 years"[PDat] AND English[lang] AND (infant[MeSH] OR child[MeSH] OR adolescent[MeSH])))) AND (((prevent strateg*[Title/Abstract]) OR prevent program*[Title/Abstract]) AND ("last 10 years"[PDat] AND English[lang] AND (infant[MeSH] OR child[MeSH] OR adolescent[MeSH]))) Filters: published in the last 10 years; English; Child: birth-18 years |
| #21 | Search relig* OR muslim* OR islam* or "far right" OR extremis* OR terror* or radicalisation OR radicalization Filters: published in the last 10 years; English; Child: birth-18 years |
| #20 | Search (prevent strateg*[Title/Abstract]) OR prevent program*[Title/Abstract] Filters: published in the last 10 years; English; Child: birth-18 years |
| #19 | Search ((relig* OR muslim* OR islam* or "far right") AND (extremis* OR terror* or radicalisation OR radicalization)) Filters: published in the last 10 years; English; Child: birth-18 years |
| #15 | Search "moral imperative" OR "ethical imperative" OR "emotional imperative" Filters: published in the last 10 years; English; Child: birth-18 years |
| #14 | Search "moral imperative" OR "ethical imperative" OR "emotional imperative" Filters: published in the last 10 years; Child: birth-18 years |
| #13 | Search "moral imperative" OR "ethical imperative" OR "emotional imperative" Filters: Child: birth-18 years |
| #12 | Search ((mandatory report*[tiab] OR mandated report*[tiab] OR mandatory notif*[tiab] OR mandated notif*[tiab])) OR (mandat*[ti] AND (report*[ti] OR notif*[ti])) Filters: Child: birth-18 years |
| #10 | Search ((mandatory report*[tiab] OR mandated report*[tiab] OR mandatory notif*[tiab] OR mandated notif*[tiab])) OR (mandat*[ti] AND (report*[ti] OR notif*[ti])) |
| #11 | Search ((mandatory report*[tiab] OR mandated report*[tiab] OR mandatory notif*[tiab] OR mandated notif*[tiab])) OR (mandat*[ti] AND (report*[ti] OR notif*[ti])) Filters: Systematic Reviews |

For peer review only

| | |
|---|---|
| #9 | Search mandat*[ti] AND (report*[ti] OR notif*[ti]) |
| #8 | Search mandatory report*[tiab] OR mandated report*[tiab] OR mandatory notif*[tiab] OR mandated notif*[tiab] |
| #7 | Search (((physician*[Title] OR doctor*[Title] OR general practi*[Title] OR family physician*[Title] OR "primary care"[Title]))) AND ((educat*[Title] OR learn*[Title] OR teach*[Title] OR train*[Title])) Filters: Systematic Reviews; published in the last 10 years; English |
| #6 | Search (((physician*[Title] OR doctor*[Title] OR general practi*[Title] OR family physician*[Title] OR "primary care"[Title]))) AND ((educat*[Title] OR learn*[Title] OR teach*[Title] OR train*[Title])) Filters: Systematic Reviews; English |
| #5 | Search (((physician*[Title] OR doctor*[Title] OR general practi*[Title] OR family physician*[Title] OR "primary care"[Title]))) AND ((educat*[Title] OR learn*[Title] OR teach*[Title] OR train*[Title])) Filters: Systematic Reviews |
| #4 | Search (((physician*[Title] OR doctor*[Title] OR general practi*[Title] OR family physician*[Title] OR "primary care"[Title]))) AND ((educat*[Title] OR learn*[Title] OR teach*[Title] OR train*[Title])) |
| #3 | Search (educat*[Title] OR learn*[Title] OR teach*[Title] OR train*[Title]) |
| #2 | Search (physician*[Title] OR doctor*[Title] OR general practi*[Title] OR family physician*[Title] OR "primary care"[Title]) |

*Web of Science (Core Collection)*

| | | |
|---|---|---|
| # 29 | 8 | #25 AND #23 |
| # 28 | 88 | #27 AND #10 |
| # 27 | 521 | #23 AND #21 |
| # 26 | 63 | TS=("prevent strateg*" OR "prevent program*") |
| # 25 | 165 | TS=("prevent strateg*" OR "prevent program*") |
| # 24 | 521 | #23 AND #21 |
| # 23 | 1,576,775 | TS=(physician* OR doctor* OR "general practi" OR "family practi*" OR "primary care" OR healthcare OR "health care") OR TI=(health* OR medicine* OR nurs*) |
| # 22 | 2 | #21 AND #12 |
| # 21 | 5,487 | #20 OR #19 |
| # 20 | 1,960 | TS=((relig* OR muslim* OR islam* or "far right") NEAR/5 (extremis* OR terror* or radicalisation OR radicalization)) |
| # 19 | 3,531 | TS=("home office" OR "illegal migrant*" OR "illegal immigra*" OR "undocumented migran*" OR "undocumented immigran*" OR illegals) |
| # 18 | 363 | #15 OR #13 Refined by: PUBLICATION YEARS: ( 2018 OR 2008 OR 2017 OR 2016 OR 2015 OR 2014 OR 2013 OR 2012 OR 2011 OR 2010 OR 2009 ) AND [excluding] DOCUMENT TYPES: ( EDITORIAL MATERIAL OR MEETING ABSTRACT OR BOOK REVIEW OR LETTER OR NEWS ITEM OR PROCEEDINGS PAPER ) |
| # 17 | 405 | #15 OR #13 Refined by: PUBLICATION YEARS: ( 2018 OR 2008 OR 2017 OR 2016 OR 2015 OR 2014 OR 2013 OR 2012 OR 2011 OR 2010 OR 2009 ) |
| # 16 | 597 | #15 OR #13 |
| # 15 | 321 | #14 AND #12 |
| # 14 | 64,467 | TS=((child* OR infant* OR teen* OR adolescen*) NEAR/3 (abuse* OR violence OR maltreat*)) OR TI=((child* OR infant* OR teen* OR adolescen*) AND (abuse* OR violence OR maltreat*)) OR TS=(safeguarding OR "safe guarding") |
| # 13 | 290 | #12 AND #10 |

For peer review only - http://bmjopen.bmj.com/site/about/guidelines.xhtml

| | | |
|---|---|---|
| # 12 | 2,582 | TOPIC: ((mandat* OR compulsory) NEAR/3 (report* OR notif*)) OR TITLE: ((mandat* OR compulsory) AND (report* OR notif*)) |
| # 11 | 64 | #10 AND #9 |
| # 10 | 5,653,563 | TOPIC: ("united kingdom" or uk or britain or british or gb or england or "northern ireland" or scotland or wales or nhs) OR ADDRESS: ("united kingdom" or uk or britain or british or gb or england or "northern ireland" or scotland or wales or nhs) |
| # 9 | 787 | TOPIC: ("cultural competenc*") AND TOPIC: (physician* OR doctor* OR "general practi*" OR "family practi" OR "primary care") |
| # 8 | 128 | TITLE: ("cultural competenc*") AND TOPIC: (educat* OR learn* OR teach* OR train*) AND TOPIC: (physician* OR doctor* OR "general practi*" OR "family practi" OR "primary care") |
| # 7 | 179 | TOPIC: ("moral imperative" OR "ethical imperative" OR "emotional imperative") AND TOPIC: (educat* OR learn* OR teach* OR train*) |
| # 6 | 4 | TOPIC: ("moral imperative" OR "ethical imperative" OR "emotional imperative") AND TOPIC: ((child* OR adolecen* OR teen* OR infan*) NEAR/5 (abuse* OR violence OR maltreat*)) |
| # 5 | 0 | TITLE: ("moral imperative" OR "ethical imperative" OR "emotional imperative") AND TOPIC: ((child* OR adolecen* OR teen* OR infan*) NEAR/5 (abuse* OR violence OR maltreat*)) |
| # 4 | 287 | TITLE: ("moral imperative" OR "ethical imperative" OR "emotional imperative") |
| # 3 | 1 | TOPIC: ("moral imperative" OR "ethical imperative" OR "emotional imperative") AND TOPIC: (((mandat* OR compulsory) NEAR/2 (report* OR notif*))) |
| # 2 | 55 | TOPIC: ("moral imperative" OR "ethical imperative" OR "emotional imperative") AND TOPIC: (doctor* OR physician* OR "general practi*" OR "family practi*") |
| # 1 | 891 | TOPIC: ("moral imperative" OR "ethical imperative" OR "emotional imperative") |

A total of 88 citations were screened by title and abstract, 36 were screened in full text and 10 were included in the final synthesis.

For peer review only

# Appendix A. Full set of derived CMOC/full programme theory.

**The need for FGM knowledge and awareness:**

1) Lacking knowledge or skills (including about the cultural contexts of FGM, safeguarding requirements, who might be affected, and the different types of FGM and their clinical consequences) impacts on GPs' ability to provide optimal care for women affected by FGM (1-7) (8, 9). Practitioners may not be aware that they lack knowledge, including which patients may be affected and their care needs (C). This lack of knowledge (M) results in their inability to meet their care needs. (O) (10-21) (22) (23).
2) Lacking the necessary knowledge and skills (C), is associated with a lack of confidence (M) which impacts on clinical care for women with FGM (O) (10, 24, 25). This includes having the knowledge and confidence to consider who may be at risk (26).
3) Feeling that they have adequate knowledge (including how to respond to a disclosure) (C) helped clinicians feel confident (M) to ask to ask (O) (27, 28).
4) Women who perceive healthcare professionals lack knowledge and skills to manage FGM (C), or who have experiences stigma (C) may lack confidence that health services (M) will meet their care needs (O) (5, 24, 29-32) (33) (10).
5) (C)Health professionals experience emotional reactions to encountering FGM such as anger, shock, and pity (34-37); they find encountering FGM without adequate knowledge is "frightening"(35)(M). Professionals try to hide their reactions but are aware their reaction may be apparent to the women (23) (O).
6) Community members see health care providers reacting with shock or horror to their FGM (C). This provokes feelings of shame (M) which reduces their likelihood of accessing services (O) (38-41) (22) (40) (42).
7) However, clinicians confident in managing FGM (C) are able to reassure women (M) and meet their care needs (O) (28, 43, 44).
8) Healthcare professionals may experience a strong emotional responses to FGM (C). This may make them feel panicked or frightened (M) and abandon their usual routines and practices (O) (28, 37).
9) FGM is usually a relatively small part of the GP workload (C) (45). GPs may not identify learning about FGM as a priority (M) for themselves. The GPs then lack knowledge and skills (O)(46).
10) Clinicians who encounter FGM more frequently in the line of their work (C), may become sensitised to FGM (M) and motivated (M) to learn more or develop their knowledge and skills (O). The converse may also be true (8, 12, 14, 47).
11) FGM can be difficult to correctly identify (48), especially types 1 and 4 and associated with less symptoms (20, 49-51) (C) and GPs may not have the expertise or confidence (M) to correctly identify or manage FGM (O) (52, 53).

**Talking about FGM and communication:**

12) A key skill GPs need is being able to talk about FGM sensitively (10, 21, 54). Fears (M) of offending women by not knowing how to raise the issue(C) can lead GPs to avoid talking about FGM (O) (4, 5, 16, 23, 29, 35, 48, 55-57) (22) (56).
13) Professionals who understand that FGM can be sensitive or taboo subject (C) (29, 58, 59) may be fearful of offending women (M) and avoid discussing FGM with women (O) (54, 55) (23). This contextual factor may be evolving as community attitudes towards the practice of FGM change including meaning that talking about FGM is less taboo in some communities (37, 60-64).
14) Not being aware of or not recognising evolving community practices (C) risks (M) offending community members thereby reducing effective communication/consultations (O) (37). Or that professionals are not able to accurately appraise risk (11) (61).
15) Raising FGM in a way that is normalised within the consultation, for example as a standard question on an assessment form (C) may reduce the embarrassment (M) and facilitate asking (O) (23). Prompts in the records may help clinicians to do this (65) (66).

For peer review only

16) Challenges around the use of terminology can complicate communication between GPs and their patients affected by FGM. Some women may find the terms FGM offensive or frightening (67) (68), or if the term FGM is not familiar to the woman (C) (69), or she does not align her cultural practice (for example labial elongation) with FGM (C) (70), then she may not relate her experience to FGM (M) or know how to reply if a GP asks her about FGM(O) (O) (71).
17) Women's experiences of poor communication and difficulties in engagement with health professionals (e.g. language, cultural differences, perceived judgement), and including non-verbal communication (72), led to them feeling not understood or respected (M), causing a lack of confidence and trust in health services (O) (21, 29, 30, 39, 72, 73) (68).
18) Women who feel pitied or judged(C) may be reluctant (M) to make a disclosure to a health care professional (O) (74, 75).
19) Members of communities affected by FGM acting as health advocates (C) may help promote trust and educate communities and professionals (M) to facilitate access to services (O) (76-78).
20) Language barriers and a lack of understanding of how the health setting works, including communicating with primary care receptionists (C) can make accessing services difficult or stressful (M) and lead to avoidance (O) (79).
21) Whether FGM is relevant to the health concern which the woman brings to her GP appointment (C), could impact on whether the GP or woman are willing to raise or discuss FGM (M), and how such a conversation may be received or experienced (O) (36, 55, 80). This may apply when GPs consider asking women about FGM to consider safeguarding needs within their families, rather than because of their own health needs (37) (81) (82).
22) Coding FGM into medical records introduces potential tensions around balancing the needs of the woman (and her confidentiality) with the potential need of her family (C) which may cause confusion or uncertainty for GPs (M), and lead to improvised strategies or inconsistent coding (O) (82, 83).
23) Women who perceive that the HCP is preoccupied with their FGM (C), can feel disrespected (M) and disengage with health care settings (O) (60) (84, 85) .
24) GPs gender (C) may influence whether the woman or GP feel it is culturally appropriate (M) to talk about FGM (O) (38, 55, 86) (23).
25) Time pressures in the consultation (C) mean GPs may be reluctant (M) to discuss FGM (O). (27, 46, 55, 87)
26) Language barriers are a significant context which influence the conversations between GPs and women about FGM (C) which reduce communication (M) between women and GPs with impacts on communication effectiveness and their care (O) (4, 17, 21, 29, 55, 57, 58, 79, 86, 88) (23).
27) Strategies to address language barriers add their own complications. Official interpreters are recommended, but may not be available(89) or trusted by women, for example if they both perceive FGM as taboo, or she fears they will not respect her confidentiality. This can lead to fear (M) and reduced engagement with health professionals (O) (4, 6, 17, 36, 56, 90) (91) (23) (38).
28) The presence of family members (as interpreters, or witnesses) in the consultation (C) may inhibit GPs feeling able to raise FGM (O) with the women, because of concerns about privacy and confidentiality (M) (4, 17, 55, 92).

**The need for guidelines and access to specialist services:**

29) Researchers and commentators suggest that having access to clear and supportive guidelines about what clinicians should do (C) will enable professionals (M) to ask women about FGM and optimise their care (O)(19, 20, 93, 94) (35). Even when guidelines exist, awareness of them may be incomplete, or they may not be followed, as demonstrated by four UK hospital studies (47, 56, 57, 95). The reasons for this in the case of FGM warrant exploration. Having prompts to normalise asking about

For peer review only

FGM may help clinicians broach the subject (96), especially if linked to training or referral pathways(97).

30) FGM is a complex area for health care professionals to manage, and this management may include needing to report women and their families to other authorities. If professionals are developing awareness of FGM, without accompanying guidance (C), they may experience uncertainty and face what they experience as ethical tensions (M) and risk making incorrect or uncertain decisions regarding reporting (O) (93, 94)
31) Lacking guidance, including guidelines and certainty about what good care comprises (C) can lead to practitioners feeling uncertain (M) and improvising how they offer care (O) (22, 23).
32) Knowing how to react or having access to specialist services (C) may help GPs and community members feel confident (M) to talk about FGM (O) (29, 52, 53, 73, 98).
33) When Health care professionals speak about FGM within a framework of offering support and services (C), it is more likely to be experienced as acceptable by the woman (M, O) (64).
34) Training is more likely to be effective (C) in changing behaviour and promoting asking (O) when it is supported by resources and referral pathways or protocol for intervention (M) (28, 97, 99, 100). Specialist access may be especially important to support practitioners in low prevalence areas (23).

**Mandated actions including mandatory reporting and the FGM Enhanced dataset requirements:**

35) The mandatory reporting duty (C) may cause distress (101)and reduce trust (M) in professionals which may deter women from seeking help or disclosing their FGM (O) (52, 55, 60, 64, 102, 103).
36) Concerns that medical encounters or records are not confidential may cause fear/apprehension (M) and deter women from a disclosure of her needs or concerns (O) (92, 104).
37) The requirement to submit personally identifiable data to the FGM enhanced dataset (C) may reduce women's trust in the confidentiality of the GP consultation (M) and make her reluctant to disclose FGM (O) or make GPs reluctant to raise FGM (O) because of concerns about confidentiality (M) (55, 76, 105-108).
38) The ways in which mandatory reporting or the enhanced dataset are raised in the consultation, including when this happens repeatedly (C), may lead women to feel that the professionals' interest in more in data collection than them, or make them feel judged or fearful (M), and avoid attending healthcare altogether (O) (60)
39) The concern (M) that making a mandated report (C) would have a potentially negative impact on trust (O) in on-going professional relationships (O) was an important potential consideration for professionals (109, 110) (111) (112), and identified as a potential deterrent for help seeking (113).
40) Another concern about managing the legislative requirements includes that FGM can be difficult to identify on examination. GPs may feel that they do not have the skills needed to identify or manage FGM and so not feel confident in being able to identify FGM to confidently code it (O)(68) (46), which may impact on data accuracy (O) (114).
41) Practitioners making mandated reports need to feel confident that their report will be adequately responded to, without causing harm (115) (116). They may be helped by training (115).
42) In addition to lack of knowledge or training (C), practitioners may have concerns about confidentiality (C/M) or fear of causing stigma (C/M) which leads to incomplete or inaccurate coding of FGM (117). Practitioners may perceive a need to feel certain (C) before making a mandated report (O) so that they do not risk making a mistake (M) (118) (119).
43) When young people know that the professional whom they are speaking to is mandated to share the information with other authorities (C), they may feel more reluctant to trust the professional (M), and less likely to make a disclosure (O) (120).
44) A Perceptions of how trustworthy the authority being referred onto may contribute to decisions as to whether or not to disclose. Those who are potentially fearful of authorities or perceive themselves to be vulnerable (C), for example those with uncertain migrant status (C), or if they fear that their disclosure risks placing others at risk of trouble (c), may be more fearful of mandatory reporting or data sharing (M) and avoid accessing services (O) (109) (121-123).

For peer review only

1. Khaja K, Lay K, Boys S. Female Circumcision: Toward an Inclusive Practice of Care. Health Care for Women International. 2010;31(8):686-99.
2. Leval A, Widmark C, Tishelman C, Ahlberg BM. THE ENCOUNTERS THAT RUPTURE THE MYTH: CONTRADICTIONS IN MIDWIVES' DESCRIPTIONS AND EXPLANATIONS OF CIRCUMCISED WOMEN IMMIGRANTS' SEXUALITY. Health Care for Women International. 2010;25(8):743-60.
3. Odemerho BI, Baier M. Female Genital Cutting and the Need for Culturally Competent Communication. The Journal for Nurse Practitioners. 2012;8(6):452-7.
4. Lazar JN, Johnson-Agbakwu CE, Davis OI, Shipp MPL. Providers' Perceptions of Challenges in Obstetrical Care for Somali Women. Obstetrics and Gynecology International. 2013;2013:149640.
5. Johnson-Agbakwu CE, Helm T, Killawi A, Padela AI. Perceptions of obstetrical interventions and female genital cutting: insights of men in a Somali refugee community. Ethnicity and Health. 2014;19(4):440-57.
6. Byrskog U, Olsson P, Essén B, Allvin M-K. Being a bridge: Swedish antenatal care midwives' encounters with Somali-born women and questions of violence; a qualitative study. BMC Pregnancy and Childbirth. 2015;15(1).
7. Varol N, Hall JJ, Black K, Turkmani S, Dawson A. Evidence-based policy responses to strengthen health, community and legislative systems that care for women in Australia with female genital mutilation / cutting. Reproductive Health. 2017;14(1):63.
8. Tantet C, Aupiais C, Bourdon M, Sorge F, Pagès A, Levy D, et al. Female genital mutilation: an evaluation of the knowledge of French general and specialized travel medicine practitioners. Journal of Travel Medicine. 2018;25(1):tax090.
9. González-Timoneda A, Ros VR, González-Timoneda M, Sánchez AC. Knowledge, attitudes and practices of primary healthcare professionals to female genital mutilation in Valencia, Spain: are we ready for this challenge? BMC health services research. 2018;18(1):579.
10. Vangen S, Johansen REB, Sundby J, Træen B, Stray-Pedersen B. Qualitative study of perinatal care experiences among Somali women and local health care professionals in Norway. European Journal of Obstetrics & Gynecology and Reproductive Biology. 2004;112(1):29-35.
11. Tamaddon L, Johnsdotter S, Liljestrand J, Essén B. Swedish Health Care Providers' Experience and Knowledge of Female Genital Cutting. Health Care for Women International. 2006;27(8):709-22.
12. Kaplan-Marcusan A, Torán-Monserrat P, Moreno-Navarro J, Fàbregas MJC, Muñoz-Ortiz L. Perception of primary health professionals about Female Genital Mutilation: from healthcare to intercultural competence. BMC Health Services Research. 2009;9(11).
13. Leye E, Ysebaert I, Deblonde J, Claeys P, Vermeulen G, Jacquemyn Y, et al. Female genital mutilation: Knowledge, attitudes and practices of Flemish gynaecologists. The European Journal of Contraception and Reproductive Health Care. 2009;13(2):182-90.
14. Zaidi N, Khalil A, Roberts C, Browne M. Knowledge of female genital mutilation among healthcare professionals. Journal of Obstetrics and Gynecology. 2009;27(2):161-4.
15. Relph S, Inamdar R, Singh H, Yoong W. Female genital mutilation/cutting: knowledge, attitude and training of health professionals in inner city London. European Journal of Obstetrics & Gynecology and Reproductive Biology. 2013;168(2):195-8.
16. Cappon S, L'Ecluse C, Clays E, Tency I, Leye E. Female genital mutilation: Knowledge, attitude and practices of Flemish midwives. Midwifery. 2015;31(3):e29-e35.
17. Dawson A, Homer CS, Turkmani S, Black K, Varol N. A systematic review of doctors' experiences and needs to support the care of women with female genital mutilation. International Journal of Gynecology & Obstetrics. 2015;131(1):35-40.
18. Dawson A, Turkmani S, Fray S, Nanayakkara S, Varol N, Homer C. Evidence to inform education, training and supportive work environments for midwives involved in the care of women with female genital mutilation: A review of global experience. Midwifery. 2015;31(1):229-38.

For peer review only

19. Zurynski Y, Sureshkumar P, Phu A, Elliott E. Female genital mutilation and cutting: a systematic literature review of health professionals' knowledge, attitudes and clinical practice. BMC international health and human rights. 2015;15:32.
20. Reig-Alcaraz M, Siles-González J, Solano-Ruiz C. A mixed-method synthesis of knowledge, experiences and attitudes of health professionals to Female Genital Mutilation. Journal of Advanced Nursing. 2015;72(2):245-60.
21. Smith H, Stein K. Health information interventions for female genital mutilation. Int ernational Journal of Gynecology and Obstetrics. 2017;136:(136:):79-82.
22. Jordal M, Wahlberg A. Challenges in providing quality care for women with female genital cutting in Sweden–A literature review. Sexual & Reproductive Healthcare. 2018;17:91-6.
23. Evans C, Tweheyo R, McGarry J, Eldridge J, Albert J, Nkoyo V, et al. Crossing cultural divides: A qualitative systematic review of factors influencing the provision of healthcare related to female genital mutilation from the perspective of health professionals. PloS one. 2019;14(3):e0211829.
24. Dawson AJ, Turkmani S, Varol N, Nanayakkara S, Sullivan E, Homer CS. Midwives' experiences of caring for women with female genital mutilation: Insights and ways forward for practice in Australia. Women and Birth. 2015;28(3):207-14.
25. Nash E, Ranka P, editors. FEMALE GENITAL MUTILATION: KNOWLEDGE, CONFIDENCE, AND APPROACH TO CARE IN CLINICAL PRACTICE OF MIDWIVES AND NURSES IN THE UK. 21st FIGO World Congress of Gynecology and Obstetrics; 2015; Vancouver, BC Canada: International Journal of Gynecology and Obstetrics.
26. Ramsay J, Rutterford C, Gregory A, Dunne D, Eldridge S, Sharp D, et al. Domestic violence: knowledge, attitudes, and clinical practice of selected UK primary healthcare clinicians. British Journal of General Practice. 2012;62(602):e647-e55.
27. Feder KH, Angela T, Gene. Violence between intimate partners: working with the whole family. 2008.
28. Sundborg E, Törnkvist L, Saleh-Stattin N, Wändell P, Hylander I. To ask, or not to ask: the hesitation process described by district nurses encountering women exposed to intimate partner violence. Journal of Clinical Nursing. 2017;26(15-16):2256-65.
29. Norman K, Hemmings J, Hussein E, Otoo-Oyortey N. FGM is always with us: Experiences, Perceptions and Beliefs of Women Affected by Female Genital Mutilation in London. Results from a PEER study London: Options Consultancy Services Ltd

FORWARD; 2009.
30. Hussein E, FORWARD. Women's Experiences, perceptions, and attitudes of female Genital Mutilation

The Bristol PEER study FORWARD; 2010.
31. Glover J, Liebling H, Barrett… H. The psychological and social impact of female genital mutilation: A holistic conceptual framework. 2017.
32. Norman K, Belay Gegzabher S, Otoo-Oyortey N. "Between Two Cultures": A Rapid PEER Study Exploring Migrant Communities' Views on Female Genital Mutilation in Essex and Norfolk, UK. London: National FGM Centre

FORWARD; 2016.
33. Mbanya VN, Gele AA, Diaz E, Kumar B. Health care-seeking patterns for female genital mutilation/cutting among young Somalis in Norway. BMC Public Health. 2018;18(1):517.
34. Ogunsiji O. Female Genital Mutilation (FGM): Australian Midwives' Knowledge and Attitudes. Health Care for Women International. 2015;36(11):1179-93.
35. Widmark C, Tishelman C, Ahlberg BM. A study of Swedish midwives' encounters with infibulated African women in Sweden. Midwifery. 2002;18(2):113-25.
36. Horowitz CR, Jackson JC. Female "circumcision" African women confront American medicine. Journal of General Internal Medicine. 1997;12(8):491-9.

For peer review only

37. Johnsdotter S, Essén B. Cultural change after migration: Circumcision of girls in Western migrant communities. Best Practice & Research Clinical Obstetrics & Gynaecology. 2016;32:15-25.
38. Abdullahi A, Copping J, Kessel A, Luck M, Bonell C. Cervical screening: Perceptions and barriers to uptake among Somali women in Camden. Public Health. 2009;123(10):680-5.
39. Oguntoye S, Otoo-Oyortey N, Hemmings J, Norman K, Hussein E. "FGM is with us Everyday": Women and Girls Speak out about Female Genital Mutilation in the UK. World Academy of Science, Engineering and Technology. 2009;54:1020-5.
40. Hamid A, Grace KT, Warren N. A Meta-Synthesis of the Birth Experiences of African Immigrant Women Affected by Female Genital Cutting. Journal of Midwifery & Women's Health. 2018;63(2):185-95.
41. Recchia N, McGarry J. "Don't judge me": narratives of living with FGM. International Journal of Human Rights in Healthcare. 2017;10(1):4-13.
42. del Mar Pastor-Bravo M, Almansa-Martínez P, Jiménez-Ruiz I. Living with mutilation: A qualitative study on the consequences of female genital mutilation in women's health and the healthcare system in Spain. Midwifery. 2018;66:119-26.
43. Vloeberghs E, van der Kwaak A, Knipscheer J, van den Muijsenbergh M. Coping and chronic psychosocial consequences of female genital mutilation in the Netherlands. Ethnicity and Health. 2013;17(6):677-95.
44. Moxey JM, Jones LL. A qualitative study exploring how Somali women exposed to female genital mutilation experience and perceive antenatal and intrapartum care in England. BMJ Open. 2016;6(1):e0009846.
45. Baillot H, Murray N, Connelly E, Howard N. Addressing female genital mutilation in Europe: a scoping review of approaches to participation, prevention, protection, and provision of services. International Journal for Equity in Health. 2018;17(1):21.
46. Baillot H, Murray N, Connelly E, Howard N. Tackling Female Genital Mutilation in Scotland: A Scottish Model of Intervention. Scotland: Scottish Refugee Council

London School of Hygiene and Tropical Medicine 2014.
47. Purchase T, Lamoudi M, Colman S, Allen S, Latthe P, Jolly K. A survey on knowledge of female genital mutilation guidelines - Purchase - 2013 - Acta Obstetricia et Gynecologica Scandinavica - Wiley Online Library. ACTA Obstetricia et Gynecologica Scandinavia. 2013(92:):858–86.
48. Abdulcadir J, Dugerdil A, Boulvain M, Yaron M, Margairaz C, Irion O, et al. Missed opportunities for diagnosis of female genital mutilation. International Journal of Gynecology & Obstetrics. 2014;125(3):256-60.
49. Hodes D, Armitage A, Robinson K, Creighton SM. Female genital mutilation in children presenting to a London safeguarding clinic: a case series. Archives of Disease in Childhood. 2016;101(3):212-6.
50. Hodes D, Armitage A, Dykes A, editors. Female genital mutilation in London and the UNICEF report; a local perspective on worldwide statistics. Annual Conference of the Royal College of Paediatrics and Child Health, RCPCH 2014; 2014 2014-04-01; Birmingham, UK: Archives of Disease in Childhood.
51. Ayadi O'Donnell N, Pall K, Leoni M, Debelle G, Lynn R, Armitage A, et al., editors. Female genital mutilation (FGM) surveillance in under 16 years olds in the UK and Ireland. Royal College of Paediatrics and Child Health Annual Conference, RCPCH 2018; 2018 2018-03-01; United Kingdom: Archives of Disease in Childhood.
52. Mathers N, Rymer J. Mandatory reporting of female genital mutilation by healthcare professionals. British Journal of General Practice. 2015;65(635):282-3.
53. RCGP. Female Genital Mutilation : a clinical approach for GPs 2017 [updated under review 8.3.2017. Available from: http://www.rcgp.org.uk/policy/rcgp-policy-areas/female-genital-mutilation.aspx.

54. Johansen REB. Care for Infibulated Women Giving Birth in Norway: An Anthropological Analysis of Health Workers' Management of a Medically and Culturally Unfamiliar Issue. Medical Anthropology Quarterly. 2006;20(4):516-44.
55. Clayton-Hathaway K. A pilot evaluation of health services for communities affected by FGM/C in Oxfordshire. Oxfordshire: Oxford Against Cutting

Healthwatch Oxfordshire; 2016.
56. Zenner N, Liao LM, Richens Y, Creighton SM. Quality of obstetric and midwifery care for pregnant women who have undergone female genital mutilation. Journal of Obstetrics and Gynecology. 2013;33(5):459-62.
57. Gabrasadig R, Asamoah F, Wilson N, editors. Female genital mutilation: knowledge, training and experience of healthcare professionals at a London hospital. Annual Conference of the Royal College of Paediatrics and Child Health, RCPCH 2015; 2015 2015-04-01; Birmingham, UK: Archives of Disease in Childhood.
58. Upvall MJ, Mohammed K, Dodge PD. Perspectives of Somali Bantu refugee women living with circumcision in the United States: A focus group approach. International Journal of Nursing Studies. 2009;46(3):360-8.
59. Safari F. A qualitative study of women's lived experience after deinfibulation in the UK. Midwifery. 2013;29(2):154-8.
60. Karlsen S, Carver N, Mogilnicka M, Pantazis C. When safeguarding becomes stigmatising: A report on the impact of FGM-safeguarding procedures on people with a Somali heritage living in Bristol. Bristol: University of Bristol; 2019.
61. Creighton SM, Samuel Z, Otoo-Oyortey N, Hodes D. Tackling female genital mutilation in the UK. BMJ. 2019;364:l15.
62. FORWARD. "A Big Wake-Up Call": Participatory Study on Shifts in Attitudes Towards FGM Amongst Community Women in Bristol, Summary Report. FORWARD

Refugee Women of Bristol; 2017.
63. Brown E, Porter C. The Tackling FGM Initiative: Evaluation of the Second Phase

(2013-2016). London: Options Consultancy Services Ltd; 2016.
64. Brown E, Porter C, Unit OP. Evaluation of FGM Prevention among Communities Affected by FGM: A Participatory Ethnographic Evaluation Research (PEER) Study. Endline Phase 2. Options Consultancy Services Ltd; 2016.
65. Horwood J, Morden A, Bailey JE, Pathak N, Feder G. Assessing for domestic violence in sexual health environments: a qualitative study. Sex Transm Infect. 2018;94(2):88-92.
66. Jewkes RK. Preventing domestic violence: most women welcome inquiries, but doctors and nurses rarely ask about it. BMJ : British Medical Journal. 2002;324(7332):253-4.
67. Mohamed N, Schickler P, Warsame Z, Glew C. 'Hear Our Voices': A Report on Participatory Workshops on FGM/C (Female Genital Mutilation/Cutting) with the Somali Community in Tower Hamlets. London: Women's Health and Family Services; 2014.
68. Turkmani S, Homer C, Varol N, Dawson A. A survey of Australian midwives' knowledge, experience, and training needs in relation to female genital mutilation. Women and Birth. 2018;31(1):25-30.
69. Ajibona A, editor The understanding of the term female genital mutilation or FGM amongst patients with FGM in a United Kingdom inner city ante-natal clinic. 21st FIGO World Congress of Gynecology and Obstetrics; 2015; Vancouver, BC Canada: International Journal of Obstetrics and Gynaecology.
70. Ariyo D, Ssali R, King-Webb L, Ikpaahindi S. Voices of the Community:

Exploring Female Genital Mutilation in the African Community

across Greater Manchester. Afruca; 2015.

71. Brown K, Beecham D, Barrett H. The Applicability of Behaviour Change in Intervention Programmes Targeted at Ending Female Genital Mutilation in the EU: Integrating Social Cognitive and Community Level Approaches. Obstetrics and Gynecology International. 2013;2013(2013):324362.
72. Straus L, McEwen A, Hussein FM. Somali women's experience of childbirth in the UK: Perspectives from Somali health workers. Midwifery. 2009;25(2):181-6.
73. Glover J, Liebling H, Barrett H, Goodman S. The psychological and social impact of female genital mutilation: A holistic conceptual framework. Journal of International Studies. 2017;10(2):219-38.
74. Feder G, Wathen CN, MacMillan HL. An evidence-based response to intimate partner violence: WHO guidelines. JAMA. 2013;310(5):479-80.
75. Spangaro J, Koziol-McLain J, Zwi A, Rutherford A, Frail M-A, Ruane J. Deciding to tell: qualitative configurational analysis of decisions to disclose experience of intimate partner violence in antenatal care. Social Science & Medicine. 2016;154:45-53.
76. Dixon S, Agha K, Ali F, El Hindi L, Kelly B, Locock L, et al. Female genital mutilation in the UK- where are we, where do we go next? Involving communities in setting the research agenda. Research Involvement and Engagement. 2018;4(29).
77. Connelly E, Murray N, Baillot H, Howard N. Missing from the debate? A qualitative study exploring the role of communities within interventions to address female genital mutilation in Europe. BMJ Open. 2018;8:e021430.
78. Gordon MMAJH. Management of female genital mutilation : the Northwick Park Hospital experience - McCafrey - 1995 - BJOG: An International Journal of Obstetrics & Gynaecology - Wiley Online Library. 1995.
79. Foundations OS. Somalis in Leicester. New York, NY, USA: Open Society Foundations; 2014.
80. Fawcett RJ, Kernohan G. A retrospective analysis of 34 potentially missed cases of female genital mutilation in the emergency department. Emergenct Medicine Journal. 2017;35(10):587-9.
81. Ambuel B. Healthcare Can Change from Within: Sustained Improvement in the Healthcare Response to Intimate Partner Violence | SpringerLink. 2013.
82. Szilassy E, Drinkwater J, Hester M, Larkins C, Stanley N, Turner W, et al. Making the links between domestic violence and child safeguarding: an evidence-based pilot training for general practice. Health & Social Care in the Community. 2017;25(6):1722-32.
83. Drinkwater J, Stanley N, Szilassy E, Larkins C, Hester M, Feder G. Juggling confidentiality and safety: a qualitative study of how general practice clinicians document domestic violence in families with children. British Journal of General Practice. 2017;67(659):e437-e44.
84. Middleton J. Preventing violent extremism: the role of doctors. The Lancet. 2016;388(10057):2219-21.
85. Scamell M, Ghumman A. The experience of maternity care for migrant women living with female genital mutilation: A qualitative synthesis. Birth. 2019;46(1):15-23.
86. Salad J, Verdonk P, de Boer F, Abma TA. "A Somali girl is Muslim and does not have premarital sex. Is vaccination really necessary?" A qualitative study into the perceptions of Somali women in the Netherlands about the prevention of cervical cancer. International Journal for Equity in Health. 2015;14(1):68.
87. Widmark C, Levál A, Tishelman C, Ahlberg BM. Obstetric care at the intersection of science and culture: Swedish doctors' perspectives on obstetric care of women who have undergone female genital cutting. Journal of Obstetrics and Gynecology. 2010;30(6):553-8.
88. Foundations OS. Somalis in London. New York, NY, USA: Open Society Foundations; 2014. Contract No.: ISBN: 978-1-940983-07-3.
89. Norman K. FGM is always with us

Experiences, Perceptions and Beliefs of Women

Affected by Female Genital Mutilation in London

For peer review only

Results from a PEER study In: Hemmings J, Hussein E, Otoo-Oyortey N, editors. London: FORWARD; 2009. p. 55.
90. Harper Bulman K, McCourt C. Somali refugee women's experiences of maternity care in west London: A case study. Critical Public Health. 2010;12(4):365-80.
91. Wellock VK. Domestic abuse: Black and minority-ethnic women's perspectives. Midwifery. 2010;26(2):181-8.
92. Feder GS, Hutson M, Ramsay J, Taket AR. Women exposed to intimate partner violence: expectations and experiences when they encounter health care professionals: a meta-analysis of qualitative studies. Archives of Internal Medicine. 2006;166(1):22-37.
93. Johnsdotter S. Discrimination of Certain Ethnic Groups? Ethical Aspects of Implementing FGM Legislation in Sweden. Report. Malmo, Sweden: Faculty of Health and Society, University of Malmo; 2009 2009. Contract No.: FoU Rapport 2009:3.
94. Leye E, Powell RA, Nienhuis G, Claeys P, Temmerman M. Health care in Europe for women with genital mutilation. Health Care for Women International. 2006;27(4):362-78.
95. Paliwal P, Ali S, Bradshaw S, Hughes A, Jolly K. Management of type III female genital mutilation in Birmingham, UK: a retrospective audit. Midwifery. 2014;30(3):282-8.
96. Horwood J, Morden A, Bailey JE, Pathak N, Feder G. Assessing for domestic violence in sexual health environments: a qualitative study. Sexually Transmitted Infections. 2017;94(2):88-92.
97. Feder G, Davies RA, Baird K, Dunne D, Eldridge S, Griffiths C, et al. Identification and Referral to Improve Safety (IRIS) of women experiencing domestic violence with a primary care training and support programme: a cluster randomised controlled trial. The Lancet. 2011;378(9805):1788-95.
98. Creighton SM, Liao LM. Tackling female genital mutilation in the UK. BMJ : British Medical Journal. 2013;347:f7150.
99. Feder G, Centre for Academic Primary Care SoSaCM, University of Bristol, Bristol, United Kingdom, Wathen CN, Faculty of Information and Media Studies WU, London, Ontario, Canada, MacMillan HL, Department of Psychiatry and Behavioural Neurosciences MU, Hamilton, Ontario, Canada, et al. An Evidence-Based Response to Intimate Partner Violence: WHO Guidelines. JAMA. 2013;310(5):479-80.
100. Turner W, Hester M, Broad J, Szilassy E, Feder G, Drinkwater J, et al. Interventions to improve the response of professionals to children exposed to domestic violence and abuse: a systematic review. Child Abuse Review. 2017;26(1):19-39.
101. Ashby J, Richardson A, Brawley D, E H, editors. National survey of practice and experience of mandatory reporting of female genital mutilation (FGM) amongst sexual health care professionals. 4th Joint Conference of the British HIV Association, BHIVA with the British Association for Sexual Health and HIV, BASHH 2018; 2018; United Kingdom: HIV Medicine.
102. Plugge E, Adam S, El Hindi L, Gitau J, Shodunke N, Mohamed-Ahmed O. The prevention of female genital mutilation in England: what can be done? Journal of public health (Oxford, England). 2018;fdy128.
103. Rymer J, editor Female genital mutilation. RCOG World Congress 2015; 2015; Brisbane, Australia: BJOG: An International Journal of Obstetrics and Gynaecology.
104. Taket A, Nurse J, Smith K, Watson J, Shakespeare J, Lavis V, et al. Routinely asking women about domestic violence in health settings. BMJ: British Medical Journal. 2003;327(7416):673-6.
105. Bewley S, Kelly B, Darke K, Erskine K, Gerada C, Lohr P, et al. Mandatory submission of patient identifiable information to third parties: FGM now, what next? BMJ : British Medical Journal. 2015;351:h5146.
106. Murray L, Windsor C, Parker E, Tewfik O. The Experiences of African Women Giving Birth in Brisbane, Australia. Health Care for Women International. 2010;31(5):458-72.
107. Naftalin J, Bewley S. Mandatory reporting of FGM. British Journal of General Practice. 2015;65(638):450-1.
108. Dixon S. The FGM enhanced dataset: how are we going to discuss this with our patients? 2015.

For peer review only

109. Emam KE, Mercer J, Moreau K, Grava-Gubins I, Buckeridge D, Jonker E. Physician privacy concerns when disclosing patient data for public health purposes during a pandemic influenza outbreak. BMC Public Health. 2011;11(1):454.
110. Bismark MM, Mathews B, Morris JM, Thomas LA, Studdert DM. Views on mandatory reporting of impaired health practitioners by their treating practitioners: a qualitative study from Australia. BMJ Open. 2016;6:e011988.
111. Feng J-Y, Chen S-J, Wilk NC, Yang W-P, Fetzer S. Kindergarten teachers' experience of reporting child abuse in Taiwan: Dancing on the edge. Children and Youth Services Review. 2009;31(3):405-9.
112. Gallagher A, Wainwright P, Tompsett H, Atkins C. Findings from a Delphi exercise regarding conflicts of interests, general practitioners and safeguarding children: 'Listen carefully, judge slowly'. Journal of Medical Ethics. 2012;38(2):87-92.
113. Beran R. Mandatory notification of impaired doctors. Internal Medicine Journal. 2014;44(12a):1161-5.
114. Erskine K. Collecting data on female genital mutilation. BMJ : British Medical Journal. 2014;348:g3222.
115. Foster R, Olson-Dorff D, Reiland H, Budzak-Garza A. Commitment, confidence and concerns: Assessing health care professionals' child maltreatment reporting attitudes. Child Abuse & Neglect. 2017;67:54-63.
116. McTavish JR, Kimber M, Devries K, Colombini M, MacGregor JCD, Wathen CN, et al. Mandated reporters' experiences with reporting child maltreatment: a meta-synthesis of qualitative studies. BMJ Open. 2017;7(10):e013942.
117. Johansen REB, Ziyada MM, Shell-Duncan B, Kaplan AM, Leye E. Health sector involvement in the management of female genital mutilation/cutting in 30 countries. BMC Health Services Research. 2018;18(1):240.
118. Falkiner M, Thomson D, Day A. Teachers' Understanding and Practice of Mandatory Reporting of Child Maltreatment. Children Australia. 2017;42(1):38-48.
119. Talsma M, Bengtsson Boström K, Östberg A-L. Facing suspected child abuse – what keeps Swedish general practitioners from reporting to child protective services? Scandinavian Journal of Primary Health Care. 2013;33(1):21-6.
120. Lawson D, Niven B. The Impact of Mandatory Reporting Legislation on New Zealand Secondary School Students' Attitudes towards Disclosure of Child Abuse. International Journal of Children's Rights. 2015;23:491-528.
121. Casla K, Roderick P, Pollock AM. Disclosure of patients' data to the UK Home Office must stop. BMJ : British Medical Journal. 2017;358:j3613.
122. Hiam L. Grenfell survivors shouldn't be afraid to go to hospital. BMJ : British Medical Journal. 2017;358:j3292.
123. Hiam L, Steele S, McKee M. Creating a 'hostile environment for migrants': the British government's use of health service data to restrict immigration is a very bad idea. Health Economics, Policy and Law. 2018;13(2):107-17.

For peer review only

Table 1 List of items to be included when reporting a realist synthesis

From: RAMESES publication standards: realist syntheses

TITLE

1 In the title, identify the document as a realist synthesis or review Yes

ABSTRACT

2 While acknowledging publication requirements and house style, abstracts should ideally contain brief details of: the study's background, review question or objectives; search strategy; methods of selection, appraisal, analysis and synthesis of sources; main results; and implications for practice. Yes

INTRODUCTION

3 Rationale for review Explain why the review is needed and what it is likely to contribute to existing understanding of the topic area. Yes

4 Objectives and focus of review State the objective(s) of the review and/or the review question(s). Define and provide a rationale for the focus of the review. Yes

METHODS

5 Changes in the review process Any changes made to the review process that was initially planned should be briefly described and justified. No changes made to the searching described by protocol. As part of our iterative searching, we actively undertook 2 update searches.

6 Rationale for using realist synthesis Explain why realist synthesis was considered the most appropriate method to use. Yes

7 Scoping the literature Describe and justify the initial process of exploratory scoping of the literature. Yes

8 Searching processes While considering specific requirements of the journal or other publication outlet, state and provide a rationale for how the iterative searching was done. Provide details on all the sources accessed for information in the review. Where searching in electronic databases has taken place, the details should include, for example, name of database, search terms, dates of coverage and date last searched. If individuals familiar with the relevant literature and/or topic area were contacted, indicate how they were identified and selected. Yes

9 Selection and appraisal of documents Explain how judgements were made about including and excluding data from documents, and justify these. Yes

10 Data extraction Describe and explain which data or information were extracted from the included documents and justify this selection. Yes

11 Analysis and synthesis processes Describe the analysis and synthesis processes in detail. This section should include information on the constructs analyzed and describe the analytic process. Yes

RESULTS

For peer review only

12 Document flow diagram Provide details on the number of documents assessed for eligibility and included in the review with reasons for exclusion at each stage as well as an indication of their source of origin (for example, from searching databases, reference lists and so on). You may consider using the example templates (which are likely to need modification to suit the data) that are provided. Yes

13 Document characteristics Provide information on the characteristics of the documents included in the review. Yes

14 Main findings Present the key findings with a specific focus on theory building and testing. We have presented the initial theory developed from scoping and presented the evolved programme theory

DISCUSSION

15 Summary of findings Summarize the main findings, taking into account the review's objective(s), research question(s), focus and intended audience(s). Yes

16 Strengths, limitations and future research directions Discuss both the strengths of the review and its limitations. These should include (but need not be restricted to) (a) consideration of all the steps in the review process and (b) comment on the overall strength of evidence supporting the explanatory insights which emerged. Yes

The limitations identified may point to areas where further work is needed.

17 Comparison with existing literature Where applicable, compare and contrast the review's findings with the existing literature (for example, other reviews) on the same topic. Yes

18 Conclusion and recommendations List the main implications of the findings and place these in the context of other relevant literature. If appropriate, offer recommendations for policy and practice. Yes

19 Funding Provide details of funding source (if any) for the review, the role played by the funder (if any) and any conflicts of interests of the reviewers. Yes

For peer review only

BMJ Open

# Conversations about FGM in primary care: A realist review on how, why, and under what circumstances FGM is discussed in general practice consultations.

| | |
|---|---|
| Journal: | *BMJ Open* |
| Manuscript ID | bmjopen-2020-039809.R1 |
| Article Type: | Original research |
| Date Submitted by the Author: | 11-Sep-2020 |
| Complete List of Authors: | Dixon, Sharon; University of Oxford, Nuffield Department of Primary Care Health Sciences<br>Duddy, Claire; University of Oxford, Nuffield Department of Primary Care Health Sciences<br>Harrison, Gabrielle; University Hospitals Coventry and Warwickshire NHS Trust<br>Papoutsi, Chrysanthi; University of Oxford, Nuffield Department of Primary Care Health Sciences<br>Ziebland, Sue; University of Oxford, NDPCHS<br>Griffiths, Frances; University of Warwick, Warwick Medical School; University of the Witwatersrand, Centre for Health Policy |
| <b>Primary Subject Heading</b>: | General practice / Family practice |
| Secondary Subject Heading: | Communication, Patient-centred medicine |
| Keywords: | PRIMARY CARE, Health policy < HEALTH SERVICES ADMINISTRATION & MANAGEMENT, Organisation of health services < HEALTH SERVICES ADMINISTRATION & MANAGEMENT, Quality in health care < HEALTH SERVICES ADMINISTRATION & MANAGEMENT |
| | |

SCHOLARONE™
Manuscripts

*I, the Submitting Author has the right to grant and does grant on behalf of all authors of the Work (as defined in the below author licence), an exclusive licence and/or a non-exclusive licence for contributions from authors who are: i) UK Crown employees; ii) where BMJ has agreed a CC-BY licence shall apply, and/or iii) in accordance with the terms applicable for US Federal Government officers or employees acting as part of their official duties; on a worldwide, perpetual, irrevocable, royalty-free basis to BMJ Publishing Group Ltd ("BMJ") its licensees and where the relevant Journal is co-owned by BMJ to the co-owners of the Journal, to publish the Work in this journal and any other BMJ products and to exploit all rights, as set out in our licence.*

*The Submitting Author accepts and understands that any supply made under these terms is made by BMJ to the Submitting Author unless you are acting as an employee on behalf of your employer or a postgraduate student of an affiliated institution which is paying any applicable article publishing charge ("APC") for Open Access articles. Where the Submitting Author wishes to make the Work available on an Open Access basis (and intends to pay the relevant APC), the terms of reuse of such Open Access shall be governed by a Creative Commons licence – details of these licences and which Creative Commons licence will apply to this Work are set out in our licence referred to above.*

*Other than as permitted in any relevant BMJ Author's Self Archiving Policies, I confirm this Work has not been accepted for publication elsewhere, is not being considered for publication elsewhere and does not duplicate material already published. I confirm all authors consent to publication of this Work and authorise the granting of this licence.*

For peer review only

# Conversations about FGM in primary care: A realist review on how, why, and under what circumstances FGM is discussed in general practice consultations.

Authors:

Sharon Dixon[1], Claire Duddy[1], Gabrielle Harrison[2], Chrysanthi Papoutsi[1], Sue Ziebland[1], Frances Griffiths[3,4]

1=Nuffield Department of Primary Care Health Sciences, University of Oxford

2=University Hospitals Coventry and Warwickshire, NHS trust

3=Warwick Medical School, University of Warwick.

4=Centre for Health Policy, University of the Witwatersrand, South Africa

Corresponding author:

Sharon Dixon

Nuffield Department of Primary Care Health Sciences, Radcliffe Primary Care Building, Radcliffe Observatory Quarter, Woodstock Rd, Oxford OX2 6GG

Telephone: 01865 617865

Email: Sharon.dixon@phc.ox.ac.uk

MESH terms:

FGM

Primary care

Realist synthesis

Word count:

4726

For peer review only

# Conversations about FGM in primary care: A realist review on how, why, and under what circumstances FGM is discussed in general practice consultations.


## Abstract

**Objectives:** Little is known about the management of FGM in primary care. There have been significant recent statutory changes relevant to GPs in England, including a mandatory reporting duty. We undertook a realist synthesis to explore what influences how and when GPs discuss FGM with their patients.

**Setting:** Primary care in England

**Data Sources**: Realist literature synthesis searching ten databases with terms: GPs, primary care, obstetrics, gynaecology, midwifery and FGM (UK and worldwide). Citation chasing was utilised, and relevant grey literature was included, including searching FGM advocacy organisation websites for relevant data. Other potentially relevant literature fields were searched for evidence to inform programme theory development. We included all study designs and papers that presented evidence about factors potentially relevant to considering how, why and in what circumstances GPs feel able to discuss FGM with their patients

**Primary outcome measure:** This realist review developed programme theory, tested against existing evidence, on what influences GPs actions and reactions to FGM in primary care consultations and, where, when and why these influences are activated.

**Results**: 124 documents were included in the synthesis. Our analysis found that GPs need knowledge and training to help them support their patients with FGM, including who may be affected, what needs they may have and , and how to talk sensitively about FGM. Access to specialist services and guidance may help them with this role. Reporting requirements may complicate these conversations.

**Conclusions:** There is a pressing need to develop (and evaluate) training to help GPs meet FGM affected communities' health needs and to promote the accessibility of primary care. Education and resources should be developed in partnership with community members. The impact of the mandatory reporting requirement and the Enhanced dataset on healthcare interactions in primary care warrants evaluation.


**Trial registration:** Prospero 2018 CRD42018091996

**Keywords:** FGM, female genital mutilation, female circumcision, GP, primary care, realist synthesis

## Strengths and limitations of this study:

- A realist approach to synthesis facilitated inclusion of wide range of data sources, and consideration of this research question despite little direct primary care research about FGM, with a comprehensive and iterative approach to data searching for relevant evidence.
- This method facilitated the inclusion of community, charity, and advocacy organisation data contributing evidence that might not have been accessible using other methods.
- We searched widely for data to inform the question in comparative fields.
- The tabulation and characterisation of the published research is itself valuable and highlights potential research gaps.

For peer review only

## Introduction

Female genital mutilation (FGM) is defined as all procedures that intentionally alter or cause injury to the female genitalia for non-medical reasons. There are no known health benefits, and many documented harms, including immediate and long term physical and psychological consequences. FGM is recognised internationally as an act of violence against women and girls. FGM is categorised into 4 types: type 1 (clitoridectomy), type 2 (partial or total removal of clitoris and labia minora/majora), Type 3 (infibulation) and type 4 (all other harmful procedures, including pricking and piercing)(1).

UNICEF estimate that around the world 200 million girls and women in 30 countries have been subjected to FGM. Global migration from areas where FGM is traditionally practiced means that FGM is now a worldwide health concern (2).

In 2011, it was estimated that 137,000 women and girls with FGM, from countries where FGM is traditionally practiced, were permanently resident in England and Wales. Prevalence of FGM was highest in urban areas, but women and girls affected by FGM were likely to live in every local authority area in England and Wales (3).

In 2014, the UK government hosted the first Girl Summit, in partnership with UNICEF, at which they pledged to mobilise domestic and international efforts to stamp out FGM within a generation, launching a raft of initiatives including a £ 1.4 million FGM prevention programme with NHS England, and legislative changes (4). The FGM prevention programme sought to improve how the NHS responds to FGM, including making training about FGM and safeguarding statutory for NHS organisations (5), and sets out expectations for NHS staff, including about data recording (6).

FGM has been a specific offence in the UK since 1985 (7). Before 2019, there had been no UK convictions, a fact described as a "national scandal" in 2014 by a Home Affairs Select Committee reporting on the case for an FGM National Action Plan (with aims included achieving a successful prosecution and improving safeguarding and services)(8). In 2015, a mandatory reporting duty was introduced in England and Wales, requiring all registered professionals to report all cases in under 18 year olds where FGM was identified on examination or through a first-hand disclosure directly to the police (9). Additionally, an FGM Enhanced Dataset was introduced in 2015 in England, mandating the submission of quarterly data returns including personally identifiable data from all GP practices (10). Data return rates from primary care to the Enhanced Dataset have been low, with only 64 GP practices in England submitting data returns in 2018-2019(11) . The reasons for this are not known. Concerns have been raised by clinicians and community members about the potential impacts of mandatory reporting and the Enhanced Dataset on trust and patient-doctor relationships (12-15).

In this realist synthesis we seek to understand factors that can potentially influence how GPs and women and girls from FGM affected communities interact in English primary care, in the current UK context. This was identified as an important area for exploration in a research user consultation where community members and professionals were asked what they identified as FGM research and service priorities (16). General Practice care in England is typically delivered by primary care health teams, including GPs, practice nurses, ANPs and increasingly pharmacists and paramedics, who are based in GP practices located within community settings. Primary care in England holds patients and families holistic and longitudinal care (and care records) and has a gatekeeper role. Prior systematic reviews have shown that around the world, health professionals do not have adequate knowledge about FGM, although these reviews primarily focused on obstetrics and gynaecology (17, 18).

Our exploratory review identified the relevant literature as disparate and heterogeneous. Therefore, we identified a need for bringing together and making sense of different types of evidence that would help develop our understanding of how, why, and under what circumstances FGM is discussed (or not) in primary care consultations in England, in the context of recent policy changes.

To explore this over-arching review aim, we identified the following review questions:

1. What influences how general practitioners (GPs) manage female genital mutilation (FGM) in their clinical practice and why?

For peer review only

2. What influences GPs actions when they consider initiating discussion about female genital mutilation (FGM) with patients in primary care? Where, when and why are these influences active?
3. What influences how GPs respond to a patient-initiated disclosure of FGM during a primary care consultation? Where, when and why are these influences active?

## Methods

For GPs, supporting women with FGM and managing the attendant reporting, safeguarding and clinical needs associated with this can be viewed as a complex intervention (defined by the Medical Research Council (MRC) as an intervention with several interacting components, and where the behaviours required by the intervention are numerous or complex (19). The FGM Prevention Programme included the provision of new educational materials, safeguarding resources and new obligations to document and report FGM. This requires GPs to participate in educational opportunities, consider when and how to discuss FGM with their patients, and consider when and how they need to comply with reporting requirements.

Realist synthesis is a theory driven and interpretive systematic review methodology with an explanatory rather than judgemental focus which can be used to evaluate the impact of complex policy. Adopting a realist synthesis methodological approach, the research question does not only explore whether an intervention works – or not – but explicitly considers under what circumstances (when, why and how) an intervention might generate outcomes. A realist synthesis seeks to explore the contexts under which outcomes occur, and the mechanisms (processes which connect the context and the outcome) which link them (20).

There is little primary research about how English GPs are managing their patients with FGM. The realist review approach as defined by RAMESES allows development of programme theory based on evidence about managing FGM in other health care settings (for example obstetrics and midwifery), from grey literature, including opinion pieces and charity publications, and for testing evolving programme theory in potentially comparable healthcare challenges in English primary care.

We used the RAMESES publication standards to develop (21) and report this realist synthesis (22), and followed methodology described in other realist syntheses (23). The study protocol is available as Appendix A.

**Patient and Public involvement:** This synthesis was developed following a patient and public involvement research priority setting project, which SD was involved in developing and reporting, which identified this question as a research priority (16). The findings of this synthesis were reviewed with stakeholders and PPI collaborators who commented on resonance and relevance.

**Initial programme theory development**: An initial programme theory (a theory describing how and why the interventions being considered are hypothesised to operate to generate outcomes) (22) considering how and under what circumstances GPs in England might talk about FGM with their patients was derived by SD based on an exploratory literature review and relevant policy documentation (Appendix B). SD works as a GP partner and is a practice safeguarding lead. Since 2015, SD has been a primary care member of her local safeguarding board FGM operational group. SD is a trustee of Oxford Against Cutting. This synthesis was developed by SD following a public and stakeholder research priority setting project, which identified service priorities (including the need for holistic services throughout the life-course) and research questions including the potential impacts of Mandatory reporting and the FGM Enhanced Dataset on trust in healthcare. Following the commencement of this literature synthesis, SD developed and began a qualitative study undertaking interviews with GPs about their perspectives on supporting women affected by FGM in the context of English Primary care following the introduction of mandatory reporting and the FGM Enhanced Dataset. An expert advisory group consisting of 6 primary healthcare professionals, including local and national FGM experts, acted as project stakeholders and advised on how the programme theory fitted within primary care processes, a recognised contribution to realist synthesis (24).

**Searching:** Searching for data for inclusion in this review was conducted in multiple stages. Initial exploratory scoping searches identified an initial set of relevant documents which informed the development of the initial programme theory and contributed to theory refinement. The main searches to identify evidence to test our

For peer review only

programme theory were developed by an information specialist and conducted in the following databases: MEDLINE, Embase, PsycINFO, Global Health, CINAHL, Web of Science, Sociological Abstracts, Anthropology Plus, Social Science Abstracts and ASSIA. We searched for literature on health professionals and FGM in the UK and worldwide, in primary care and obstetrics and gynaecology, and we searched for international qualitative studies and reviews on FGM. There was no time limit set for the search. We included other health settings where the reporting and communication requirements might be similar because of the lack of primary care data. We built our dataset iteratively by searching reference lists and conducting forward citation searches for key papers. We included grey literature identified in this way and utilised recommendations from experts in FGM, including systematically searching for reports from English FGM advocacy organisations. We conducted update searches, to ensure that the most recent evidence was included. We also conducted secondary searches in the following bodies of literature, identified as areas where comparable contexts and mechanisms may occur to inform our developing programme theory: intimate partner violence (IPV) in primary care, Driving and vehicle Licensing Agency (DVLA) reporting, the Prevent programme, and mandatory reporting (in health and education). The searches are available in the online supplementary file (Appendix C).

**Selection and appraisal of documents:** We included all study designs and papers that presented evidence about factors potentially relevant to considering how, why and in what circumstances GPs feel able to discuss (or not) FGM with their patients in English primary care settings, following the recent regulatory changes. Where documents from non-UK settings were identified, we excluded those written in languages other than English and from contexts likely to be significantly different from UK primary care. We included opinion pieces in influential UK medical journals as these could provide explanations relevant to UK primary care. All titles and abstracts were screened by SD and were included for full text review if they were judged as potentially containing information relevant to conversations about FGM in primary care. Papers that contained information possibly informative about any part of the process of primary care consultation were considered. Our scoping had shown us that there was no primary evidence that covered all aspects of our review question, and so we identified that to having broad and open criterion would facilitate the development of responsive and wide-ranging programme theory. In keeping with realist methodology, data selection and inclusion were iterative, beginning with our initial programme theory and then re-visited as new areas of theory emerged. For example, as the importance of emotive responses to FGM emerged as an important relevant factor from our initial searching, we re-visited our search for evidence to explore this, as is established in realist synthesis methodology (20). In keeping with realist synthesis methodology, papers were appraised for their relevance (whether the study provided evidence relevant to the theory under development) and rigour (whether the inferences within the evidence make a credible contribution to testing a developing theory). The concept of data saturation is applicable within realist synthesis (20). Together, these methodological parameters made up the study framework which determined inclusion and exclusion. Ten percent of selected abstracts were independently reviewed by GH/CD and discrepancies discussed to inform the remaining screening process. All included full text documents were reviewed and inclusion was agreed with GH/CD/study team.

**Data extraction and analysis:** Papers selected for full text review were loaded into NVivo and coded. We based initial codes on the initial programme theory, adding further emergent codes during analysis. We examined the coded data for patterns (demi-regularities (25)) from which we identified potential contexts, mechanisms and outcomes of interest. These were iteratively examined within the evidence to develop configurations of contexts, mechanisms, and outcomes (CMOC). The expert advisory group commented on the resonance of the developing and finally presented CMOCs. We prioritised high quality empirical data, however we included evidence from other sources if it was relevant to the developing primary care CMOCs (20, 26). The resulting programme theory was developed in realist format depicting the relationship between context, outcome, and the mechanism linking them. Developing theory was tested for resonance by considering it against evidence from comparative literature fields (24)

## Results:

In the searching directly related to FGM (including the scoping, main and update searches), 4035 abstracts were identified and screened, leading to full text review of 346 papers, with an additional 51 papers added for full text review from recommendation, advocacy organisation searching and citation tracking leading to the

For peer review only

final inclusion of 94 papers from the FGM literature. Comparative literature was identified from the secondary searches from UK primary care in IPV, the Prevent strategy, DVLA reporting, and mandated reporting. Reading within the Prevent literature, we identified evidence related to data-sharing in the context of immigration enforcement and this was also included where relevant; these searches identified 593 citations, of which 121 were included for full text review, and 30 informed the synthesis. Figure 1 summarises the search strategy, table 1 summarises the characteristics of the included FGM evidence, and table 2 summarises the characteristics of the included evidence from the comparative literature.

Figure 1:

For peer review only

| Type of literature | Number of papers |
|---|---|
| Systematic/literature review | 12 |
| Qualitative | 32 |
| Quantitative survey/questionnaires | 15 |
| Audit / case series | 9 |
| Charity reports | 9 |
| Other (e.g. editorial opinion piece) | 17 |
| **Country of research origin** | |
| UK | 35 |
| Other European | 21 |
| America / Canada | 5 |
| Australia | 4 |
| Africa | 1 |
| **Whose perspective** | |
| Provider | 29 |
| Community | 28 |
| Both | 10 |
| **Details of providers** | |
| Obstetrics /gynaecology / midwifery | 20 |
| Other secondary care | 11 |
| Mixed including primary care | 6 |
| **Country of origin of women research conducted with/included** | |
| Somalia / infibulated women/type 3 FGM | 23 |
| Mixed African | 12 |

Table 1. Characteristic of the 94 papers making up the FGM literature evidence which contributed to CMOC derivation are detailed below:

For peer review only

| | |
|---|---|
| Mixed | 2 |

Table 2: Characteristics of the 30 papers included from literature identifies in secondary searches:

| **Intimate Partner Violence** | **Number of papers** |
|---|---|
| Primary qualitative | 7 |
| Primary quantitative | 2 |
| Other/opinion/guidance/review/service evaluation | 6 |
| **Mandatory Reporting** | |
| Primary qualitative | 4 |
| Primary quantitative | 4 |
| Other/opinion/guidance/review | 2 |
| **Prevent Strategy/ data sharing** | |
| Primary qualitative | 2 |
| Primary quantitative | |
| Other/opinion/guidance/review | 3 |

## Results:

This section is a narrative account of the final programme theory (explanatory configurations of contexts, mechanisms and outcomes) developed by this synthesis considering how, why and under what circumstances GPs might initiate (or not) or respond to FGM (or not) in the setting of English primary care. A full set of CMOCs with supporting data is available as a supplementary file (Appendix D). The programme theory is presented within four themes: FGM knowledge and awareness, communication about FGM, the role of guidelines and service provision, and mandatory reporting requirements. The over-arching realist programme theory is illustrated graphically in figures 2 and 3.

## FGM knowledge and awareness:

Health professionals need to have adequate knowledge about FGM to meet the care needs of patients who may be affected by FGM (including having had FGM or potentially being at risk of FGM) (17, 18, 27-47). Awareness of who might be affected by FGM, and the different types of FGM and their associated clinical consequences, will influences whether GPs identify a need to consider or ask about FGM with their patients. Health professionals also need to know about relevant legislation, and their statutory and safeguarding requirements in relation to FGM(17,18,34, 38,41,47,48,49,)

Whether GPs are aware (or not) of what they may not know about FGM, lacking knowledge about FGM affects practitioners' ability and confidence when caring for women with FGM (36, 50, 51), including confidence to

consider who may be at risk (52). Knowing how to respond to a disclosure or when identifying that a woman has FGM may help GPs feel confident to ask (53, 54). In turn, women who perceive that GPs do not have the knowledge or skills to recognise their FGM related care needs, or who feel potentially stigmatised because of their FGM, may lack confidence in accessing healthcare (31, 36, 50, 55-59).

Health professionals report experiencing strong emotional reactions to encountering FGM, including anger, shock and pity (48, 60-62), and that seeing FGM without having adequate knowledge can be "frightening" (61). Experiencing these strong emotional responses may contribute to clinicians feeling panicked, and abandoning usual practices and routines (48,54 ). Although professionals describe trying to hide their reactions, they were aware that they may be apparent to the women (47). This observation is mirrored by evidence from community members who describe feeling ashamed or judged when health care professionals react with shock or horror to their FGM, notably during physical examinations (55,63-66). This can impact on their willingness to access services (46, 63-65, 67), including attending for cervical smears (68). This could be mitigated against when professionals were able to act with confidence and sensitivity (54, 66, 69).

Health professionals with experience of supporting patients with FGM were likely to have more knowledge about FGM (34, 38, 40, 70). A potential challenge for GPs is that FGM may form only a small part of their workload (71, 72), meaning that learning about FGM may not be identified as a priority. Another potential difficulty is that FGM can be hard to identify (73). Recent data from a specialist paediatric clinic in England noted that the examination signs in type 4 can be subtle, and potentially difficult to identify (74-76) and others have noted that not all GPs will have the necessary expertise to identify all types of FGM (12, 77). Added to this complexity is that self-reporting by women about their FGM type in health settings has been shown not to be reliable (78), which could impact on how women feel able to respond to some questions about FGM.

**Talking about FGM and communication:**

A key skill GPs need is being able to talk about FGM sensitively (36, 45, 79). GPs who do not feel confident in raising the subject of FGM, for example because they are worried that they will upset or offend women, may avoid talking about FGM (14, 30, 31, 42, 46, 47, 49,55, 61, 73, , 80). Health professionals who perceived that discussing FGM can be culturally taboo or a sensitive subject could also be fearful of offending women reported sometimes avoiding talking about FGM (14, 47, 55, 79, 81, 82), described in one study as an "expression of respect"(79). This contextual factor may be evolving as community attitudes towards talking about FGM are shifting, meaning that talking about FGM is becoming less taboo in some communities (48, 83-87). Lacking awareness of shifting community attitudes can risk offending or alienating members of those communities (48, 84) which risks reducing effective communication.

The words or terminology that GPs use when they talk about FGM can complicate communication with their patients. For example, if the term FGM is not familiar to the woman (88), offends or alarms her (89, 90), or she does not align her cultural practice (e.g. labial elongation) with FGM (91), then she may not relate her experience to FGM. This can complicate conversations about the potential health consequences of FGM or whether women perceive the conversation is relevant to their experience of FGM (92).

Women who were aware of previous difficult experiences with communication and engagement with health professionals (e.g. language, cultural differences, perceived judgement) where they had  not felt understood or respected describe a lack of confidence and trust in health services (45, 55-57, 63, 90, 93). Women who feel pitied or judged may be less likely to feel able to make a disclosure to healthcare professionals (94, 95). Language barriers or a lack of understanding about how health services work, including negotiating with primary care reception staff can complicate access to healthcare (96).

A potential strategy that could help facilitate both the acceptability and accessibility of services is the involvement of community health advocates, such as members of FGM affected communities, who can act as a bridge between communities and services, for example by promoting trust and providing education for both community members and health professionals (16, 97, 98).

For GPs, whether FGM is raised by the patient or the GP, an important contextual factor is whether FGM is perceived to be relevant to the health concern which the woman brings to her GP appointment. This can

For peer review only

influence both whether the subject of FGM is broached, and then if broached, how it is received and experienced (14, 62, 99). Women who feel that the healthcare professional is pre-occupied with their FGM, rather than their health concerns may disengage from the healthcare setting (83, 100, 101). This is also potentially relevant when GPs consider asking women about their experience of FGM with the aim of considering the safeguarding needs within their families, rather than the woman's own health needs (48, 102, 103). Balancing the needs of the woman who is presenting with the potential needs of her wider family may introduce complex considerations for GPs when they are considering how and into whose medical notes they code FGM into primary care medical records (103-105).

An important context which influences how able (and enabled) GPs and their patients are to effectively communicate about FGM is whether there is a language barrier between them, or not (14, 18, 30, 45, 47, 55, 80, 81, 96, 106-108) . Strategies to address language barriers add their own complications. Official interpreters are recommended, but may not be available or trusted by women, for example if they both perceive FGM as taboo, or she fears they will not respect her confidentiality. This can lead to fear and reduced engagement with health professionals (18, 30, 32, 47, 49, 62, 68, , 109, 110). The presence of family members (as interpreters, or witnesses) in the consultation may inhibit GPs from feeling able to raise FGM with the women, because of concerns about privacy and confidentiality (14, 30, 111).

Finally factors such as the GPs gender may influence whether the woman or GP feel it is culturally appropriate to talk about FGM (14, 47, 68, 106). Time constraints in the consultation may act as a potential barrier which deters GPs when they are contemplating discussing FGM (14, 53, 72, 112).

**The need for guidelines and access to specialist services:**

Researchers and commentators suggest that having access to clear guidelines will enable professionals to ask women about FGM and optimise their care (17, 44, 61, 113, 114). However even when guidelines exist, awareness of them may be incomplete, or they may not be followed, as demonstrated by four UK hospital studies (49, 70, , 80, 115). Access to guidance may be especially important for clinicians who see FGM less often (47). Lacking guidance, including a lack of certainty of what "good care" comprises can lead clinicians to feel unsure, and improvise how they offer care (46, 47), and risks incorrect decision making (113, 114).

Normalising asking about FGM, for example by using prompts in the medical record may overcome some clinician barriers and help them begin these discussions (116), especially if these are then linked to resources or care pathways (117).

Having access to services which could offer specialist assessments, treatment or advice may help GPs feel enabled to raise the subject of FGM (12, 55, 57, 77, 118). When health professionals speak about FGM within a framework of offering support and services, it is more likely to be experienced as acceptable by their patients (87). When training and education are supported by referral pathways or protocols for intervention, they are more likely to be effective in changing behaviour and promoting clinicians asking (54, 94, 117, 119).

**Mandated actions including The Mandatory Reporting Duty and The FGM Enhanced Dataset requirements:**

It is unknown what impact the FGM reporting duties have on healthcare interactions, but concerns have been raised that the Mandatory Reporting Duty in FGM may cause women distress (120), or reduce their trust in healthcare professionals, which may deter women from seeking healthcare or disclosing their FGM to healthcare professionals (12-14, 83, 121). Furthermore, if women perceive that the healthcare professional is more interested in data management about their FGM than their needs, they may feel disrespected and avoid healthcare settings (83).

The requirement to send personally identifiable data to the FGM Enhanced Dataset was also identified as a potential barrier to talking about FGM, by both women and GPs (14-16, 122, 123). When women are not

For peer review only - http://bmjopen.bmj.com/site/about/guidelines.xhtml

confident that their medical encounters or records are confidential, they may feel fearful and avoid making disclosures (111, 124).

The concern that making a mandated report would have a negative impact on on-going effective doctor-patient relationships may be an important consideration for professionals (125-129), including whether this might deter patients from accessing services (130). Lacking confidence that making a mandated report will be met with an acceptable or adequate response may pose challenges for healthcare professionals (128, 131).

Practitioners may need to feel certain before making a mandated report (132, 133), and challenges in identifying less apparent forms of FGM may add tensions to the requirements for mandated reporting (128). In the case of FGM, this could be complicated if GPs do not feel confident that they have the knowledge or skills to correctly identify FGM (72, 90), especially type 1 and 4 which may be harder to visualise on examination and more commonly encountered (74, 134). Mandatory reporting by health professionals should be supported by educational resources (135); reporting without training may lead to inaccurate data collection (136) Clinician concerns about confidentiality and stigma could also contribute to incomplete or inaccurate data coding (137, 138).

When young people know that the professional whom they are speaking to is mandated to share the information with other authorities, they may feel more reluctant to trust the professional, and less likely to make a disclosure (139). Those who are potentially fearful of authorities or perceive themselves to be vulnerable, for example those with uncertain migrant status, may be more fearful of mandatory reporting or data sharing and avoid accessing services (139-143).

**Over-arching programme theory:**

Figures 2 and 3 bring together the emerging CMOC configurations that made up the programme theory for this review and depicts a theoretical framework for how these might inter-relate, thus offering a conceptual summary of this synthesis.

Figure2:

Figure3:

**Discussion:**

**Summary:**

GPs need adequate knowledge to support their patients with FGM including the different FGM types and their different clinical presentations, needs, and cultural contexts. This includes needing to be aware of local legislative, statutory, and safeguarding requirements. GPs need skills to discuss FGM sensitively and with appropriate terminology. Language barriers can complicate conversations about FGM. Access to official interpreters is recommended but they may not always be available. Even when available, there are potential pitfalls which GPs should be aware of, including consideration of who else is present in the consultation. The requirements of mandatory reporting and the FGM Enhanced Dataset may bring additional complications into the primary care consultation. Community health advocates could have a role in facilitating access to services.

**Strengths and Limitations:**

As illustrated by table 1, only limited evidence was directly relevant to primary care. GPs have a vital role in managing FGM, yet there is little evidence about their attitudes, knowledge or behaviour towards managing FGM in primary care, and none in the context of the 2015 policy changes. This synthesis therefore utilises evidence from provider experiences in other healthcare settings, predominantly specialist clinics, and obstetrics and gynaecology services. Some challenges are likely to be comparable between these settings and primary care, namely needing adequate knowledge and managing challenges with language and communication. However, there are differences between primary and secondary care that may limit this

For peer review only - http://bmjopen.bmj.com/site/about/guidelines.xhtml

extrapolation, for example that in obstetrics, FGM will almost always be relevant to the woman's reason for attendance, which is not the case in general practice. To address this lack of direct evidence we have also undertaken a primary qualitative study with GPs (Dixon et al. BJGP, in press). The lack of primary data about GPs necessitated complex searches and we may have inadvertently not identified important evidence.

We identified limited evidence on the experiences and needs of women from outside of Africa and with forms of FGM other than type 3. Given the evidence that these types may not be those that GPs most commonly encounter, this is a potentially significant limitation. Whether these findings can be extrapolated to inform healthcare for women with different FGM types or from other FGM practicing countries (for example Malaysia or Indonesia) is unclear.

The GP consultation represents a coming together of GP and patient, and a strength of this synthesis is that it explores whether GPs talk about FGM using experiential evidence from both perspectives. Realist methodology allowed us to generate explanatory programme theory relevant to GPs from evidence on managing FGM in other healthcare settings and within other healthcare contexts, despite the lack of primary care data. Realist methodology supports the inclusion of grey literature. This project has benefitted from information on English community and GP perspectives reported by FGM advocacy organisations, which may not have been included in a traditional systematic review. Using iterative searching and citation tracking maximised data inclusion.

**Comparison with existing literature:**

The FGM literature is predominantly descriptive. This helps define potential challenges but offers less evidence about effective interventions with which to address them.

There is a rich literature about FGM, including a number of previous systematic reviews and qualitative meta-syntheses, including a number cited within this synthesis as contributing evidence, perspectives and commentary relevant to this synthesis question and research objectives (17,18,43,44,45,46,47,64,101). This review includes other syntheses which included grey literature (47) and evidence syntheses that included both provider and community perspectives (45,46). In addition to their review of healthcare provider perspectives, Evans et al have undertaken a comprehensive systematic qualitative synthesis of women affected by FGMs experiences of healthcare. This wide-ranging review included grey literature, and considered experiences across all healthcare settings, including primary care. Their model of culturally safe care resonates with the realist theory we propose in this paper, as do their findings including the importance (and potential challenges) of effective communication, the value of positive encounters characterised by knowledge and compassion, and the adverse impacts on women when healthcare professionals reacted to their FGM in ways which made them feel ashamed or judged (144).

This synthesis can add to this important developing body of literature by contributing a realist perspective, thus considering contextual and generative mechanisms across a range of processes potentially relevant to communication within the context of a primary care consultation. Utilising grey literature and opinion pieces and bringing together evidence from provider and community perspectives allowed us to triangulate possible influential factors. Finally, the realist methodology supported the inclusion of evidence from other research fields. This allowed us to postulate theory where there was no directly available evidence. For example, Evans et al noted in their synthesis of factors relevant to healthcare provision of FGM healthcare from the perspectives of healthcare providers that there was no evidence about the delivery of safeguarding care (47). Utilising insights from evidence about intimate partner violence offered potential evidence to test our tentative CMOC with and support their development, for example about what helps clinicians in responding to disclosures (53,54), and about the tensions experienced by GPs in their role of coding into the longitudinal patient care record (103-105). This synthesis adds consideration of the potential impacts of mandated reporting and data sharing on communication in primary care consultations.

In this synthesis, we are presenting postulated programme theory about any factors that might be relevant in the dynamics of the primary care setting, which we hope will be further tested, appraised, and improved. Indeed, since we submitted this synthesis, a qualitative study exploring women with FGM's experiences of primary care in the Netherlands has been published, which is a welcome addition to the available literature.

For peer review only - http://bmjopen.bmj.com/site/about/guidelines.xhtml

This study reported women's perceptions of challenges when seeing a GP, including concerns that their GP would not have adequate knowledge about FGM, or be able to help them, and concerns about limited time in appointments. Satisfaction was increased when GPs were able to convey that they understood and were supportive of their care needs (145). We consider that these findings resonate with the theory postulated in this synthesis.

In their realist synthesis considering the experiences of UK maternity care by women with social risk factors, which included consideration of women with FGM, Rayment-Jones et al also identified the importance of perceptions of kindness and respectfulness from healthcare professionals, the value of trusting relationships and potential role for health advocates, and how factors such as language and access to interpreters contribute to the concept of candidacy for care (146). This review also identified that fear of judgement and perceptions that health services role is surveillance rather than care as an important contextual factor relevant to accessing care. In their systematic review considering challenges and facilitators for refugees and asylum seekers in high income countries, Robertshaw et al also identified the importance of trusting relationships, acceptable and accessible interpreters where there were language barriers, and the importance of cultural competency in primary care, and the need for education and training to support this (147). A 2020 qualitative study exploring English Health Care Providers perspectives on the impacts of data sharing for immigration enforcement reported concerns about impacts on health access, patient/clinician relationships, and about the interface between policy and their professional ethics, notably confidentiality and trust(148).

The lack of (and need for) an evaluation of Mandatory Reporting has been commented on by other authors (149).

We have identified deficiencies in professional knowledge as an important contextual factor that can influence whether GPs talk about FGM. Other authors have noted the need for those in primary care to be better informed to improve care for those affected by FGM (139). One study with eleven midwives evaluated the effectiveness of an educational intervention and found that it was successful in promoting knowledge and confidence in managing FGM (150). This intervention used case studies, which have been suggested by community advocates for FGM education (14, 55). A 2016 survey of medical students from five medical schools in London reported that the majority of respondents had not received formal teaching on FGM and were not aware of potential associated health issues. Having had formal education increased awareness, but despite this, only 50% of respondents who had been formally educated felt confident about identifying FGM on examination (151). UK medical students have voted to ask for education about FGM. Medical students reported that after attending a workshop which included education on FGM, the UK law, and how to talk about FGM, that 75% of them felt more confident about communicating with a patient who had had FGM (152). There is a call for FGM education to promote professionals' cultural competency (18, 29, 33, 153). Cultural competency education for healthcare professionals has benefits (154), including for patients (155), although formal cultural competency training is often lacking in general practice (156). We have not identified any literature evaluating the impact of FGM education for primary care practitioners on their clinical confidence or cultural competencies. A systematic review conducted in 2019 by 3 authors of this review (GH, SD, FG) found that, despite increases in FGM awareness in both healthcare and public spheres, HCPs remained subjectively and objectively under-educated and underprepared on the issue. Whilst isolated countries, such as Sweden, had managed to target their education effectively, the majority (including high prevalence nations) struggled to approach FGM education and training adequately. Much of this stemmed from issues of cultural competency and confidence in knowledge, as is reflected in this study (157).

That it can be important for health professionals to manage their own emotional reactions when they are supporting patients affected by FGM is resonant with research into intimate partner violence, which tells us that clinician responses including blaming, judging or pitying should be avoided (94).

In tabulating the available evidence relevant to our synthesis question, we note that the available FGM evidence is predominantly from obstetric settings, with a lack of evidence from other settings, notably primary care, an important point documented in other systematic reviews (17,47)The holistic life-course health needs for women with FGM, including their FGM related needs outside of safeguarding, paediatric, or obstetric settings, are important service and research needs (16, 158). We also note that much of the identified research considers the needs and experience of women with type 3 FGM, yet an English specialist paediatric clinic most commonly identified type 4 FGM and no cases of infibulation and identified a girl from Malaysia with FGM

(74). That the existing evidence is potentially skewed towards type 3 FGM has been documented previously in a systematic review of healthcare providers experiences of caring for women with FGM (47). It is important that clinicians are aware of the practice of FGM in some Asian countries (including Indonesia and Malaysia) although there is little evidence about prevalence to guide them (74).

**Implications for research and practice:**

This research will help GPs (and GP educators) consider what knowledge or skills are needed to support GPs to feel confident to talk about FGM with their patients. It may help them consider the challenges when using interpreters to talk about FGM and highlights the potential challenges of managing the FGM reporting requirements.

Research is needed to explore what FGM-affected communities need from GPs and primary care. This needs to include all types of FGM, and all communities that practice FGM, and all aspects of access to and experiences of care, whether directly related to FGM or not.

There is a need to research and understand FGM related health needs throughout the life-course, including for example the needs of women living with FGM throughout and after their menopause. That there is no research considering the experiences of FGM safeguarding is an important observation which warrants further research. Expert commentators have noted the lack of evidence under-pinning some of the guidance that clinicians are offered about what signs they should look for to try to prevent or anticipate FGM (74). Clinicians urgently need evidence that will help them protect girls and families, including consideration about what policies and strategies are acceptable and effective within communities. We need evidence to inform this and believe that this needs to be developed in partnership with community members and front-line clinicians.

The effectiveness of policies and legislation on deterring community practice and working towards eliminating the practice of FGM are vital considerations but which were beyond the scope of this review, which focussed on their impacts on communication in consultation. However, the potential impacts of the mandatory reporting and FGM Enhanced dataset requirements on healthcare interactions does need to be evaluated, including GP and community perspectives.

Educational interventions, research, and services should be developed in partnership with community members, utilising their expertise and experience, to ensure resources meet their needs.

Figure 1 summarises our searching and screening processes, while Tables 1 and 2 summarise the characteristics of the papers included in the synthesis

Figure 2: Conceptual depiction of factors that may influence when GPs may or may not talk about FGM with their patients

Figure 3: Conceptual diagram illustrating the over-arching programme theory for this synthesis:

Funding Declaration: Sharon Dixon was funded by a National Institute for Health Research (NIHR) In-Practice fellowship (IPF-16-10-03, 2016-2018) during this research project. The views expressed are those of the author(s) and not necessarily those of the NIHR or the Department of Health and Social Care.

Claire Duddy is supported by an National Institute of Health Research (NIHR) Research Methods Programme Systematic Review Fellowship (NIHR-RM-SR-2017-08-018)

w only

Professor Sue Ziebland is an NIHR Senior Investigator

Competing Interests Statement: Sharon Dixon is a trustee of Oxford Against Cutting. Following this project, she has acted as the RCGP college representative for FGM. Sharon Dixon has held small grants to develop and report on the patient and public involvement project that under-pinned this research.

There are no other author competing interests to declare.

Author Contributions:

Sharon Dixon and Frances Griffiths wrote and developed the study protocol, the initial programme theory,  and conducted the data analysis and synthesis.

Sue Ziebland contributed to the study design and development and contributed writing and data presentation advice and expertise and contributed to analysis and theory development.

Chrysanthi Papoutsi provided methodological guidance and advice.

Claire Duddy and Gabrielle Harrison read and agreed on abstracts and data for final inclusion.

Claire Duddy assisted with study searching and data management.

All authors contributed to the development and writing of this manuscript

Data Availability statement:  For this realist review we analysed published literature that is in the public domain

For peer review only

References

1. WHO. Female genital mutilation Fact Sheet. World Health Organization; 2017 2017-02-07 11:25:18.
2. UNICEF. Female genital mutilation/cutting: A global concern - UNICEF DATA. New York: United Nations Children's Fund; 2016 2016-02-03.
3. Macfarlane A, Dorkenoo E. Prevalence of Female Genital Mutilation

in England and Wales:

National and local estimates. 2015.
4. Girl Summit 2014 - GOV.UK [press release]. 2019.
5. England N. NHS England » Female genital mutilation – care and prevention 2019 [Available from: https://www.england.nhs.uk/ourwork/safeguarding/our-work/fgm/.
6. England N. NHS England » Female Genital Mutilation Prevention Programme: Requirements for NHS staff 2017 [Available from: https://www.england.nhs.uk/2014/12/fgm-prevention/.
7. Female Genital Mutilation Prosecution Guidance | The Crown Prosecution Service: s of information; 2019 [Available from: https://www.cps.gov.uk/legal-guidance/female-genital-mutilation-prosecution-guidance.
8. Home Affairs Committee HoC. Female genital mutilation: the case for a national action plan Second Report of Session 2014–15. London: the House of Commons; 2014.
9. Government HM. Mandatory reporting of female genital mutilation: procedural information. In: Office TH, editor. London: HMSO; 2015.
10. Department of Health H. FGM Prevention Programme. Understanding the FGM Enhanced dataset – updated guidance and clarification to support implementation. In: Health Do, editor. London: The Stationary Office; 2015.
11. Female Genital Mutilation (FGM) Enhanced Dataset

April 2018 to March 2019, England, experimental statistics, Annual Report NHS Digital: HSCIC; 2019 [27.9.2019]. Available from: https://digital.nhs.uk/data-and-information/publications/statistical/female-genital-mutilation/april-2018---march-2019.
12. Mathers N, Rymer J. Mandatory reporting of female genital mutilation by healthcare professionals. British Journal of General Practice. 2015;65(635):282-3.
13. Plugge E, Adam S, El Hindi L, Gitau J, Shodunke N, Mohamed-Ahmed O. The prevention of female genital mutilation in England: what can be done? Journal of public health (Oxford, England). 2018;fdy128.
14. Clayton-Hathaway K. A pilot evaluation of health services for communities affected by FGM/C in Oxfordshire. Oxfordshire: Oxford Against Cutting

Healthwatch Oxfordshire; 2016.
15. Bewley S, Kelly B, Darke K, Erskine K, Gerada C, Lohr P, et al. Mandatory submission of patient identifiable information to third parties: FGM now, what next? BMJ : British Medical Journal. 2015;351:h5146.
16. Dixon S, Agha K, Ali F, El Hindi L, Kelly B, Locock L, et al. Female genital mutilation in the UK-where are we, where do we go next? Involving communities in setting the research agenda. Research Involvement and Engagement. 2018;4(29).
17. Zurynski Y, Sureshkumar P, Phu A, Elliott E. Female genital mutilation and cutting: a systematic literature review of health professionals' knowledge, attitudes and clinical practice. BMC international health and human rights. 2015;15:32.
18. Dawson A, Homer CS, Turkmani S, Black K, Varol N. A systematic review of doctors' experiences and needs to support the care of women with female genital mutilation. International Journal of Gynecology & Obstetrics. 2015;131(1):35-40.

For peer review only

19. Craig P, Dieppe P, Macintyre S, Michie S, Nazareth I, Petticrew M. Developing and evaluating complex interventions: the new Medical Research Council guidance. 2008.
20. Pawson R, Greenhalgh T, Harvey G, Walshe K. Realist review--a new method of systematic review designed for complex policy interventions. Journal of health services research & policy. 2005;10 Suppl 1:21-34.
21. Wong G, Greenhalgh T, Westhorp G, Pawson R. Development of methodological guidance, publication standards and training materials for realist and meta-narrative reviews: the RAMESES (Realist And Meta-narrative Evidence Syntheses – Evolving Standards) project. 2014.
22. Wong G, Greenhalgh T, Westhorp G, Buckingham J, Pawson R. RAMESES publication standards: realist syntheses. BMC medicine. 2013;11:21.
23. Papoutsi C, Nuffield Department of Primary Care Health Sciences UoO, Radcliffe Observatory Quarter, Woodstock Road, Oxford OX2 6GG, UK, Mattick K, Centre for Research in Professional Learning UoE, St Luke's Campus, Exeter EX1 2LU, UK, Pearson M, National Institute for Health Research (NIHR) Collaboration for Leadership in Applied Health Research and Care South West Peninsula IoHR, University of Exeter Medical School, South Cloisters, St Luke's Campus, Exeter EX1 2LU, UK, et al. Social and professional influences on antimicrobial prescribing for doctors-in-training: a realist review. Journal of Antimicrobial Chemotherapy. 2017;72(9):2418-30.
24. Pawson R, Greenhalgh T, Harvey G, Walshe K. Realist synthesis: an introduction. University of Manchester: ESRC Research Methods Programme; 2004.
25. Jagosh J, Pluye P, Wong G, argo M, Salsberg J, Bush PL, et al. Critical reflections on realist review: insights from customizing the methodology to the needs of participatory research assessment. *Research synthesis methods*. 2014;*5* (2):pp.131-41.
26. Pawson R. Digging for Nuggets: How 'Bad' Research Can Yield 'Good' Evidence. http://dxdoiorg/101080/13645570600595314. 2007.
27. Leval A, Widmark C, Tishelman C, Ahlberg BM. THE ENCOUNTERS THAT RUPTURE THE MYTH: CONTRADICTIONS IN MIDWIVES' DESCRIPTIONS AND EXPLANATIONS OF CIRCUMCISED WOMEN IMMIGRANTS' SEXUALITY. Health Care for Women International. 2010;25(8):743-60.
28. Khaja K, Lay K, Boys S. Female Circumcision: Toward an Inclusive Practice of Care. Health Care for Women International. 2010;31(8):686-99.
29. Odemerho BI, Baier M. Female Genital Cutting and the Need for Culturally Competent Communication. The Journal for Nurse Practitioners. 2012;8(6):452-7.
30. Lazar JN, Johnson-Agbakwu CE, Davis OI, Shipp MPL. Providers' Perceptions of Challenges in Obstetrical Care for Somali Women. Obstetrics and Gynecology International. 2013;2013:149640.
31. Johnson-Agbakwu CE, Helm T, Killawi A, Padela AI. Perceptions of obstetrical interventions and female genital cutting: insights of men in a Somali refugee community. Ethnicity and Health. 2014;19(4):440-57.
32. Byrskog U, Olsson P, Essén B, Allvin M-K. Being a bridge: Swedish antenatal care midwives' encounters with Somali-born women and questions of violence; a qualitative study. BMC Pregnancy and Childbirth. 2015;15(1).
33. Varol N, Hall JJ, Black K, Turkmani S, Dawson A. Evidence-based policy responses to strengthen health, community and legislative systems that care for women in Australia with female genital mutilation / cutting. Reproductive Health. 2017;14(1):63.
34. Tantet C, Aupiais C, Bourdon M, Sorge F, Pagès A, Levy D, et al. Female genital mutilation: an evaluation of the knowledge of French general and specialized travel medicine practitioners. Journal of Travel Medicine. 2018;25(1):tax090.
35. González-Timoneda A, Ros VR, González-Timoneda M, Sánchez AC. Knowledge, attitudes and practices of primary healthcare professionals to female genital mutilation in Valencia, Spain: are we ready for this challenge? BMC health services research. 2018;18(1):579.
36. Vangen S, Johansen REB, Sundby J, Træen B, Stray-Pedersen B. Qualitative study of perinatal care experiences among Somali women and local health care professionals in Norway. European Journal of Obstetrics & Gynecology and Reproductive Biology. 2004;112(1):29-35.

For peer review only

37. Tamaddon L, Johnsdotter S, Liljestrand J, Essén B. Swedish Health Care Providers' Experience and Knowledge of Female Genital Cutting. Health Care for Women International. 2006;27(8):709-22.
38. Kaplan-Marcusan A, Torán-Monserrat P, Moreno-Navarro J, Fàbregas MJC, Muñoz-Ortiz L. Perception of primary health professionals about Female Genital Mutilation: from healthcare to intercultural competence. BMC Health Services Research. 2009;9(11).
39. Leye E, Ysebaert I, Deblonde J, Claeys P, Vermeulen G, Jacquemyn Y, et al. Female genital mutilation: Knowledge, attitudes and practices of Flemish gynaecologists. The European Journal of Contraception and Reproductive Health Care. 2009;13(2):182-90.
40. Zaidi N, Khalil A, Roberts C, Browne M. Knowledge of female genital mutilation among healthcare professionals. Journal of Obstetrics and Gynecology. 2009;27(2):161-4.
41. Relph S, Inamdar R, Singh H, Yoong W. Female genital mutilation/cutting: knowledge, attitude and training of health professionals in inner city London. European Journal of Obstetrics & Gynecology and Reproductive Biology. 2013;168(2):195-8.
42. Cappon S, L'Ecluse C, Clays E, Tency I, Leye E. Female genital mutilation: Knowledge, attitude and practices of Flemish midwives. Midwifery. 2015;31(3):e29-e35.
43. Dawson A, Turkmani S, Fray S, Nanayakkara S, Varol N, Homer C. Evidence to inform education, training and supportive work environments for midwives involved in the care of women with female genital mutilation: A review of global experience. Midwifery. 2015;31(1):229-38.
44. Reig-Alcaraz M, Siles-González J, Solano-Ruiz C. A mixed-method synthesis of knowledge, experiences and attitudes of health professionals to Female Genital Mutilation. Journal of Advanced Nursing. 2015;72(2):245-60.
45. Smith H, Stein K. Health information interventions for female genital mutilation. International Journal of Gynecology & Obstetrics. 2017;136(S1):79-82.
46. Jordal M, Wahlberg A. Challenges in providing quality care for women with female genital cutting in Sweden–A literature review. Sexual & Reproductive Healthcare. 2018;17:91-6.
47. Evans C, Tweheyo R, McGarry J, Eldridge J, Albert J, Nkoyo V, et al. Crossing cultural divides: A qualitative systematic review of factors influencing the provision of healthcare related to female genital mutilation from the perspective of health professionals. PloS one. 2019;14(3):e0211829.
48. Johnsdotter S, Essén B. Cultural change after migration: Circumcision of girls in Western migrant communities. Best Practice & Research Clinical Obstetrics & Gynaecology. 2016;32:15-25.
49. Zenner N, Liao LM, Richens Y, Creighton SM. Quality of obstetric and midwifery care for pregnant women who have undergone female genital mutilation. Journal of Obstetrics and Gynecology. 2013;33(5):459-62.
50. Dawson AJ, Turkmani S, Varol N, Nanayakkara S, Sullivan E, Homer CS. Midwives' experiences of caring for women with female genital mutilation: Insights and ways forward for practice in Australia. Women and Birth. 2015;28(3):207-14.
51. Nash E, Ranka P, editors. FEMALE GENITAL MUTILATION: KNOWLEDGE, CONFIDENCE, AND APPROACH TO CARE IN CLINICAL PRACTICE OF MIDWIVES AND NURSES IN THE UK. 21st FIGO World Congress of Gynecology and Obstetrics; 2015; Vancouver, BC Canada: International Journal of Gynecology and Obstetrics.
52. Ramsay J, Rutterford C, Gregory A, Dunne D, Eldridge S, Sharp D, et al. Domestic violence: knowledge, attitudes, and clinical practice of selected UK primary healthcare clinicians. British Journal of General Practice. 2012;62(602):e647-e55.
53. Hegarty K, Taft A, Feder G. Violence between intimate partners: working with the whole family. BMJ : British Medical Journal. 2008;337:a839.
54. Sundborg E, Törnkvist L, Saleh-Stattin N, Wändell P, Hylander I. To ask, or not to ask: the hesitation process described by district nurses encountering women exposed to intimate partner violence. Journal of Clinical Nursing. 2017;26(15-16):2256-65.
55. Norman K, Hemmings J, Hussein E, Otoo-Oyortey N. FGM is always with us: Experiences, Perceptions and Beliefs of Women Affected by Female Genital Mutilation in London. Results from a PEER study London: Options Consultancy Services Ltd FORWARD; 2009.

For peer review only

56. Hussein E. Women's Experiences, Perceptions and Attitudes of Female Genital Mutilation: The Bristol PEER study FORWARD; 2010.
57. Glover J, Liebling H, Barrett H, Goodman S. The psychological and social impact of female genital mutilation: A holistic conceptual framework. Journal of International Studies. 2017;10(2):219-38.
58. Norman K, Belay Gegzabher S, Otoo-Oyortey N. “Between Two Cultures”: A Rapid PEER Study Exploring Migrant Communities’ Views on Female Genital Mutilation in Essex and Norfolk, UK. London: National FGM Centre

FORWARD; 2016.
59. Mbanya VN, Gele AA, Diaz E, Kumar B. Health care-seeking patterns for female genital mutilation/cutting among young Somalis in Norway. BMC Public Health. 2018;18(1):517.
60. Ogunsiji O. Female Genital Mutilation (FGM): Australian Midwives’ Knowledge and Attitudes. Health Care for Women International. 2015;36(11):1179-93.
61. Widmark C, Tishelman C, Ahlberg BM. A study of Swedish midwives' encounters with infibulated African women in Sweden. Midwifery. 2002;18(2):113-25.
62. Horowitz CR, Jackson JC. Female “circumcision” African women confront American medicine. Journal of General Internal Medicine. 1997;12(8):491-9.
.
63 Oguntoye S, Otoo-Oyortey N, Hemmings J, Norman K, Hussein E. “FGM is with us Everyday”: Women and Girls Speak out about Female Genital Mutilation in the UK. World Academy of Science, Engineering and Technology. 2009;54:1020-5.
64. Hamid A, Grace KT, Warren N. A Meta-Synthesis of the Birth Experiences of African Immigrant Women Affected by Female Genital Cutting. Journal of Midwifery & Women's Health. 2018;63(2):185-95.
65. Recchia N, McGarry J. “Don’t judge me”: narratives of living with FGM. International Journal of Human Rights in Healthcare. 2017;10(1):4-13.
66 Vloeberghs E, van der Kwaak A, Knipscheer J, van den Muijsenbergh M. Coping and chronic psychosocial consequences of female genital mutilation in the Netherlands. Ethnicity and Health. 2013;17(6):677-95.
67. del Mar Pastor-Bravo M, Almansa-Martínez P, Jiménez-Ruiz I. Living with mutilation: A qualitative study on the consequences of female genital mutilation in women's health and the healthcare system in Spain. Midwifery. 2018;66:119-26.
68. Abdullahi A, Copping J, Kessel A, Luck M, Bonell C. Cervical screening: Perceptions and barriers to uptake among Somali women in Camden. Public Health. 2009;123(10):680-5.
.
69. Moxey JM, Jones LL. A qualitative study exploring how Somali women exposed to female genital mutilation experience and perceive antenatal and intrapartum care in England. BMJ Open. 2016;6(1):e0009846.
70. Purchase TC, Lamoudi M, Colman S, Allen S, Latthe P, Jolly K. A survey on knowledge of female genital mutilation guidelines. Acta Obstetricia et Gynecologica Scandinavica. 2013;92(7):858-61.
71. Baillot H, Murray N, Connelly E, Howard N. Addressing female genital mutilation in Europe: a scoping review of approaches to participation, prevention, protection, and provision of services. International Journal for Equity in Health. 2018;17(1):21.
72. Baillot H, Murray N, Connelly E, Howard N. Tackling Female Genital Mutilation in Scotland: A Scottish Model of Intervention. Scotland: Scottish Refugee Council

London School of Hygiene and Tropical Medicine 2014.
73. Abdulcadir J, Dugerdil A, Boulvain M, Yaron M, Margairaz C, Irion O, et al. Missed opportunities for diagnosis of female genital mutilation. International Journal of Gynecology & Obstetrics. 2014;125(3):256-60.

For peer review only

74. Hodes D, Armitage A, Robinson K, Creighton SM. Female genital mutilation in children presenting to a London safeguarding clinic: a case series. Archives of Disease in Childhood. 2016;101(3):212-6.
75. Hodes D, Armitage A, Dykes A, editors. Female genital mutilation in London and the UNICEF report; a local perspective on worldwide statistics. Annual Conference of the Royal College of Paediatrics and Child Health, RCPCH 2014; 2014 2014-04-01; Birmingham, UK: Archives of Disease in Childhood.
76. Ayadi O'Donnell N, Pall K, Leoni M, Debelle G, Lynn R, Armitage A, et al., editors. Female genital mutilation (FGM) surveillance in under 16 years olds in the UK and Ireland. Royal College of Paediatrics and Child Health Annual Conference, RCPCH 2018; 2018 2018-03-01; United Kingdom: Archives of Disease in Childhood.
77. Female Genital Mutilation: A clinical approach for GPs. Royal College of General Practitioners; 2017. Contract No.: 5.3.2017.
78. Elmusharaf S, Elhadi N, Almroth L. Reliability of self reported form of female genital mutilation and WHO classification: cross sectional study. BMJ. 2006;333(7559):124.
79. Johansen REB. Care for Infibulated Women Giving Birth in Norway: An Anthropological Analysis of Health Workers' Management of a Medically and Culturally Unfamiliar Issue. Medical Anthropology Quarterly. 2006;20(4):516-44.

80. Gabrasadig R, Asamoah F, Wilson N, editors. Female genital mutilation: knowledge, training and experience of healthcare professionals at a London hospital. Annual Conference of the Royal College of Paediatrics and Child Health, RCPCH 2015; 2015 2015-04-01; Birmingham, UK: Archives of Disease in Childhood.
81. Upvall MJ, Mohammed K, Dodge PD. Perspectives of Somali Bantu refugee women living with circumcision in the United States: A focus group approach. International Journal of Nursing Studies. 2009;46(3):360-8.
82. Safari F. A qualitative study of women's lived experience after deinfibulation in the UK. Midwifery. 2013;29(2):154-8.
83. Karlsen S, Carver N, Mogilnicka M, Pantazis C. When safeguarding becomes stigmatising: A report on the impact of FGM-safeguarding procedures on people with a Somali heritage living in Bristol. Bristol: University of Bristol; 2019.
84. Creighton SM, Samuel Z, Otoo-Oyortey N, Hodes D. Tackling female genital mutilation in the UK. BMJ. 2019;364:l15.
85. FORWARD. "A Big Wake-Up Call": Participatory Study on Shifts in Attitudes Towards FGM Amongst Community Women in Bristol, Summary Report. FORWARD

Refugee Women of Bristol; 2017.
86. Brown E, Porter C. The Tackling FGM Initiative: Evaluation of the Second Phase

(2013-2016). London: Options Consultancy Services Ltd; 2016.
87. Brown E, Porter C, Unit OP. Evaluation of FGM Prevention among Communities Affected by FGM: A Participatory Ethnographic Evaluation Research (PEER) Study. Endline Phase 2. Options Consultancy Services Ltd; 2016.
88. Ajibona A, editor The understanding of the term female genital mutilation or FGM amongst patients with FGM in a United Kingdom inner city ante-natal clinic. 21st FIGO World Congress of Gynecology and Obstetrics; 2015; Vancouver, BC Canada: International Journal of Obstetrics and Gynaecology.
89. Mohamed N, Schickler P, Warsame Z, Glew C. 'Hear Our Voices': A Report on Participatory Workshops on FGM/C (Female Genital Mutilation/Cutting) with the Somali Community in Tower Hamlets. London: Women's Health and Family Services; 2014.

For peer review only

90. Turkmani S, Homer C, Varol N, Dawson A. A survey of Australian midwives' knowledge, experience, and training needs in relation to female genital mutilation. Women and Birth. 2018;31(1):25-30.
91. Ariyo D, Ssali R, King-Webb L, Ikpaahindi S. Voices of the Community: Exploring Female Genital Mutilation in the African Community across Greater Manchester. Afruca; 2015.
92. Brown K, Beecham D, Barrett H. The Applicability of Behaviour Change in Intervention Programmes Targeted at Ending Female Genital Mutilation in the EU: Integrating Social Cognitive and Community Level Approaches. Obstetrics and Gynecology International. 2013;2013(2013):324362.
93. Straus L, McEwen A, Hussein FM. Somali women's experience of childbirth in the UK: Perspectives from Somali health workers. Midwifery. 2009;25(2):181-6.
94. Feder G, Wathen CN, MacMillan HL. An evidence-based response to intimate partner violence: WHO guidelines. JAMA. 2013;310(5):479-80.
95. Spangaro J, Koziol-McLain J, Zwi A, Rutherford A, Frail M-A, Ruane J. Deciding to tell: qualitative configurational analysis of decisions to disclose experience of intimate partner violence in antenatal care. Social Science & Medicine. 2016;154:45-53.
96. Foundations OS. Somalis in Leicester. New York, NY, USA: Open Society Foundations; 2014.
97. Connelly E, Murray N, Baillot H, Howard N. Missing from the debate? A qualitative study exploring the role of communities within interventions to address female genital mutilation in Europe. BMJ Open. 2018;8:e021430.
98. McCafrey M, Jankowska A, Gordon H. Management of female genital mutilation : the Northwick Park Hospital experience. BJOG: An International Journal of Obstetrics & Gynaecology. 1995;102(10):787-90.
99. Fawcett RJ, Kernohan G. A retrospective analysis of 34 potentially missed cases of female genital mutilation in the emergency department. Emergenct Medicine Journal. 2017;35(10):587-9.
100. Middleton J. Preventing violent extremism: the role of doctors. The Lancet. 2016;388(10057):2219-21.
101. Scamell M, Ghumman A. The experience of maternity care for migrant women living with female genital mutilation: A qualitative synthesis. Birth. 2019;46(1):15-23.
102. Ambuel B, Hamberger LK, Guse CE, Melzer-Lange M, Phelan MB, Kistner A. Healthcare can change from within: sustained improvement in the healthcare response to intimate partner violence. Journal of Family Violence. 2013;28(8):833-47.
103. Szilassy E, Drinkwater J, Hester M, Larkins C, Stanley N, Turner W, et al. Making the links between domestic violence and child safeguarding: an evidence-based pilot training for general practice. Health & Social Care in the Community. 2017;25(6):1722-32.
104. Drinkwater J, Stanley N, Szilassy E, Larkins C, Hester M, Feder G. Juggling confidentiality and safety: a qualitative study of how general practice clinicians document domestic violence in families with children. British Journal of General Practice. 2017;67(659):e437-e44.
105. Woodman J, Allister J, Rafi I, de Lusignan S, Belsey J, Petersen I, et al. A simple approach to improve recording of concerns about childmaltreatment in primary care records: developing a quality improvement intervention. British Journal of General Practice. 2012;62(600):e478-e86.
106. Salad J, Verdonk P, de Boer F, Abma TA. "A Somali girl is Muslim and does not have premarital sex. Is vaccination really necessary?" A qualitative study into the perceptions of Somali women in the Netherlands about the prevention of cervical cancer. International Journal for Equity in Health. 2015;14(1):68.
107. Foundations OS. Somalis in London. New York, NY, USA: Open Society Foundations; 2014. Contract No.: ISBN: 978-1-940983-07-3.
108. Murray L, Windsor C, Parker E, Tewfik O. The Experiences of African Women Giving Birth in Brisbane, Australia. Health Care for Women International. 2010;31(5):458-72.
109. Harper Bulman K, McCourt C. Somali refugee women's experiences of maternity care in west London: A case study. Critical Public Health. 2010;12(4):365-80.

For peer review only

110. Wellock VK. Domestic abuse: Black and minority-ethnic women's perspectives. Midwifery. 2010;26(2):181-8.
111. Feder GS, Hutson M, Ramsay J, Taket AR. Women exposed to intimate partner violence: expectations and experiences when they encounter health care professionals: a meta-analysis of qualitative studies. Archives of Internal Medicine. 2006;166(1):22-37.
112. Widmark C, Levál A, Tishelman C, Ahlberg BM. Obstetric care at the intersection of science and culture: Swedish doctors' perspectives on obstetric care of women who have undergone female genital cutting. Journal of Obstetrics and Gynecology. 2010;30(6):553-8.
113. Johnsdotter S. Discrimination of Certain Ethnic Groups? Ethical Aspects of Implementing FGM Legislation in Sweden. Report. Malmo, Sweden: Faculty of Health and Society, University of Malmo; 2009 2009. Contract No.: FoU Rapport 2009:3.
114. Leye E, Powell RA, Nienhuis G, Claeys P, Temmerman M. Health care in Europe for women with genital mutilation. Health Care for Women International. 2006;27(4):362-78.
115. Paliwal P, Ali S, Bradshaw S, Hughes A, Jolly K. Management of type III female genital mutilation in Birmingham, UK: a retrospective audit. Midwifery. 2014;30(3):282-8.
116. Horwood J, Morden A, Bailey JE, Pathak N, Feder G. Assessing for domestic violence in sexual health environments: a qualitative study. Sexually Transmitted Infections. 2017;94(2):88-92.
117. Feder G, Davies RA, Baird K, Dunne D, Eldridge S, Griffiths C, et al. Identification and Referral to Improve Safety (IRIS) of women experiencing domestic violence with a primary care training and support programme: a cluster randomised controlled trial. The Lancet. 2011;378(9805):1788-95.
118. Creighton SM, Liao LM. Tackling female genital mutilation in the UK. BMJ : British Medical Journal. 2013;347:f7150.
119. Turner W, Hester M, Broad J, Szilassy E, Feder G, Drinkwater J, et al. Interventions to improve the response of professionals to children exposed to domestic violence and abuse: a systematic review. Child Abuse Review. 2017;26(1):19-39.
120. Ashby J, Richardson A, Brawley D, E H, editors. National survey of practice and experience of mandatory reporting of female genital mutilation (FGM) amongst sexual health care professionals. 4th Joint Conference of the British HIV Association, BHIVA with the British Association for Sexual Health and HIV, BASHH 2018; 2018; United Kingdom: HIV Medicine.
121. Rymer J, editor Female genital mutilation. RCOG World Congress 2015; 2015; Brisbane, Australia: BJOG: An International Journal of Obstetrics and Gynaecology.
122. Naftalin J, Bewley S. Mandatory reporting of FGM. British Journal of General Practice. 2015;65(638):450-1.
123. Dixon S. The FGM enhanced dataset: how are we going to discuss this with our patients? British Journal of General Practice. 2015;65(641):629.
124. Taket A, Nurse J, Smith K, Watson J, Shakespeare J, Lavis V, et al. Routinely asking women about domestic violence in health settings. BMJ: British Medical Journal. 2003;327(7416):673-6.
125. Emam KE, Mercer J, Moreau K, Grava-Gubins I, Buckeridge D, Jonker E. Physician privacy concerns when disclosing patient data for public health purposes during a pandemic influenza outbreak. BMC Public Health. 2011;11(1):454.
126. Bismark MM, Mathews B, Morris JM, Thomas LA, Studdert DM. Views on mandatory reporting of impaired health practitioners by their treating practitioners: a qualitative study from Australia. BMJ Open. 2016;6:e011988.
127. Feng J-Y, Chen S-J, Wilk NC, Yang W-P, Fetzer S. Kindergarten teachers' experience of reporting child abuse in Taiwan: Dancing on the edge. Children and Youth Services Review. 2009;31(3):405-9.
128. McTavish JR, Kimber M, Devries K, Colombini M, MacGregor JCD, Wathen CN, et al. Mandated reporters' experiences with reporting child maltreatment: a meta-synthesis of qualitative studies. BMJ Open. 2017;7(10):e013942.

129. Gallagher A, Wainwright P, Tompsett H, Atkins C. Findings from a Delphi exercise regarding conflicts of interests, general practitioners and safeguarding children: 'Listen carefully, judge slowly'. Journal of Medical Ethics. 2012;38(2):87-92.
130. Beran R. Mandatory notification of impaired doctors. Internal Medicine Journal. 2014;44(12a):1161-5.
131. Foster R, Olson-Dorff D, Reiland H, Budzak-Garza A. Commitment, confidence and concerns: Assessing health care professionals' child maltreatment reporting attitudes. Child Abuse & Neglect. 2017;67:54-63.
132. Falkiner M, Thomson D, Day A. Teachers' Understanding and Practice of Mandatory Reporting of Child Maltreatment. Children Australia. 2017;42(1):38-48.
133. Talsma M, Bengtsson Boström K, Östberg A-L. Facing suspected child abuse – what keeps Swedish general practitioners from reporting to child protective services? Scandinavian Journal of Primary Health Care. 2013;33(1):21-6.
134. Hodes D. Female Genital Mutilation (FGM): 10 Year Case Series from the First UK Dedicated Clinic - Journal of Adolescent Health. 2018.
135. Creighton SM, Hodes D. Female genital mutilation: what every paediatrician should know. Archives of disease in childhood. 2016 Mar 1;101(3):267-71.
136. Erskine K. Collecting data on female genital mutilation. BMJ : British Medical Journal. 2014;348:g3222.
137. Johansen REB, Ziyada MM, Shell-Duncan B, Kaplan AM, Leye E. Health sector involvement in the management of female genital mutilation/cutting in 30 countries. BMC Health Services Research. 2018;18(1):240.

138. Lawson D, Niven B. The Impact of Mandatory Reporting Legislation on New Zealand Secondary School Students' Attitudes towards Disclosure of Child Abuse. International Journal of Children's Rights. 2015;23:491-528.
139. Simpson J, Robinson K, Creighton SM, Hodes D. Female genital mutilation: the role of health professionals in prevention, assessment, and management. Bmj. 2012 Mar 14;344:e1361.
140. Casla K, Roderick P, Pollock AM. Disclosure of patients' data to the UK Home Office must stop. BMJ : British Medical Journal. 2017;358:j3613.
141. Hiam L. Grenfell survivors shouldn't be afraid to go to hospital. BMJ : British Medical Journal. 2017;358:j3292.
142. Hiam L, Steele S, McKee M. Creating a 'hostile environment for migrants': the British government's use of health service data to restrict immigration is a very bad idea. Health Economics, Policy and Law. 2018;13(2):107-17.
143. Potter JL, Milner A. Tuberculosis: looking beyond 'migrant'as a category to understand experience. Race Equality Foundation Briefing Paper: A Better Health Briefing. 2018;44.
144. Evans, C., Tweheyo, R., McGarry, J., Eldridge, J., Albert, J., Nkoyo, V. and Higginbottom, G.M.A., 2019. Seeking culturally safe care: a qualitative systematic review of the healthcare experiences of women and girls who have undergone female genital mutilation/cutting. *BMJ open*, *9*(5), p.e027452.
145 Kawous, R., Allwood, E., Norbart, E. and van den Muijsenbergh, M.E., 2020. Female genital mutilation and women's healthcare experiences with general practitioners in the Netherlands: A qualitative study. *Plos one*, *15*(7), p.e0235867.
146 Rayment-Jones, H., Harris, J., Harden, A., Khan, Z. and Sandall, J., 2019. How do women with social risk factors experience United Kingdom maternity care? A realist synthesis. *Birth*, *46*(3), pp.461-474.

147 Robertshaw, L., Dhesi, S. and Jones, L.L., 2017. Challenges and facilitators for health professionals providing primary healthcare for refugees and asylum seekers in high-income countries: a systematic review and thematic synthesis of qualitative research. *BMJ open*, *7*(8).

148. Papageorgiou, V., Wharton-Smith, A., Campos-Matos, I. and Ward, H., 2020. Patient data-sharing for immigration enforcement: a qualitative study of healthcare providers in England. *BMJ open*, *10*(2).

149 Gerry, F., Rowland, A., Fowles, S., Smith, S., Hodes, D. and Creighton, S., 2016. Failure to evaluate introduction of female genital mutilation mandatory reporting. *Archives of disease in childhood*, *101*(8), pp.778-779.

150. Jacoby SD, Smith A. Increasing Certified Nurse-Midwives' Confidence in Managing the Obstetric Care of Women with Female Genital Mutilation/Cutting. *Journal of midwifery & women's health*. 2013;*58* (4),:pp.451-6.

151. Al-Saadi N, Khan H, Auckburally S, Malik B, Al-Saadi A, Saleem A. Reforming medical education: A formal analysis of London medical school's teaching on female genital cutting: EP12. Bjog: An International Journal of Obstetrics and Gynaecology. 2016;123.

152. John N, Raval H, Handa N, editors. Is female genital mutilation adequately taught to medical students? BJOG-AN INTERNATIONAL JOURNAL OF OBSTETRICS AND GYNAECOLOGY; 2018: WILEY 111 RIVER ST, HOBOKEN 07030-5774, NJ USA.

153. Holmes V, Farrington R, Mulongo P. Educating about female genital mutilation. http://dxdoiorg/101080/1473987920161245589. 2016.

154. Horvat L, Horey D, Romios P K-RJ. Cultural competence education for health professionals.: The Cochrane Collaboration; 2014.

155. Truong M, Paradies Y, Priest N. Interventions to improve cultural competency in healthcare: a systematic review of reviews. BMC Health Services Research. 2014;14(1):99.

156. Watt K, Abbott P, Reath J. Developing cultural competence in general practitioners: an integrative review of the literature. BMC Family Practice. 2016;17(1):158.

157 Harrison GA, Dixon S, Griffiths F, Healthcare Professionals' Knowledge, Attitude and Feelings Towards Female Genital Mutilation: A Systematic Review, RCOG: World Summit, 2019

158. Farage MA, Miller KW, Tzeghai GE, Azuka CE, Sobel JD, Ledger WJ. Female Genital Cutting: Confronting Cultural Challenges and Health Complications Across the Lifespan:. http://dxdoiorg/102217/WHE1463. 2015.

For peer review only

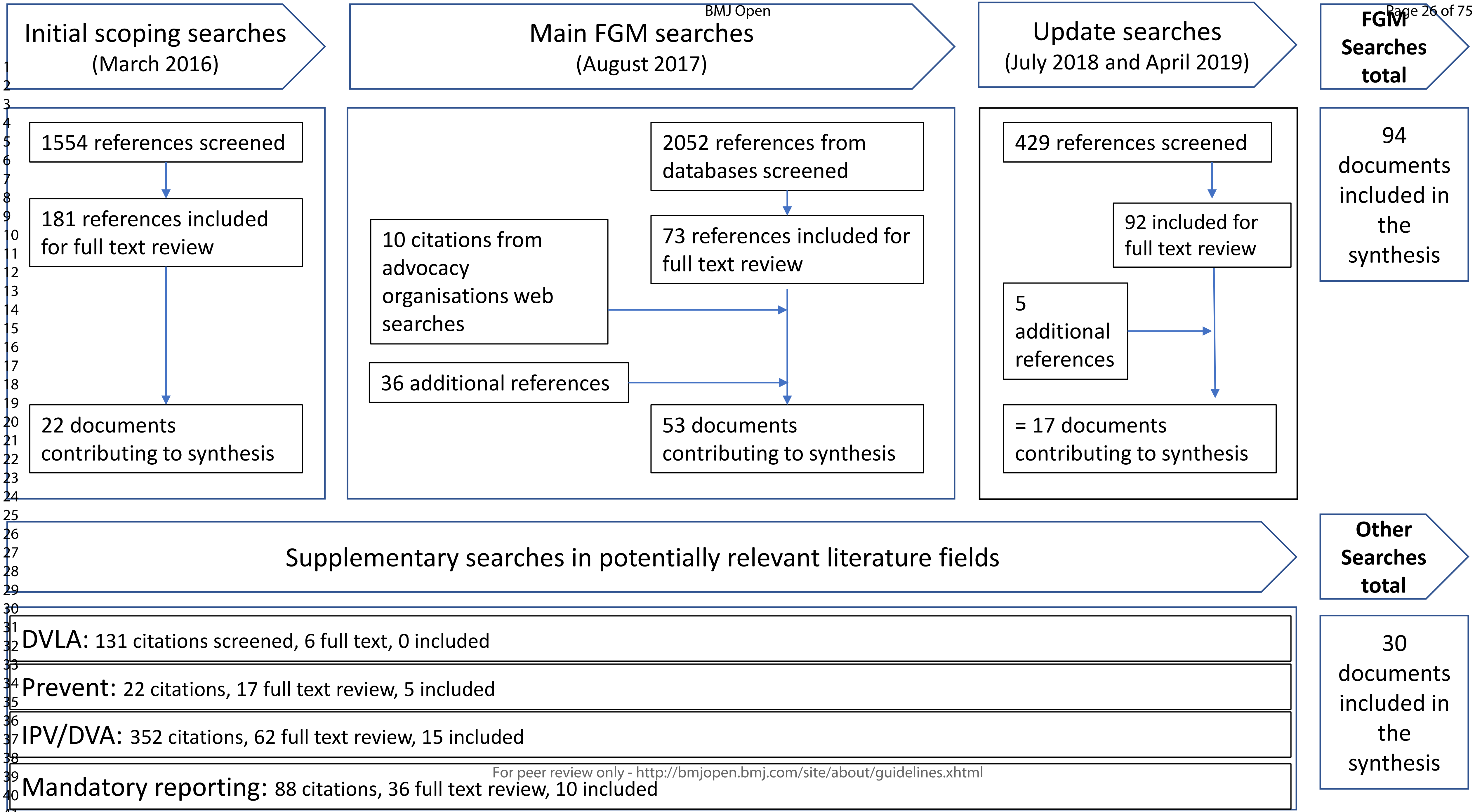
Initial scoping searches
(March 2016)
1554 references screened
181 references included for full text review
22 documents contributing to synthesis
Main FGM searches
(August 2017)
2052 references from databases screened
73 references included for full text review
10 citations from advocacy organisations web searches
36 additional references
53 documents contributing to synthesis
Update searches
(July 2018 and April 2019)
429 references screened
92 included for full text review
5 additional references
= 17 documents contributing to synthesis
FGM Searches total
94 documents included in the synthesis
Supplementary searches in potentially relevant literature fields
DVLA: 131 citations screened, 6 full text, 0 included
Prevent: 22 citations, 17 full text review, 5 included
IPV/DVA: 352 citations, 62 full text review, 15 included
Mandatory reporting: 88 citations, 36 full text review, 10 included
Other Searches total
30 documents included in the synthesis

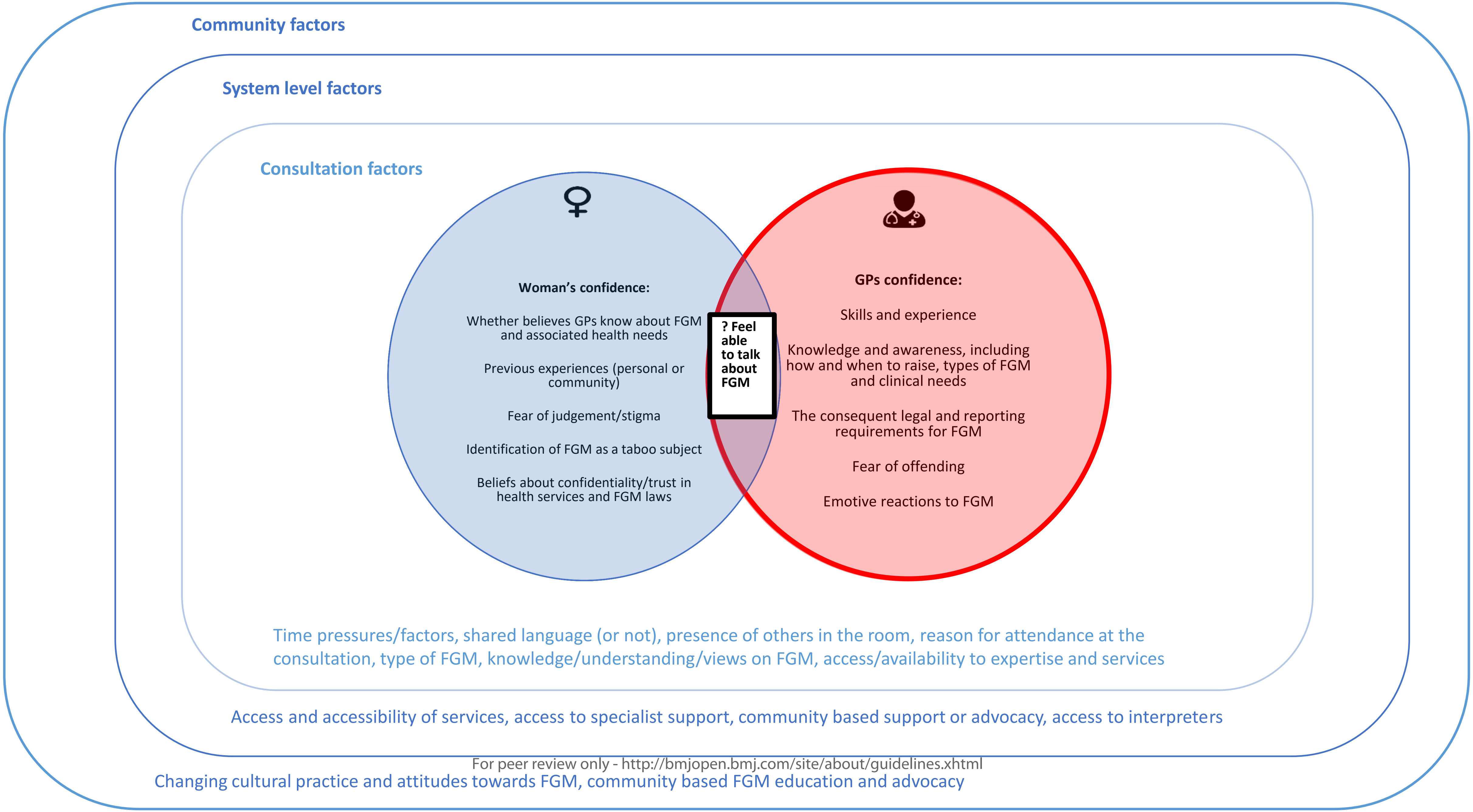
Community factors
System level factors
Consultation factors
Woman's confidence:
Whether believes GPs know about FGM and associated health needs
Previous experiences (personal or community)
Fear of judgement/stigma
Identification of FGM as a taboo subject
Beliefs about confidentiality/trust in health services and FGM laws
? Feel able to talk about FGM
GPs confidence:
Skills and experience
Knowledge and awareness, including how and when to raise, types of FGM and clinical needs
The consequent legal and reporting requirements for FGM
Fear of offending
Emotive reactions to FGM
Time pressures/factors, shared language (or not), presence of others in the room, reason for attendance at the consultation, type of FGM, knowledge/understanding/views on FGM, access/availability to expertise and services
Access and accessibility of services, access to specialist support, community based support or advocacy, access to interpreters
Changing cultural practice and attitudes towards FGM, community based FGM education and advocacy

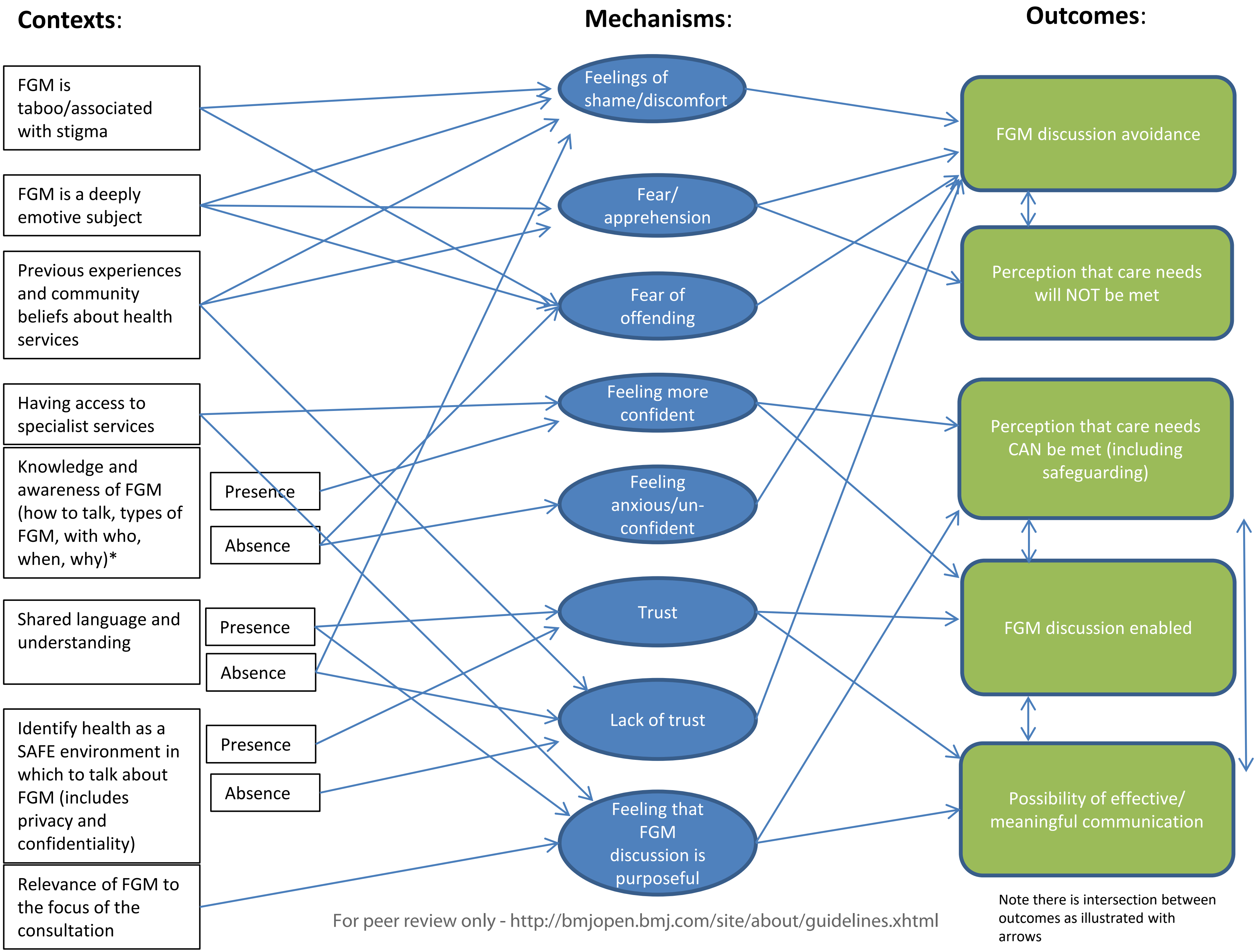




* This is a Continuum including awareness of lack of knowledge, and lack of this awareness

**English general medical practitioners managing female genital mutilation in light of recent policy and legislative changes: A realist synthesis**

Sharon Dixon, Claire Duddy, Gabrielle Harrison, Chrysanthi Papoutsi, Sue Ziebland, Frances Griffiths.

Prospero: CRD4201891996

**Review Question:**

1. From a realist synthesis of published literature, what influences how GPs manage female genital mutilation (FGM) in their clinical practice and why?
2. What influences general medical practitioners (GPs) actions when they consider initiating discussion about female genital mutilation (FGM) with patients in primary care? Where, when and why are these influences active?
3. What influences how GPs respond to a patient-initiated disclosure of FGM during a primary care consultation? Where, when and why are these influences active?

4. **Searches:**

We will use the RAMESES quality standards for realist synthesis to guide this realist review.

We will use an information specialist to support the literature searches.

Initial programme theory will be derived from reviewing governmental policy documents relevant to the management of FGM in primary care and FGM guidance from doctors' professional bodies. The subsequent search strategy will be designed to identify empirical and theoretical literature to test the initial programme theories. The search process in a realist review is iterative and responsive to evolving programme theory. . The preliminary search will be run with keywords identified from an exploratory literature review, and will include FGM, primary care, and general practice/GP. e would search broadly, including using the following databases: Medline, Embase, PsycINFO, Global Health, Cinahl, Web of Science, Sociological Abstracts, Anthropology Plus, Social Science Abstracts and ASSIA, and undertake forward and backward citation searches to identify relevant papers

Subsequent literature searches will be responsive to emergent programme theory including potentially relevant contexts, mechanisms, and outcomes. As new theory emerges, new evidence needs to be considered to test this. We will purposively search and iteratively review evidence. All steps and decisions taken during the review will be reported and justified, including details about actions taken We will report review search strategies (initial and subsequent searches), the number of studies identified and assessed for relevance, the number and type of papers included and excluded and reasons for exclusion.

For peer review only - http://bmjopen.bmj.com/site/about/guidelines.xhtml

**Types of study to be included:**

All study designs and theoretical papers, including conceptual papers and commentaries will be considered if they contribute to the development or testing of programme theory. Papers considering interventions in health care contexts very different to the UK healthcare system will be excluded. Where there is a large body of literature, we will identify systematic reviews in the first instance. We will include papers written in English.

**Condition or domain being studied:**

How GPs are managing women and families from FGM affected communities

**Participants/population:**

GPs working in the UK

**Intervention/exposure:**

Any identified factor or context that may influence GPs' actions and reactions in relation to their management of patients affected by FGM.

**Comparator/control:**

Contexts where an identified influence is not present

**Primary outcome:**

This realist review will result in programme theory, tested against existing evidence, on what influences GPs actions and reactions in relation to FGM in primary care consultations and, where, when and why these influences are activated.

**Timing and effect measures:**

No limitation placed on timing of influences.

Effect is defined as action or reaction of GPs in relation to FGM within primary care consultations

**Secondary outcome:**

None

**Data extraction (selection and coding):**

For peer review only

Data extraction and organisation of the data will be undertaken by SD. Documents will be initially screened with title and abstract, and selected for inclusion for full text screening. Following full text screening, relevant articles will be included for coding in Nvivo. A random sample of 10% of finally included documents, screened abstracts, and coded full text documents will be independently checked by another member of the study team as a quality assurance measure. Disagreements will be resolved by discussion within the study team.

The full text of included papers will be uploaded onto qualitative data analysis software tool (NVivo)a. We will code text sections for their relevance (or not) to each programme theory, and within that code context, mechanisms, and outcomes. The coding structure will be initially deductive derived from the initial programme theory, and also inductive, generated to categorise data in included papers. The processes of both coding and analysis are iterative. Through cross comparison of coded data, we expect to generate theory. This may suggest the need for further interrogation of collated evidence and so new coding and searches for new evidence.

**Risk of bias (quality) assessment:**

The study will be guided by the RAMESES quality standards for realist reviews (http://www.ramesesproject.org/Standards_and_Training_materials.php#qual_stand_rs)

Papers will be appraised for relevance to the theory under test, not necessarily the topic under consideration, and for the rigour of the conclusions the authors drew. All included papers will be assessed independently by two team members.

**Strategy for data synthesis:**

Data analysis and synthesis will use a realist logic of analysis. In a realist review, the process of data analysis and synthesis co-exist and overlap. In realist terms, synthesis is progress towards explanations, which are framed as relationships between the contexts (C) in which outcomes under observation occur (O), and the mechanisms that potentially link these (M). These relationships are postulated as CMO configurations (CMOC), which are considered within over-arching programme theory. During analysis, we will use interpretive cross comparison, to understand and explain how different interventions have produced outcomes in different contexts and how we understand the potential mechanisms that link these. During CMOC development, we will review how the data supports the developing CMOCs, how new CMOCs relate to other postulated CMOCs and how they relate to programme theory being developed. The synthesis will include retroductive analysis seeking to identify hidden causes that can explain observed patterns in the data.

**Analysis of subgroups or subsets:**

We will potentially be testing more than one programme theory

For peer review only

.

For peer review only

For peer review only - http://bmjopen.bmj.com/site/about/guidelines.xhtml

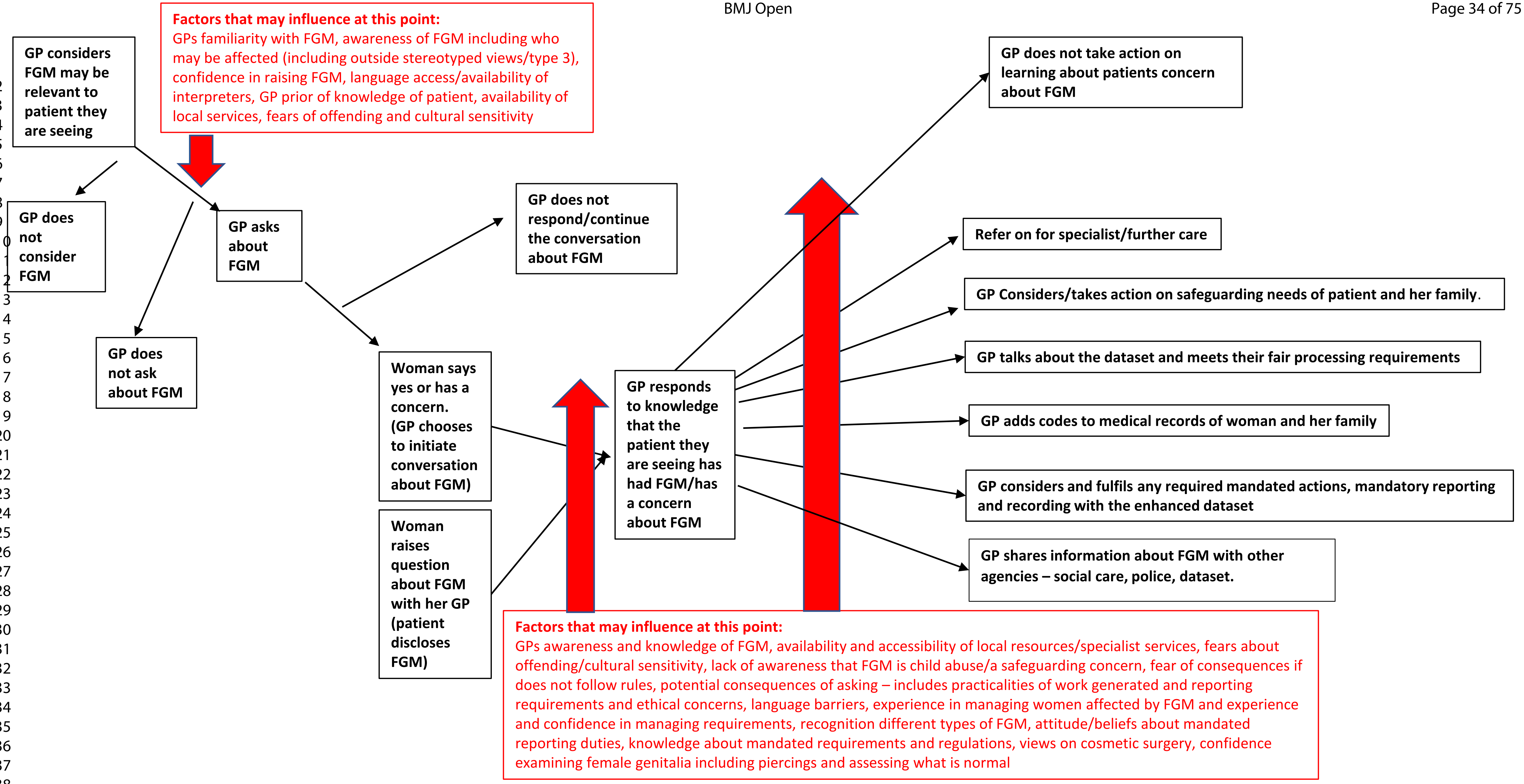


For peer review only - http://bmjopen.bmj.com/site/about/guidelines.xhtml

*The initial programme theory mapped onto a primary care consultation. The black boxes represent hypothesised possible outcomes and the red text and boxes represent potential contextual or mechanistic factors identified from an exploratory literature review and stakeholder expertise.*

Appendix C: Search strategies

Initial scoping searches (March 2016)

Ovid MEDLINE Search #1

# ▲ Searches

1 Circumcision, Female/

2 female genital mutilation.ti,ab.

3 ((female? or women or girl*) adj2 circumcis*).ti,ab.

4 ((genital? adj2 (cut or cuts or cutting)) and (female? or women or girl*)).ti,ab.

5 1 or 2 or 3 or 4

6 Prevalence/

7 *"surveys and questionnaires"/ or health care surveys/ or health surveys/

8 (prevalen* or burden or trend? or estimat* or survey?).ti.

9 6 or 7 or 8

10 5 and 9

11 limit 10 to yr="2006 -Current"

Ovid MEDLINE Search #2

#▲ Searches

1 Circumcision, Female/

2 female genital mutilation.ti,ab.

3 ((female? or women or girl*) adj2 circumcis*).ti,ab.

4 ((genital? adj2 (cut or cuts or cutting)) and (female? or women or girl*)).ti,ab.

5 1 or 2 or 3 or 4

6 (Qualitative systematic review* or (systematic review and qualitative)).ti,ab.

7 (evidence synthesis or realist synthesis).ti,ab.

8 (Qualitative and synthesis).ti,ab.

9 (meta-synthesis* or meta synthesis* or metasynthesis).ti,ab.

10 (meta-ethnograph* or metaethnograph* or meta ethnograph*).ti,ab.

11 (meta-study or metastudy or meta study).ti,ab.

12 (realist review? or realist synthesis).ti,ab.

13 systematic review*.ti,ab. and qualitative research/

14 6 or 7 or 8 or 9 or 10 or 11 or 12 or 13

For peer review only

15 5 and 14

16 qualitative research/

17 *interviews as topic/ or focus groups/ or narration/

18 observation.ti.

19 interview?.ti.

20 (qualitative adj2 (interview* or study)).ti,ab.

21 (qualitative or focus group? or story or stories or narration or narrative* or discourse or discursive or grounded theory or ethnogra* or phenomenolog*).ti,ab.

22 16 or 17 or 18 or 19 or 20 or 21

23 5 and 22

24 15 or 23

Global Health Search #1

# ▲ Searches

1 female genital mutilation.ti,ab.

2 ((female? or women or girl*) adj2 circumcis*).ti,ab.

3 ((genital? adj2 (cut or cuts or cutting)) and (female? or women or girl*)).ti,ab.

4 1 or 2 or 3

5 Prevalence/

6 surveys/ or household surveys/

7 (prevalen* or burden or trend? or estimat* or survey?).ti.

8 5 or 6 or 7

9 4 and 8

10 limit 9 to yr="2006 -Current"

Global Health Search #2

#

▲

Searches

1 female genital mutilation.ti,ab.

2 ((genital? adj2 (cut or cuts or cutting)) and (female? or women or girl*)).ti,ab.

3 ((female? or women or girl*) adj2 circumcis*).ti,ab.

4 1 or 2 or 3

For peer review only

5 (qualitative or focus group? or story or stories or narration or narrative* or discourse or discursive or grounded theory or ethnogra* or phenomenolog*).ti,ab.

6 interview*.ti,ab.

7 5 or 6

8 4 and 7

Embase

# ▲ Searches

1 exp female genital mutilation/

2 female genital mutilation.ti,ab.

3 ((female? or women or girl*) adj2 circumcis*).ti,ab.

4 ((genital? adj2 (cut or cuts or cutting)) and (female? or women or girl*)).ti,ab.

5 1 or 2 or 3 or 4

6 (Qualitative systematic review* or (systematic review and qualitative)).ti,ab.

7 (evidence synthesis or realist synthesis).ti,ab.

8 (Qualitative and synthesis).ti,ab.

9 (meta-synthesis* or meta synthesis* or metasynthesis).ti,ab.

10 (meta-ethnograph* or metaethnograph* or meta ethnograph*).ti,ab.

11 (meta-study or metastudy or meta study).ti,ab.

12 (realist review? or realist synthesis).ti,ab.

13 systematic review*.mp. and exp qualitative studies/

14 6 or 7 or 8 or 9 or 10 or 11 or 12 or 13

15 5 and 14

16 grounded theory/ or naturalistic inquiry/ or qualitative research/

17 exp *interview/

18 observation.ti.

19 interview?.ti.

20 (qualitative adj2 (interview* or study)).ti,ab.

21 (qualitative or focus group? or story or stories or narration or narrative* or discourse or discursive or grounded theory or ethnogra* or phenomenolog*).ti,ab.

22 16 or 17 or 18 or 19 or 20 or 21

23 5 and 22

For peer review only

24 15 or 23

PsycINFO

# ▲ Searches

1 circumcision/

2 female genital mutilation.ti,ab.

3 ((genital? adj2 (cut or cuts or cutting)) and (female? or women or girl*)).ti,ab.

4 ((female? or women or girl*) adj2 circumcis*).ti,ab.

5 1 or 2 or 3 or 4

6 (Qualitative systematic review* or (systematic review and qualitative)).ti,ab.

7 (evidence synthesis or realist synthesis).ti,ab.

8 (Qualitative and synthesis).ti,ab.

9 (meta-synthesis* or meta synthesis* or metasynthesis).ti,ab.

10 (meta-ethnograph* or metaethnograph* or meta ethnograph*).ti,ab.

11 (meta-study or metastudy or meta study).ti,ab.

12 (realist review? or realist synthesis).ti,ab.

13 systematic review*.ti,ab. and qualitative research/

14 6 or 7 or 8 or 9 or 10 or 11 or 12 or 13

15 5 and 14

16 qualitative research/ or grounded theory/ or observation methods/

17 exp *interviews/

18 observation.ti.

19 interview?.ti.

20 (qualitative adj2 (interview* or study)).ti,ab.

21 (qualitative or focus group? or story or stories or narration or narrative* or discourse or discursive

or grounded theory or ethnogra* or phenomenolog*).ti,ab.

22 16 or 17 or 18 or 19 or 20 or 21

23 5 and 22

24 15 or 23

These search strategies were also adapted and run in

• ASSIA,

For peer review only

• Sociological Abstracts

• CINAHL

• Anthropology Plus

• Web of Science (Core Collection databases)

A total of 1554 references were screened by title and abstract, 181 were read in full text and 22 contributed to the synthesis.

Main FGM searches (August 2017)

Summary of searching and results

Database Interface Coverage Date GP

hits

UK

Hits

Ovid MEDLINE(R) Epub

Ahead of Print, In-Process &

Other Non-Indexed

Citations, Ovid MEDLINE(R)

Daily and Ovid MEDLINE(R)

OvidSp 1946-present 22/08/2017 21 384

Embase OvidSp 1974 to 2017

August 21

22/08/2017 31 396

PsycINFO OvidSp 1967 to August

Week 2 2017

22/08/2017 0 57

CINAHL EBSCOHost 1982-present 22/08/2017 16 575

Web of Science Core

Collection

Thomson

Reuters

1945-present 22/08/2017 0 280

Nexis UK Lexis Nexis 22/08/2017 0 260

For peer review only

Proquest Social Science

Databases

Proquest 22/08/2017 5 100

MEDLINE

#

▲

Searches

1 Circumcision, Female/

2 (femal genital mutilation or fgm).ti,ab.

3 ((genital? adj2 (cut or cuts or cutting)) and (female? or women or girls)).ti,ab.

4 ((female? or women or girl*) adj2 circumcis*).ti,ab.

5 1 or 2 or 3 or 4

6 exp General Practice/

7 general practitioners/ or physicians, family/ or physicians, primary care/

8 Primary Health Care/

9 (((general or family) adj2 (practi* or physician? or doctor?)) or gp or gps).ti,ab.

10 (doctor? or physician?).ti.

11 (primary adj2 (care or healthcare)).ti,ab.

12 6 or 7 or 8 or 9 or 10 or 11

13 exp United Kingdom/

14 (united kingdom or uk or britain or gb or england or wales or scotland or northern ireland).ti,ab,in.

15 (nhs or national health service).ti,ab,in.

16 (british or bjgp or bmj or hsj or pulse or gp or general practi* or primary care or nursing standard or nursing times).jw.

17 13 or 14 or 15 or 16

18 5 and 12 and 17

19 5 and 17

Embase

#

▲

For peer review only

Searches

1 female genital mutilation/

2 (femal genital mutilation or fgm).ti,ab.

3 ((genital? adj2 (cut or cuts or cutting)) and (female? or women or girls)).ti,ab.

4 ((female? or women or girl*) adj2 circumcis*).ti,ab.

5 1 or 2 or 3 or 4

6 General Practice/

7 general practitioner/

8 Primary Medical Care/

9 (((general or family) adj2 (practi* or physician? or doctor?)) or gp or gps).ti,ab.

10 (doctor? or physician?).ti.

11 (primary adj2 (care or healthcare)).ti,ab.

12 6 or 7 or 8 or 9 or 10 or 11

13 exp United Kingdom/

14 (united kingdom or uk or britain or gb or england or wales or scotland or northern ireland).ti,ab,in.

15 (nhs or national health service).ti,ab,in.

16 (british or bjgp or bmj or hsj or pulse or gp or general practi* or primary care or nursing standard or nursing times).jw.

17 13 or 14 or 15 or 16

18 5 and 12 and 17

19 5 and 17

PsycINFO

#

▲

Searches

1 (exp Human Females/ or exp Female Genitalia/) and exp Circumcision/

2 (femal genital mutilation or fgm).ti,ab.

3 ((genital? adj2 (cut or cuts or cutting)) and (female? or women or girls)).ti,ab.

4 ((female? or women or girl*) adj2 circumcis*).ti,ab.

5 1 or 2 or 3 or 4

For peer review only

6 general practitioners/

7 Primary Health Care/

8 (((general or family) adj2 (practi* or physician? or doctor?)) or gp or gps).ti,ab.

9 (doctor? or physician?).ti.

10 (primary adj2 (care or healthcare)).ti,ab.

11 6 or 7 or 8 or 9 or 10

12 (united kingdom or uk or britain or gb or england or wales or scotland or northern ireland).ti,ab,in.

13 (nhs or national health service).ti,ab,in.

14 (british or bjgp or bmj or hsj or pulse or gp or general practi* or primary care or nursing standard or nursing times).jw.

15 12 or 13 or 14

16 5 and 11 and 15

17 5 and 15

CINAHL

# Query

S19 S5 AND S17

S18 S5 AND S12 AND S17

S17 S13 OR S14 OR S15 OR S16

S16 SO british or bjgp or bmj or hsj or pulse or gp or general practi* or primary care or nursing standard or nursing times)

S15 TX nhs or "national health service"

S14 TX "united kingdom" or uk or britain or gb or england or wales or scotland or "northern ireland"

S13 (MH "United Kingdom+")

S12 S6 OR S7 OR S8 OR S9 OR S10 OR S11

S11 TI ( (primary N2 (care or healthcare)) ) OR AB ( (primary N2 (care or healthcare)) )

S10 TI doctor? or physician?

S9 TI ( (((general or family) N2 (practi* or physician? or doctor?)) or gp or gps) ) OR AB ( (((general or family) N2 (practi* or physician? or doctor?)) or gp or gps) )

S8 (MH "Primary Health Care")

S7 (MH "Physicians, Family")

For peer review only

S6 (MH "Family Practice")

S5 S1 OR S2 OR S3 OR S4

S4 TI ( ((female? or women or girl*) N2 circumcis*) ) OR AB ( ((female? or women or girl*) N2 circumcis*) )

S3 TI ( ((genital? N2 (cut or cuts or cutting)) and (female? or women or girls)) ) OR AB ( ((genital? N2 (cut or cuts or cutting)) and (female? or women or girls)) )

S2 TI ( "femal genital mutilation" or fgm ) OR AB ( "femal genital mutilation" or fgm )

S1 (MH "Circumcision, Female")

Web of Science (Core Collection)

Set Results

# 5 280 #3 AND #1

# 4 0 #3 AND #2 AND #1

# 3 5,132,695 TOPIC: ("united kingdom" or uk or britain or gb or england or wales or scotland or "northern ireland") OR ADDRESS: ("united kingdom" or uk or britain or gb or england or wales or scotland or "northern ireland") OR TOPIC: (nhs or "national health service") OR ADDRESS: (nhs or "national health service") OR PUBLICATION NAME: (british or bjgp or bmj or hsj or pulse or gp or general practi* or primary care or nursing standard or nursing times)

# 2 339,275 TOPIC: ((((general or family) NEAR/2 (practi* or physician? or doctor?)) or gp or gps)) OR TITLE: (doctor? or physician?) OR TOPIC: ((primary NEAR/2 (care or healthcare)))

# 1 5,251 TOPIC: ("femal genital mutilation" or fgm) OR TOPIC: (((genital? NEAR/2 (cut or cuts or cutting)) and (female? or women or girls))) OR TOPIC: (((female? or women or girl*) NEAR/2 circumcis*))

Nexis UK

fgm OR "female genital mutilation" In the Headline

gp OR gps OR general practitioners OR doctors Anywhere in the text

Limits

Previous 2 year

UK publications

Proquest Social Science Databases

Set Search

For peer review only

S7 S3 AND S5

S6 S3 AND S4 AND S5

S5 all("united kingdom" or uk or britain or gb or england or wales or scotland or "northern ireland") OR all(nhs OR "national health service")

S4 all(((general or family) NEAR/2 (practi* or physician or doctor)) or gp or gps) OR ti(doctor OR physician) OR all("primary care" OR "primary healthcare" OR "primary health care")

S3 all("femal genital mutilation" or fgm) OR all((((genital? NEAR/2 (cut or cuts or cutting)) and (female? or women or girls)))) OR all((((female? or women or girl*) NEAR/2 circumcis*)))

A total of 2052 references were screened by title and abstract, 73 were read in full text.

Website searches

In addition to these searches, a search of relevant advocacy organisation websites was undertaken, including searching:

• Sharon add a list of links to relevant organisation homepages here – just illustrative not everything, say, maybe 5?

Update searches (July 2018, April 2019)

Summary of searching and results (July 2018)

Database Interface Coverage Date GP

hits

UK

Hits

Ovid MEDLINE(R) Epub

Ahead of Print, In-Process &

Other Non-Indexed

Citations, Ovid MEDLINE(R)

Daily and Ovid MEDLINE(R)

OvidSp 1946-present 22/08/2017 21 384

Embase OvidSp 1974 to 2017

August 21

22/08/2017 31 396

For peer review only

PsycINFO OvidSp 1967 to August

Week 2 2017

22/08/2017 0 57

CINAHL EBSCOHost 1982-present 22/08/2017 16 575

Web of Science Core

Collection

Thomson

Reuters

1945-present 22/08/2017 0 280

Nexis UK Lexis Nexis 22/08/2017 0 260

Proquest Social Science

Databases

Proquest 22/08/2017 5 100

Update searches in July 2018 replicated the Main FGM searches shown above, limited to results added to databases from August 2017 onwards.

Summary of searching and results (April 2019)

Database Interface Coverage Date GP

hits

Other

health

profs

Hits

Ovid MEDLINE(R) Epub

Ahead of Print, In-Process

& Other Non-Indexed

Citations, Ovid

MEDLINE(R) Daily and

Ovid MEDLINE(R)

OvidSp 1946-present 23/04/2019 2 7

Embase OvidSp 1974 to 2017

August 21

For peer review only

23/04/2019 1 31

PsycINFO OvidSp 1967 to August

Week 2 2017

23/04/2019 0 0

CINAHL EBSCOHost 1982-present 23/04/2019 3 10

Web of Science Core

Collection

Thomson

Reuters

1945-present 23/04/2019 7 Not

searched

Nexis UK Lexis Nexis 23/04/2019 90 Not

searched

Proquest Social Science

Databases

Proquest 23/04/2019 3 48

Update searches in April 2019 replicated the Main FGM searches shown above, limited to results added to databases from July 2018 onwards. For pragmatic reasons, WoS Core Collection and Nexis UK were only searched for terms relating to GPs/primary care and not other health professional groups.

A total of 429 new references were identified in the update searches and screened by title and abstract, 92 were read in full text.

Supplementary searches (Various dates)

Summary of searching and results: DVLA, IPV/DVA and Prevent searches (August 2017)

Database Interface Coverage Date DVLA IPV Prevent

Ovid MEDLINE(R) Epub

Ahead of Print, InProcess & Other NonIndexed Citations, Ovid

MEDLINE(R) Daily and

Ovid MEDLINE(R)

OvidSp 1946-present 22/08/2017 52 142 7

Embase OvidSp 1974 to 2017

For peer review only

August 21

22/08/2017 137 244 9

PsycINFO OvidSp 1967 to August

Week 2 2017

22/08/2017 8 69 1

CINAHL EBSCOHost 1982-present 22/08/2017 53 399 69

Web of Science Core

Collection

Thomson

Reuters

1945-present 22/08/2017 74 150 14

Proquest Social Science

Databases

Proquest 22/08/2017 10 57 16

Summary of searching and results: Mandatory reporting search (May 2018)

Database Interface Coverage Date Results

PubMed PubMed 1946-present May 2018

Web of Science (Core

Collection)

Thomson

Reuters

1945-present May 2018

DVLA (August 2017)

MEDLINE

#

▲

Searches

1 ("driver and vehicle licensing agency" or dvla).ti,ab.

2 ((driving or driver?) adj5 (notif* or report* or inform*)).ti,ab.

3 ((driving or driver?) adj5 (ability or competen* or incompeten* or status or continu*

or discontinu* or stop* or quit* or cease* or cessation)).ti,ab.

For peer review only

4 ((driving or driver?) adj3 (licens* or law* or legal*)).ti,ab.

5 (driv* adj3 (fit or fitness)).ti,ab.

6 Licensure/ and Automobile Driving/

7 1 or 2 or 3 or 4 or 5 or 6

8 exp General Practice/

9 general practitioners/ or physicians, family/ or physicians, primary care/

10 Primary Health Care/

11 (((general or family) adj2 (practi* or physician? or doctor?)) or gp or gps).ti,ab.

12 (doctor? or physician?).ti.

13 (primary adj2 (care or healthcare)).ti,ab.

14 8 or 9 or 10 or 11 or 12 or 13

15 exp United Kingdom/

16 (united kingdom or uk or britain or gb or england or wales or scotland or northern ireland).ti,ab,in.

17 (nhs or national health service).ti,ab,in.

18 (british or bjgp or bmj or hsj or pulse or gp or general practi* or primary care or nursing standard or nursing times).jw.

19 15 or 16 or 17 or 18

20 7 and 14 and 19

Embase

#

▲

Searches

1 ("driver and vehicle licensing agency" or dvla).ti,ab.

2 ((driving or driver?) adj5 (notif* or report* or inform*)).ti,ab.

3 ((driving or driver?) adj5 (ability or competen* or incompeten* or status or continu* or discontinu* or stop* or quit* or cease* or cessation)).ti,ab.

4 ((driving or driver?) adj3 (licens* or law* or legal*)).ti,ab.

5 (driv* adj3 (fit or fitness)).ti,ab.

6 driving ability/ or driver licence/

7 1 or 2 or 3 or 4 or 5 or 6

For peer review only

8 General Practice/

9 general practitioner/

10 Primary Medical Care/

11 (((general or family) adj2 (practi* or physician? or doctor?)) or gp or gps).ti,ab.

12 (doctor? or physician?).ti.

13 (primary adj2 (care or healthcare)).ti,ab.

14 8 or 9 or 10 or 11 or 12 or 13

15 exp United Kingdom/

16 (united kingdom or uk or britain or gb or england or wales or scotland or northern ireland).ti,ab,in.

17 (nhs or national health service).ti,ab,in.

18 (british or bjgp or bmj or hsj or pulse or gp or general practi* or primary care or nursing standard or nursing times).jw.

19 15 or 16 or 17 or 18

20 7 and 14 and 19

PsycINFO

# ▲ Searches

1 ("driver and vehicle licensing agency" or dvla).ti,ab.

2 ((driving or driver?) adj5 (notif* or report* or inform*)).ti,ab.

3 ((driving or driver?) adj5 (ability or competen* or incompeten* or status or continu* or discontinu* or stop* or quit* or cease* or cessation)).ti,ab.

4 ((driving or driver?) adj3 (licens* or law* or legal*)).ti,ab.

5 (driv* adj3 (fit or fitness)).ti,ab.

6 1 or 2 or 3 or 4 or 5

7 general practitioners/

8 Primary Health Care/

9 (((general or family) adj2 (practi* or physician? or doctor?)) or gp or gps).ti,ab.

10 (doctor? or physician?).ti.

11 (primary adj2 (care or healthcare)).ti,ab.

12 7 or 8 or 9 or 10 or 11

13 (united kingdom or uk or britain or gb or england or wales or scotland or northern

For peer review only

ireland).ti,ab,in.

14 (nhs or national health service).ti,ab,in.

15 (british or bjgp or bmj or hsj or pulse or gp or general practi* or primary care or nursing standard or nursing times).jw.

16 13 or 14 or 15

17 6 and 12 and 16

CINAHL

S6 S3 AND S4 AND S5

S5 ( ((driving or driver?) N5 (notif* or report* or inform*)) ) OR ( ((driving or driver?) N5 (ability or competen* or incompeten* or status or continu* or discontinu* or stop* or quit* or cease* or cessation)) ) OR ( ((driving or driver?) N3 (licens* or law* or legal*)) ) OR ( (driv* N3 (fit or fitness)) ) OR ( "driver and vehicle licensing agency" or dvla )

S4 (MH "United Kingdom+") OR ( AB "united kingdom" or uk or britain or gb or england or wales or scotland or "northern ireland" ) OR ( AB nhs or "national health service" ) OR ( SO british or bjgp or bmj or hsj or pulse or gp or general practi* or primary care or nursing standard or nursing times ) OR ( TI "united kingdom" or uk or britain or gb or england or wales or scotland or "northern ireland" ) OR ( TI nhs or "national health service" )

S3 S1 OR S2

S2 TI ( (((general or family) N2 (practi* or physician? or doctor?)) or gp or gps) ) OR AB ( (((general or family) N2 (practi* or physician? or doctor?)) or gp or gps) ) OR TI ( doctor? or physician? ) OR TI ( (primary N2 (care or healthcare)) ) OR AB ( (primary N2 (care or healthcare)) )

S1 (MH "Family Practice") OR (MH "Physicians, Family") OR (MH "Primary Health Care")

Web of Science (Core Collection)

#

4

74 #3 AND #2 AND #1

#

3

36,160 TS=("driver and vehicle licensing agency" or dvla) OR TS=(((driving or driver?) NEAR/5 (notif* or report* or inform*))) OR TS=(((driving or driver?) NEAR/5

(ability or competen* or incompeten* or status or continu* or discontinu* or stop* or quit* or cease* or cessation))) OR TS=(((driving or driver?) NEAR/3 (licens* or law* or legal*))) OR TS=((driv* NEAR/3 (fit or fitness)))

#

2

5,132,695 TS=("united kingdom" or uk or britain or gb or england or wales or scotland or "northern ireland") OR ADDRESS: ("united kingdom" or uk or britain or gb or england or wales or scotland or "northern ireland") OR TS=(nhs or "national health service") OR ADDRESS: (nhs or "national health service") OR PUBLICATION NAME: (british or bjgp or bmj or hsj or pulse or gp or general practi* or primary care or nursing standard or nursing times)

#

1

339,275 TS=((((general or family) NEAR/2 (practi* or physician? or doctor?)) or gp or gps)) OR TI=(doctor? or physician?) OR TS=((primary NEAR/2 (care or healthcare)))

Proquest Social Science Databases

S6 S1 AND S2 AND S5

S5 all("driver and vehicle licensing agency" OR dvla) OR all((driving OR driver) NEAR/5 (notif* OR report* OR inform*)) OR all(((driving OR driver) NEAR/5 (ability OR competen* OR incompeten* OR status OR continu* OR discontinu* OR stop* OR quit* OR cease* OR cessation))) OR all(((driving OR driver) NEAR/3 (licens* OR law* OR legal*))) OR all((driv* NEAR/3 (fit OR fitness)))

S2 all(((general or family) NEAR/2 (practi* or physician or doctor)) or gp or gps) OR ti(doctor OR physician) OR all("primary care" OR "primary healthcare" OR "primary health care")

S1 all("united kingdom" or uk or britain or gb or england or wales or scotland or "northern ireland") OR all(nhs OR "national health service")

A total of 131 citations were screened by title and abstract, 6 were screened in full text and none were included in the final synthesis.

Prevent (August 2017)

MEDLINE

For peer review only

#

▲ Searches

1 (prevent adj (program* or strateg* or initiative or policy)).ti,ab.

2

(prevent* and (terroris* or counterterroris* or antiterroris* or extremis* or counterextremis* or antiextremis* or radicali* or counterradicali* or antiradicali*)).ti.

3

(prevent* adj5 (terroris* or counterterroris* or antiterroris* or extremis* or counterextremis* or antiextremis* or radicali* or counterradicali* or antiradicali*)).ti,ab.

4 1 or 2 or 3

5 exp General Practice/

6 general practitioners/ or physicians, family/ or physicians, primary care/

7 Primary Health Care/

8 (((general or family) adj2 (practi* or physician? or doctor?)) or gp or gps).ti,ab.

9 (doctor? or physician?).ti.

10 (primary adj2 (care or healthcare)).ti,ab.

11 5 or 6 or 7 or 8 or 9 or 10

12 exp United Kingdom/

13

(united kingdom or uk or britain or gb or england or wales or scotland or northern ireland).ti,ab,in.

14 (nhs or national health service).ti,ab,in.

15

(british or bjgp or bmj or hsj or pulse or gp or general practi* or primary care or nursing standard or nursing times).jw.

16 12 or 13 or 14 or 15

17 4 and 11 and 16

Embase

#

▲ Searches

1 (prevent adj (program* or strateg* or initiative or policy)).ti,ab.

For peer review only

2

(prevent* and (terroris* or counterterroris* or antiterroris* or extremis* or counterextremis* or antiextremis* or radicali* or counterradicali* or antiradicali*)).ti.

3

(prevent* adj5 (terroris* or counterterroris* or antiterroris* or extremis* or counterextremis* or antiextremis* or radicali* or counterradicali* or antiradicali*)).ti,ab.

4 1 or 2 or 3

5 General Practice/

6 general practitioner/

7 Primary Medical Care/

8 (((general or family) adj2 (practi* or physician? or doctor?)) or gp or gps).ti,ab.

9 (doctor? or physician?).ti.

10 (primary adj2 (care or healthcare)).ti,ab.

11 5 or 6 or 7 or 8 or 9 or 10

12 exp United Kingdom/

13

(united kingdom or uk or britain or gb or england or wales or scotland or northern ireland).ti,ab,in.

14 (nhs or national health service).ti,ab,in.

15

(british or bjgp or bmj or hsj or pulse or gp or general practi* or primary care or nursing standard or nursing times).jw.

16 12 or 13 or 14 or 15

17 4 and 11 and 16

PsycINFO

▲ Searches

1 (prevent adj (program* or strateg* or initiative or policy)).ti,ab.

2

(prevent* and (terroris* or counterterroris* or antiterroris* or extremis* or counterextremis* or antiextremis* or radicali* or counterradicali* or antiradicali*)).ti.

3

For peer review only

(prevent* adj5 (terroris* or counterterroris* or antiterroris* or extremis* or counterextremis* or antiextremis* or radicali* or counterradicali* or antiradicali*)).ti,ab.

4 1 or 2 or 3

5 general practitioners/

6 Primary Health Care/

7 (((general or family) adj2 (practi* or physician? or doctor?)) or gp or gps).ti,ab.

8 (doctor? or physician?).ti.

9 (primary adj2 (care or healthcare)).ti,ab.

10 5 or 6 or 7 or 8 or 9

11

(united kingdom or uk or britain or gb or england or wales or scotland or northern ireland).ti,ab,in.

12 (nhs or national health service).ti,ab,in.

13

(british or bjgp or bmj or hsj or pulse or gp or general practi* or primary care or nursing standard or nursing times).jw.

14 11 or 12 or 13

15 4 and 10 and 14

CINAHL

S12 S3 AND S4 AND S11

S11 AB ( (prevent N1 (program* or strateg* or initiative or policy)) ) OR TI ( (prevent* and (terroris* or counterterroris* or antiterroris* or extremis* or counterextremis* or antiextremis* or radicali* or counterradicali* or antiradicali*)) ) OR AB ( (prevent* N5 (terroris* or counterterroris* or antiterroris* or extremis* or counterextremis* or antiextremis* or radicali* or counterradicali* or antiradicali*)) )

S4 (MH "United Kingdom+") OR ( AB "united kingdom" or uk or britain or gb or england or wales or scotland or "northern ireland" ) OR ( AB nhs or "national health service" ) OR ( SO british or bjgp or bmj or hsj or pulse or gp or general practi* or primary care or nursing standard or nursing times ) OR ( TI "united kingdom" or uk or britain or gb or england or wales or scotland or "northern ireland" ) OR ( TI nhs or "national health service" )

S3 S1 OR S2

For peer review only

S2 TI ( (((general or family) N2 (practi* or physician? or doctor?)) or gp or gps) ) OR AB ( (((general or family) N2 (practi* or physician? or doctor?)) or gp or gps) ) OR TI ( doctor? or physician? ) OR TI ( (primary N2 (care or healthcare)) ) OR AB ( (primary N2 (care or healthcare)) )

S1 (MH "Family Practice") OR (MH "Physicians, Family") OR (MH "Primary Health Care")

Web of Science (Core Collection)

# 8 14 #7 AND #2 AND #1

# 7 2,755 TS=((prevent NEXT (program* or strateg* or initiative or policy))) OR TI=((prevent* and (terroris* or counterterroris* or antiterroris* or extremis* or counterextremis* or antiextremis* or radicali* or counterradicali* or antiradicali*))) OR TS=((prevent* NEAR/5 (terroris* or counterterroris* or antiterroris* or extremis* or counterextremis* or antiextremis* or radicali* or counterradicali* or antiradicali*)))

# 2 5,132,695 TS=("united kingdom" or uk or britain or gb or england or wales or scotland or "northern ireland") OR ADDRESS: ("united kingdom" or uk or britain or gb or england or wales or scotland or "northern ireland") OR TS=(nhs or "national health service") OR ADDRESS: (nhs or "national health service") OR PUBLICATION NAME: (british or bjgp or bmj or hsj or pulse or gp or general practi* or primary care or nursing standard or nursing times)

# 1 339,275 TS=((((general or family) NEAR/2 (practi* or physician? or doctor?)) or gp or gps)) OR TI=(doctor? or physician?) OR TS=((primary NEAR/2 (care or healthcare)))

Proquest Social Science Databases

Set Search

S11 S1 AND S2 AND S10

S10 all((prevent NEXT (program* OR strateg* OR initiative OR policy))) OR ti((prevent* AND (terroris* OR counterterroris* OR antiterroris* OR extremis* OR counterextremis* OR antiextremis* OR radicali* OR counterradicali* OR antiradicali*))) OR all((prevent* NEAR/5 (terroris* OR counterterroris* OR antiterroris* OR extremis* OR counterextremis* OR antiextremis* OR radicali* OR counterradicali* OR antiradicali*)))

S2 all(((general or family) NEAR/2 (practi* or physician or doctor)) or gp or gps) OR ti(doctor OR physician) OR all("primary care" OR "primary healthcare" OR "primary health care")

S1 all("united kingdom" or uk or britain or gb or england or wales or scotland or "northern ireland") OR all(nhs OR "national health service")

For peer review only

A total of 22 citations were screened by title and abstract, 17 were screened in full text and 5 were included in the final synthesis.

IPV/DVA (August 2017)

MEDLINE

# ▲ Searches

1 domestic violence/ or spouse abuse/

2 ((domestic or spous* or partner? or wife or wives or husband) adj2 (violence or abuse*)).ti,ab.

3 (batter* adj2 (spous* or partner? or wife or wives or husband)).ti,ab.

4 1 or 2 or 3

5 exp General Practice/

6 general practitioners/ or physicians, family/ or physicians, primary care/

7 Primary Health Care/

8 (((general or family) adj2 (practi* or physician? or doctor?)) or gp or gps).ti,ab.

9 (doctor? or physician?).ti.

10 (primary adj2 (care or healthcare)).ti,ab.

11 5 or 6 or 7 or 8 or 9 or 10

12 exp United Kingdom/

13

(united kingdom or uk or britain or gb or england or wales or scotland or northern ireland).ti,ab,in.

14 (nhs or national health service).ti,ab,in.

15

(british or bjgp or bmj or hsj or pulse or gp or general practi* or primary care or nursing standard or nursing times).jw.

16 12 or 13 or 14 or 15

17 4 and 11 and 16

Embase

▲ Searches

1 domestic violence/ or battered woman/ or family violence/ or exp partner violence/

2 ((domestic or spous* or partner? or wife or wives or husband) adj2 (violence or abuse*)).ti,ab.

3 (batter* adj2 (spous* or partner? or wife or wives or husband)).ti,ab.

For peer review only

4 1 or 2 or 3

5 General Practice/

6 general practitioner/

7 Primary Medical Care/

8 (((general or family) adj2 (practi* or physician? or doctor?)) or gp or gps).ti,ab.

9 (doctor? or physician?).ti.

10 (primary adj2 (care or healthcare)).ti,ab.

11 5 or 6 or 7 or 8 or 9 or 10

12 exp United Kingdom/

13

(united kingdom or uk or britain or gb or england or wales or scotland or northern ireland).ti,ab,in.

14 (nhs or national health service).ti,ab,in.

15

(british or bjgp or bmj or hsj or pulse or gp or general practi* or primary care or nursing standard or nursing times).jw.

16 12 or 13 or 14 or 15

17 4 and 11 and 16

PsycINFO

▲ Searches

1 domestic violence/ or battered females/ or intimate partner violence/ or exp partner abuse/

2 ((domestic or spous* or partner? or wife or wives or husband) adj2 (violence or abuse*)).ti,ab.

3 (batter* adj2 (spous* or partner? or wife or wives or husband)).ti,ab.

4 1 or 2 or 3

5 general practitioners/

6 Primary Health Care/

7 (((general or family) adj2 (practi* or physician? or doctor?)) or gp or gps).ti,ab.

8 (doctor? or physician?).ti.

9 (primary adj2 (care or healthcare)).ti,ab.

10 5 or 6 or 7 or 8 or 9

11

For peer review only

(united kingdom or uk or britain or gb or england or wales or scotland or northern ireland).ti,ab,in.

12 (nhs or national health service).ti,ab,in.

13

(british or bjgp or bmj or hsj or pulse or gp or general practi* or primary care or nursing standard or nursing times).jw.

14 11 or 12 or 13

15 4 and 10 and 14

CINAHL

S10 S3 AND S4 AND S9

S9 S7 OR S8

S8 AB ( ((domestic or spous* or partner? or wife or wives or husband) N2 (violence or abuse*)) ) OR AB ( (batter* N2 (spous* or partner? or wife or wives or husband)) ) OR TI ( ((domestic or spous* or partner? or wife or wives or husband) N2 (violence or abuse*)) ) OR TI ( (batter* N2 (spous* or partner? or wife or wives or husband)) )

S7 (MH "Domestic Violence") OR (MH "Intimate Partner Violence")

S4 (MH "United Kingdom+") OR ( AB "united kingdom" or uk or britain or gb or england or wales or scotland or "northern ireland" ) OR ( AB nhs or "national health service" ) OR ( SO british or bjgp or bmj or hsj or pulse or gp or general practi* or primary care or nursing standard or nursing times ) OR ( TI "united kingdom" or uk or britain or gb or england or wales or scotland or "northern ireland" ) OR ( TI nhs or "national health service" )

S3 S1 OR S2

S2 TI ( (((general or family) N2 (practi* or physician? or doctor?)) or gp or gps) ) OR AB ( (((general or family) N2 (practi* or physician? or doctor?)) or gp or gps) ) OR TI ( doctor? or physician? ) OR TI ( (primary N2 (care or healthcare)) ) OR AB ( (primary N2 (care or healthcare)) )

S1 (MH "Family Practice") OR (MH "Physicians, Family") OR (MH "Primary Health Care")

Web of Science (Core Collection)

# 6 150 #5 AND #2 AND #1

# 5 15,568 TS=(((domestic or spous* or partner? or wife or wives or husband) NEAR/2 (violence or abuse*))) OR TS=((batter* NEAR/2 (spous* or partner? or wife or wives or husband)))

# 2 5,132,695 TS=("united kingdom" or uk or britain or gb or england or wales or scotland or

For peer review only

"northern ireland") OR ADDRESS: ("united kingdom" or uk or britain or gb or england or wales or scotland or "northern ireland") OR TS=(nhs or "national health service") OR ADDRESS: (nhs or "national health service") OR PUBLICATION NAME: (british or bjgp or bmj or hsj or pulse or gp or general practi* or primary care or nursing standard or nursing times)

# 1 339,275 TS=((((general or family) NEAR/2 (practi* or physician? or doctor?)) or gp or gps)) OR TI=(doctor? or physician?) OR TS=((primary NEAR/2 (care or healthcare)))

Proquest Social Science Databases

S8 S1 AND S2 AND S7

S7 all(((domestic or spous* or partner? or wife or wives or husband) NEAR/2 (violence or abuse*))) OR all((batter* NEAR/2 (spous* or partner? or wife or wives or husband)))

S2 all(((general or family) NEAR/2 (practi* or physician or doctor)) or gp or gps) OR ti(doctor OR physician) OR all("primary care" OR "primary healthcare" OR "primary health care")

S1 all("united kingdom" or uk or britain or gb or england or wales or scotland or "northern ireland") OR all(nhs OR "national health service")

A total of 352 citations were screened by title and abstract, 62 were screened in full text and 15 were included in the final synthesis.

Mandatory reporting

PubMed

Search Query

#29 Select 6 document(s) Filters: published in the last 10 years; English

#28 Search (((((relig* OR muslim* OR islam* or "far right") AND (extremis* OR terror* or radicalisation OR radicalization)) OR (((relig* OR muslim* OR islam* or "far right" OR extremis* OR terror* or radicalisation OR radicalization) AND ((prevent strateg*[Title/Abstract]) OR prevent program*[Title/Abstract])))) AND "last 10 years"[PDat] AND English[lang])) AND ((uk OR "united kingdom" OR gb OR britian OR england OR wales OR scotland OR "northern ireland" OR nhs OR british) AND "last 10 years"[PDat] AND English[lang]) Filters: published in the last 10 years; English

#27 Search uk OR "united kingdom" OR gb OR britian OR england OR wales OR scotland OR "northern ireland" OR nhs OR british Filters: published in the last 10 years;

English

#26 Search ((relig* OR muslim* OR islam* or "far right") AND (extremis* OR terror* or radicalisation OR radicalization)) OR (((relig* OR muslim* OR islam* or "far right" OR extremis* OR terror* or radicalisation OR radicalization) AND ((prevent strateg*[Title/Abstract]) OR prevent program*[Title/Abstract]))) Filters: published in the last 10 years; English

#24 Search (((((relig* OR muslim* OR islam* or "far right") AND (extremis* OR terror* or radicalisation OR radicalization))) AND ("last 10 years"[PDat] AND English[lang] AND (infant[MeSH] OR child[MeSH] OR adolescent[MeSH])))) OR (((((relig* OR muslim* OR islam* or "far right" OR extremis* OR terror* or radicalisation OR radicalization) AND ("last 10 years"[PDat] AND English[lang] AND (infant[MeSH] OR child[MeSH] OR adolescent[MeSH])))) AND (((prevent strateg*[Title/Abstract]) OR prevent program*[Title/Abstract]) AND ("last 10 years"[PDat] AND English[lang] AND (infant[MeSH] OR child[MeSH] OR adolescent[MeSH])))) AND ("last 10 years"[PDat] AND English[lang] AND (infant[MeSH] OR child[MeSH] OR adolescent[MeSH]))) Filters: published in the last 10 years; English

#23 Search (((((relig* OR muslim* OR islam* or "far right") AND (extremis* OR terror* or radicalisation OR radicalization))) AND ("last 10 years"[PDat] AND English[lang] AND (infant[MeSH] OR child[MeSH] OR adolescent[MeSH])))) OR (((((relig* OR muslim* OR islam* or "far right" OR extremis* OR terror* or radicalisation OR radicalization) AND ("last 10 years"[PDat] AND English[lang] AND (infant[MeSH] OR child[MeSH] OR adolescent[MeSH])))) AND (((prevent strateg*[Title/Abstract]) OR prevent program*[Title/Abstract]) AND ("last 10 years"[PDat] AND English[lang] AND (infant[MeSH] OR child[MeSH] OR adolescent[MeSH])))) AND ("last 10 years"[PDat] AND English[lang] AND (infant[MeSH] OR child[MeSH] OR adolescent[MeSH]))) Filters: published in the last 10 years; English; Child: birth-18 years

#22 Search (((relig* OR muslim* OR islam* or "far right" OR extremis* OR terror* or radicalisation OR radicalization) AND ("last 10 years"[PDat] AND English[lang] AND (infant[MeSH] OR child[MeSH] OR adolescent[MeSH])))) AND (((prevent strateg*[Title/Abstract]) OR prevent program*[Title/Abstract]) AND ("last 10

For peer review only

years"[PDat] AND English[lang] AND (infant[MeSH] OR child[MeSH] OR adolescent[MeSH]))) Filters: published in the last 10 years; English; Child: birth-18 years

#21 Search relig* OR muslim* OR islam* or "far right" OR extremis* OR terror* or radicalisation OR radicalization Filters: published in the last 10 years; English; Child: birth-18 years

#20 Search (prevent strateg*[Title/Abstract]) OR prevent program*[Title/Abstract] Filters: published in the last 10 years; English; Child: birth-18 years

#19 Search ((relig* OR muslim* OR islam* or "far right") AND (extremis* OR terror* or radicalisation OR radicalization)) Filters: published in the last 10 years; English; Child: birth-18 years

#15 Search "moral imperative" OR "ethical imperative" OR "emotional imperative" Filters: published in the last 10 years; English; Child: birth-18 years

#14 Search "moral imperative" OR "ethical imperative" OR "emotional imperative" Filters: published in the last 10 years; Child: birth-18 years

#13 Search "moral imperative" OR "ethical imperative" OR "emotional imperative" Filters: Child: birth-18 years

#12 Search ((mandatory report*[tiab] OR mandated report*[tiab] OR mandatory notif*[tiab] OR mandated notif*[tiab])) OR (mandat*[ti] AND (report*[ti] OR notif*[ti])) Filters: Child: birth-18 years

#10 Search ((mandatory report*[tiab] OR mandated report*[tiab] OR mandatory notif*[tiab] OR mandated notif*[tiab])) OR (mandat*[ti] AND (report*[ti] OR notif*[ti]))

#11 Search ((mandatory report*[tiab] OR mandated report*[tiab] OR mandatory notif*[tiab] OR mandated notif*[tiab])) OR (mandat*[ti] AND (report*[ti] OR notif*[ti])) Filters: Systematic Reviews

#9 Search mandat*[ti] AND (report*[ti] OR notif*[ti])

#8 Search mandatory report*[tiab] OR mandated report*[tiab] OR mandatory notif*[tiab] OR mandated notif*[tiab]

#7 Search (((physician*[Title] OR doctor*[Title] OR general practi*[Title] OR family physician*[Title] OR "primary care"[Title]))) AND ((educat*[Title] OR learn*[Title]

OR teach*[Title] OR train*[Title])) Filters: Systematic Reviews; published in the last 10 years; English

#6 Search (((physician*[Title] OR doctor*[Title] OR general practi*[Title] OR family physician*[Title] OR "primary care"[Title]))) AND ((educat*[Title] OR learn*[Title] OR teach*[Title] OR train*[Title])) Filters: Systematic Reviews; English

#5 Search (((physician*[Title] OR doctor*[Title] OR general practi*[Title] OR family physician*[Title] OR "primary care"[Title]))) AND ((educat*[Title] OR learn*[Title] OR teach*[Title] OR train*[Title])) Filters: Systematic Reviews

#4 Search (((physician*[Title] OR doctor*[Title] OR general practi*[Title] OR family physician*[Title] OR "primary care"[Title]))) AND ((educat*[Title] OR learn*[Title] OR teach*[Title] OR train*[Title]))

#3 Search (educat*[Title] OR learn*[Title] OR teach*[Title] OR train*[Title])

#2 Search (physician*[Title] OR doctor*[Title] OR general practi*[Title] OR family physician*[Title] OR "primary care"[Title])

Web of Science (Core Collection)

# 29 8 #25 AND #23

# 28 88 #27 AND #10

# 27 521 #23 AND #21

# 26 63 TS=("prevent strateg*" OR "prevent program*")

# 25 165 TS=("prevent strateg*" OR "prevent program*")

# 24 521 #23 AND #21

# 23 1,576,775 TS=(physician* OR doctor* OR "general practi" OR "family practi*" OR "primary care" OR healthcare OR "health care") OR TI=(health* OR medicine* OR nurs*)

# 22 2 #21 AND #12

# 21 5,487 #20 OR #19

# 20 1,960 TS=((relig* OR muslim* OR islam* or "far right") NEAR/5 (extremis* OR terror* or radicalisation OR radicalization))

# 19 3,531 TS=("home office" OR "illegal migrant*" OR "illegal immigra*" OR "undocumented migran*" OR "undocumented immigran*" OR illegals)

# 18 363 #15 OR #13 Refined by: PUBLICATION YEARS: ( 2018 OR 2008 OR 2017 OR

For peer review only

2016 OR 2015 OR 2014 OR 2013 OR 2012 OR 2011 OR 2010 OR 2009 ) AND [excluding] DOCUMENT TYPES: ( EDITORIAL MATERIAL OR MEETING ABSTRACT OR BOOK REVIEW OR LETTER OR NEWS ITEM OR PROCEEDINGS PAPER )

# 17 405 #15 OR #13 Refined by: PUBLICATION YEARS: ( 2018 OR 2008 OR 2017 OR 2016 OR 2015 OR 2014 OR 2013 OR 2012 OR 2011 OR 2010 OR 2009 )

# 16 597 #15 OR #13

# 15 321 #14 AND #12

# 14 64,467 TS=((child* OR infant* OR teen* OR adolescen*) NEAR/3 (abuse* OR violence OR maltreat*)) OR TI=((child* OR infant* OR teen* OR adolescen*) AND (abuse* OR violence OR maltreat*)) OR TS=(safeguarding OR "safe guarding")

# 13 290 #12 AND #10

# 12 2,582 TOPIC: ((mandat* OR compulsory) NEAR/3 (report* OR notif*)) OR TITLE: ((mandat* OR compulsory) AND (report* OR notif*))

# 11 64 #10 AND #9

# 10 5,653,563 TOPIC: ("united kingdom" or uk or britain or british or gb or england or "northern ireland" or scotland or wales or nhs) OR ADDRESS: ("united kingdom" or uk or britain or british or gb or england or "northern ireland" or scotland or wales or nhs)

# 9 787 TOPIC: ("cultural competenc*") AND TOPIC: (physician* OR doctor* OR "general practi*" OR "family practi" OR "primary care")

# 8 128 TITLE: ("cultural competenc*") AND TOPIC: (educat* OR learn* OR teach* OR train*) AND TOPIC: (physician* OR doctor* OR "general practi*" OR "family practi" OR "primary care")

# 7 179 TOPIC: ("moral imperative" OR "ethical imperative" OR "emotional imperative") AND TOPIC: (educat* OR learn* OR teach* OR train*)

# 6 4 TOPIC: ("moral imperative" OR "ethical imperative" OR "emotional imperative") AND TOPIC: ((child* OR adolecen* OR teen* OR infan*) NEAR/5 (abuse* OR violence OR maltreat*))

# 5 0 TITLE: ("moral imperative" OR "ethical imperative" OR "emotional

For peer review only

imperative") AND TOPIC: ((child* OR adolecen* OR teen* OR infan*) NEAR/5 (abuse* OR violence OR maltreat*))

# 4 287 TITLE: ("moral imperative" OR "ethical imperative" OR "emotional imperative")

# 3 1 TOPIC: ("moral imperative" OR "ethical imperative" OR "emotional imperative") AND TOPIC: (((mandat* OR compulsory) NEAR/2 (report* OR notif*)))

# 2 55 TOPIC: ("moral imperative" OR "ethical imperative" OR "emotional imperative") AND TOPIC: (doctor* OR physician* OR "general practi*" OR "family practi*")

# 1 891 TOPIC: ("moral imperative" OR "ethical imperative" OR "emotional imperative")

A total of 88 citations were screened by title and abstract, 36 were screened in full text and 10 were included in the final synthesis.

For peer review only

## Appendix D. Full set of derived CMOC/full programme theory.

**The need for FGM knowledge and awareness:**

1) Lacking knowledge or skills (including about the cultural contexts of FGM, safeguarding requirements, who might be affected, and the different types of FGM and their clinical consequences) impacts on GPs' ability to provide optimal care for women affected by FGM (1-7) (8, 9). Practitioners may not be aware that they lack knowledge, including which patients may be affected and their care needs (C). This lack of knowledge (M) results in their inability to meet their care needs. (O) (10-21) (22) (23).
2) Lacking the necessary knowledge and skills (C), is associated with a lack of confidence (M) which impacts on clinical care for women with FGM (O) (10, 24, 25). This includes having the knowledge and confidence to consider who may be at risk (26).
3) Feeling that they have adequate knowledge (including how to respond to a disclosure) (C) helped clinicians feel confident (M) to ask to ask (O) (27, 28).
4) Women who perceive healthcare professionals lack knowledge and skills to manage FGM (C), or who have experiences stigma (C) may lack confidence that health services (M) will meet their care needs (O) (5, 24, 29-32) (33) (10).
5) (C)Health professionals experience emotional reactions to encountering FGM such as anger, shock, and pity (34-37); they find encountering FGM without adequate knowledge is "frightening"(35)(M). Professionals try to hide their reactions but are aware their reaction may be apparent to the women (23) (O).
6) Community members see health care providers reacting with shock or horror to their FGM (C). This provokes feelings of shame (M) which reduces their likelihood of accessing services (O) (38-41) (22) (40) (42).
7) However, clinicians confident in managing FGM (C) are able to reassure women (M) and meet their care needs (O) (28, 43, 44).
8) Healthcare professionals may experience a strong emotional responses to FGM (C). This may make them feel panicked or frightened (M) and abandon their usual routines and practices (O) (28, 37).
9) FGM is usually a relatively small part of the GP workload (C) (45). GPs may not identify learning about FGM as a priority (M) for themselves. The GPs then lack knowledge and skills (O)(46).
10) Clinicians who encounter FGM more frequently in the line of their work (C), may become sensitised to FGM (M) and motivated (M) to learn more or develop their knowledge and skills (O). The converse may also be true (8, 12, 14, 47).
11) FGM can be difficult to correctly identify (48), especially types 1 and 4 and associated with less symptoms (20, 49-51) (C) and GPs may not have the expertise or confidence (M) to correctly identify or manage FGM (O) (52, 53).

**Talking about FGM and communication:**

12) A key skill GPs need is being able to talk about FGM sensitively (10, 21, 54). Fears (M) of offending women by not knowing how to raise the issue(C) can lead GPs to avoid talking about FGM (O) (4, 5, 16, 23, 29, 35, 48, 55-57) (22) (56).
13) Professionals who understand that FGM can be sensitive or taboo subject (C) (29, 58, 59) may be fearful of offending women (M) and avoid discussing FGM with women (O) (54, 55) (23). This contextual factor may be evolving as community attitudes towards the practice of FGM change including meaning that talking about FGM is less taboo in some communities (37, 60-64).
14) Not being aware of or not recognising evolving community practices (C) risks (M) offending community members thereby reducing effective communication/consultations (O) (37). Or that professionals are not able to accurately appraise risk (11) (61).
15) Raising FGM in a way that is normalised within the consultation, for example as a standard question on an assessment form (C) may reduce the embarrassment (M) and facilitate asking (O) (23). Prompts in the records may help clinicians to do this (65) (66).

16) Challenges around the use of terminology can complicate communication between GPs and their patients affected by FGM. Some women may find the terms FGM offensive or frightening (67) (68), or if the term FGM is not familiar to the woman (C) (69), or she does not align her cultural practice (for example labial elongation) with FGM (C) (70), then she may not relate her experience to FGM (M) or know how to reply if a GP asks her about FGM(O) (O) (71).
17) Women's experiences of poor communication and difficulties in engagement with health professionals (e.g. language, cultural differences, perceived judgement), and including non-verbal communication (72), led to them feeling not understood or respected (M), causing a lack of confidence and trust in health services (O) (21, 29, 30, 39, 72, 73) (68).
18) Women who feel pitied or judged(C) may be reluctant (M) to make a disclosure to a health care professional (O) (74, 75).
19) Members of communities affected by FGM acting as health advocates (C) may help promote trust and educate communities and professionals (M) to facilitate access to services (O) (76-78).
20) Language barriers and a lack of understanding of how the health setting works, including communicating with primary care receptionists (C) can make accessing services difficult or stressful (M) and lead to avoidance (O) (79).
21) Whether FGM is relevant to the health concern which the woman brings to her GP appointment (C), could impact on whether the GP or woman are willing to raise or discuss FGM (M), and how such a conversation may be received or experienced (O) (36, 55, 80). This may apply when GPs consider asking women about FGM to consider safeguarding needs within their families, rather than because of their own health needs (37) (81) (82).
22) Coding FGM into medical records introduces potential tensions around balancing the needs of the woman (and her confidentiality) with the potential need of her family (C) which may cause confusion or uncertainty for GPs (M), and lead to improvised strategies or inconsistent coding (O) (82, 83).
23) Women who perceive that the HCP is preoccupied with their FGM (C), can feel disrespected (M) and disengage with health care settings (O) (60) (84, 85) .
24) GPs gender (C) may influence whether the woman or GP feel it is culturally appropriate (M) to talk about FGM (O) (38, 55, 86) (23).
25) Time pressures in the consultation (C) mean GPs may be reluctant (M) to discuss FGM (O). (27, 46, 55, 87)
26) Language barriers are a significant context which influence the conversations between GPs and women about FGM (C) which reduce communication (M) between women and GPs with impacts on communication effectiveness and their care (O) (4, 17, 21, 29, 55, 57, 58, 79, 86, 88) (23).
27) Strategies to address language barriers add their own complications. Official interpreters are recommended, but may not be available(89) or trusted by women, for example if they both perceive FGM as taboo, or she fears they will not respect her confidentiality. This can lead to fear (M) and reduced engagement with health professionals (O) (4, 6, 17, 36, 56, 90) (91) (23) (38).
28) The presence of family members (as interpreters, or witnesses) in the consultation (C) may inhibit GPs feeling able to raise FGM (O) with the women, because of concerns about privacy and confidentiality (M) (4, 17, 55, 92).

**The need for guidelines and access to specialist services:**

29) Researchers and commentators suggest that having access to clear and supportive guidelines about what clinicians should do (C) will enable professionals (M) to ask women about FGM and optimise their care (O)(19, 20, 93, 94) (35). Even when guidelines exist, awareness of them may be incomplete, or they may not be followed, as demonstrated by four UK hospital studies (47, 56, 57, 95). The reasons for this in the case of FGM warrant exploration. Having prompts to normalise asking about

FGM may help clinicians broach the subject (96), especially if linked to training or referral pathways(97).

30) FGM is a complex area for health care professionals to manage, and this management may include needing to report women and their families to other authorities. If professionals are developing awareness of FGM, without accompanying guidance (C), they may experience uncertainty and face what they experience as ethical tensions (M) and risk making incorrect or uncertain decisions regarding reporting (O) (93, 94)
31) Lacking guidance, including guidelines and certainty about what good care comprises (C) can lead to practitioners feeling uncertain (M) and improvising how they offer care (O) (22, 23).
32) Knowing how to react or having access to specialist services (C) may help GPs and community members feel confident (M) to talk about FGM (O) (29, 52, 53, 73, 98).
33) When Health care professionals speak about FGM within a framework of offering support and services (C), it is more likely to be experienced as acceptable by the woman (M, O) (64).
34) Training is more likely to be effective (C) in changing behaviour and promoting asking (O) when it is supported by resources and referral pathways or protocol for intervention (M) (28, 97, 99, 100). Specialist access may be especially important to support practitioners in low prevalence areas (23).

**Mandated actions including mandatory reporting and the FGM Enhanced dataset requirements:**

35) The mandatory reporting duty (C) may cause distress (101)and reduce trust (M) in professionals which may deter women from seeking help or disclosing their FGM (O) (52, 55, 60, 64, 102, 103).
36) Concerns that medical encounters or records are not confidential may cause fear/apprehension (M) and deter women from a disclosure of her needs or concerns (O) (92, 104).
37) The requirement to submit personally identifiable data to the FGM enhanced dataset (C) may reduce women's trust in the confidentiality of the GP consultation (M) and make her reluctant to disclose FGM (O) or make GPs reluctant to raise FGM (O) because of concerns about confidentiality (M) (55, 76, 105-108).
38) The ways in which mandatory reporting or the enhanced dataset are raised in the consultation, including when this happens repeatedly (C), may lead women to feel that the professionals' interest in more in data collection than them, or make them feel judged or fearful (M), and avoid attending healthcare altogether (O) (60)
39) The concern (M) that making a mandated report (C) would have a potentially negative impact on trust (O) in on-going professional relationships (O) was an important potential consideration for professionals (109, 110) (111) (112), and identified as a potential deterrent for help seeking (113).
40) Another concern about managing the legislative requirements includes that FGM can be difficult to identify on examination. GPs may feel that they do not have the skills needed to identify or manage FGM and so not feel confident in being able to identify FGM to confidently code it (O)(68) (46), which may impact on data accuracy (O) (114). Education (C) is needed to help practitioners feel able (M) to approach mandatory reporting (O) (124).
41) Practitioners making mandated reports need to feel confident that their report will be adequately responded to, without causing harm (115) (116). They may be helped by training (115).
42) In addition to lack of knowledge or training (C), practitioners may have concerns about confidentiality (C/M) or fear of causing stigma (C/M) which leads to incomplete or inaccurate coding of FGM (117). Practitioners may perceive a need to feel certain (C) before making a mandated report (O) so that they do not risk making a mistake (M) (118) (119).
43) When young people know that the professional whom they are speaking to is mandated to share the information with other authorities (C), they may feel more reluctant to trust the professional (M), and less likely to make a disclosure (O) (120).
44) A Perceptions of how trustworthy the authority being referred onto may contribute to decisions as to whether or not to disclose. Those who are potentially fearful of authorities or perceive themselves to be vulnerable (C), for example those with uncertain migrant status (C), or if they fear that their

disclosure risks placing others at risk of trouble (c), may be more fearful of mandatory reporting or data sharing (M) and avoid accessing services (O) (109) (121-123). (125)


1. Khaja K, Lay K, Boys S. Female Circumcision: Toward an Inclusive Practice of Care. Health Care for Women International. 2010;31(8):686-99.
2. Leval A, Widmark C, Tishelman C, Ahlberg BM. THE ENCOUNTERS THAT RUPTURE THE MYTH: CONTRADICTIONS IN MIDWIVES' DESCRIPTIONS AND EXPLANATIONS OF CIRCUMCISED WOMEN IMMIGRANTS' SEXUALITY. Health Care for Women International. 2010;25(8):743-60.
3. Odemerho BI, Baier M. Female Genital Cutting and the Need for Culturally Competent Communication. The Journal for Nurse Practitioners. 2012;8(6):452-7.
4. Lazar JN, Johnson-Agbakwu CE, Davis OI, Shipp MPL. Providers' Perceptions of Challenges in Obstetrical Care for Somali Women. Obstetrics and Gynecology International. 2013;2013:149640.
5. Johnson-Agbakwu CE, Helm T, Killawi A, Padela AI. Perceptions of obstetrical interventions and female genital cutting: insights of men in a Somali refugee community. Ethnicity and Health. 2014;19(4):440-57.
6. Byrskog U, Olsson P, Essén B, Allvin M-K. Being a bridge: Swedish antenatal care midwives' encounters with Somali-born women and questions of violence; a qualitative study. BMC Pregnancy and Childbirth. 2015;15(1).
7. Varol N, Hall JJ, Black K, Turkmani S, Dawson A. Evidence-based policy responses to strengthen health, community and legislative systems that care for women in Australia with female genital mutilation / cutting. Reproductive Health. 2017;14(1):63.
8. Tantet C, Aupiais C, Bourdon M, Sorge F, Pagès A, Levy D, et al. Female genital mutilation: an evaluation of the knowledge of French general and specialized travel medicine practitioners. Journal of Travel Medicine. 2018;25(1):tax090.
9. González-Timoneda A, Ros VR, González-Timoneda M, Sánchez AC. Knowledge, attitudes and practices of primary healthcare professionals to female genital mutilation in Valencia, Spain: are we ready for this challenge? BMC health services research. 2018;18(1):579.
10. Vangen S, Johansen REB, Sundby J, Træen B, Stray-Pedersen B. Qualitative study of perinatal care experiences among Somali women and local health care professionals in Norway. European Journal of Obstetrics & Gynecology and Reproductive Biology. 2004;112(1):29-35.
11. Tamaddon L, Johnsdotter S, Liljestrand J, Essén B. Swedish Health Care Providers' Experience and Knowledge of Female Genital Cutting. Health Care for Women International. 2006;27(8):709-22.
12. Kaplan-Marcusan A, Torán-Monserrat P, Moreno-Navarro J, Fàbregas MJC, Muñoz-Ortiz L. Perception of primary health professionals about Female Genital Mutilation: from healthcare to intercultural competence. BMC Health Services Research. 2009;9(11).
13. Leye E, Ysebaert I, Deblonde J, Claeys P, Vermeulen G, Jacquemyn Y, et al. Female genital mutilation: Knowledge, attitudes and practices of Flemish gynaecologists. The European Journal of Contraception and Reproductive Health Care. 2009;13(2):182-90.
14. Zaidi N, Khalil A, Roberts C, Browne M. Knowledge of female genital mutilation among healthcare professionals. Journal of Obstetrics and Gynecology. 2009;27(2):161-4.
15. Relph S, Inamdar R, Singh H, Yoong W. Female genital mutilation/cutting: knowledge, attitude and training of health professionals in inner city London. European Journal of Obstetrics & Gynecology and Reproductive Biology. 2013;168(2):195-8.
16. Cappon S, L'Ecluse C, Clays E, Tency I, Leye E. Female genital mutilation: Knowledge, attitude and practices of Flemish midwives. Midwifery. 2015;31(3):e29-e35.
17. Dawson A, Homer CS, Turkmani S, Black K, Varol N. A systematic review of doctors' experiences and needs to support the care of women with female genital mutilation. International Journal of Gynecology & Obstetrics. 2015;131(1):35-40.


For peer review only

18. Dawson A, Turkmani S, Fray S, Nanayakkara S, Varol N, Homer C. Evidence to inform education, training and supportive work environments for midwives involved in the care of women with female genital mutilation: A review of global experience. Midwifery. 2015;31(1):229-38.
19. Zurynski Y, Sureshkumar P, Phu A, Elliott E. Female genital mutilation and cutting: a systematic literature review of health professionals' knowledge, attitudes and clinical practice. BMC international health and human rights. 2015;15:32.
20. Reig-Alcaraz M, Siles-González J, Solano-Ruiz C. A mixed-method synthesis of knowledge, experiences and attitudes of health professionals to Female Genital Mutilation. Journal of Advanced Nursing. 2015;72(2):245-60.
21. Smith H, Stein K. Health information interventions for female genital mutilation. Int ernational Journal of Gynecology and Obstetrics. 2017;136:(136:):79-82.
22. Jordal M, Wahlberg A. Challenges in providing quality care for women with female genital cutting in Sweden–A literature review. Sexual & Reproductive Healthcare. 2018;17:91-6.
23. Evans C, Tweheyo R, McGarry J, Eldridge J, Albert J, Nkoyo V, et al. Crossing cultural divides: A qualitative systematic review of factors influencing the provision of healthcare related to female genital mutilation from the perspective of health professionals. PloS one. 2019;14(3):e0211829.
24. Dawson AJ, Turkmani S, Varol N, Nanayakkara S, Sullivan E, Homer CS. Midwives' experiences of caring for women with female genital mutilation: Insights and ways forward for practice in Australia. Women and Birth. 2015;28(3):207-14.
25. Nash E, Ranka P, editors. FEMALE GENITAL MUTILATION: KNOWLEDGE, CONFIDENCE, AND APPROACH TO CARE IN CLINICAL PRACTICE OF MIDWIVES AND NURSES IN THE UK. 21st FIGO World Congress of Gynecology and Obstetrics; 2015; Vancouver, BC Canada: International Journal of Gynecology and Obstetrics.
26. Ramsay J, Rutterford C, Gregory A, Dunne D, Eldridge S, Sharp D, et al. Domestic violence: knowledge, attitudes, and clinical practice of selected UK primary healthcare clinicians. British Journal of General Practice. 2012;62(602):e647-e55.
27. Feder KH, Angela T, Gene. Violence between intimate partners: working with the whole family. 2008.
28. Sundborg E, Törnkvist L, Saleh-Stattin N, Wändell P, Hylander I. To ask, or not to ask: the hesitation process described by district nurses encountering women exposed to intimate partner violence. Journal of Clinical Nursing. 2017;26(15-16):2256-65.
29. Norman K, Hemmings J, Hussein E, Otoo-Oyortey N. FGM is always with us: Experiences, Perceptions and Beliefs of Women Affected by Female Genital Mutilation in London. Results from a PEER study London: Options Consultancy Services Ltd

FORWARD; 2009.
30. Hussein E, FORWARD. Women's Experiences, perceptions, and attitudes of female Genital Mutilation

The Bristol PEER study FORWARD; 2010.
31. Glover J, Liebling H, Barrett… H. The psychological and social impact of female genital mutilation: A holistic conceptual framework. 2017.
32. Norman K, Belay Gegzabher S, Otoo-Oyortey N. "Between Two Cultures": A Rapid PEER Study Exploring Migrant Communities' Views on Female Genital Mutilation in Essex and Norfolk, UK. London: National FGM Centre

FORWARD; 2016.
33. Mbanya VN, Gele AA, Diaz E, Kumar B. Health care-seeking patterns for female genital mutilation/cutting among young Somalis in Norway. BMC Public Health. 2018;18(1):517.
34. Ogunsiji O. Female Genital Mutilation (FGM): Australian Midwives' Knowledge and Attitudes. Health Care for Women International. 2015;36(11):1179-93.
35. Widmark C, Tishelman C, Ahlberg BM. A study of Swedish midwives' encounters with infibulated African women in Sweden. Midwifery. 2002;18(2):113-25.

36. Horowitz CR, Jackson JC. Female “circumcision” African women confront American medicine. Journal of General Internal Medicine. 1997;12(8):491-9.
37. Johnsdotter S, Essén B. Cultural change after migration: Circumcision of girls in Western migrant communities. Best Practice & Research Clinical Obstetrics & Gynaecology. 2016;32:15-25.
38. Abdullahi A, Copping J, Kessel A, Luck M, Bonell C. Cervical screening: Perceptions and barriers to uptake among Somali women in Camden. Public Health. 2009;123(10):680-5.
39. Oguntoye S, Otoo-Oyortey N, Hemmings J, Norman K, Hussein E. “FGM is with us Everyday”: Women and Girls Speak out about Female Genital Mutilation in the UK. World Academy of Science, Engineering and Technology. 2009;54:1020-5.
40. Hamid A, Grace KT, Warren N. A Meta-Synthesis of the Birth Experiences of African Immigrant Women Affected by Female Genital Cutting. Journal of Midwifery & Women's Health. 2018;63(2):185-95.
41. Recchia N, McGarry J. “Don’t judge me”: narratives of living with FGM. International Journal of Human Rights in Healthcare. 2017;10(1):4-13.
42. del Mar Pastor-Bravo M, Almansa-Martínez P, Jiménez-Ruiz I. Living with mutilation: A qualitative study on the consequences of female genital mutilation in women's health and the healthcare system in Spain. Midwifery. 2018;66:119-26.
43. Vloeberghs E, van der Kwaak A, Knipscheer J, van den Muijsenbergh M. Coping and chronic psychosocial consequences of female genital mutilation in the Netherlands. Ethnicity and Health. 2013;17(6):677-95.
44. Moxey JM, Jones LL. A qualitative study exploring how Somali women exposed to female genital mutilation experience and perceive antenatal and intrapartum care in England. BMJ Open. 2016;6(1):e0009846.
45. Baillot H, Murray N, Connelly E, Howard N. Addressing female genital mutilation in Europe: a scoping review of approaches to participation, prevention, protection, and provision of services. International Journal for Equity in Health. 2018;17(1):21.
46. Baillot H, Murray N, Connelly E, Howard N. Tackling Female Genital Mutilation in Scotland: A Scottish Model of Intervention. Scotland: Scottish Refugee Council

London School of Hygiene and Tropical Medicine 2014.
47. Purchase T, Lamoudi M, Colman S, Allen S, Latthe P, Jolly K. A survey on knowledge of female genital mutilation guidelines - Purchase - 2013 - Acta Obstetricia et Gynecologica Scandinavica - Wiley Online Library. ACTA Obstetricia et Gynecologica Scandinavia. 2013(92:):858–86.
48. Abdulcadir J, Dugerdil A, Boulvain M, Yaron M, Margairaz C, Irion O, et al. Missed opportunities for diagnosis of female genital mutilation. International Journal of Gynecology & Obstetrics. 2014;125(3):256-60.
49. Hodes D, Armitage A, Robinson K, Creighton SM. Female genital mutilation in children presenting to a London safeguarding clinic: a case series. Archives of Disease in Childhood. 2016;101(3):212-6.
50. Hodes D, Armitage A, Dykes A, editors. Female genital mutilation in London and the UNICEF report; a local perspective on worldwide statistics. Annual Conference of the Royal College of Paediatrics and Child Health, RCPCH 2014; 2014 2014-04-01; Birmingham, UK: Archives of Disease in Childhood.
51. Ayadi O’Donnell N, Pall K, Leoni M, Debelle G, Lynn R, Armitage A, et al., editors. Female genital mutilation (FGM) surveillance in under 16 years olds in the UK and Ireland. Royal College of Paediatrics and Child Health Annual Conference, RCPCH 2018; 2018 2018-03-01; United Kingdom: Archives of Disease in Childhood.
52. Mathers N, Rymer J. Mandatory reporting of female genital mutilation by healthcare professionals. British Journal of General Practice. 2015;65(635):282-3.
53. RCGP. Female Genital Mutilation : a clinical approach for GPs 2017 [updated under review 8.3.2017. Available from: http://www.rcgp.org.uk/policy/rcgp-policy-areas/female-genital-mutilation.aspx.

54. Johansen REB. Care for Infibulated Women Giving Birth in Norway: An Anthropological Analysis of Health Workers' Management of a Medically and Culturally Unfamiliar Issue. Medical Anthropology Quarterly. 2006;20(4):516-44.
55. Clayton-Hathaway K. A pilot evaluation of health services for communities affected by FGM/C in Oxfordshire. Oxfordshire: Oxford Against Cutting

Healthwatch Oxfordshire; 2016.
56. Zenner N, Liao LM, Richens Y, Creighton SM. Quality of obstetric and midwifery care for pregnant women who have undergone female genital mutilation. Journal of Obstetrics and Gynecology. 2013;33(5):459-62.
57. Gabrasadig R, Asamoah F, Wilson N, editors. Female genital mutilation: knowledge, training and experience of healthcare professionals at a London hospital. Annual Conference of the Royal College of Paediatrics and Child Health, RCPCH 2015; 2015 2015-04-01; Birmingham, UK: Archives of Disease in Childhood.
58. Upvall MJ, Mohammed K, Dodge PD. Perspectives of Somali Bantu refugee women living with circumcision in the United States: A focus group approach. International Journal of Nursing Studies. 2009;46(3):360-8.
59. Safari F. A qualitative study of women's lived experience after deinfibulation in the UK. Midwifery. 2013;29(2):154-8.
60. Karlsen S, Carver N, Mogilnicka M, Pantazis C. When safeguarding becomes stigmatising: A report on the impact of FGM-safeguarding procedures on people with a Somali heritage living in Bristol. Bristol: University of Bristol; 2019.
61. Creighton SM, Samuel Z, Otoo-Oyortey N, Hodes D. Tackling female genital mutilation in the UK. BMJ. 2019;364:l15.
62. FORWARD. "A Big Wake-Up Call": Participatory Study on Shifts in Attitudes Towards FGM Amongst Community Women in Bristol, Summary Report. FORWARD

Refugee Women of Bristol; 2017.
63. Brown E, Porter C. The Tackling FGM Initiative: Evaluation of the Second Phase

(2013-2016). London: Options Consultancy Services Ltd; 2016.
64. Brown E, Porter C, Unit OP. Evaluation of FGM Prevention among Communities Affected by FGM: A Participatory Ethnographic Evaluation Research (PEER) Study. Endline Phase 2. Options Consultancy Services Ltd; 2016.
65. Horwood J, Morden A, Bailey JE, Pathak N, Feder G. Assessing for domestic violence in sexual health environments: a qualitative study. Sex Transm Infect. 2018;94(2):88-92.
66. Jewkes RK. Preventing domestic violence: most women welcome inquiries, but doctors and nurses rarely ask about it. BMJ : British Medical Journal. 2002;324(7332):253-4.
67. Mohamed N, Schickler P, Warsame Z, Glew C. 'Hear Our Voices': A Report on Participatory Workshops on FGM/C (Female Genital Mutilation/Cutting) with the Somali Community in Tower Hamlets. London: Women's Health and Family Services; 2014.
68. Turkmani S, Homer C, Varol N, Dawson A. A survey of Australian midwives' knowledge, experience, and training needs in relation to female genital mutilation. Women and Birth. 2018;31(1):25-30.
69. Ajibona A, editor The understanding of the term female genital mutilation or FGM amongst patients with FGM in a United Kingdom inner city ante-natal clinic. 21st FIGO World Congress of Gynecology and Obstetrics; 2015; Vancouver, BC Canada: International Journal of Obstetrics and Gynaecology.
70. Ariyo D, Ssali R, King-Webb L, Ikpaahindi S. Voices of the Community:

Exploring Female Genital Mutilation in the African Community

across Greater Manchester. Afruca; 2015.

For peer review only

71. Brown K, Beecham D, Barrett H. The Applicability of Behaviour Change in Intervention Programmes Targeted at Ending Female Genital Mutilation in the EU: Integrating Social Cognitive and Community Level Approaches. Obstetrics and Gynecology International. 2013;2013(2013):324362.
72. Straus L, McEwen A, Hussein FM. Somali women's experience of childbirth in the UK: Perspectives from Somali health workers. Midwifery. 2009;25(2):181-6.
73. Glover J, Liebling H, Barrett H, Goodman S. The psychological and social impact of female genital mutilation: A holistic conceptual framework. Journal of International Studies. 2017;10(2):219-38.
74. Feder G, Wathen CN, MacMillan HL. An evidence-based response to intimate partner violence: WHO guidelines. JAMA. 2013;310(5):479-80.
75. Spangaro J, Koziol-McLain J, Zwi A, Rutherford A, Frail M-A, Ruane J. Deciding to tell: qualitative configurational analysis of decisions to disclose experience of intimate partner violence in antenatal care. Social Science & Medicine. 2016;154:45-53.
76. Dixon S, Agha K, Ali F, El Hindi L, Kelly B, Locock L, et al. Female genital mutilation in the UK- where are we, where do we go next? Involving communities in setting the research agenda. Research Involvement and Engagement. 2018;4(29).
77. Connelly E, Murray N, Baillot H, Howard N. Missing from the debate? A qualitative study exploring the role of communities within interventions to address female genital mutilation in Europe. BMJ Open. 2018;8:e021430.
78. Gordon MMAJH. Management of female genital mutilation : the Northwick Park Hospital experience - McCafrey - 1995 - BJOG: An International Journal of Obstetrics & Gynaecology - Wiley Online Library. 1995.
79. Foundations OS. Somalis in Leicester. New York, NY, USA: Open Society Foundations; 2014.
80. Fawcett RJ, Kernohan G. A retrospective analysis of 34 potentially missed cases of female genital mutilation in the emergency department. Emergenct Medicine Journal. 2017;35(10):587-9.
81. Ambuel B. Healthcare Can Change from Within: Sustained Improvement in the Healthcare Response to Intimate Partner Violence | SpringerLink. 2013.
82. Szilassy E, Drinkwater J, Hester M, Larkins C, Stanley N, Turner W, et al. Making the links between domestic violence and child safeguarding: an evidence-based pilot training for general practice. Health & Social Care in the Community. 2017;25(6):1722-32.
83. Drinkwater J, Stanley N, Szilassy E, Larkins C, Hester M, Feder G. Juggling confidentiality and safety: a qualitative study of how general practice clinicians document domestic violence in families with children. British Journal of General Practice. 2017;67(659):e437-e44.
84. Middleton J. Preventing violent extremism: the role of doctors. The Lancet. 2016;388(10057):2219-21.
85. Scamell M, Ghumman A. The experience of maternity care for migrant women living with female genital mutilation: A qualitative synthesis. Birth. 2019;46(1):15-23.
86. Salad J, Verdonk P, de Boer F, Abma TA. "A Somali girl is Muslim and does not have premarital sex. Is vaccination really necessary?" A qualitative study into the perceptions of Somali women in the Netherlands about the prevention of cervical cancer. International Journal for Equity in Health. 2015;14(1):68.
87. Widmark C, Levál A, Tishelman C, Ahlberg BM. Obstetric care at the intersection of science and culture: Swedish doctors' perspectives on obstetric care of women who have undergone female genital cutting. Journal of Obstetrics and Gynecology. 2010;30(6):553-8.
88. Foundations OS. Somalis in London. New York, NY, USA: Open Society Foundations; 2014. Contract No.: ISBN: 978-1-940983-07-3.
89. Norman K. FGM is always with us

Experiences, Perceptions and Beliefs of Women

Affected by Female Genital Mutilation in London

For peer review only

Results from a PEER study In: Hemmings J, Hussein E, Otoo-Oyortey N, editors. London: FORWARD; 2009. p. 55.
90. Harper Bulman K, McCourt C. Somali refugee women's experiences of maternity care in west London: A case study. Critical Public Health. 2010;12(4):365-80.
91. Wellock VK. Domestic abuse: Black and minority-ethnic women's perspectives. Midwifery. 2010;26(2):181-8.
92. Feder GS, Hutson M, Ramsay J, Taket AR. Women exposed to intimate partner violence: expectations and experiences when they encounter health care professionals: a meta-analysis of qualitative studies. Archives of Internal Medicine. 2006;166(1):22-37.
93. Johnsdotter S. Discrimination of Certain Ethnic Groups? Ethical Aspects of Implementing FGM Legislation in Sweden. Report. Malmo, Sweden: Faculty of Health and Society, University of Malmo; 2009 2009. Contract No.: FoU Rapport 2009:3.
94. Leye E, Powell RA, Nienhuis G, Claeys P, Temmerman M. Health care in Europe for women with genital mutilation. Health Care for Women International. 2006;27(4):362-78.
95. Paliwal P, Ali S, Bradshaw S, Hughes A, Jolly K. Management of type III female genital mutilation in Birmingham, UK: a retrospective audit. Midwifery. 2014;30(3):282-8.
96. Horwood J, Morden A, Bailey JE, Pathak N, Feder G. Assessing for domestic violence in sexual health environments: a qualitative study. Sexually Transmitted Infections. 2017;94(2):88-92.
97. Feder G, Davies RA, Baird K, Dunne D, Eldridge S, Griffiths C, et al. Identification and Referral to Improve Safety (IRIS) of women experiencing domestic violence with a primary care training and support programme: a cluster randomised controlled trial. The Lancet. 2011;378(9805):1788-95.
98. Creighton SM, Liao LM. Tackling female genital mutilation in the UK. BMJ : British Medical Journal. 2013;347:f7150.
99. Feder G, Centre for Academic Primary Care SoSaCM, University of Bristol, Bristol, United Kingdom, Wathen CN, Faculty of Information and Media Studies WU, London, Ontario, Canada, MacMillan HL, Department of Psychiatry and Behavioural Neurosciences MU, Hamilton, Ontario, Canada, et al. An Evidence-Based Response to Intimate Partner Violence: WHO Guidelines. JAMA. 2013;310(5):479-80.
100. Turner W, Hester M, Broad J, Szilassy E, Feder G, Drinkwater J, et al. Interventions to improve the response of professionals to children exposed to domestic violence and abuse: a systematic review. Child Abuse Review. 2017;26(1):19-39.
101. Ashby J, Richardson A, Brawley D, E H, editors. National survey of practice and experience of mandatory reporting of female genital mutilation (FGM) amongst sexual health care professionals. 4th Joint Conference of the British HIV Association, BHIVA with the British Association for Sexual Health and HIV, BASHH 2018; 2018; United Kingdom: HIV Medicine.
102. Plugge E, Adam S, El Hindi L, Gitau J, Shodunke N, Mohamed-Ahmed O. The prevention of female genital mutilation in England: what can be done? Journal of public health (Oxford, England). 2018;fdy128.
103. Rymer J, editor Female genital mutilation. RCOG World Congress 2015; 2015; Brisbane, Australia: BJOG: An International Journal of Obstetrics and Gynaecology.
104. Taket A, Nurse J, Smith K, Watson J, Shakespeare J, Lavis V, et al. Routinely asking women about domestic violence in health settings. BMJ: British Medical Journal. 2003;327(7416):673-6.
105. Bewley S, Kelly B, Darke K, Erskine K, Gerada C, Lohr P, et al. Mandatory submission of patient identifiable information to third parties: FGM now, what next? BMJ : British Medical Journal. 2015;351:h5146.
106. Murray L, Windsor C, Parker E, Tewfik O. The Experiences of African Women Giving Birth in Brisbane, Australia. Health Care for Women International. 2010;31(5):458-72.
107. Naftalin J, Bewley S. Mandatory reporting of FGM. British Journal of General Practice. 2015;65(638):450-1.
108. Dixon S. The FGM enhanced dataset: how are we going to discuss this with our patients? 2015.

For peer review only

109. Emam KE, Mercer J, Moreau K, Grava-Gubins I, Buckeridge D, Jonker E. Physician privacy concerns when disclosing patient data for public health purposes during a pandemic influenza outbreak. BMC Public Health. 2011;11(1):454.
110. Bismark MM, Mathews B, Morris JM, Thomas LA, Studdert DM. Views on mandatory reporting of impaired health practitioners by their treating practitioners: a qualitative study from Australia. BMJ Open. 2016;6:e011988.
111. Feng J-Y, Chen S-J, Wilk NC, Yang W-P, Fetzer S. Kindergarten teachers' experience of reporting child abuse in Taiwan: Dancing on the edge. Children and Youth Services Review. 2009;31(3):405-9.
112. Gallagher A, Wainwright P, Tompsett H, Atkins C. Findings from a Delphi exercise regarding conflicts of interests, general practitioners and safeguarding children: 'Listen carefully, judge slowly'. Journal of Medical Ethics. 2012;38(2):87-92.
113. Beran R. Mandatory notification of impaired doctors. Internal Medicine Journal. 2014;44(12a):1161-5.
114. Erskine K. Collecting data on female genital mutilation. BMJ : British Medical Journal. 2014;348:g3222.
115. Foster R, Olson-Dorff D, Reiland H, Budzak-Garza A. Commitment, confidence and concerns: Assessing health care professionals' child maltreatment reporting attitudes. Child Abuse & Neglect. 2017;67:54-63.
116. McTavish JR, Kimber M, Devries K, Colombini M, MacGregor JCD, Wathen CN, et al. Mandated reporters' experiences with reporting child maltreatment: a meta-synthesis of qualitative studies. BMJ Open. 2017;7(10):e013942.
117. Johansen REB, Ziyada MM, Shell-Duncan B, Kaplan AM, Leye E. Health sector involvement in the management of female genital mutilation/cutting in 30 countries. BMC Health Services Research. 2018;18(1):240.
118. Falkiner M, Thomson D, Day A. Teachers' Understanding and Practice of Mandatory Reporting of Child Maltreatment. Children Australia. 2017;42(1):38-48.
119. Talsma M, Bengtsson Boström K, Östberg A-L. Facing suspected child abuse – what keeps Swedish general practitioners from reporting to child protective services? Scandinavian Journal of Primary Health Care. 2013;33(1):21-6.
120. Lawson D, Niven B. The Impact of Mandatory Reporting Legislation on New Zealand Secondary School Students' Attitudes towards Disclosure of Child Abuse. International Journal of Children's Rights. 2015;23:491-528.
121. Casla K, Roderick P, Pollock AM. Disclosure of patients' data to the UK Home Office must stop. BMJ : British Medical Journal. 2017;358:j3613.
122. Hiam L. Grenfell survivors shouldn't be afraid to go to hospital. BMJ : British Medical Journal. 2017;358:j3292.
123. Hiam L, Steele S, McKee M. Creating a 'hostile environment for migrants': the British government's use of health service data to restrict immigration is a very bad idea. Health Economics, Policy and Law. 2018;13(2):107-17.

124. Creighton SM, Hodes D. Female genital mutilation: what every paediatrician should know. Archives of disease in childhood. 2016 Mar 1;101(3):267-71.

125. Simpson J, Robinson K, Creighton SM, Hodes D. Female genital mutilation: the role of health professionals in prevention, assessment, and management. Bmj. 2012 Mar 14;344:e1361.

For peer review only

Table 1 List of items to be included when reporting a realist synthesis

From: RAMESES publication standards: realist syntheses

TITLE

1 In the title, identify the document as a realist synthesis or review Yes

ABSTRACT

2 While acknowledging publication requirements and house style, abstracts should ideally contain brief details of: the study's background, review question or objectives; search strategy; methods of selection, appraisal, analysis and synthesis of sources; main results; and implications for practice. Yes

INTRODUCTION

3 Rationale for review Explain why the review is needed and what it is likely to contribute to existing understanding of the topic area. Yes

4 Objectives and focus of review State the objective(s) of the review and/or the review question(s). Define and provide a rationale for the focus of the review. Yes

METHODS

5 Changes in the review process Any changes made to the review process that was initially planned should be briefly described and justified. No changes made to the searching described by protocol. As part of our iterative searching, we actively undertook 2 update searches.

6 Rationale for using realist synthesis Explain why realist synthesis was considered the most appropriate method to use. Yes

7 Scoping the literature Describe and justify the initial process of exploratory scoping of the literature. Yes

8 Searching processes While considering specific requirements of the journal or other publication outlet, state and provide a rationale for how the iterative searching was done. Provide details on all the sources accessed for information in the review. Where searching in electronic databases has taken place, the details should include, for example, name of database, search terms, dates of coverage and date last searched. If individuals familiar with the relevant literature and/or topic area were contacted, indicate how they were identified and selected. Yes

9 Selection and appraisal of documents Explain how judgements were made about including and excluding data from documents, and justify these. Yes

10 Data extraction Describe and explain which data or information were extracted from the included documents and justify this selection. Yes

11 Analysis and synthesis processes Describe the analysis and synthesis processes in detail. This section should include information on the constructs analyzed and describe the analytic process. Yes

RESULTS

For peer review only

12 Document flow diagram Provide details on the number of documents assessed for eligibility and included in the review with reasons for exclusion at each stage as well as an indication of their source of origin (for example, from searching databases, reference lists and so on). You may consider using the example templates (which are likely to need modification to suit the data) that are provided. Yes

13 Document characteristics Provide information on the characteristics of the documents included in the review. Yes

14 Main findings Present the key findings with a specific focus on theory building and testing. We have presented the initial theory developed from scoping and presented the evolved programme theory

DISCUSSION

15 Summary of findings Summarize the main findings, taking into account the review's objective(s), research question(s), focus and intended audience(s). Yes

16 Strengths, limitations and future research directions Discuss both the strengths of the review and its limitations. These should include (but need not be restricted to) (a) consideration of all the steps in the review process and (b) comment on the overall strength of evidence supporting the explanatory insights which emerged. Yes

The limitations identified may point to areas where further work is needed.

17 Comparison with existing literature Where applicable, compare and contrast the review's findings with the existing literature (for example, other reviews) on the same topic. Yes

18 Conclusion and recommendations List the main implications of the findings and place these in the context of other relevant literature. If appropriate, offer recommendations for policy and practice. Yes

19 Funding Provide details of funding source (if any) for the review, the role played by the funder (if any) and any conflicts of interests of the reviewers. Yes

For peer review only
